# Supplementary material for: Validity and effectiveness of paediatric early warning systems and track and trigger tools for identifying and reducing clinical deterioration in hospitalised children: a systematic review
Source: BMJ Open. 2019 May 5;9(5):e022105. doi: 10.1136/bmjopen-2018-022105 (PMC6502038; doi:10.1136/bmjopen-2018-022105)
Supplement: Supplementary data [file bmjopen-2018-022105supp001.pdf]

Supplementary Table 1 – Details of search strategy

Database Search

The search was across a range of databases from their inception to January 2015 then an update was carried out in September 2016 and the second update May 2018.

A preliminary search strategy was developed using a set of key papers known to the group for Ovid Medline using both text words and Medical subject headings. The search strategy was modified according to the indexing systems of the other databases.

Databases and Database platform	Original search results January 2015	Update September 2016	Update May 2018
British Nursing Index (Proquest)	19	12	25
CINAHL (Cumulative Index of Nursing and Allied Health Literature) (Ebsco)	206	17	29
Cochrane Central Register of Controlled Trials (Wiley)	43	4	30
EMBASE (OVID)	1065	206	431
HMIC (Health Management Information Centre) (OVID)	70	1	75
Medline (OVID)	943	135	328
Medline in Process (OVID)	43	69	45
Scopus (Elsevier)	747	85	234
Web of Knowledge (Science Science Citation Indexes) (Thomson Reuter)	400	82	166
**Total**	**3536** *(prior to removing duplicates and irrelevant studies)*	**611** *(prior to removing duplicates and irrelevant studies)*	**1363** *(prior to removing duplicates and irrelevant studies)*

Supplementary search

Search Information

Supplementary search

NB. Restricted each of the below searches by dates: 01/01/2016 – 16/05/2018

Trials Registers	Hits January 2015	Update September 2016	Update June 2018
ClinicalTrials.gov<br>https://clinicaltrials.gov/	6	4	0
UK Clinical Trials Gateway<br>http://www.ukctg.nihr.ac.uk/default.aspx	3 (duplicates)	5 (1 duplicate)	0
The WHO trial search portal for studies worldwide:<br>http://apps.who.int/trialsearch	1 (duplicate)	0	0
Journal site	Hits		
Archives of Disease in Childhood<br>http://adc.bmj.com/	14	4	7
BMJ<br>http://www.bmj.com/theBMJ	1	0	1
BMJ Quality and safety<br>http://qualitysafety.bmj.com/	7	4	2
JAMA Pediatrics<br>http://archpedi.jamanetwork.com/journal.aspx	1	0	0
Journal of Critical Care<br>http://www.jccjournal.org/	3	1	0
Journal of Pediatrics (American)<br>http://www.jpeds.com/	1	0	2
Journal of Paediatrics and Child Health (Australian)<br>http://onlinelibrary.wiley.com/journal/10.1111/(ISSN)1440-1754	2	2	0

Lancet<br>http://www.thelancet.com/	0	0	0
New England Journal of Medicine<br>http://www.nejm.org/	0	0	0
Pediatrics<br>http://pediatrics.aappublications.org/	6	2	0
Pediatric Critical Care Medicine<br>http://journals.lww.com/pccmjournal/pages/default.aspx	14	6	3
Websites and organisations	HITS		
American Society of Anesthesiologists<br>https://www.asahq.org/	1	0	0
American Academy of Pediatrics<br>http://www.aap.org/en-us/Pages/Default.aspx	1		0
Association of Anaesthetists of Great Britain and Ireland<br>http://www.aagbi.org/	0	0	0
Australian Medical Council<br>http://www.amc.org.au/	1	0	0
Royal College of Paediatrics and Child Health<br>http://www.rcpch.ac.uk/	1	0	4
Paediatric Nursing Association Europe<br>http://www.rcn.org.uk/	9		0
European Federation of Critical Care Nursing Associations<br>http://www.efccna.org/	No Search Option	No Search Option	No Search Option
Royal Australasian College of Physicians (Division of Child Health)<br>https://www.racp.edu.au/page/paed-policy	0	0	0
Royal College of Physicians (inclusive of National Clinical Guideline Centre)<br>https://www.rcplondon.ac.uk/	2	0	0
The NHS Institute for Innovation and Improvement<br>http://www.institute.nhs.uk/	4	Site cease to exist	Site cease to exist
NICE: Eyes on Evidence	4	1	1

https://www.evidence.nhs.uk/about-evidence-services/bulletins-and-alerts/eyes-on-evidence			
**TOTAL**	82	30	20

Total = 112

Search Strategies

British Nursing Index
"Paediatric Early Warning" OR ("pediatric early warning" OR "pediatric rapid response") OR ("paediatric rapid response" OR "Bedside paediatric early warning") OR ("Pediatric Advanced Warning Score" OR "Paediatric Advanced Warning Score")

Cochrane Controlled Register of Trials (CENTRAL)
Last Saved: 16/05/2018 11:39:08.703
Description:

ID	Search
#1	"early warning score*"
#2	"early warning system*"
#3	"early warning tool*"
#4	"VitalPAC Early Warning Score"
#5	"activation criteria"
#6	"Rapid Response Team"
#7	"Rapid Response system*"
#8	"Track and trigger"
#9	"trigger tools"
#10	"calling criteria"
#11	"Alert criteria"

#12	"Rapid Response"
#13	#1 or #2 or #3 or #4 or #5 or #6 or #7 or #8 or #9 or #10 or #11 or #12
#14	pediatric* or paediatric* or infant* or child* or baby or toddler or babies or teen* or adolescent*
#15	#13 and #14
#16	"Pediatric Early Warning"
#17	"Paediatric Early Warning"
#18	"p?ediatric alert"
#19	"Pediatric Rapid Response"
#20	"Pediatric Advanced Warning Score*"
#21	"Paediatric Advanced Warning Score*"
#22	"infant early warning"
#23	"Bedside PEWS"
#24	"Bedside paediatric early warning"
#25	#16 or #17 or #18 or #19 or #20 or #21 or #22 or #23 or #24
#26	#15 or #25 Publication Year from 2016 to 2018

Cumulative Index to Nursing and Allied Health Literature (CINAHL) via EBSCO

	Search ID#	Search Terms
☐	S11	S7 OR S10
☐	S10	S1 AND S8
☐	S9	S2 AND S8
☐	S8	S3 AND S4
☐	S7	S5 OR S6
☐	S6	TX "infant early warning" OR TX "bedside PEWS" OR TX "Bedside paediatric early warning"
☐	S5	TX "p?ediatric early warning system" OR TX "P?ediatric Early Warning" OR TX "p?ediatric early warning score" OR TX "p?ediatric risk of mortality" OR TX "P?ediatric Rapid Response Team" OR TX "P?ediatric alert"
☐	S4	AB pediatric* or paediatric* or infant*1 or child* or baby or toddler or babies or teen* or adolescent*

☐ S3 TX "track-and-trigger" OR TX "VitalPAC Early Warning Score" OR TX "activation criteria". OR TX "trigger tool*" OR TX "Rapid Response" OR TX "activation criteria". OR TX "early warning" OR TX "Alert criteria" OR TX outreach N3 emergency

☐ S2 Detecting W3 deterioration

☐ S1 "early warning"

Database of Abstracts of Review of Effects (DARE)
(Paediatric early warning) OR (pediatric early warning) OR (Paediatric Rapid Response) IN DARE
(early warning) OR (track-and-trigger system) OR (Rapid Response) IN DARE
(emergency team) AND (early warning) IN DARE

Excerpta Medica Database (EMBASE)
Database: EMBASE <1947-Present>
Search Strategy:
----
1 ("early warning" adj5 scor*).ab,ti. (568)
2 ("early warning" adj5 system* adj5 (deteriorat* or mortality or death or outcome* or harm* or safety)).ab,ti. (51)
3 "acute illness severity".mp. (38)
4 early intervention/ and ((prevent* or reduc* or improv*) adj5 (deteriorat* or mortality or death or outcome* or harm* or safety)).ab,ti. (1185)
5 ("early medical intervention" adj5 (tool* or scor* or index* or indicator* or indice* or assessment* or guide* or instrument* or criteria or parameter* or deteriorat* or mortality or death or monitor* or outcome* or harm* or safety)).ab,ti. (10)
6 *"severity of illness index"/ and ((tool* or scor* or index* or indicator* or indice* or assessment* or instrument* or criteria or parameter*) adj5 ((prevent* or reduc* or improv*) adj5 (deteriorat* or mortality or death or outcome* or harm* or safety))).ab,ti. (3)
7 exp Health Status Indicators/ and ((tool* or scor* or index* or indicator* or indice* or assessment* or instrument* or criteria or parameter*) adj3 ((prevent* or reduc* or improv*) adj3 (deteriorat* or mortality or death or outcome* or harm* or safety))).ab,ti. (7)
8 rapid response team/ (849)
9 "alarm monitor"/ and (prevent* or reduc* or improv*).mp. (245)
10 ("clinical alarm" adj5 (prevent* or reduc* or improv*)).mp. (2)
11 (outreach adj3 emergency).tw. (46)
12 VitalPAC Early Warning Score.tw. (15)
13 medical emergency team.tw. (395)
14 Rapid Response Systems.mp. (140)

15 ("rapid response" adj5 (prevent* or reduc* or improv*)).tw. (191)
16 ("medical device" adj3 (prevent* or reduc* or improv*)).mp. (187)
17 (((Detecting or managing) adj3 deterioration) and warning).tw. (11)
18 track-and-trigger system.tw. (24)
19 (Track adj trigger).tw. (4)
20 (Track and trigger).tw. (241)
21 trigger tools.tw. (47)
22 ("alert criteria" or "activation criteria" or "calling criteria").tw. (209)
23 SBAR technique*.mp. (5)
24 (score adj3 severity of illness).tw. (393)
25 or/1-24 (4295)
26 limit 25 to (infant <to one year> or child <unspecified age> or preschool child <1 to 6 years> or school child <7 to 12 years> or adolescent <13 to 17 years>) (533)
27 P?ediatric Early Warning.mp. (120)
28 p?ediatric alert.tw. (7)
29 p?ediatric early warning systems.mp. (4)
30 p?ediatric risk of mortality.tw. (527)
31 P?ediatric Rapid Response Team.tw. (14)
32 Point-of-Care Systems/ and ((paediatric or pediatric) adj3 (improve or identify or detect* or outcome or early or critical or emergency)).tw. (23)
33 P?ediatric Advanced Warning Score.tw. (3)
34 neonatal early warning.tw. (1)
35 infant early warning.tw. (0)
36 p?ediatric rapid response.tw. (31)
37 Bedside paediatric early warning.tw. (5)
38 Bedside PEWS.tw. (7)
39 or/27-38 (707)
40 26 or 39 (1155)
41 limit 40 to human (1065)

Health Management Information Consortium (HMIC) database
Database: HMIC Health Management Information Consortium
Search Strategy:
----

1 ("early warning" adj5 scor*).ab,ti. (23)
2 ("early warning" adj5 system* adj5 (deteriorat* or mortality or death or outcome* or harm* or safety)).ab,ti. (6)
3 "acute illness severity".mp. (3)
4 "early medical intervention"/ and ((prevent* or reduc* or improv*) adj5 (deteriorat* or mortality or death or outcome* or harm* or safety)).ab,ti. (0)
5 ("early medical intervention" adj5 (tool* or scor* or index* or indicator* or indice* or assessment* or guide* or instrument* or criteria or parameter* or deteriorat* or mortality or death or monitor* or outcome* or harm* or safety)).ab,ti. (0)
6 Health Status Indicators.mp. and ((tool* or scor* or index* or indicator* or indice* or assessment* or instrument* or criteria or parameter*) adj3 ((prevent* or reduc* or improv*) adj3 (deteriorat* or mortality or death or outcome* or harm* or safety))).ab,ti. (0)
7 exp "Severity of illness index"/ and ((tool* or scor* or index* or indicator* or indice* or assessment* or instrument* or criteria or parameter*) adj5 ((prevent* or reduc* or improv*) adj5 (deteriorat* or mortality or death or outcome* or harm* or safety))).ab,ti. (0)
8 "activation criteria".ab,ti. (2)
9 exp Rapid response teams/ (39)
10 Clinical Alarms.mp. (0)
11 (outreach adj3 emergency).tw. (2)
12 VitalPAC Early Warning Score.tw. (0)
13 medical emergency team.tw. (15)
14 Rapid Response Systems.mp. (8)
15 Rapid Response Team.tw. (27)
16 ((Detecting or managing) adj3 deterioration).tw. (1)
17 track-and-trigger system.tw. (2)
18 (Track adj trigger).tw. (1)
19 (Track and trigger).tw. (8)
20 trigger tools.tw. (4)
21 Calling criteria.tw. (1)
22 Alert criteria.mp. (1)
23 Rapid response.tw. (111)
24 (score adj3 severity of illness).tw. (3)
25 or/1-24 (171)
26 (pediatric* or paediatric* or infant*1 or child* or baby or toddler or babies or teen* or adolescent*).mp. (40161)
27 25 and 26 (14)
28 p?ediatric alert.tw. (0)
29 p?ediatric early warning systems.mp. (1)
30 p?ediatric risk of mortality.tw. (4)

31 Pediatric Rapid Response Team.tw. (0)
32 Point-of-Care.mp. and ((paediatric or pediatric) adj3 (improve or identify or detect* or outcome or early or critical or emergency)).tw. (0)
33 Pediatric Advanced Warning Score.tw. (0)
34 neonatal early warning.tw. (0)
35 infant early warning.tw. (0)
36 paediatric rapid response.tw. (1)
37 pediatric rapid response.tw. (0)
38 Bedside paediatric early warning.tw. (0)
39 Bedside PEWS.tw. (0)
40 p?ediatric early warning.mp. (2)
41 care.mp. and ((paediatric or pediatric) adj3 (improve or identify or detect* or outcome or early or critical or emergency)).tw. [mp=title, other title, abstract, heading words] (57)
42 or/28-41 (59)
43 27 or 42 (70)

Medline
Database: Ovid MEDLINE(R) <1946 to January Week 2 2015>
Search Strategy:
----
1 ("early warning" adj5 scor*).ab,ti. (260)
2 ("early warning" adj5 system* adj5 (deteriorat* or mortality or death or outcome* or harm* or safety)).ab,ti. (24)
3 "acute illness severity".mp. (21)
4 "early medical intervention"/ and ((prevent* or reduc* or improv*) adj5 (deteriorat* or mortality or death or outcome* or harm* or safety)).ab,ti. (99)
5 ("early medical intervention" adj5 (tool* or scor* or index* or indicator* or indice* or assessment* or guide* or instrument* or criteria or parameter* or deteriorat* or mortality or death or monitor* or outcome* or harm* or safety)).ab,ti. (7)
6 exp Health Status Indicators/ and ((tool* or scor* or index* or indicator* or indice* or assessment* or instrument* or criteria or parameter*) adj3 ((prevent* or reduc* or improv*) adj3 (deteriorat* or mortality or death or outcome* or harm* or safety))).ab,ti. (166)
7 "Severity of Illness Index"/ and ((tool* or scor* or index* or indicator* or indice* or assessment* or instrument* or criteria or parameter*) adj5 ((prevent* or reduc* or improv*) adj5 (deteriorat* or mortality or death or outcome* or harm* or safety))).ab,ti. (274)
8 exp Hospitals/ and ((Detecting or managing) adj3 deterioration).tw. (2)
9 ("medical device" adj3 (prevent* or reduc* or improv*)).mp. (58)
10 ("alert criteria" or "activation criteria" or "calling criteria").tw. (121)

11 Hospital Rapid Response Team/ (334)
12 Clinical Alarms/ (332)
13 (outreach adj3 emergency).tw. (32)
14 VitalPAC Early Warning Score.tw. (10)
15 medical emergency team.tw. (247)
16 Rapid Response Systems.mp. (87)
17 Rapid Response Team.tw. (185)
18 (((Detecting or managing) adj3 deterioration) and warning).tw. (8)
19 track-and-trigger system.tw. (14)
20 (Track adj trigger).tw. (2)
21 (Track and trigger).tw. (137)
22 trigger tools.tw. (22)
23 SBAR technique*.mp. (3)
24 ("rapid response" adj5 (prevent* or reduc* or improv*)).tw. (117)
25 (score adj3 severity of illness).tw. (243)
26 or/1-25 (2286)
27 limit 26 to (humans and "all child (0 to 18 years)") (453)
28 P?ediatric Early Warning.mp. (38)
29 p?ediatric alert.tw. (5)
30 p?ediatric early warning systems.mp. (3)
31 p?ediatric risk of mortality.tw. (400)
32 P?ediatric Rapid Response Team.tw. (6)
33 Point-of-Care Systems/ and ((paediatric or pediatric) adj3 (improve or identify or detect* or outcome or early or critical or emergency)).tw. (79)
34 P?ediatric Advanced Warning Score.tw. (2)
35 neonatal early warning.tw. (0)
36 infant early warning.tw. (0)
37 p?ediatric rapid response.tw. (20)
38 Bedside paediatric early warning.tw. (2)
39 Bedside PEWS.tw. (2)
40 or/28-39 (542)
41 27 or 40 (943)

Scopus

(TITLE-ABS-KEY ("Paediatric Early Warning" OR "Pediatric Early Warning" OR "Pediatric Advanced Warning Score" OR "Paediatric Advanced Warning Score" OR "neonatal early warning" OR "infant early warning" OR "pediatric rapid response" OR "Paedatric rapid response")) OR (((TITLE-ABS-KEY ("early warning" W/5 scor*)) OR (TITLE-ABS-KEY ("Rapid Response")) OR (TITLE-ABS-KEY ("track-and-trigger system")) OR (TITLE-ABS-KEY ("track and trigger")) OR (TITLE-ABS-KEY ("trigger tool*")) OR (TITLE-ABS-KEY ("alert criteria")) OR (TITLE-ABS-KEY ("activation criteria")) OR (TITLE-ABS-KEY ("VitalPAC Early Warning Score"))) AND (TITLE-ABS-KEY (pediatric* OR paediatric* OR infant* OR child* OR baby OR toddler OR babies OR teen* OR adolescent*))) AND (LIMIT-TO (SUBJAREA , "MEDI") OR LIMIT-TO (SUBJAREA , "NURS") OR LIMIT-TO (SUBJAREA , "NEUR"))

Web of Science

# 19	400	#17 OR #1<br>**Refined by:** [excluding] **WEB OF SCIENCE CATEGORIES:** (PARASITOLOGY OR PUBLIC ENVIRONMENTAL OCCUPATIONAL HEALTH OR BIOCHEMISTRY MOLECULAR BIOLOGY OR OPTICS OR HEALTH CARE SCIENCES SERVICES OR MYCOLOGY OR MANAGEMENT OR LINGUISTICS OR INSTRUMENTS INSTRUMENTATION OR MICROBIOLOGY OR INFORMATION SCIENCE LIBRARY SCIENCE OR MATHEMATICAL COMPUTATIONAL BIOLOGY OR GERIATRICS GERONTOLOGY OR ENGINEERING BIOMEDICAL OR FOOD SCIENCE TECHNOLOGY OR ENVIRONMENTAL STUDIES OR ENGINEERING ENVIRONMENTAL OR ENGINEERING ELECTRICAL ELECTRONIC OR HEALTH POLICY SERVICES OR TOXICOLOGY OR EDUCATION EDUCATIONAL RESEARCH OR NUTRITION DIETETICS OR SUBSTANCE ABUSE OR ECONOMICS OR MEDICINE RESEARCH EXPERIMENTAL OR STATISTICS PROBABILITY OR DEVELOPMENTAL BIOLOGY OR MEDICAL INFORMATICS OR SOCIOLOGY OR DENTISTRY ORAL SURGERY MEDICINE OR PSYCHOLOGY EXPERIMENTAL OR COMPUTER SCIENCE ARTIFICIAL INTELLIGENCE OR METEOROLOGY ATMOSPHERIC SCIENCES OR CHEMISTRY ANALYTICAL OR MEDICAL LABORATORY TECHNOLOGY OR CELL BIOLOGY OR DEMOGRAPHY OR BUSINESS FINANCE OR COMPUTER SCIENCE INTERDISCIPLINARY APPLICATIONS OR AUDIOLOGY SPEECH LANGUAGE PATHOLOGY OR PSYCHOLOGY DEVELOPMENTAL OR COMPUTER SCIENCE INFORMATION SYSTEMS OR PLANNING DEVELOPMENT)<br>Indexes=SCI-EXPANDED, SSCI, CPCI-S, CPCI-SSH Timespan=1900-2015
# 18	499	#17 OR #1<br>Indexes=SCI-EXPANDED, SSCI, CPCI-S, CPCI-SSH Timespan=1900-2015
#	487	#16 AND #15

17		Indexes=SCI-EXPANDED, SSCI, CPCI-S, CPCI-SSH Timespan=1900-2015
# 16	8,044	#14 OR #13 OR #12 OR #11 OR #10 OR #9 OR #8 OR #7 OR #6 OR #5 OR #4 OR #3 OR #2<br>Indexes=SCI-EXPANDED, SSCI, CPCI-S, CPCI-SSH Timespan=1900-2015
# 15	1,689,232	**TOPIC:** ((pediatric* OR paediatric* OR infant* OR child* OR baby OR toddler OR babies OR teen* OR adolescent*))<br>Indexes=SCI-EXPANDED, SSCI, CPCI-S, CPCI-SSH Timespan=1900-2015
# 14	130	**TOPIC:** ("Severity of Illness Index" and ((tool* or scor* or index* or indicator* or indice* or assessment* or instrument* or criteria or parameter*) SAME ((prevent* or reduc* or improv*) SAME (deteriorat* or mortality or death or outcome* or harm* or safety))))<br>Indexes=SCI-EXPANDED, SSCI, CPCI-S, CPCI-SSH Timespan=1900-2015
# 13	63	**TOPIC:** (("early medical intervention" SAME (tool* or scor* or index* or indicator* or indice* or assessment* or guide* or instrument* or criteria or parameter* or deteriorat* or mortality or death or monitor* or outcome* or harm* or safety)))<br>Indexes=SCI-EXPANDED, SSCI, CPCI-S, CPCI-SSH Timespan=1900-2015
# 12	28	**TOPIC:** ("early medical intervention" and ((prevent* or reduc* or improv*) SAME (deteriorat* or mortality or death or outcome* or harm* or safety)))<br>Indexes=SCI-EXPANDED, SSCI, CPCI-S, CPCI-SSH Timespan=1900-2015
# 11	1,206	**TOPIC:** ("early warning" SAME system* SAME (deteriorat* or mortality or death or outcome* or harm* or safety))<br>Indexes=SCI-EXPANDED, SSCI, CPCI-S, CPCI-SSH Timespan=1900-2015
# 10	2	**TOPIC:** ("SBAR technique")<br>Indexes=SCI-EXPANDED, SSCI, CPCI-S, CPCI-SSH Timespan=1900-2015
# 9	7	**TOPIC:** ("VitalPAC Early Warning Score")<br>Indexes=SCI-EXPANDED, SSCI, CPCI-S, CPCI-SSH Timespan=1900-2015
# 8	123	**TOPIC:** ("activation criteria")<br>Indexes=SCI-EXPANDED, SSCI, CPCI-S, CPCI-SSH Timespan=1900-2015
# 7	16	TS=("alert criteria")<br>Indexes=SCI-EXPANDED, SSCI, CPCI-S, CPCI-SSH Timespan=1900-2015
# 6	159	TS=("trigger tool*")<br>Indexes=SCI-EXPANDED, SSCI, CPCI-S, CPCI-SSH Timespan=1900-2015
#	45	TS=("track and trigger")

5		Indexes=SCI-EXPANDED, SSCI, CPCI-S, CPCI-SSH Timespan=1900-2015
# 4	15	TS=("track-and-trigger system")<br>Indexes=SCI-EXPANDED, SSCI, CPCI-S, CPCI-SSH Timespan=1900-2015
# 3	6,100	TS=("Rapid Response")<br>Indexes=SCI-EXPANDED, SSCI, CPCI-S, CPCI-SSH Timespan=1900-2015
# 2	604	TS=("early warning" SAME scor*)<br>Indexes=SCI-EXPANDED, SSCI, CPCI-S, CPCI-SSH Timespan=1900-2015
# 1	88	TS=("Paediatric Early Warning" OR "Pediatric Early Warning" OR "Pediatric Advanced Warning Score" OR "Paediatric Advanced Warning Score" OR "neonatal early warning" OR "infant early warning" OR "pediatric rapid response" OR "Paedatric rapid response")<br>Indexes=SCI-EXPANDED, SSCI, CPCI-S, CPCI-SSH Timespan=1900-2015

PUMA Supplementary searches

Search terms to use:

"Pediatric Early warning"
"Paediatric Early warning"
“Pediatric Rapid Response Team”
“Paediatric Rapid Response Team”
PEWS
“Paediatric trigger tools”
“Pediatric trigger tools”
