# Supplementary material for: Validity and effectiveness of paediatric early warning systems and track and trigger tools for identifying and reducing clinical deterioration in hospitalised children: a systematic review
Source: BMJ Open. 2019 May 5;9(5):e022105. doi: 10.1136/bmjopen-2018-022105 (PMC6502038; doi:10.1136/bmjopen-2018-022105)
Supplement: Supplementary data [file bmjopen-2018-022105supp002.pdf]

Supplementary Table 2 - PICOS criteria for inclusion of studies

Question 1 – development / validation studies

**Parameter**	**Inclusion criteria**	**Exclusion criteria**
Patients	Children aged 0-18 who are in-patients in a hospital	Adult patients; children in emergency departments or neonatal unit
Intervention	Development or validation of a PTTT	Acuity or triage tools, tools developed for use in emergency departments
Comparator	Not applicable	
Outcomes	Mortality and critical events including: arrests, code calls, transfer to higher level of care (e.g., ICU/HDU), senior review, RRT/MET activation, acuity at PICU admission and critical interventions on the ward or PICU	
Study design	Chart or case reviews; cohort studies; case-control studies, observational studies	Reviews, editorials or opinion pieces

Question 2 – effectiveness studies

**Parameter**	**Inclusion criteria**	**Exclusion criteria**
Patients	Children aged 0-18 who are in-patients in a hospital	Adult patients<br>Children in emergency departments or neonatal unit
Intervention	Implementation of any ‘paediatric early warning system’ intervention (with or without a PTTT) – including implementing a new PTTT, RRT/MET implementation, educational initiatives or communications tools aimed at improving identification of deteriorating in-patients	Acuity or triage tools, tools developed for use in emergency departments, interventions whose purpose was not identification of deteriorating in-patients
Comparator	Not applicable	
Outcomes	Mortality and critical events including: arrests, code calls, transfer to higher level of care (e.g., ICU/HDU), senior review, RRT/MET activation, acuity at PICU admission and critical interventions on the ward or PICU	
Study design	Randomised controlled trials, non-randomised controlled trials, before-after studies (controlled or uncontrolled); interrupted time series studies	Reviews, editorials or opinion pieces
