# Supplementary material for: Validity and effectiveness of paediatric early warning systems and track and trigger tools for identifying and reducing clinical deterioration in hospitalised children: a systematic review
Source: BMJ Open. 2019 May 5;9(5):e022105. doi: 10.1136/bmjopen-2018-022105 (PMC6502038; doi:10.1136/bmjopen-2018-022105)
Supplement: Supplementary data [file bmjopen-2018-022105supp003.pdf]

Supplementary Table 3 – Template Quality Assessment Forms

QUALITY ASSESSMENT FOR DEVELOPMENT AND VALIDATION STUDIES

Criteria		Yes (2)	Partial (1)	No (0)	N/A	Score
**1**	Is the hypothesis / aim / objective of the study clearly described?	Easily identified in introduction / method.	Vague / incomplete or found in other parts of paper (than introduction/method)	Aim / Objective no reported		
**2**	Was the score developed comprehensively?	Evidence base / Expert opinion / Delphi method	Decided within research team	No info / unclear		
**3**	Are the characteristics of the patients in the study clearly described?	Reproducible criteria used to categorise participants	Poorly define criteria / incomplete information	No baseline / demographic info		
**4**	Is the study design well described and appropriate?	Well described, easy to find in paper	Design not clearly described / design only partially answers the question	Design poorly described or does not answer study question		
**5**	Are the study sample representative of the intended population?	A full description of the target population is given with the sample selected in a non-biased manner	Sample selected from a known population however, selection strategy likely introduces bias but not enough to seriously distort results	Sample recruited from an unknown population in an opportunistic fashion		
**6**	Are population characteristics controlled for and adequately described?	Appropriate control at design/analysis stage	Incomplete control/description or not considered but unlikely to seriously influence results	Not controlled for and likely to seriously influence results		
**7**	Was compliance/use of the PEWS reliable?	Compliance / use was well described and reliably implemented	Compliance / use was not well described or not reliably implemented	Compliance / use was not reported		
**8**	Was consideration given for data collected at different times / sites	Well described reason why data was collected at different time points	Data was collected at different times due to specific opportunity	No explanation for data collection at different time points	Data was collected at the same time point	
**9**	Are the main findings clearly described?	Simple outcome data reported for all major findings	Incomplete or inappropriate descriptive statistics	No/inadequate descriptive statistics		
**10**	Are methods of analysis adequately described and appropriate?	Described and appropriate	Not reported but probably appropriate or some tests appropriate, some not	Methods not described and cannot be determined		
**11**	Are the conclusions supported by the results	All conclusions supported by data	Some of the major conclusions are supported by the data; some are not or speculative interpretations are not indicated as such	None/few of major conclusions supported by the data		
**12**	How was missing data handled	Missing data was reported and handled appropriately	Missing data was reported but unable to determine how it was handled or it wasn't handled appropriately	Missing data was not reported	No missing data	
					**Total**	

MAX. Score: 24

QUALITY ASSESSMENT FOR EFFECTIVENESS STUDIES

Criteria		Yes (2)	Partial (1)	No (0)	N/A	Score
1	Is the hypothesis / aim / objective of the study clearly described?	Easily identified in introduction / method.	Vague / incomplete or found in other parts of paper (than introduction/method)	Aim / Objective no reported		
2	Was the score developed comprehensively?	Evidence base / Expert opinion / Delphi method	Decided within research team	No info / unclear		
3	Are the characteristics of the patients in the study clearly described?	Reproducible criteria used to categorise participants	Poorly define criteria / incomplete information	No baseline / demographic info		
4	Is the study design well described and appropriate?	Well described, easy to find in paper	Design not clearly described / design only partially answers the question	Design poorly described or does not answer study question		
5	Are the study sample representative of the intended population?	A full description of the target population is given with the sample selected in a non-biased manner	Sample selected from a known population however, selection strategy likely introduces bias but not enough to seriously distort results	Sample recruited from an unknown population in an opportunistic fashion		
6	Was the PEWS well implemented?	Implementation was well reported and appropriately applied	Implementation was not well reported or not appropriate	No info / unclear		
7	Are population characteristics controlled for and adequately described?	Appropriate control at design/analysis stage	Incomplete control/description or not considered but unlikely to seriously influence results	Not controlled for and likely to seriously influence results		
8	Was compliance/use of the PEWS reliable?	Compliance / use was well described and reliably implemented	Compliance / use was not well described or not reliably implemented	Compliance / use was not reported		
9	Was consideration given for data collected at different times / sites	Well described reason why data was collected at different time points	Data was collected at different times due to specific opportunity	No explanation for data collection at different time points	Data was collected at the same time point	
10	Are the main findings clearly described?	Simple outcome data reported for all major findings	Incomplete or inappropriate descriptive statistics	No/inadequate descriptive statistics		
11	Are methods of analysis adequately described and appropriate?	Described and appropriate	Not reported but probably appropriate or some tests appropriate, some not	Methods not described and cannot be determined		
12	Are the conclusions supported by the results	All conclusions supported by data	Some of the major conclusions are supported by the data; some are not or speculative interpretations are not indicated as such	None/few of major conclusions supported by the data		
13	How was missing data handled	Missing data was reported and handled appropriately	Missing data was reported but unable to determine how it was handled or it wasn't handled appropriately	Missing data was not reported	No missing data	
					Total	

MAX. Score: 26
