# Supplementary material for: Validity and effectiveness of paediatric early warning systems and track and trigger tools for identifying and reducing clinical deterioration in hospitalised children: a systematic review
Source: BMJ Open. 2019 May 5;9(5):e022105. doi: 10.1136/bmjopen-2018-022105 (PMC6502038; doi:10.1136/bmjopen-2018-022105)
Supplement: Supplementary data [file bmjopen-2018-022105supp004.pdf]

Supplementary Table 4 –Validation papers excluded from analysis

PTTT	First author, year	Country	Study population	Study design	Number of centres	PTTT used in practice?	Internal / external validation study?	Outcome measures	Sample size	Score or trigger?	Study overview and reason for exclusion from validation results	Quality score (max = 24)
Modified Brighton PEWS (a)	Garlick 2013[20]	US	All in-patients (MET calls only)	Case-control study (retrospective)	1	N	Ext	Transfer to PICU	267 (116 cases)	S	Describes review of MET calls (n=267) to evaluate predictive ability of Modified Brighton PEWS tool for identifying children requiring transfer to PICU (n=116). Results presented in terms of association between PEWS and odds of transfer to higher level of care – no evaluation of performance characteristics such as AUROC, sensitivity or specificity.	8
	Medar 2015[21]	Unclear	RRT calls only	Chart review (retrospective)	1	NR	Ext	RRT call	61	S	Describes retrospective review of RRT calls (n=61) to evaluate Modified Brighton PEWS at time of admission and time of RRT call. Report higher median PEWS score for patients at time of RRT call compared to admission. No evaluation of performance characteristics such as AUROC, sensitivity or specificity.	6
Texas Children's Hospital (TCH) PAWS	Bell 2013[22]	US	General medical ward & two specialist units	Chart review (retrospective)	1	Y	Int	Other validated scales (e.g., Glasgow Coma Scale)	150	S	Describes development and implementation of the TCH PAWS tool in three wards of a specialist paediatric unit in the US. TCH PAWS amended locally from the Brighton PEWS. Reports on internal reliability (correlation coefficients between 3 categories of the score) and inter-rater reliability of scoring among nurses. Also compares scores on sub-categories to other measures, e.g., the Behavioural sub-score is compared to the Glasgow Coma Scale. No evaluation of performance characteristics such as AUROC, sensitivity or specificity.	12
Cardiac Children's Hospital Early Warning Score (C-CHEWS)	McLellan 2013[23]	US	Cardiac unit	Tool development	1	Y	Int	Cardiac ICU transfer	27	S	Describes the development and implementation of a modified version of the Children's Hospital Early Warning score for cardiac patients. Results focus on tool modification and implementation challenges – no evaluation of performances characteristics such as AUROC, sensitivity or specificity. Validation of the tool described in a separate paper.	9
Burn-specific PEWS	Rahman 2014[24]	US	Specialist burn unit	Chart review (retrospective)	1	Y	Int	Burn injuries	50	S	Conference abstract only. Describes development and implementation of a modified version of the Brighton PEWS, for use with in-patients with burn injuries. Analysis of 50 randomly selected charts – results focus on compliance with scoring and relationship between PTTT score and extent of burn injuries. No evaluation of performance characteristics such as AUROC, sensitivity or specificity.	13

Bedside Paediatric Early Warning Score (PEWS)	Hopkins 2013[25]	US	All in-patients (code blue and RRT calls only)	Chart review (retrospective)	1	N	Ext	PICU transfer and critical intervention in PICU among RRT and code calls	113 (64 cases)	S	Conference abstract only. Describes retrospective chart review of code blue and RRT calls over a year – Bedside PEWS scores calculated and comparisons drawn between patients eventually transferred to PICU and those who stayed on ward. Preliminary analysis given in terms of mean PEWS scores for different groups – no evaluation of performance characteristics such as AUROC, sensitivity or specificity.	6
	Gawronski 2013[26]	Italy	Bone marrow transplant unit	Case-control study (retrospective)	1	N	Ext	Urgent PICU transfer, PICU consult or death	21 (11 cases)	S	Conference abstract only. Describes case-control study evaluating Bedside PEWS in an Italian bone marrow transplant unit, in relation to urgent PICU transfers or consultations. Preliminary analysis only – comparison of mean PTTT scores for cases and controls. No evaluation of performance characteristics such as AUROC, sensitivity or specificity.	6
Bristol Paediatric Early Warning Tool (PEWT)	Haines 2006[12]	UK	All in-patients	Chart review (retrospective)	1	Y	Int	Transfer to PICU or HDU	360 (180 cases)	T	Describes development and piloting of the Bristol PEWT in a UK tertiary centre. Only included children who would have triggered the pilot version of the tool (n=360) and then identified PICU or HDU transfers from this population. Paper presents specificity and sensitivity outcomes but they are incorrectly calculated, so results not included in analysis.	9
Modified Bristol PEWT (a)	Sefton 2014[27]	UK	All in-patients	Chart review (retrospective)	1	Y	Int	Transfer to PICU, cardiac / respiratory arrest or unexpected death	Unclear	T	Conference abstract only. Describes a retrospective review of 5 years of data from locally implemented PTTT in a UK tertiary centre, presenting a multiple regression model identifying seven components (including age) most strongly associated with subsequent adverse event if triggered. Of the six clinical elements, all were associated with increased odds of an adverse event, except nurse concern which was significantly associated with decreased odds of an adverse event. No evaluation of overall PTTT performance characteristics such as AUROC, sensitivity or specificity.	10

All studies conducted in a specialist / tertiary centre.

Studies classified as internal validation if the setting for the study was the same hospital and same research team as those who developed the score. Studies classified as external validation if the score was tested in a different centre and by a different research team to those who developed it.

AUROC, area under the receiver operator characteristic curve; Ext, external validation ; HFNC, high flow nasal cannula; Int, Internal validation; NPV, negative predictive value; PHDU, paediatric high-dependency unit; PICU, paediatric intensive care unit ; PPV, positive predictive value; PTTT, paediatric track and trigger tool; RRT, rapid response team; S, score; T, trigger; UK, United Kingdom; US, United States;
