# Supplementary material for: Validity and effectiveness of paediatric early warning systems and track and trigger tools for identifying and reducing clinical deterioration in hospitalised children: a systematic review
Source: BMJ Open. 2019 May 5;9(5):e022105. doi: 10.1136/bmjopen-2018-022105 (PMC6502038; doi:10.1136/bmjopen-2018-022105)
Supplement: Supplementary data [file bmjopen-2018-022105supp005.pdf]

Supplementary Table 5 – Effectiveness papers excluded from analysis

First author, year	Intervention: Implemented a new PTTT	Implemented new RRT / MET	Modified escalation process	Staff training / education	PTTT	Country	Number of centres	Specialist unit?	Existing RRT / MET?	Population	Study design	Study duration in months (before & after intervention)	Description and reason for excluding from analysis	Quality score (max = 26)
Mistry 2006[51]	✓	✓		✓	Paediatric Rapid Response Team activation criteria*	US	1	Y	N	All in-patients	Uncontrolled before-after study (prospective)	11 (6 before, 5 after)	Describes implementation of a PRRT with calling criteria (not defined). Looked at impact on mortality, cardiac arrests and PICU outcomes among PICU transfers. Reports absolute decreases in numbers of deaths and arrests post-intervention, but no denominator data provided or further statistical details given.	3
Demmel 2010[52]	✓				Modified Brighton PEWS (e)	US	1	Y	Y	Haematology / oncology patients	Uncontrolled before-after study (prospective)	Unclear (unclear, 8 after)	Implemented a locally modified version of the Brighton PEWS in a specialist haematology / oncology unit. Discusses challenges in the development and implementation of the tool. Refers to number of days between cardiopulmonary arrests being 299 immediately before implementation, and 1,053 days eight months after implementation – however, no denominator data or further statistical details given.	8
Sandhu 2010[53]		✓			Unclear	UK	1	Y	N	Unclear	Uncontrolled before-after study (retrospective)	Unclear (unclear, 3 months)	Conference abstract only. Reported implementing an ‘outreach response team’ alongside an existing ‘paediatric early warning tool’ (unclear which tool) in a UK tertiary centre. Reference to comparable triggering rate of PTTT before (28% of patients) and after (28% of patients) piloting the outreach team, and 2 arrests before piloting, and 0 after – but no denominator data or further statistical details given.	8
Randhawa 2011[54]	✓		✓	✓	Brighton PEWS	US	1	Y	Y	All in-patients	Uncontrolled before-after study (prospective)	Unclear	Describes implementation of the Brighton PEWS in a specialist paediatric centre. Details various cycles of change during implementation of the tool across different wards, and efforts at staff education. Reports reduction in rate of cardiopulmonary arrests post-intervention, but no absolute numbers, denominator data or further statistical details given.	12

Camacho 2011[55]	✓				Modified Brighton PEWS (a) †	US	1	Y	NR	Cardiac and renal patients	Uncontrolled before-after study (prospective)	8 (3 before, 5 after)	Conference abstract only. Reported piloting and modifying Tucker's modified Brighton PEWS for specialist cardiac and renal population. Unclear if RRT/MET in place. Referred to there being 5 code calls in the quarter (3 months) before implementation, and 0 in the following 5 months. However, no denominator data or further statistical details given.	8
Heyden 2012[56]	✓	✓			Paediatric Rapid Response Team activation criteria*	US	1	Y	N	All in-patients	Uncontrolled before-after study (retrospective)	72 (24 before, 48 after)	Conference abstract only. Describes implementation of an RRT in a US tertiary centre, with an associated 'broad calling criteria' (limited details given). Reports number of cardiac arrests on ward and PICU before and after intervention, and refers to increase in RRT calls over time. No denominator data or further statistical details given.	7
Somberg 2013	✓	✓			Unclear	US	1	N	N	All in-patients	Uncontrolled before-after study (unclear)	Unclear	Conference abstract only. Reported developing and implementing a PTTT (tool not named) and RRT for a paediatric unit in a community hospital. Reference to no intubation or code calls since intervention, but no pre-intervention comparison, time frames, denominator data or further statistical details given.	2
Norville 2013[57]	✓				Texas Children's Hospital (TCH) Paediatric Advanced Warning Score (PAWS)†	US	1	Y	Y	Bone marrow transplant patients	Uncontrolled before-after study (unclear)	23 (12 before, 11 after)	Conference abstract only. Describes implementation of TCH PAWS, with amended algorithm for specialist bone marrow transplant unit. Looked at impact on code calls and RRT calls – refers to 3 code calls and 18 RRT calls pre-intervention, compared to 0 codes and 25 RRT calls post-intervention. No denominator data or further statistical details given.	5
Ambati 2014[58]				✓	Not applicable	US	1	Y	Y	Unclear	Uncontrolled before-after study (unclear)	48 (12 before, 36 after)	Conference abstract only. Reported effect of implementing a "simulation based curriculum" for clinical staff on subsequent RRT utilisation. Reference to increase in RRT calls year on year post implementation, but no denominator data or further statistical details given.	3
Ocholi 2014[59]	✓				Bedside Paediatric Early Warning Score (PEWS)	UK	1	Y	N	Unclear	Uncontrolled before-after study (unclear)	12 months (6 before, 6 after)	Conference abstract only. Describes implementation of Bedside PEWS in a UK tertiary centre. Looked at impact of intervention on ward outcomes and outcomes of children transferred to PICU. Reference to impact of tool on number of 'adverse incidents' (not defined) on the ward and median length of stay in PICU among PICU transfers, but no denominator data or further statistical details given.	6
Fenix 2016[39]	✓			✓	Unclear	US	1	Y	NR	Two general paediatric wards	Uncontrolled before-after study (retrospective)	46 months (16 before, 30 after)	Conference abstract only. Describes implementation of a 'Situational Awareness' tool, with integrated PTTT (unclear which tool) in a tertiary centre. Retrospective review of rates of Critical Deterioration (CD) events on two of seven general paediatric wards. Reports a significant decrease in trend and trajectory of CD events post-implementation, but no event numbers, denominator data or further statistical details given.	6

* Indicates PTTT not fully described or validated in the published literature

† PTTT modified by local team, but exact modifications not described

MET, medical emergency team; PICU, paediatric intensive care unit; PTTT, paediatric track and trigger tool; RRT, rapid response team; UK, United Kingdom; US, United States.
