# Supplementary material for: Validity and effectiveness of paediatric early warning systems and track and trigger tools for identifying and reducing clinical deterioration in hospitalised children: a systematic review
Source: BMJ Open. 2019 May 5;9(5):e022105. doi: 10.1136/bmjopen-2018-022105 (PMC6502038; doi:10.1136/bmjopen-2018-022105)
Supplement: Author's manuscript [file bmjopen-2018-022105.draft_revisions.pdf]

BMJ Open is committed to open peer review. As part of this commitment we make the peer review history of every article we publish publicly available.

When an article is published we post the peer reviewers' comments and the authors' responses online. We also post the versions of the paper that were used during peer review. These are the versions that the peer review comments apply to.

The versions of the paper that follow are the versions that were submitted during the peer review process. They are not the versions of record or the final published versions. They should not be cited or distributed as the published version of this manuscript.

BMJ Open is an open access journal and the full, final, typeset and author-corrected version of record of the manuscript is available on our site with no access controls, subscription charges or pay-per-view fees (http://bmjopen.bmj.com).

If you have any questions on BMJ Open's open peer review process please email info.bmjopen@bmj.com

BMJ Open

Systematic review of paediatric track and trigger tools for identifying clinical deterioration of children in hospital: their development, validation and effectiveness

Journal:	*BMJ Open*
Manuscript ID	bmjopen-2018-022105
Article Type:	Research
Date Submitted by the Author:	06-Feb-2018
Complete List of Authors:	Trubey, Rob; Cardiff University, Centre for Trials Research<br>Huang, Chao; Cardiff University, Centre for Trials Research<br>Lugg-Widger, Fiona; Cardiff University, Centre for Trials Research<br>Hood, Kerenza; Cardiff University, Centre for Trials Research<br>Allen, Davina; Cardiff University, School of Healthcare Sciences<br>Edwards, Dawn; Morriston Hospital, Department of Paediatrics<br>Lacy, David; Wirral Unviersity Teaching Hospital<br>Lloyd, Amy; Cardiff University, Centre for Trials Research<br>Mann, Mala; Cardiff University, University Library Services<br>Mason, Brendan; Public Health Wales<br>Oliver, Alison; Noah's Ark Children's Hospital for Wales, Department of Paediatric Intensive Care<br>Roland, Damian; Paediatric Emergency Medicine Leicester Academic (PEMLA) Group, Emergency Department<br>Sefton, G.; Alder Hey Childrens NHS Fdn Trust,<br>Skone, Richard; Noah's Ark Children's Hospital for Wales, Department of Paediatric Intensive Care<br>Thomas-Jones, Emma; Cardiff University, Centre for Trials Research<br>Tume, Lyvonne N.; University of the West of England Bristol, Faculty of Health and Applied Sciences (HAS)<br>Powell, Colin; Children's Hospital for Wales, Paediatrics
Keywords:	Quality in health care < HEALTH SERVICES ADMINISTRATION & MANAGEMENT, Paediatric intensive & critical care < INTENSIVE & CRITICAL CARE, PAEDIATRICS

SCHOLARONE™
Manuscripts

Systematic review of paediatric track and trigger tools for identifying clinical deterioration of children in hospital: their development, validation and effectiveness

Authors: Rob Trubey[1]†, Chao Huang[1], Fiona Lugg-Widger[1], Kerenza Hood[1], Davina Allen[2], Dawn Edwards[3], David Lacy[4], Amy Lloyd[1], Mala Mann[5], Brendan Mason[6], Alison Oliver[7], Damian Roland[8], Gerri Sefton[9], Richard Skone[7], Emma Thomas-Jones[1], Lyvonne Tume[9], Colin Powell[10]

1 Centre for Trials Research, Cardiff University, Heath Park, Cardiff, UK
2 School of Healthcare Sciences, Cardiff University, Eastgate House, Cardiff, UK
3 Department of Paediatrics, Morriston Hospital, Swansea, UK
4 Wirral University Teaching Hospital, Wirral, UK
5 Specialist Unit for Review Evidence (SURE), Cardiff University, Heath Park, Cardiff, UK
6 Public Health Wales, Cardiff, UK
7 Department of Paediatric Intensive Care, Children's Hospital for Wales, Cardiff, UK
8 Paediatric Emergency Medicine Leicester Academic (PEMLA) Group, Emergency Department, Infirmary Square, Leicester, UK
9 Paediatric Intensive Care Unit, Alder Hey Children's NHS Foundation Trust, Liverpool, UK
10 Department of Child Health, Children's Hospital for Wales, School of Medicine, Cardiff University, Cardiff, UK

Corresponding author:
Dr. Rob Trubey, Centre for Trials Research, Cardiff University, Heath Park, Cardiff, UK
trubeyrj@cf.ac.uk

Keywords: PEWS, track and trigger scores, early warning scores, clinical deterioration, children, systematic review

Word count: 3.999

For peer review only

ABSTRACT

Objectives

To assess (1) how well validated existing paediatric track and trigger tools (PTTT) are, and (2) how effective paediatric early warning systems (with or without a PTTT) are at reducing mortality and morbidity outcomes in hospitalised children.

Methods

A systematic search was carried out from inception through September 2016, across several databases. Supplementary searches were carried out to identify published and unpublished literature. Studies reporting on the development, validation and effectiveness of PTTT, or broader early warning systems with or without a PTTT were eligible for inclusion. Study selection, data extraction and quality assessment were conducted by two independent reviewers and disagreements resolved by discussion. PROSPERO registration number CRD42015015326.

Results

35 validation studies and 24 effectiveness studies were included. They were predominantly carried out in North America, Australia and the UK, and were largely single-site studies conducted in specialist centres. Outcome measures varied considerably. Validation studies were largely retrospective, case-control studies: PTTT either demonstrated high sensitivity or specificity, but not both. Positive predictive value was typically low, suggesting the potential for alarm fatigue. No studies accounted for the co-occurrence of routine clinical intervention and the longitudinal nature of the predictors in evaluating the link between high PTTT scores and subsequent deterioration. Some effectiveness studies showed significant reductions in mortality or morbidity outcomes following introduction of a PTTT, but we consider some methodological issues in interpreting their findings.

Conclusion

There are a number of fundamental methodological limitations in the PTTT literature, and a predominance of single-site studies carried out in specialist centres limits generalisability. With limited evidence of effectiveness, we would argue that calls to make their use mandatory across all paediatric units are not supported by the evidence base. We discuss

For peer review only

future research requirements, including the need to understand the impact of PTTT implementation on the wider clinical microsystem.

For peer review only

STRENGTHS AND LIMITATIONS OF THIS STUDY

- Paediatric track and trigger tools (PTTT) are increasingly used by paediatric units across Europe, North America and Australia – this study is a timely review of the evidence for their validity and effectiveness
- A comprehensive search was carried out across multiple databases and included published as well as grey literature
- The review highlights the weaknesses in the current evidence base and makes suggestions for future research
- Inconsistencies in study populations and outcome measures make comparisons across studies and PTTT difficult
- The generalisability of the PTTT findings is limited by the predominance of single-site studies conducted in specialist centres, and a reliance on retrospective and before-and-after studies

For peer review only

BACKGROUND

Failure to recognise and respond to clinical deterioration in hospitalised children is a major safety concern in healthcare. The underlying causes of this problem are clearly multi-factorial[1 –3] but 'early warning scores' or 'track and trigger tools' have been strongly advocated as one approach to improving recognition of deterioration in paediatric units[1,2,4].

These scores or tools, now commonly employed in adult care, provide a framework for evaluating a child's routine physiological and behavioural signs for early indicators of potential deterioration. If one or more observations fall outside of an age-specific threshold, bedside staff are required to escalate care accordingly [4]. Consequently, they can be considered a form of clinical prediction tool, "intended to guide clinicians in their everyday decision making"[5]. They have been referred to in the literature using a number of different terms: paediatric early warning scores or systems (PEWS); paediatric early warning tools (PEWT), track and trigger tools (TTT) and many others. Here, we refer to them using the umbrella term 'paediatric track and trigger tools' (PTTT).

Several different PTTT have been developed, typically by teams based in specialist paediatric centres and designed to be used alongside a rapid response team (RRT). However, their advocacy has recently led to widespread uptake across a variety of different paediatric units, including many non-specialist centres where patient populations and resources may differ. In the United Kingdom (UK), for instance, a recent cross-sectional survey found that 85% of paediatric units were using some form of PTTT, the vast majority of which were non-specialist centres without an RRT [6]. Clinical prediction rules should be subjected to a number of evaluation phases before being implemented in practice: derivation and development, narrow and broad validation and impact analysis [7]. It is unclear whether any of the PTTT in use across paediatric units have followed this process: two recent systematic reviews of published PTTT were critical of the evidence-base, focusing on the lack of evidence for their effectiveness for improving patient outcomes [8,9]. Although there is a growing literature base considering adult track and trigger tools, the generalisability of these findings to paediatrics is limited by differences in the nature of deterioration in adult and paediatric in-patients. The current review aimed to build on the existing paediatric work, assessing in depth the evidence base for both the validation and impact of PTTT through two research questions:

- Question 1: How well validated are existing paediatric track and trigger scores and their component parts?

For peer review only

- Question 2: How effective are paediatric early warning systems (with or without a PTTT) at reducing mortality and critical events?

For peer review only

METHODS

This systematic review was conducted and reported in accordance with the Preferred Reporting Items for Systematic Review and Meta-Analyses (PRISMA) guidelines[10]. Our review is registered with the PROSPERO database CRD42015015326.

Search strategy

A comprehensive search was conducted across a range of databases to identify relevant studies in the English language. Both published and unpublished literature was considered where publicly available, as were studies in press. The following databases were searched from inception through September 2016: British Nursing Index, CINAHL (Cumulative Index of Nursing and Allied Health Literature), Cochrane Central Register of Controlled Trials, Database of Abstracts of Reviews of Effectiveness, EMBASE, HMIC (Health Management Information Centre), Medline, Medline in Process, Scopus and Web of Knowledge (Science Citation Indexes). To identify additional papers, published, unpublished or research reported in the grey literature a range of relevant websites and trial registers were searched including Clinical Trials.gov. To identify published papers that had not yet been catalogued in the electronic databases, recent editions of key journals were hand-searched. The search terms included ‘early warning scores’, ‘alert criteria’, ‘rapid response’, ‘track and trigger’ and ‘early medical intervention’. Further details are supplied in supplementary table 1.

Eligibility criteria and study selection

Studies reporting the development and validation of PTTT and/or the effectiveness of broader early warning systems at reducing mortality and critical events in paediatric units up until September 2016 were eligible for inclusion. The review included studies with children aged 0-18 who were in-patients in a hospital.

Outcome measures were mortality and critical events, including: unplanned admission to a higher level of care, cardiac arrest, respiratory arrest, medical emergencies requiring immediate assistance, children reviewed by Paediatric Intensive Care Unit (PICU) staff on the ward (in specialist centres) or reviewed by external PICU staff (for non-specialist centres), acuity at PICU admission and the receipt of critical interventions at ward or PICU level.

For peer review only

Two of the review authors independently screened the titles and abstracts yielded in the search. Full texts were then reviewed independently by six reviewers against the eligibility criteria and were assigned to the relevant review question. Reasons for exclusion were recorded.

A data extraction form was developed for the two questions which had some common elements relating to both research questions (study design, country, setting, study population, exact nature of the PTTT or early warning system, statistical techniques used, outcomes assessed). Additional data items for validation studies included the items in the PTTT, modifications to the PTTT from previous versions, predictive ability of individual items and the overall tool, sensitivity and specificity and inter and intra-rater reliability. Effectiveness studies included an assessment of outcomes in terms of mortality and various morbidity variables. Data extraction was carried out by two reviewers and discrepancies were resolved by discussion. The methodological quality of the included studies was assessed using a modified version of the Downs and Black rating scale[11].

For peer review only

REVIEW RESULTS

Figure 1 shows the PRISMA flow diagram.

<Figure 1 here>

Study characteristics

Table 1 summarises the study characteristics of the 35 validation (Question 1) and 24 effectiveness (Question 2) papers included in the review, with full details in supplementary table 2 and 3.

Validation studies (n=35)			Effectiveness studies (n=24)		
	n	%		n	%
**Type**			**Type**		
Full text	22	62.9	Full text	16	66.7
Abstract	13	37.1	Abstract	8	33.3
**Country**			**Country**		
United States	21	60.0	United States	14	58.3
United Kingdom	12	34.3	United Kingdom	3	12.5
Canada	3	8.6	Canada	2	8.3
Australia	0	0.0	Australia	3	12.5
Other	6	17.1	Other	2	8.3
**Year of study**			**Year of study**		
Pre-2012	11	31.4	Pre-2012	14	58.3
2012	4	11.5	2012	1	4.2
2013	6	17.1	2013	2	8.3
2014	5	14.3	2014	6	25.0
2015	7	20.0	2015	0	0.0
2016	2	5.7	2016	1	4.2
**Setting**			**Setting**		
Specialist / tertiary	32	91.4	Specialist / tertiary	23	95.8
Non-specialist / community	0	0.0	Non-specialist / community	1	4.2
Unclear	3	8.6	Unclear	0	0.0
**Single / multi-centre**			**Single / multi-centre**		
Single-centre	34	97.1	Single-centre	23	95.8
Multi-centre	1	2.9	Multi-centre	1	4.2
**Study population**			**Study population**		
General in-patients	23	65.7	General in-patients	16	66.7
Specialist population	10	28.6	Specialist population	3	12.5
Unclear	2	5.7	Unclear	5	19.2
**Study design**			**Study design / analysis**		
Cohort	7	20.0	Before & after	24	92.4
Case-control	18	51.4	Interrupted Time Series	2	7.6
Case / chart review	9	25.7			
Pilot study	1	2.9			

For peer review only

Table 1: Summary study characteristics of validation and effectiveness papers in the review

Question 1 – How well validated are PTTT and component parts?

Types of PTTT and components

A range of different PTTT were identified in the validation studies, but the vast majority were based on four different PTTT: the Brighton PEWS[12–21], the Bedside PEWS (and its predecessor, the 'Paediatric Early Warning System score')[18,22–30], the Bristol PEWT[3,28,31–35] and the Melbourne Activation Criteria (including a modification, the Cardiff & Vale PEWS)[3,36,37]. Of the four most commonly cited tools, two are originally single-item triggering systems, where escalation of care is prompted by any one abnormal parameter (Bristol PEWS, Melbourne Activation Criteria) and two are aggregate-score systems, where the number of abnormal parameters are summed, typically with a graded escalation plan associated with different scoring thresholds (Bedside PEWS, Bristol PEWT). Modifications to existing PTTT were common, including the addition or removal of scoring items, adjustments to scoring or triggering thresholds (often tailored to specialist unit populations) and adjustments to the escalation procedure or 'algorithm' linked to the aggregate score. Other PTTT referenced included the National Health Service Institute for Innovation and Improvement (NHS III) PEWS[38], the most commonly used PTTT in United Kingdom paediatric settings[6], and a small number of prediction algorithms which draw on electronic health records[18,39].

While their underlying mechanisms differ, there was significant crossover between the various tools in terms of physiological and behavioural components (Table 2). Notably, each PTTT requires a minimum of seven recorded parameters to assess potential deterioration. The thresholds at which each of the vital signs or observations triggered or contributed to an aggregate score varied between PTTT. Most tools use age-adjusted thresholds for establishing abnormal vital signs, derived by either consensus-based or evidence-based means.

For peer review only

Required vital signs or observations for scoring	Bedside PEWS	Brighton PEWS	Bristol PEWT	Melbourne Activation Criteria
Respiratory rate	✓	✓	✓	✓
Heart rate	✓	✓	✓	✓
Respiratory effort / distress	✓	✓	✓	✓
Level of consciousness / behaviour		✓	✓	✓
Oxygen saturation	✓		✓	✓
Capillary refill time	✓	✓	✓	
Oxygen therapy	✓	✓		
Systolic blood pressure	✓		✓	✓
Other		✓	✓	
Pain			✓	
Staff concern			✓	✓
Skin colour		✓		
Airway obstruction / abnormality			✓	✓
Temperature				
Pulses				
Family concern				

Table 2: Common physiological or behavioural components underpinning published PTTT

Outcome measures

Most studies evaluated the validity of the PTTT in terms of diagnostic statistics such as the Area under the Receiver Operating Characteristic (AUROC), sensitivity, specificity and positive predictive value (PPV).

The definition of case patients (case control studies, chart reviews) or primary outcome measure (cohort studies, observational studies) varied greatly between studies, with composite measures common. Cardiac/respiratory arrest or a "code call" was used as an outcome measure (or part of a composite outcome) in several studies, but most studies used proxy measures of deterioration such as transfer to a higher level of care (e.g., PICU or PHDU), RRT/MET call or senior review. Of note, the majority (20/35) of validation papers had 'bench tested' the PTTT – that is, the PTTT was not prospectively tested. In these studies, the performance of the PTTT was retrospectively evaluated by extracting

For peer review only

documented observations from medical records and charts and using these to generate a score to assess whether a triggering threshold would have been met.

AUROC, Sensitivity, Specificity and PPV

With few exceptions[36], almost all papers reported on the performance characteristics of the PTTT as a whole rather than reporting the contribution of individual components. Seventeen papers reported an AUROC, with scores ranging from 0.73-0.92, and18 papers reported both the sensitivity and specificity of the PTTT and a further six studies reported sensitivity only.

Table 3 summarises the results of the PTTT validation studies, with full details in supplementary table 4. Three papers[3,18,28] reported outcomes for multiple PTTT in the same study, while one paper described three separate studies[23].

<Table 3 here>

Overall, PTTT demonstrated either high sensitivity or high specificity, but not both. Studies reporting performance characteristics of a PTTT at a range of different scoring thresholds[19,36] demonstrate the expected interaction between sensitivity and specificity – at a lower threshold, sensitivity is high but specificity is low; at higher thresholds, the opposite is true. While very few authors articulated the reason for preferring a certain threshold, the vast majority of studies where sufficient data was reported chose thresholds at which specificity was higher than sensitivity. Exceptions to this pattern were largely found in studies using versions of the Brighton PEWS, where cut-off points of 2, 2.5 and 3 resulted in higher sensitivity than specificity in some studies[16,19,21], and one instance of the Bristol PEWT[28]. Studies using similar PTTT and scoring thresholds produced notably different characteristics in different centres: Zhai and colleagues reported a specificity of 82% using a modified version of the Brighton PEWS with a triggering threshold of 2[18]; while Mandell and colleagues used the same version and of the tool with the same threshold, reporting a specificity of 58%[21].

PPV values reported in the four cohort studies were consistently low: 5.9% for the Cardiff & Vale PEWS[37], 3.6% for the Melbourne Activation Criteria[36], 5.8% for the Brighton PEWS[19] and 2.6% for the NHS III PEWS[38]. While a small number of case-control studies did report higher PPV percentages[16], they do not appear to have been adjusted to account for the underlying prevalence of adverse events among the inpatient population, and so are likely to be distorted.

Inter-rater reliability and missing data

For peer review only

Some PTTT contain items whose scoring requires some subjective judgement, most notably the Brighton PEWS. However, only three papers reported on details of inter-rater reliability of scoring[12,16,19], with mixed results: Tucker *et al.* reported that two nurses scored 55 patients on their modification of the Brighton PEWS[19], achieving an intra-class coefficient of 0.92, but McLellan *et al*., reported only 67% agreement in scoring on their C-CHEWS tool between a study nurse and the bedside nurse[16]. Missing or incomplete data was reported in just 11 papers. Fuijkschot *et al.*[23] reported that in 59% of cases reviewed "the PEWS was correctly performed and could be used for inclusion in the study". Edwards *et al.*[36] did not implement their PTTT, but modified observation charts to include all eight items in the Cardiff & Vale PEWS. They reported an average completion rate of44% for the different parameters. In the only multi-centre validation study identified, Parshuram *et al.*[26] reported that "of the 23,288 hours studied; only 5.1% had measurements on all 7 items, indicating that incomplete data were very common".

Question 2 – how effective are early warning systems?

Type of early warning system

The 'early warning system' interventions described in the 24 studies were typically multi-faceted. Overall, 22 of the studies included the introduction of a new PTTT, with one paper reporting an educational intervention only[40] and one paper reporting the introduction of a mandatory triggering system for a pre-existing PTTT and RRT[41]. A total of 14 of the 24 studies involved the introduction of a new medical emergency team (MET) or RRT alongside the PTTT, with a further six studies where an RRT or MET was already in place. Only two studies evaluated the effectiveness of a PTTT in the absence of a dedicated response team[42,43]. A range of PTTT were used in Question 2 studies, with several using unpublished, in-house activation criteria[44–50]. The most commonly used PTTT were versions of the Brighton PEWS[41,51–54], the Bedside PEWS[42,55,56] and Melbourne Activation Criteria[57–60].

Outcome measures

A variety of patient outcome measures were used to evaluate the effectiveness of early warning systems. Mortality, arrests and emergency "code calls" were the most common primary outcome measures, whereas in some cases the primary outcome measure was the

For peer review only

number of RRT/MET calls, with the stated aim of trying to increase utilisation of these dedicated teams[40,41,44,54].

Mortality and morbidity

Table 4 summarises the key findings from the 24 papers identified for Question 2, while supplementary table 5 gives full details of the studies included.

<Table 4 here>

Overall, three papers reported a significant decrease in mortality following implementation of an early warning system[48,58,60], each of which involved the use of a single-item trigger PTTT (Melbourne Activation Criteria or in-house, unpublished calling criteria) and an RRT or MET. One of these papers did not report any denominator data[48], so their reported reduction was in overall numbers rather than a rate, meaning the changes may have reflected underlying admission numbers. The other two papers reported reductions in all-hospital mortality per 100 discharges[58] or per 1,000 admissions[60]. The same three papers also reported significant reductions in either cardiac arrests[48] (although again based solely on overall numbers), code calls which lead to critical interventions[58] and ‘preventable’ cardiac arrest[60].

Three further papers reported significant reductions in arrests[46] (no denominator used), respiratory arrests only[47] (per 1,000 patient-days), and code calls but not actual arrests[57] (per 1,000 admissions). Again, each of these centres implemented a single-item trigger (Melbourne Activation Criteria or in-house, unpublished activation criteria) and an RRT or MET alongside the PTTT.

Four other studies reported significant findings. In one of the few studies conducted in a non-specialist centres, Parshuram *et al*. used the Bedside PEWS (without a dedicated response team) and reported a significant increase in the rate (per 1,000 patient-days) of children requiring transfer to an external paediatric intensive care unit (PICU), but with a significant decrease in the rate of children requiring a critical intervention at ward level prior to transfer[42]. Following the introduction of the Bedside PEWS alongside a MET, Bonafide *et al.* found a trend towards reductions in mortality and arrest rates, but neither was statistically significant[56]. They did, however, report a significant reduction in the number of children who required a ‘critical intervention’ within 12 hours of being admitted to PICU. Of note, this was the only study to use an Interrupted Time Series (ITS) design, in which the trend (or slope) in outcome measures is compared for a set period of time pre and post intervention. Sefton *et al.* implemented the Bristol PEWT (without a dedicated response team) and evaluated

characteristics of PICU admissions before and after the PTTT[43]. They found decreases in acuity of children at PICU admission, the percentage of children requiring invasive ventilation in PICU and the median length of stay on the intensive care unit. Finally, Douglas *et al*. implemented the Brighton PEWS alongside an existing RRT team, and reported a significant increase in the number of RRT calls per 1,000 patient-days but no associated reduction in mortality or morbidity[54].

For peer review only

DISCUSSION

This paper aimed to review the published PTTT and early warning system literature in order to assess the extent to which PTTT had been validated (Question 1) and the extent to which early warning systems had been shown to be effective at reducing mortality and morbidity (Question 2). We believe these specific questions have not been answered in previous reviews.

How well validated are existing tools?

Although a number of studies report findings that suggest that PTTT have some clinical predictive utility, we would argue that there a number of common methodological issues among validation studies that require the results to be interpreted with caution.

Importantly, each of the studies was conducted in a clinical setting where paediatric in-patients are subject to various forms of routine clinical intervention throughout their admission. This complicates the relationship between reaching a certain PTTT threshold at 'time A' and the occurrence of an adverse event at 'time B' – in a hypothetical 'perfect' hospital, children who displayed certain vital sign abnormalities would receive immediate clinical intervention and there would be no statistical relationship between a high PTTT score and adverse outcomes. Moreover, because events such as mortality or arrests are extremely rare, the majority of outcome measures used in the validation studies are clinical interventions themselves (e.g., PICU transfer). This is not to say that assessing the performance characteristics of a PTTT in a clinical setting is impossible – there are numerous statistical modelling techniques which can account for co-occurrence of clinical interventions and the longitudinal nature of the predictors[61,62]. However, none of the studies included in the review adequately account for this limitation and so authors' estimates of PTTT performance should be interpreted with some caution..

There are other methodological issues with the validation studies reviewed. Given that the majority of studies used a case-control design, PPV in particular will be influenced by which case and control patients reflect the broader in-patient population. The variety of different outcome measures and study populations, and the dominance of studies conducted in specialist centres also greatly limit the generalisability of the results from these validation studies. The tendency for centres to modify existing scores prevents broad validation of any one tool in a variety of settings and study populations.

For peer review only

Finally, the fact that most studies only 'bench-tested' the PTTT limits the understanding of the way in which a tool might interact with the wider clinical microsystem. As a result, a balanced understanding of the potential benefits and limitations for using a PTTT in clinical practice is currently lacking from the literature. For instance, studies reporting missing data/completeness rates and PPV values point to two potential barriers for implementing and sustaining a PTTT in practice. Each PTTT pre-supposes that several vital signs and observations are regularly completed in order to properly score patients. However, this appears to be uncommon, and evidence from the adult literature points to the potential for tools to inadvertently mask deterioration when core observations are missing[63]. Missing data can in itself be informative, however none of the papers studied in this review modelled for it. Some studies report PTTT triggering for almost 50% of children on their unit at some point during admission[36], and low PPV values point to the potential risk of 'alarm fatigue' due to the vast majority of triggering episodes occurring for children who are not deteriorating (although, as mentioned above, these figures may be distorted due to routine intervention preventing deterioration in clinical practice). For some centres, these issues may be mitigated to some extent by dedicated response teams or other available resources, but other hospitals may not be able to sustain the increased workload of responding to PTTT triggers.

There has, to date, been a particular focus on evaluating and comparing the statistical properties of PTTT, with less discussion of wider contextual factors and the complexity of embedding a PTTT in clinical practice. Given the methodological limitations discussed above, we would suggest that such an approach puts too much weight on poorly estimated statistical characteristics, and not enough on the various human and organisational impacts of using different PTTT in everyday practice.

How effective are existing tools?

Several effectiveness papers reported significant reductions in mortality or morbidity outcomes following implementation of an early warning system inclusive of a PTTT. However, interpretation of these results requires caution, given that the studies almost exclusively employed a 'before and after' study design, where rates of mortality and morbidity are compared between one time period pre-implementation and a second time period post-implementation. Before and after designs have inherent limitations in terms of establishing causality, in that they do not exclude the possibility that mortality or morbidity rates would have decreased over time irrespective of the introduction of a PTTT[65]. Bonafide

For peer review only

and colleagues did notably evaluate the impact of their Bedside PEWS using a more sophisticated 'interrupted time series' approach, demonstrating a significant reduction in the proportion of children requiring critical intervention within 12 hours of being admitted to PICU after introduction of the PTTT[56]. This occurred alongside a parallel increase in overall PICU admissions, and so it is not clear whether the PTTT specifically improved identification of at-risk children, or rather encouraged a broadly more conservative approach to admitting children to the ICU.

The majority of studies reporting significant findings also implemented a dedicated response team (or already had one in place) in addition to staff training or education alongside the PTTT. It is therefore difficult to isolate the effect of a PTTT from other organisational or systemic factors that typically accompany its implication. While dedicated response teams are more common in the United States and Australia, most hospitals in other countries (including the UK) typically have response teams whose members have other clinical roles. Many of the calling criteria or PTTT reported in the effectiveness studies may therefore be unsuitable for wider adoption. A handful of papers reported results pointing to a broader pattern of increased PICU transfers but reductions in acuity or the necessity for critical interventions among those that were transferred[34,56]. In the absence of more information about how this trend impacts on workload and resources, it is again possible that some centres would be unable to sustain the level of transfers to a higher level of care that would result from implementing a PTTT and an associated escalation policy. As with validation studies, the generalisability of the results from the effectiveness studies is further limited by the heterogeneity of outcome measures used, and the specialist nature of almost all units reporting outcomes.

In the effectiveness studies reviewed here, there was very little data presented on the wider impact of PTTT[7]: including fidelity or adherence, or staff and resource implications of using a tool. Evidence from the adult care literature points to instances of poor compliance among clinical staff when early warning tools are implemented in practice[66,67], and highlights various sociocultural barriers to their adoption[68]. Future studies should give similar consideration to the wider impact of using a PTTT in paediatric units. The limited evidence of PTTT effectiveness identified in this review is consistent with similar conclusions drawn by recent systematic reviews of the area [8,9].

Conclusion

For peer review only

The PTTT literature is currently characterised by an 'absence of evidence' rather than an 'evidence of absence'. That is, PTTT may well have some clinical predictive utility for aiding with the detection and timely response to deteriorating patients, but the existing evidence base is too limited to form any clear judgements. Validation studies have focused heavily on the statistical properties of PTTT, with arguably too much confidence expressed in these findings given methodological limitations. In contrast, there is little understanding of how PTTT impact on, and interact with, the wider clinical microsystem in paediatric units and how they are received and used by the clinical staff whose decisions they are intended to aid. Almost all of the PTTT reported in the literature have been developed and tested in specialist centres, typically in units with access to dedicated response teams – this considerably limits the generalisability of the findings, and is noteworthy given that PTTT appear to be commonly adopted by non-specialist units with little modification. Combined with the current lack of evidence for PTTT improving patient outcomes, we would urge caution among policymakers in calling for their use to become mandatory across all units based on the current evidence base. More work is required to understand the wider impact of PTTT implementation in different clinical settings before it is possible to evaluate their overall contribution to the wider safety mechanisms and systems aimed at identifying and responding to deteriorating in paediatric patients.

For peer review only

FOOTNOTES

Contributors

RT: screening and review of papers, contribution to design of work, preparation of manuscript; CH: screening and review of papers, contribution to concept and design of work, review of manuscript; FL: contribution to design of work, screening and review of papers, review of manuscript; KH: contribution to concept and design of work, screening and review of papers, review of manuscript; CP, DR, BM, AO, DE, RS, GS, DL, LT, DA, AL, ETJ: contribution to concept and design of work, screening and review of papers, review of manuscript; MM: information specialist, review of manuscript.

Acknowledgements

We would like to acknowledge the contribution of Dr James Bunn to the review.

Competing interests

None declared.

Funding

This study is funded by the National Institute for Health Research (NIHR) Health Services and Delivery Research (HS&DR) programme (12/178/17).

Data sharing statement

No additional data are available.

For peer review only

FIGURE LEGENDS

Figure 1 – PRISMA flow diagram of study inclusion

For peer review only

REFERENCES

1. Pearson, G. a (Ed). *Why Children Die: A Pilot Study 2006; England (South West, North East and West Midlands), Wales and Northern Ireland.* (2008). doi:ISBN: 978-0-9558055-0-9

2. Joint Commission. 2009 National Patient Safety Goals, Requirements and Rationales. *Jt. Comm. Perspect. Patient Saf.* **8,** 8–12 (2009).

3. Tume, L. The deterioration of children in ward areas in a specialist children's hospital. *Nurs. Crit. Care* **12,** 12–19 (2007).

4. NICE. Acutely ill patients in hospital: Recognition of and response to acute illness in adults in hospital. *NICE Clin. Guidel. 50* 1–107 (2007). doi:10.1136/bmj.39272.679688.47

5. Adams, S. T. & Leveson, S. H. Clinical prediction rules. *BMJ* **344,** d8312 (2012).

6. Roland, D., Oliver, A., Edwards, E. D., Mason, B. W. & Powell, C. V. E. Use of paediatric early warning systems in Great Britain: has there been a change of practice in the last 7 years? *Arch. Dis. Child.* **99,** 26–9 (2014).

7. Toll, D. B., Janssen, K. J. M., Vergouwe, Y. & Moons, K. G. M. Validation, updating and impact of clinical prediction rules: A review. *Journal of Clinical Epidemiology* **61,** 1085–1094 (2008).

8. Chapman, S. M., Wray, J., Oulton, K. & Peters, M. J. Systematic review of paediatric track and trigger systems for hospitalised children. *Resuscitation* **109,** 87–109 (2016).

9. Lambert, V., Matthews, A., MacDonell, R. & Fitzsimons, J. Paediatric early warning systems for detecting and responding to clinical deterioration in children: a systematic review. *BMJ Open* **7,** e014497 (2017).

10. Moher, D., Liberati, A., Tetzlaff, J. & Altman, D. G. Preferred reporting items for systematic reviews and meta-analyses: the PRISMA statement. *BMJ* **339,** (2009).

11. Downs, S. H. & Black, N. The feasibility of creating a checklist for the assessment of the methodological quality both of randomised and non-randomised studies of health care interventions. *J. Epidemiol. Community Health* **52,** 377–384 (1998).

12. Bell, D. *et al.* The texas children's hospital pediatric advanced warning score as a predictor of clinical deterioration in hospitalized infants and children: A modification of the pews tool. *J. Pediatr. Nurs.* **28,** (2013).

13. Garlick, J. *et al.* Modified pediatric early warning scores, a predictor of appropriate transfer to higher level of care. *Crit. Care Med.* **41,** A166–A167 (2013).

14. Akre, M. *et al.* Sensitivity of the pediatric early warning score to identify patient deterioration. *Pediatrics* **125,** e763–9 (2010).

For peer review only

15. McLellan, M. C. & Connor, J. A. The Cardiac Children's Hospital Early Warning Score (C-CHEWS). *J. Pediatr. Nurs.* **28,** 171–178 (2013).

16. Mclellan, M. C., Gauvreau, K. & Connor, J. A. Validation of the cardiac children's hospital early warning score: An early warning scoring tool to prevent cardiopulmonary arrests in children with heart disease. *Congenit. Heart Dis.* **9,** 194–202 (2014).

17. Skaletzky, S. M., Raszynski, A. & Totapally, B. R. Validation of a modified pediatric early warning system score: a retrospective case-control study. *Clin. Pediatr. (Phila).* **51,** 431–5 (2012).

18. Zhai, H. *et al.* Developing and evaluating a machine learning based algorithm to predict the need of pediatric intensive care unit transfer for newly hospitalized children. *Resuscitation* **85,** 1065–1071 (2014).

19. Tucker, K. M., Brewer, T. L., Baker, R. B., Demeritt, B. & Vossmeyer, M. T. Prospective evaluation of a pediatric inpatient early warning scoring system. *J. Spec. Pediatr. Nurs.* **14,** 79–85 (2009).

20. Fenix, J. B., Gillespie, C. W., Levin, A. & Dean, N. Comparison of Pediatric Early Warning Score to Physician Opinion for Deteriorating Patients. *Hosp. Pediatr.* **5,** 474–479 (2015).

21. Mandell, I. M. *et al.* Pediatric Early Warning Score and unplanned readmission to the pediatric intensive care unit. *J. Crit. Care* **30,** 1090–1095 (2015).

22. Duncan, H., Hutchison, J. & Parshuram, C. S. The pediatric early warning system score: A severity of illness score to predict urgent medical need in hospitalized children. *J. Crit. Care* **21,** 271–278 (2006).

23. Fuijkschot, J., Vernhout, B., Lemson, J., Draaisma, J. M. T. & Loeffen, J. L. C. M. Validation of a paediatric early warning score: First results and implications of usage. *Eur. J. Pediatr.* **174,** 15–21 (2015).

24. Hopkins, M., Rowan, C., Rigby, M. & Tori, A. PEWS score predicts the need for PICU transfer and critical care interventions. *Crit. Care Med.* **Conference,** 43rd Critical Care Congress of the Society of Crit (2013).

25. Gawronski, O. *et al.* Bedside pews in an Italian pediatric bone marrow transplant unit: Preliminary results of a longitudinal retrospective chart review. *Intensive Care Med.* **39,** S130 (2013).

26. Parshuram, C. S. *et al.* Multicentre validation of the bedside paediatric early warning system score: a severity of illness score to detect evolving critical illness in hospitalised children. *Crit Care* **15,** R184 (2011).

27. Parshuram, C. S., Hutchison, J. & Middaugh, K. Development and initial validation of the Bedside Paediatric Early Warning System score. *Crit. Care* **13,** R135–R135 (2009).

For peer review only

28. Robson, M.-A. J., Cooper, C. L., Medicus, L. a, Quintero, M. J. & Zuniga, S. a. Comparison of three acute care pediatric early warning scoring tools. *J. Pediatr. Nurs.* **28,** e33–41 (2013).

29. Ross, C. *et al.* Sensitivity and specificity of a pediatric early warning system using data-driven vital signs. *Crit. Care Med.* **43,** 39 (2015).

30. Gawronski, O. *et al.* Accuracy of Bedside Paediatric Early Warning System (BedsidePEWS) in a Pediatric Stem Cell Transplant Unit. *J. Pediatr. Oncol. Nurs.* **33,** 249–256 (2016).

31. Clayson, R., Selby, A. & Tume, L. Is a paediatric early warning tool (PEWT) useful in cardiac patients? *Pediatr. Crit. Care Med.* **15,** 35–36 (2014).

32. Wright, D., Sefton, G. & Horan, M. Charted observations and the use of a paediatric early warning tool did not predict the majority of cardio-respiratory arrest calls in a paediatric hospital. *Arch. Dis. Child.* **96,** A59 (2011).

33. Haines, C., Perrott, M. & Weir, P. Promoting care for acutely ill children - Development and evaluation of a Paediatric Early Warning Tool. *Intensive Crit. Care Nurs.* **22,** 73–81 (2006).

34. Sefton, G., Lane, S. & Carrol, E. D. Performance of individual predictors of deterioration used in a paediatric early warning score. *Pediatr. Crit. Care Med.* **15,** 29–30 (2014).

35. O'Loughlin, K. *et al.* The effectiveness of the paediatric early warning tool (PEWT) in identifying children requiring admission to a critical care unit. *Arch. Dis. Child.* **97,** A154 (2012).

36. Edwards, E. D., Mason, B. W., Oliver, A. & Powell, C. V. E. Cohort study to test the predictability of the Melbourne criteria for activation of the medical emergency team. *Arch. Dis. Child.* **96,** 174–179 (2011).

37. Edwards, E. D., Powell, C. V, Mason, B. W. & Oliver, A. Prospective cohort study to test the predictability of the Cardiff and Vale paediatric early warning system. *Arch Dis Child* **94,** 602–606 (2009).

38. Mason, B. W., Edwards, E. D., Oliver, A. & Powell, C. V. E. Cohort study to test the predictability of the NHS Institute for Innovation and Improvement Paediatric Early Warning System. *Arch. Dis. Child.* archdischild–2015–308465 (2016). doi:10.1136/archdischild-2015-308465

39. Lin, J. *et al.* A simplified pediatric early warning system that relies solely on objective measures. *Crit. Care Med.* **43,** 192 (2015).

40. Ambati, S. R., Sweberg, T., Silver, P. & Gangadharan, S. Effect of simulation based curriculum on the utilization of rapid response team activations (RRT). *Crit. Care Med.* **42,** A1432 (2014).

For peer review only

41. Panesar, R. *et al.* Characteristics and outcomes of pediatric rapid response teams before and after mandatory triggering by an elevated Pediatric Early Warning System (PEWS) score. *Hosp. Pediatr.* **4,** 135–140 (2014).

42. Parshuram, C. S., Bayliss, A., Reimer, J., Middaugh, K. & Blanchard, N. Implementing the Bedside Paediatric Early Warning System in a community hospital: A prospective observational study. *Paediatr. Child Health* **16,** e18–22 (2011).

43. Sefton, G. *et al.* What impact did a Paediatric Early Warning system have on emergency admissions to the paediatric intensive care unit? An observational cohort study. *Intensive Crit. care Nurs.* **31,** 91–99 (2015).

44. Duns, N. *et al.* Introduction of a track and trigger system is associated with increased paediatric intensive care outreach utilisation and a trend towards improved patient outcomes in a tertiary children's hospital. *Aust. Crit. Care* **27,** 61 (2014).

45. Hanson, C. C. *et al.* A reduction in cardiac arrests and duration of clinical instability after implementation of a paediatric rapid response system. *Postgrad. Med. J.* **86,** 314–318 (2010).

46. Heyden, M. V, Mudge, B. & Casella, S. Initiation of a dedicated pediatric rapid response team reduces cardiopulmonary arrests outside of the intensive care unit in a small children's hospital. *Crit. Care Med.* **40,** 147 (2012).

47. Hunt, E. A. *et al.* Transition from a traditional code team to a medical emergency team and categorization of cardiopulmonary arrests in a children's center. *Arch. Pediatr. Adolesc. Med.* **162,** 117–122 (2008).

48. Mistry, K. P., Turi, J., Hueckel, R., Mericle, J. M. & Meliones, J. N. Pediatric Rapid Response Teams in the Academic Medical Center. *Clin. Pediatr. Emerg. Med.* **7,** 241–247 (2006).

49. Anwar-Ul-Haque, Saleem, A. F., Zaidi, S. & Haider, S. R. Experience of pediatric rapid response team in a tertiary care hospital in Pakistan. *Indian J. Pediatr.* **77,** 273–276 (2010).

50. Zenker, P. *et al.* Implementation and impact of a rapid response team in a children's hospital. *Jt. Comm. J. Qual. Patient Saf.* **33,** 418–425 (2007).

51. Camacho, E. A., Holman, D., Badgett, V., St. Peters, J. & Southworth, B. Modification of a Pediatric Early Warning Tool for Use With Patients in a Specialized Cardiac/Renal Unit. *J. Pediatr. Nurs.* **26,** e2–e3 (2011).

52. Demmel, K. M., Williams, L. & Flesch, L. Implementation of the pediatric early warning scoring system on a pediatric hematology/oncology unit. *J. Pediatr. Oncol. Nurs.* **27,** 229–240 (2010).

53. Randhawa, S., Roberts-Turner, R., Woronick, K. & DuVal, J. Implementing and Sustaining Evidence-Based Nursing Practice to Reduce Pediatric Cardiopulmonary Arrest. *West. J. Nurs. Res.* **33,** 443–456 (2010).

For peer review only

54. Douglas, K., Collado, J. C. & Keller, S. Implementation of a Pediatric Early Warning Scoring System at an Academic Medical Center. *Crit. Care Nurs. Q.* **39,** 363–370 (2016).

55. Ocholi, T., Downing, E., Rzeskiewicz, A., Pang, R. & Broughton, S. Impact of the Bedside PEWS on early detection of deteriorating patients. *Arch. Dis. Child.* **99,** A89–A90 (2014).

56. Bonafide, C. P. *et al.* Impact of Rapid Response System Implementation on Critical Deterioration Events in Children. *JAMA Pediatr.* **168,** 25 (2014).

57. Kotsakis, A. *et al.* Implementation of a multicenter rapid response system in pediatric academic hospitals is effective. *Pediatrics* **128,** 72–78 (2011).

58. Sharek, P. J. *et al.* Effect of a rapid response team on hospital-wide mortality and code rates outside the ICU in a Children's Hospital. *JAMA* **298,** 2267–2274 (2007).

59. Tibballs, J., Kinney, S., Duke, T., Oakley, E. & Hennessy, M. Reduction of paediatric in-patient cardiac arrest and death with a medical emergency team: preliminary results. *Arch. Dis. Child.* **90,** 1148–1152 (2005).

60. Tibballs, J. & Kinney, S. Reduction of hospital mortality and of preventable cardiac arrest and death on introduction of a pediatric medical emergency team. *Pediatr. Crit. Care Med.* **10,** 306–312 (2009).

61. Guerra, M. W., Shults, J., Amsterdam, J. & Ten-Have, T. The analysis of binary longitudinal data with time-dependent covariates. *Stat. Med.* **31,** 931–948 (2012).

62. Selig, J. P., Preacher, K. J. & Little, T. D. Modeling Time-Dependent Association in Longitudinal Data: A Lag as Moderator Approach. *Multivariate Behav. Res.* **47,** 697–716 (2012).

63. Clifton, D. A. *et al.* 'Errors' and omissions in paper-based early warning scores: the association with changes in vital signs-a database analysis. *BMJ Open* **5,** e007376 (2015).

64. Chapman, S. M. *et al.* 'The Score Matters': wide variations in predictive performance of 18 paediatric track and trigger systems. *Arch. Dis. Child.* archdischild–2016–311088 (2017). doi:10.1136/archdischild-2016-311088

65. Joffe, A. R., Anton, N. R. & Burkholder, S. C. Reduction in hospital mortality over time in a hospital without a pediatric medical emergency team: limitations of before-and-after study designs. *Arch. Pediatr. Adolesc. Med.* **165,** 419–423 (2011).

66. Niegsch, M., Fabritius, M. L. & Anh??j, J. Imperfect Implementation of an Early Warning Scoring System in a Danish Teaching Hospital: A Cross-Sectional Study. *PLoS One* **8,** (2013).

67. Hands, C. *et al.* Patterns in the recording of vital signs and early warning scores: Compliance with a clinical escalation protocol. *BMJ Qual. Saf.* **22,** 719–726 (2013).

For peer review only

68. Lydon, S., Byrne, D., Offiah, G. & Gleeson, L. A mixed-methods investigation of health professionals' perceptions of a physiological track and trigger system. *BMJ Qual.* 688–695 (2015). doi:10.1136/bmjqs-2015-004261

69. Egdell, P., Finlay, L. & Pedley, D. K. The PAWS score: validation of an early warning scoring system for the initial assessment of children in the emergency department. *Emerg Med J* **25,** 745–749 (2008).

70. Rahman, Z. H. *et al.* Application of the burn center Pediatric Early Warning Score (PEWS) tool to clinical practice: A pilot validation study. *J. Burn Care Res.* **35,** S187 (2014).

71. Ahmed, M. *et al.* Burton paediatric early warning system score. *Arch. Dis. Child.* **97,** A421 (2012).

72. Bonafide, C. P. *et al.* Development of a score to predict clinical deterioration in hospitalized children. *J. Hosp. Med.* **7,** 345–349 (2012).

73. Dean, N., Spaeder, M., Fenix, J. B. & Levin, A. Evaluation of a pediatric early warning score across different subspecialty patients. *Crit. Care Med.* **43,** 193 (2015).

74. Medar, S., Weingarten-Arams, J. & Katyal, C. Pediatric early warning score (PEWS) is a reliable predictor of rapid response team (RRT) call. *Crit. Care Med.* **43,** 228 (2015).

75. Sandhu, S. *et al.* 726 The Paediatric Early Warning Tool in Combination with a Nurse-Led Outreach Team: Impact on Outcomes for Critically Ill Children. *Pediatr Res* **68,** 368 (2010).

76. Norville, R., Wills-Bagnato, P., Staton, S. & Kennedy-Nasser, A. Improvement in early recognition of deteriorating pediatric bone marrow transplant patients. *Biol. Blood Marrow Transplant.* **19,** S173 (2013).

For peer review only

For peer review only

Table 3 – Summary of PTTT validation study findings

Reference	Tool name	Used in practice?	Setting	Study population	Study design	Outcome measure	Threshold tested	AUROC	Sensitivity	Specificity	PPV
Paediatric Early Warning System score / Bedside PEWS (and variants of)											
Duncan, 2006[22]	Paediatric Early Warning System score	No	Specialist / tertiary	General in-patients	Case-control	Arrest / code call	5	0.90	78.0	95.0	4.2*
Robson, 2013[28]	Paediatric Early Warning System score	No	Specialist / tertiary	General in-patients	Case-control	Arrest / code call	5	0.85	86.6	72.2	
Parshuram, 2009[27]	Bedside PEWS	No	Specialist / tertiary	General in-patients	Case-control	PICU / PHDU transfer	8	0.91	82.0	93.0	
Parshuram, 2011[26]	Bedside PEWS	No	Specialist / tertiary	General in-patients	Case-control	Arrest / code call; PICU / PHDU transfer	7	0.87	64.0	91.0	
Robson, 2013[28]	Bedside PEWS	No	Specialist / tertiary	General in-patients	Case-control	Arrest / code call	7	0.73	56.3	78.1	
Zhai, 2014[18]	Bedside PEWS	No	Specialist / tertiary	General in-patients	Case-control	PICU / PHDU transfer	7	0.82	73.6	71.7	2.1*
Brighton PEWS (and variants of)											
Tucker, 2009[19]	Modified PEWS	Yes	Specialist / tertiary	General in-patients	Cohort	PICU / PHDU transfer	3	0.89	90.2	74.4	5.8
Skaletzky, 2012[17]	Modified PEWS	No	Specialist / tertiary	General in-patients	Case-control	Arrest / code call; PICU / PHDU transfer; RRT / MET call	2.5	0.81	62.0	89.0	
McLellan, 2014[16]	Cardiac CHEWS	No	Specialist / tertiary	Specialist ward (cardiac)	Case-control	Arrest / code call; PICU / PHDU transfer	3	0.86	95.3	76.2	50.8*
Mandell, 2015[21]	Modified PEWS	Yes	Specialist / tertiary	PICU discharges to ward	Case-control	Re-admission to PICU	2	0.77	71.0	58.0	
Bristol PEWT (and variants of)											
Robson, 2013[28]	Paediatric Early Warning Tool	No	Specialist / tertiary	General in-patients	Case-control	Arrest / code call	*Single item trigger*	0.75	76.3	61.5	
O'Loughlin, 2012[35]	Paediatric Early Warning Tool	Yes	Specialist / tertiary	General in-patients	Cohort	PICU / PHDU transfer	*Single item trigger*	0.91	100.0	81.0	11.2*
Melbourne Activation Criteria (and variants of)											
Edwards, 2011[36]	Melbourne Activation Criteria	No	Specialist / tertiary	General in-patients	Cohort	Mortality; arrest / code call; PICU / PHDU transfer	*Single item trigger*	0.79	68.3	83.2	3.6
Edwards, 2009[37]	Cardiff & Vale PEWS	No	Specialist / tertiary	General in-patients	Cohort	Mortality; arrest / code call; PICU / PHDU transfer	2	0.86	69.5	89.9	5.9
Other											
Mason, 2016[38]	NHS III score	No	Specialist / tertiary	General in-patients	Cohort	Mortality; arrest / code call; PICU / PHDU transfer	2	0.83	73.2	75.2	2.6

*Estimated PPV based on case/control ratio or imputed prevalence rate
Note: A number of papers included in the review did not include sufficient statistical detail (e.g., AUROC, sensitivity and specificity) to include in this summary table [3,12–15,20,23–25,29–34,39,69–74]

Table 4 – Summary of effectiveness study findings

Reference	PTTT name	RRT / MET in place?	Setting	Study population	Study design	Outcomes						
						Mortality	Arrests / code calls	PICU / PHDU transfers	Acuity on PICU admission	Critical interventions	RRT/MET calls	Denominator
Paediatric Early Warning System score / Bedside PEWS (and variants of)												
Parshuram, 2011	Bedside PEWS	No	Non-specialist / community	General in-patients	Before & after			↑* (transfers to external specialist centre)		↓** (critical intervention prior to transfer)		Per 1,000 patient-days
Bonafide, 2014	Bedside PEWS	Yes	Specialist / tertiary	General in-patients	Interrupted Time Series		↓	↑		↓** (critical intervention within 12hrs of PICU admission)		Per 1,000 patient-days
Brighton PEWS (and variants of)												
Douglas, 2016	Modified PEWS	Yes	Specialist / tertiary	General in-patients	Before & after		↓				↑** (RRT calls)	Per 1,000 patient days
Bristol PEWT (and variants of)												
Sefton, 2014	Modified PEWT	No	Specialist / tertiary	General in-patients	Before & after				↓** (PIM2 median)	↓** (critical intervention during PICU admission)		Percentage of all PICU admissions
Melbourne Activation Criteria (and variants of)												
Sharek, 2007	Melbourne Activation Criteria	Yes	Specialist / tertiary	General in-patients	Before & after	↓** (hospital-wide mortality rate)	↓** (code call requiring critical intervention)					Per 1,000 patient-days (codes); Per 100 discharges (deaths)
Tibbals, 2009	Melbourne Activation Criteria	Yes	Specialist / tertiary	General in-patients	Before & after							
Kotsaki, 2011	Melbourne Activation Criteria	Yes	Specialist / tertiary	General in-patients	Before & after							
Other												
Mistry, 2006	Unpublished activation criteria	Yes	Specialist / tertiary	General in-patients	Before & after	↓** (non PICU inpatient	↓** (cardiac arrests)					None used

For peer review only

						deaths)						
Hunt, 2008	Unpublished activation criteria	Yes	Specialist / tertiary	General in-patients	Before & after		↓* (respiratory arrests only)					Per 1,000 patient-days
Heyden, 2012	Unpublished activation criteria	Yes	Specialist / tertiary	General in-patients	Before & after		↓** (arrests)				↑	None used

*p<0.05, ** p<0.01
Note: A number of papers included in the review did not report significant reductions in mortality or morbidity findings and so are not included in this summary table [40,41,44,45,49–53,55,59,75,76]

For peer review only

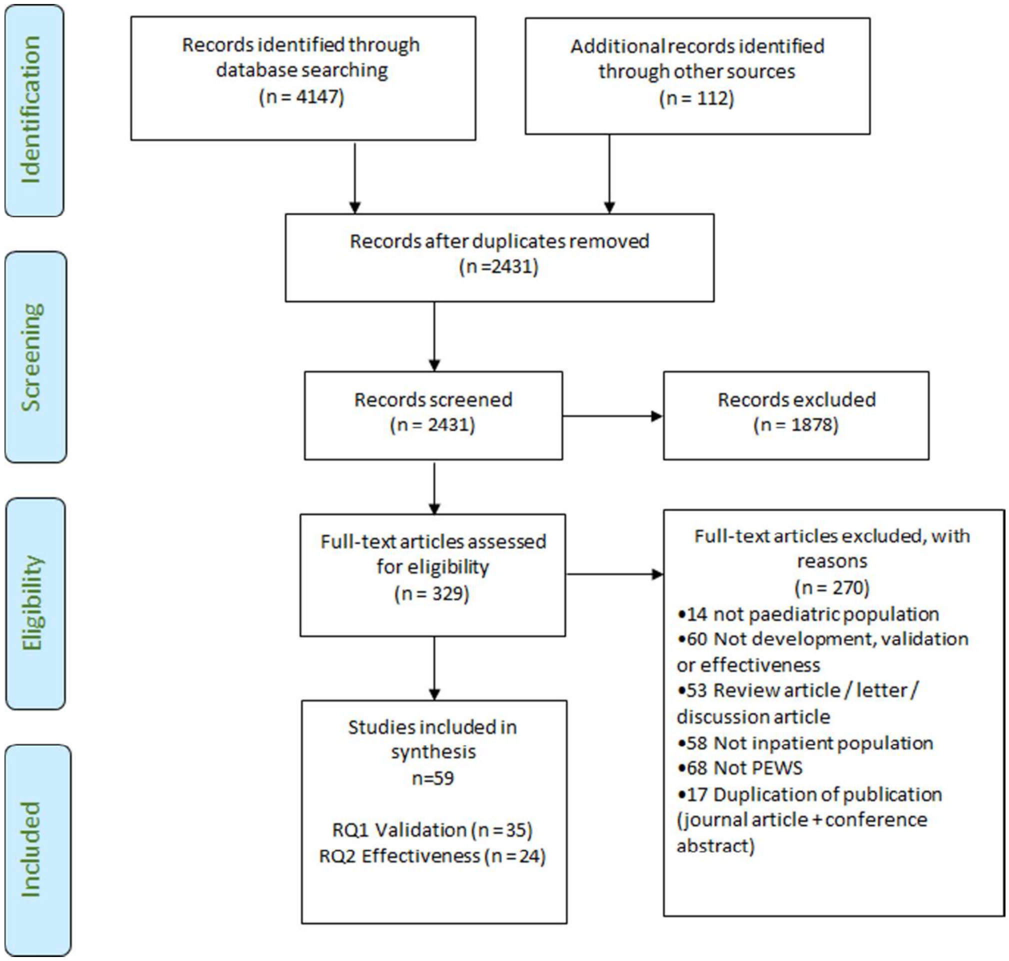

Figure 1: PRISMA flow diagram of study inclusion

99x95mm (300 x 300 DPI)

PUMA - Paediatric early warning score Utilisation and Mortality Avoidance Search Methods

Database Search

The search was across a range of databases from their inception to January 2015 then an update was carried out in September 2016

A preliminary search strategy was developed using a set of key papers known to the group for Ovid Medline using both text words and Medical subject headings. The search strategy was modified according to the indexing systems of the other databases.

Databases and Database platform	Original search results January 2015	Update September 2016
British Nursing Index (Proquest)	19	12
CINAHL (Cumulative Index of Nursing and Allied Health Literature) (Ebsco)	206	17
Cochrane Central Register of Controlled Trials (Wiley)	43	4
Database of Abstracts of Reviews of Effectiveness (Centre for Reviews and Dissemination)	0	0
EMBASE (OVID)	1065	206
HMIC (Health Management Information Centre) (OVID)	70	1
Medline (OVID)	943	135
Medline in Process (OVID)	43	69
Scopus (Elsevier)	747	85
Web of Knowledge (Science Science Citation Indexes) (Thomson Reuter)	400	82
**Total**	**3536**	**611**

Total 4147

Supplementary search

Trials Registers	Original search results January 2015	Update September 2016
ClinicalTrials.gov https://clinicaltrials.gov/	6	4
UK Clinical Trials Gateway http://www.ukctg.nihr.ac.uk/default.aspx	3 (duplicates)	5 (1 duplicate)
The WHO trial search portal for studies worldwide: http://apps.who.int/trialsearch	1 (duplicate)	0
Journal site	Hits	

For peer review only

Archives of Disease in Childhood<br>http://adc.bmj.com/	14	4
BMJ<br>http://www.bmj.com/theBMJ	1	0
BMJ Quality and safety<br>http://qualitysafety.bmj.com/	7	4
JAMA Pediatrics<br>http://archpedi.jamanetwork.com/journal.aspx	1	0
Journal of Critical Care<br>http://www.jccjournal.org/	3	1
Journal of Pediatrics (American)<br>http://www.jpeds.com/	1	0
Journal of Paediatrics and Child Health (Australian)<br>http://onlinelibrary.wiley.com/journal/10.1111/(ISSN)1440-1754	2	2
Lancet<br>http://www.thelancet.com/	0	0
New England Journal of Medicine<br>http://www.nejm.org/	0	0
Pediatrics<br>http://pediatrics.aappublications.org/	6	2
Pediatric Critical Care Medicine<br>http://journals.lww.com/pccmjournal/pages/default.aspx	14	6
Websites and organisations	HITS	
American Society of Anesthesiologists<br>https://www.asahq.org/	1	0
American Academy of Pediatrics<br>http://www.aap.org/en-us/Pages/Default.aspx	1	
Association of Anaesthetists of Great Britain and Ireland<br>http://www.aagbi.org/	0	0
Australian Medical Council<br>http://www.amc.org.au/	1	0
Royal College of Paediatrics and Child Health<br>http://www.rcpch.ac.uk/	1	0
Paediatric Nursing Association Europe<br>http://www.rcn.org.uk/	9	
European Federation of Critical Care Nursing Associations<br>http://www.efccna.org/	No Search Option	No Search Option
Royal Australasian College of Physicians (Division of Child Health)<br>https://www.racp.edu.au/page/paed-policy	0	0
Royal College of Physicians (inclusive of National Clinical Guideline Centre)<br>https://www.rcplondon.ac.uk/	2	0
The NHS Institute for Innovation and Improvement<br>http://www.institute.nhs.uk/	4	Site cease to exist
NICE: Eyes on Evidence<br>https://www.evidence.nhs.uk/about-evidence-services/bulletins-and-alerts/eyes-on-evidence	4	1
**TOTAL**	82	30

For peer review only

Total = 112

Search Strategies

BNI
"Paediatric Early Warning" OR ("pediatric early warning" OR "pediatric rapid response") OR ("paediatric rapid response" OR "Bedside paediatric early warning") OR ("Pediatric Advanced Warning Score" OR "Paediatric Advanced Warning Score")

CENTRAL
Search Name: PUMA search 15 jan 2015
Last Saved: 15/01/2015 16:12:08.536
Description:

ID	Search
#1	"early warning score*"
#2	"early warning system*"
#3	"early warning tool*"
#4	"VitalPAC Early Warning Score"
#5	"activation criteria"
#6	"Rapid Response Team"
#7	"Track and trigger"
#8	"trigger tools"
#9	"calling criteria"
#10	"Alert criteria"
#11	"Rapid Response"
#12	#1 or #2 or #3 or #4 or #5 or #6 or #7 or #8 or #9 or #10 or #11
#13	pediatric* or paediatric* or infant* or child* or baby or toddler or babies or teen* or adolescent*
#14	#12 and #13
#15	"Pediatric Early Warning"
#16	"Paediatric Early Warning"
#17	"paediatric alert"
#18	"pediatric alert"
#19	"Pediatric Rapid Response"
#20	"Paediatric Rapid Response"
#21	"Pediatric Advanced Warning Score*"
#22	"Paediatric Advanced Warning Score*"
#23	"infant early warning"
#24	"Bedside PEWS"
#25	"Bedside paediatric early warning"
#26	#15 or #16 or #17 or #18 or #19 or #20 or #21 or #22 or #23 or #24 or #25
#27	#14 or #26

CINAHL via EBSCO

	Search ID#	Search Terms
☐	S11	S7 OR S10

For peer review only

☐	S10	S1 AND S8
☐	S9	S2 AND S8
☐	S8	S3 AND S4
☐	S7	S5 OR S6
☐	S6	TX "infant early warning" OR TX "bedside PEWS" OR TX "Bedside paediatric early warning"
☐	S5	TX "p?ediatric early warning system" OR TX "P?ediatric Early Warning" OR TX "p?ediatric early warning score" OR TX "p?ediatric risk of mortality" OR TX "P?ediatric Rapid Response Team" OR TX "P?ediatric alert"
☐	S4	AB pediatric* or paediatric* or infant*1 or child* or baby or toddler or babies or teen* or adolescent*
☐	S3	TX "track-and-trigger" OR TX "VitalPAC Early Warning Score" OR TX "activation criteria". OR TX "trigger tool*" OR TX "Rapid Response" OR TX "activation criteria". OR TX "early warning" OR TX "Alert criteria" OR TX outreach N3 emergency
☐	S2	Detecting W3 deterioration
☐	S1	"early warning"

DARE
(Paediatric early warning) OR (pediatric early warning) OR (Paediatric Rapid Response) IN DARE
(early warning) OR (track-and-trigger system) OR (Rapid Response) IN DARE
(emergency team) AND (early warning) IN DARE
HMIC
Database: HMIC Health Management Information Consortium
Search Strategy:
----
1 ("early warning" adj5 scor*).ab,ti. (23)
2 ("early warning" adj5 system* adj5 (deteriorat* or mortality or death or outcome* or harm* or safety)).ab,ti. (6)
3 "acute illness severity".mp. (3)
4 "early medical intervention"/ and ((prevent* or reduc* or improv*) adj5 (deteriorat* or mortality or death or outcome* or harm* or safety)).ab,ti. (0)
5 ("early medical intervention" adj5 (tool* or scor* or index* or indicator* or indice* or assessment* or guide* or instrument* or criteria or parameter* or deteriorat* or mortality or death or monitor* or outcome* or harm* or safety)).ab,ti. (0)
6 Health Status Indicators.mp. and ((tool* or scor* or index* or indicator* or indice* or assessment* or instrument* or criteria or parameter*) adj3 ((prevent* or reduc* or improv*) adj3 (deteriorat* or mortality or death or outcome* or harm* or safety))).ab,ti. (0)
7 exp "Severity of illness index"/ and ((tool* or scor* or index* or indicator* or indice* or assessment* or instrument* or criteria or parameter*) adj5 ((prevent* or reduc* or improv*) adj5 (deteriorat* or mortality or death or outcome* or harm* or safety))).ab,ti. (0)
8 "activation criteria".ab,ti. (2)
9 exp Rapid response teams/ (39)
10 Clinical Alarms.mp. (0)
11 (outreach adj3 emergency).tw. (2)
12 VitalPAC Early Warning Score.tw. (0)
13 medical emergency team.tw. (15)
14 Rapid Response Systems.mp. (8)

For peer review only

15 Rapid Response Team.tw. (27)
16 ((Detecting or managing) adj3 deterioration).tw. (1)
17 track-and-trigger system.tw. (2)
18 (Track adj trigger).tw. (1)
19 (Track and trigger).tw. (8)
20 trigger tools.tw. (4)
21 Calling criteria.tw. (1)
22 Alert criteria.mp. (1)
23 Rapid response.tw. (111)
24 (score adj3 severity of illness).tw. (3)
25 or/1-24 (171)
26 (pediatric* or paediatric* or infant*1 or child* or baby or toddler or babies or teen* or adolescent*).mp. (40161)
27 25 and 26 (14)
28 p?ediatric alert.tw. (0)
29 p?ediatric early warning systems.mp. (1)
30 p?ediatric risk of mortality.tw. (4)
31 Pediatric Rapid Response Team.tw. (0)
32 Point-of-Care.mp. and ((paediatric or pediatric) adj3 (improve or identify or detect* or outcome or early or critical or emergency)).tw. (0)
33 Pediatric Advanced Warning Score.tw. (0)
34 neonatal early warning.tw. (0)
35 infant early warning.tw. (0)
36 paediatric rapid response.tw. (1)
37 pediatric rapid response.tw. (0)
38 Bedside paediatric early warning.tw. (0)
39 Bedside PEWS.tw. (0)
40 p?ediatric early warning.mp. (2)
41 care.mp. and ((paediatric or pediatric) adj3 (improve or identify or detect* or outcome or early or critical or emergency)).tw. [mp=title, other title, abstract, heading words] (57)
42 or/28-41 (59)
43 27 and 42 (3)
44 27 or 42 (70)

Embase

Database: EMBASE <1947-Present>
Search Strategy:
----
1 ("early warning" adj5 scor*).ab,ti. (568)
2 ("early warning" adj5 system* adj5 (deteriorat* or mortality or death or outcome* or harm* or safety)).ab,ti. (51)
3 "acute illness severity".mp. (38)
4 early intervention/ and ((prevent* or reduc* or improv*) adj5 (deteriorat* or mortality or death or outcome* or harm* or safety)).ab,ti. (1185)
5 ("early medical intervention" adj5 (tool* or scor* or index* or indicator* or indice* or assessment* or guide* or instrument* or criteria or parameter* or deteriorat* or mortality or death or monitor* or outcome* or harm* or safety)).ab,ti. (10)
6 *"severity of illness index"/ and ((tool* or scor* or index* or indicator* or indice* or assessment* or instrument* or criteria or parameter*) adj5 ((prevent* or reduc* or improv*) adj5 (deteriorat* or mortality or death or outcome* or harm* or safety))).ab,ti. (3)

For peer review only

7 exp Health Status Indicators/ and ((tool* or scor* or index* or indicator* or indice* or assessment* or instrument* or criteria or parameter*) adj3 ((prevent* or reduc* or improv*) adj3 (deteriorat* or mortality or death or outcome* or harm* or safety))).ab,ti. (7)
8 rapid response team/ (849)
9 "alarm monitor"/ and (prevent* or reduc* or improv*).mp. (245)
10 ("clinical alarm" adj5 (prevent* or reduc* or improv*)).mp. (2)
11 (outreach adj3 emergency).tw. (46)
12 VitalPAC Early Warning Score.tw. (15)
13 medical emergency team.tw. (395)
14 Rapid Response Systems.mp. (140)
15 ("rapid response" adj5 (prevent* or reduc* or improv*)).tw. (191)
16 ("medical device" adj3 (prevent* or reduc* or improv*)).mp. (187)
17 (((Detecting or managing) adj3 deterioration) and warning).tw. (11)
18 track-and-trigger system.tw. (24)
19 (Track adj trigger).tw. (4)
20 (Track and trigger).tw. (241)
21 trigger tools.tw. (47)
22 ("alert criteria" or "activation criteria" or "calling criteria").tw. (209)
23 SBAR technique*.mp. (5)
24 (score adj3 severity of illness).tw. (393)
25 or/1-24 (4295)
26 limit 25 to (infant <to one year> or child <unspecified age> or preschool child <1 to 6 years> or school child <7 to 12 years> or adolescent <13 to 17 years>) (533)
27 P?ediatric Early Warning.mp. (120)
28 p?ediatric alert.tw. (7)
29 p?ediatric early warning systems.mp. (4)
30 p?ediatric risk of mortality.tw. (527)
31 P?ediatric Rapid Response Team.tw. (14)
32 Point-of-Care Systems/ and ((paediatric or pediatric) adj3 (improve or identify or detect* or outcome or early or critical or emergency)).tw. (23)
33 P?ediatric Advanced Warning Score.tw. (3)
34 neonatal early warning.tw. (1)
35 infant early warning.tw. (0)
36 p?ediatric rapid response.tw. (31)
37 Bedside paediatric early warning.tw. (5)
38 Bedside PEWS.tw. (7)
39 or/27-38 (707)
40 26 or 39 (1155)
41 limit 40 to human (1065)

Medline
Database: Ovid MEDLINE(R) <1946 to January Week 2 2015>
Search Strategy:
----
1 ("early warning" adj5 scor*).ab,ti. (260)
2 ("early warning" adj5 system* adj5 (deteriorat* or mortality or death or outcome* or harm* or safety)).ab,ti. (24)
3 "acute illness severity".mp. (21)
4 "early medical intervention"/ and ((prevent* or reduc* or improv*) adj5 (deteriorat* or mortality or death or outcome* or harm* or safety)).ab,ti. (99)

For peer review only

5 ("early medical intervention" adj5 (tool* or scor* or index* or indicator* or indice* or assessment* or guide* or instrument* or criteria or parameter* or deteriorat* or mortality or death or monitor* or outcome* or harm* or safety)).ab,ti. (7)
6 exp Health Status Indicators/ and ((tool* or scor* or index* or indicator* or indice* or assessment* or instrument* or criteria or parameter*) adj3 ((prevent* or reduc* or improv*) adj3 (deteriorat* or mortality or death or outcome* or harm* or safety))).ab,ti. (166)
7 "Severity of Illness Index"/ and ((tool* or scor* or index* or indicator* or indice* or assessment* or instrument* or criteria or parameter*) adj5 ((prevent* or reduc* or improv*) adj5 (deteriorat* or mortality or death or outcome* or harm* or safety))).ab,ti. (274)
8 exp Hospitals/ and ((Detecting or managing) adj3 deterioration).tw. (2)
9 ("medical device" adj3 (prevent* or reduc* or improv*)).mp. (58)
10 ("alert criteria" or "activation criteria" or "calling criteria").tw. (121)
11 Hospital Rapid Response Team/ (334)
12 Clinical Alarms/ (332)
13 (outreach adj3 emergency).tw. (32)
14 VitalPAC Early Warning Score.tw. (10)
15 medical emergency team.tw. (247)
16 Rapid Response Systems.mp. (87)
17 Rapid Response Team.tw. (185)
18 (((Detecting or managing) adj3 deterioration) and warning).tw. (8)
19 track-and-trigger system.tw. (14)
20 (Track adj trigger).tw. (2)
21 (Track and trigger).tw. (137)
22 trigger tools.tw. (22)
23 SBAR technique*.mp. (3)
24 ("rapid response" adj5 (prevent* or reduc* or improv*)).tw. (117)
25 (score adj3 severity of illness).tw. (243)
26 or/1-25 (2286)
27 limit 26 to (humans and "all child (0 to 18 years)") (453)
28 P?ediatric Early Warning.mp. (38)
29 p?ediatric alert.tw. (5)
30 p?ediatric early warning systems.mp. (3)
31 p?ediatric risk of mortality.tw. (400)
32 P?ediatric Rapid Response Team.tw. (6)
33 Point-of-Care Systems/ and ((paediatric or pediatric) adj3 (improve or identify or detect* or outcome or early or critical or emergency)).tw. (79)
34 P?ediatric Advanced Warning Score.tw. (2)
35 neonatal early warning.tw. (0)
36 infant early warning.tw. (0)
37 p?ediatric rapid response.tw. (20)
38 Bedside paediatric early warning.tw. (2)
39 Bedside PEWS.tw. (2)
40 or/28-39 (542)
41 27 or 40 (943)

Scopus
(TITLE-ABS-KEY ("Paediatric Early Warning" OR "Pediatric Early Warning" OR "Pediatric Advanced Warning Score" OR "Paediatric Advanced Warning Score" OR "neonatal early warning" OR "infant early warning" OR "pediatric rapid response" OR "Paedatric rapid response")) OR (((TITLE-ABS-KEY ("early warning" W/5 scor*)) OR (TITLE-ABS-KEY ("Rapid Response")) OR (TITLE-ABS-KEY ("track-and-trigger system")) OR (TITLE-ABS-KEY ("track and trigger")) OR (TITLE-ABS-

For peer review only

KEY ("trigger tool*")) OR (TITLE-ABS-KEY ("alert criteria")) OR (TITLE-ABS-KEY ("activation criteria")) OR (TITLE-ABS-KEY ("VitalPAC Early Warning Score"))) AND (TITLE-ABS-KEY (pediatric* OR paediatric* OR infant* OR child* OR baby OR toddler OR babies OR teen* OR adolescent*))) AND (LIMIT-TO (SUBJAREA , "MEDI") OR LIMIT-TO (SUBJAREA , "NURS") OR LIMIT-TO (SUBJAREA , "NEUR"))

Web of Science

# 19	400	#17 OR #1<br>**Refined by:** [excluding] **WEB OF SCIENCE CATEGORIES:** (PARASITOLOGY OR PUBLIC ENVIRONMENTAL OCCUPATIONAL HEALTH OR BIOCHEMISTRY MOLECULAR BIOLOGY OR OPTICS OR HEALTH CARE SCIENCES SERVICES OR MYCOLOGY OR MANAGEMENT OR LINGUISTICS OR INSTRUMENTS INSTRUMENTATION OR MICROBIOLOGY OR INFORMATION SCIENCE LIBRARY SCIENCE OR MATHEMATICAL COMPUTATIONAL BIOLOGY OR GERIATRICS GERONTOLOGY OR ENGINEERING BIOMEDICAL OR FOOD SCIENCE TECHNOLOGY OR ENVIRONMENTAL STUDIES OR ENGINEERING ENVIRONMENTAL OR ENGINEERING ELECTRICAL ELECTRONIC OR HEALTH POLICY SERVICES OR TOXICOLOGY OR EDUCATION EDUCATIONAL RESEARCH OR NUTRITION DIETETICS OR SUBSTANCE ABUSE OR ECONOMICS OR MEDICINE RESEARCH EXPERIMENTAL OR STATISTICS PROBABILITY OR DEVELOPMENTAL BIOLOGY OR MEDICAL INFORMATICS OR SOCIOLOGY OR DENTISTRY ORAL SURGERY MEDICINE OR PSYCHOLOGY EXPERIMENTAL OR COMPUTER SCIENCE ARTIFICIAL INTELLIGENCE OR METEOROLOGY ATMOSPHERIC SCIENCES OR CHEMISTRY ANALYTICAL OR MEDICAL LABORATORY TECHNOLOGY OR CELL BIOLOGY OR DEMOGRAPHY OR BUSINESS FINANCE OR COMPUTER SCIENCE INTERDISCIPLINARY APPLICATIONS OR AUDIOLOGY SPEECH LANGUAGE PATHOLOGY OR PSYCHOLOGY DEVELOPMENTAL OR COMPUTER SCIENCE INFORMATION SYSTEMS OR PLANNING DEVELOPMENT)<br>Indexes=SCI-EXPANDED, SSCI, CPCI-S, CPCI-SSH<br>Timespan=1900-2015
# 18	499	#17 OR #1<br>Indexes=SCI-EXPANDED, SSCI, CPCI-S, CPCI-SSH<br>Timespan=1900-2015
# 17	487	#16 AND #15<br>Indexes=SCI-EXPANDED, SSCI, CPCI-S, CPCI-SSH<br>Timespan=1900-2015
# 16	8,044	#14 OR #13 OR #12 OR #11 OR #10 OR #9 OR #8 OR #7 OR #6 OR #5 OR #4 OR #3 OR #2<br>Indexes=SCI-EXPANDED, SSCI, CPCI-S, CPCI-SSH<br>Timespan=1900-2015
# 15	1,689,232	**TOPIC:** ((pediatric* OR paediatric* OR infant* OR child* OR baby OR toddler OR babies OR teen* OR adolescent*))

For peer review only

	Indexes=SCI-EXPANDED, SSCI, CPCI-S, CPCI-SSH Timespan=1900-2015
# 130 14	**TOPIC:** ("Severity of Illness Index" and ((tool* or scor* or index* or indicator* or indice* or assessment* or instrument* or criteria or parameter*) SAME ((prevent* or reduc* or improv*) SAME (deteriorat* or mortality or death or outcome* or harm* or safety)))) Indexes=SCI-EXPANDED, SSCI, CPCI-S, CPCI-SSH Timespan=1900-2015
# 63 13	**TOPIC:** (("early medical intervention" SAME (tool* or scor* or index* or indicator* or indice* or assessment* or guide* or instrument* or criteria or parameter* or deteriorat* or mortality or death or monitor* or outcome* or harm* or safety))) Indexes=SCI-EXPANDED, SSCI, CPCI-S, CPCI-SSH Timespan=1900-2015
# 28 12	**TOPIC:** ("early medical intervention" and ((prevent* or reduc* or improv*) SAME (deteriorat* or mortality or death or outcome* or harm* or safety))) Indexes=SCI-EXPANDED, SSCI, CPCI-S, CPCI-SSH Timespan=1900-2015
# 1,206 11	**TOPIC:** ("early warning" SAME system* SAME (deteriorat* or mortality or death or outcome* or harm* or safety)) Indexes=SCI-EXPANDED, SSCI, CPCI-S, CPCI-SSH Timespan=1900-2015
# 2 10	**TOPIC:** ("SBAR technique") Indexes=SCI-EXPANDED, SSCI, CPCI-S, CPCI-SSH Timespan=1900-2015
# 7 9	**TOPIC:** ("VitalPAC Early Warning Score") Indexes=SCI-EXPANDED, SSCI, CPCI-S, CPCI-SSH Timespan=1900-2015
# 123 8	**TOPIC:** ("activation criteria") Indexes=SCI-EXPANDED, SSCI, CPCI-S, CPCI-SSH Timespan=1900-2015
# 16 7	TS=("alert criteria") Indexes=SCI-EXPANDED, SSCI, CPCI-S, CPCI-SSH Timespan=1900-2015
# 159 6	TS=("trigger tool*") Indexes=SCI-EXPANDED, SSCI, CPCI-S, CPCI-SSH Timespan=1900-2015
# 45 5	TS=("track and trigger") Indexes=SCI-EXPANDED, SSCI, CPCI-S, CPCI-SSH Timespan=1900-2015
# 15 4	TS=("track-and-trigger system") Indexes=SCI-EXPANDED, SSCI, CPCI-S, CPCI-SSH Timespan=1900-2015
# 6,100 3	TS=("Rapid Response") Indexes=SCI-EXPANDED, SSCI, CPCI-S, CPCI-SSH Timespan=1900-2015
# 604	TS=("early warning" SAME scor*)

For peer review only

2 Indexes=SCI-EXPANDED, SSCI, CPCI-S, CPCI-SSH
Timespan=1900-2015

88
1

TS=("Paediatric Early Warning" OR "Pediatric Early Warning" OR "Pediatric Advanced Warning Score" OR "Paediatric Advanced Warning Score" OR "neonatal early warning" OR "infant early warning" OR "pediatric rapid response" OR "Paedatric rapid response")
Indexes=SCI-EXPANDED, SSCI, CPCI-S, CPCI-SSH
Timespan=1900-2015

PUMA Supplementary searches

Search terms to use:
"Pediatric Early warning"
"Paediatric Early warning"
“Pediatric Rapid Response Team”
“Paediatric Rapid Response Team”
PEWS
“Paediatric trigger tools”
“Pediatric trigger tools”

For peer review only

Supplementary table 2 - study characteristics of PTTS validation studies

First author	Year	Full text?	PTTS name	Based on	Modification from original version				Score / trigger	Study design	Bench tested / implemented?	Data collection (intervention period)	Primary outcome measure	Country	Single / multi centre	Setting	Study population	Inclusion criteria (case / cohort)	Inclusion criteria (control)	Sample size (overall)	Sample size (cases / adverse events)
					Added items	Removed items	Modified thresholds	Modified escalation algorithm													
Duncan	2006	Y	Paediatric Early Warning System score						Score	Case-control	No	Retrospective	Arrest / code call	Canada	Single	Tertiary / specialist centre	All in-patients	Code blue call	No code blue call or PICU admission within 48 hours of study duration	215	128
Bell	2013	Y	Texas Children's Hospital (TCH) PAWS	Brighton PEWS	✓	✓	✓	✓	Score			Retrospective		US	Single	Tertiary / specialist centre	General medicine & two specialist wards			150	
Edwards	2008	Y	Cardiff & Vale PEWS	Melbourne Activation Criteria					Score	Cohort	No	Prospective	PICU / HDU transfer, arrest or death	UK	Single	Tertiary / specialist centre	All in-patients	Any in-patient admission		1,000	16
Egdell	2008	Y	Paediatric Advance Warning Score (PAWS)						Score	Case-control	No	Retrospective	PICU admission	UK	Single	Tertiary / specialist centre	Emergency department only	PICU admission direct from ED	Ward admission direct from ED		
Fuijkschot (Study 1)	2015	Y	Modified PEWS	Bedside PEWS	✓		✓		Score	Case-cohort		Retrospective		Netherlands	Single	Tertiary / specialist centre	Specialist ward only (oncology)			118	15
Fuijkschot (Study 2)	2015	Y	Modified PEWS	Bedside PEWS	✓		✓		Score	Case-cohort		Retrospective		Netherlands	Single	Tertiary / specialist centre	All in-patients			Unclear	24
Fuijkschot (Study 3)	2015	Y	Modified PEWS	Bedside PEWS	✓		✓		Score	Case-cohort		Prospective		Netherlands	Single	Tertiary / specialist centre	All in-patients			Unclear	14
Clayson	2014	N	Adjusted PEWT	Bristol PEWT			✓		Trigger	Cohort	Unclear	Prospective	Unclear	UK	Single	Tertiary / specialist centre	Specialist ward only (cardiac)	Unclear		126	
Rahman	2014	N	Burn-specific PEWS	Brighton PEWS	✓		✓		Score	Case / chart review	Unclear	Retrospective	Unclear	US	Single	Tertiary / specialist centre	Specialist ward only (burns unit)	Non-intubated, LOS > 3 days		50	
Wright	2011	N	Paediatric Early Warning Tool (PEWT)	Bristol PEWT					Trigger	Case / chart review	Yes	Retrospective	Cardiac arrest	UK	Single	Tertiary / specialist centre	All in-patients	All cardiac arrest calls		55	
Hopkins	2013	N	Modified PEWS	Bedside PEWS	7-item version				Score	Case-control	Unclear	Retrospective	PICU transfer following code/RRT call	US	Single	Tertiary / specialist centre	All in-patients	Code/RRT calls requiring PICU transfer	Code / RRT calls remaining on ward	66	47
Gawronski	2013	N	Bedside PEWS		Unclear				Score	Case-control	No	Retrospective	PICU transfer	Italy	Single	Tertiary / specialist centre	Specialist ward only (bone marrow transplant unit)	PICU transfer / review / death	No transfer / review / death during stay	11	10
Haines	2006	Y	Bristol PEWT						Trigger	Case-control (only cases analysed)	No	Prospective	PICU / HDU admission, arrest or death	UK	Single	Tertiary / specialist centre	All in-patients	Patients who triggered PEWT during admission	Patients who did not trigger PEWT during admission	360	180
Garlick	2013	N	Modified PEWS	Brighton PEWS	✓				Score	Case / chart review	Unclear	Retrospective	PICU transfer	US	Single	Tertiary / specialist centre	All in-patients	Patients who had MET activation		267	
Ahmed	2012	N	Burton PEWS	Unclear					Score	Case / chart review	No	Retrospective	PICU transfer	UK	Single	Unclear	Unclear	All PICU transfers		23	23

For peer review only

Akre	2010	Y	Paediatric Early Warning Score	Brighton PEWS			✓		Score	Case / chart review	No	Retrospective	Code / RRT call	US	Single	Tertiary / specialist centre	All in-patients	Code or RRT call		186	186
Edwards	2011	Y	Melbourne Activation Criteria						Trigger	Cohort	No	Retrospective	PICU/HDU transfer, arrest, death	UK	Single	Tertiary / specialist centre	All in-patients	Any in-patient admission		1,000	16
McLellan	2013	Y	Cardiac CHEWS	Brighton PEWS	✓		✓	✓	Score	Pilot study	Yes			US	Single	Tertiary / specialist centre	Specialist unit only (cardiac)				
McLellan	2014	Y	Cardiac CHEWS	Brighton PEWS	✓		✓	✓	Score	Case-control	Yes	Prospective	PICU transfer / cardiac arrest	US	Single	Tertiary / specialist centre	Specialist unit only (cardiac)	PICU transfer / cardiac arrest	No transfer or arrest	312	64
Skaletzky	2012	Y	Modified Paediatric Early Warning Score	Brighton PEWS			✓		Score	Case-control	No	Retrospective	PICU transfer	US	Single	Tertiary / specialist centre	Medical-surgical ward only	PICU transfer	No transfer	350	100
Zhai	2014	Y	Automated algorithm						Score	Case-control	No	Retrospective	PICU transfer	US	Single	Tertiary / specialist centre	All in-patients	PICU transfer within 24 hr of ward admission	No PICU transfer	7,298 (measurements)	526 (measurements)
Zhai	2014	Y	Brighton PEWS	Brighton PEWS					Score	Case-control	No	Retrospective	PICU transfer	US	Single	Tertiary / specialist centre	All in-patients	PICU transfer within 24 hr of ward admission	No PICU transfer	7,298 (measurements)	526 (measurements)
Zhai	2014	Y	Bedside PEWS	Bedside PEWS					Score	Case-control	No	Retrospective	PICU transfer	US	Single	Tertiary / specialist centre	All in-patients	PICU transfer within 24 hr of ward admission	No PICU transfer	7,298 (measurements)	526 (measurements)
Bonafide	2012	Y	7-item score (non-vital signs)						Score	Case-control	No	Retrospective	Arrest, resp compromise or urgent PICU transfer	US	Single	Tertiary / specialist centre	All in-patients	Arrest, resp compromise or urgent PICU transfer	On in-patient ward at same time as matched case	564	141
Sefton	2014	N	Modified Bristol PEWT	Bristol PEWT	Unclear				Score	Chart review	Unclear	Retrospective	Arrest, PICU transfer or unexpected death	UK	Single	Tertiary / specialist centre	Unclear			Unclear	Unclear
Parshuram	2011	Y	Bedside PEWS	Bedside PEWS	7-item				Score	Case-control	No	Prospective		UK / Canada	Multi	Tertiary / specialist centre	All in-patients	Urgent PICU transfer or call to resscuc team	No PICU transfer or ressucc call within 48 hours	2,074	686
Parshuram	2009	Y	Bedside PEWS	Paediatric Early Warning System (PEWS) score		✓			Score	Case-control	No	Retrospective	Urgent PICU transfer	Canada	Single	Tertiary / specialist centre	All in-patients	Urgent PICU transfer (not from 'code blue')	No 'code blue' or urgent PICU transfer within 48 hours	180	60
Tucker	2009	Y	Modified Paediatric Early Warning Score	Brighton PEWS				✓	Score	Cohort	Yes	Prospective	PICU transfer	US	Single	Tertiary / specialist centre	General medical unit only	PICU transfer		2,979	51
Tume	2007	Y	Bristol PEWT						Trigger	Chart review	No	Retrospective	Unplanned PICU / PHDU transfer	UK	Single	Tertiary / specialist centre	Unplanned PICU/PHDU transfers only	Unplanned PICU / PHDU transfer		29	29
Tume	2007	Y	Melbourne Activation Criteria						Trigger	Chart review	No	Retrospective	Unplanned PICU / PHDU transfer	UK		Tertiary / specialist centre	Unplanned PICU/PHDU transfers only	Unplanned PICU / PHDU transfer		29	29
O'Loughlin	2012	N	Paediatric Early Warning Tool	Bristol PEWT					Trigger	Cohort	Yes	Prospective	PICU transfer	UK	Single	Tertiary / specialist centre	All in-patients	Any in-patient admission		331	7

For peer review only

Robson	2013	Y	Paediatric Early Warning Tool	Bristol PEWT					Trigger	Case-control	No	Retrospective	Code call / arrest	US	Single	Tertiary / specialist centre	All in-patients	Code call / arrest	No code call / arrest	192	96
Robson	2013	Y	PEW System Score						Score	Case-control	No	Retrospective	Code call / arrest	US	Single	Tertiary / specialist centre	All in-patients	Code call / arrest	No code call / arrest	192	96
Robson	2013	Y	Bedside PEWS						Score	Case-control	No	Retrospective	Code call / arrest	US	Single	Tertiary / specialist centre	All in-patients	Code call / arrest	No code call / arrest	192	96
Dean	2015	N	Unclear	Unclear					Unclear	Cohort	Unclear	Prospective	PICU transfer / CD event	US	Single	Unclear	All in-patients	PICU transfer / CD event		Unclear	Unclear
Fenix	2015	Y	Modified Paediatric Early Warning Score	Brighton PEWS			✓	✓	Score	Case-control	Yes	Retrospective	PICU transfer / CD event	US	Single	Tertiary / specialist centre	All in-patients, except: haematology-oncology, surgical and cardiac	PICU transfer followed by CD event	PICU transfer but no CD event	97	51
Lin	2015	N	Automated EHR-based tool						Score	Case-control	No	Retrospective	PICU transfer; Code call / arrest; senior review	US	Single	Tertiary / specialist centre	All in-patients	PICU transfer; code call / arrest; senior review	No transfer / arrest / review	2,310	701
Mandell	2015	Y	Modified Paediatric Early Warning Score	Brighton PEWS	✓		✓	✓	Score	Case-control	Yes	Prospective	PICU re-admission	US	Single	Tertiary / specialist centre	PICU discharge patients only	PICU discharge to ward and re-admission to PICU within 48 hours	PICU discharge to ward and no re-admission to PICU within 48 hours	189	38
Medar	2015	N	Unclear	Unclear					Unclear	Case / chart review	No	Retrospective	RRT/MET call	Unclear	Single	Unclear	All in-patients with RRT calls	RRT call		61	61
Ross	2015	N	Bedside PEWS				✓		Score	Case-control	No	Retrospective	Urgent ICU transfer	US	Single	Tertiary / specialist centre	All in-patients	Urgent ICU transfer	No ICU transfer	4628	848
Gawronski	2016	Y	Bedside PEWS	7-item						Case-control (nested)	No	Prospective	Urgent ICU transfer; RRT / MET call; death	Italy	Single	Tertiary / specialist centre	Specialist unit only (stem-cell transplant)	Urgent ICU transfer; RRT / MET call; death	No transfers, RRT call or death	99	19
Mason	2016	Y	NHS III PEWS						Score	Cohort	No	Prospective	PICU / HDU transfer, arrest or death	UK	Single	Tertiary / specialist centre	All in-patients	Any in-patient admission		1,000	16

For peer review only

Supplementary table 3 - study characteristics of effectiveness studies

			Intervention															
**First author**	**Year**	**Full text?**	**New PTTS**	**New RRT / MET**	**New escalation process linked to PTTS**	**Staff training / education package**	**Type of PTTS**	**Modification from original version**	**Existing RRT / MET?**	**Primary outcome measure**	**Study design / analysis**	**Data collection (intervention period)**	**Country**	**Setting**	**Study population**	**Single / multi centre**	**Pre-intervention period (mths)**	**Post-intervention period (mths)**
Camacho	2011	N	✓				Brighton PEWS	Modified thresholds / definitions	Unclear	Arrests / code calls	Before & after study	Prospective	US	Tertiary / specialist	Specialist ward only (cardiac & renal)	Single	3	5
Demmel	2010	Y	✓		✓	✓	Brighton PEWS	Modified escalation algorithm; added "exception algorithm"	Yes	Arrests / code calls	Before & after study	Prospective	US	Tertiary / specialist	Specialist ward only (haematology & oncology)	Single	Unclear	8
Panesar	2014	Y			✓		Brighton PEWS	Modified thresholds / definitions; mandated RRT call for high scores	No	RRT/MET calls	Before & after study	Retrospective	US	Tertiary / specialist	All in-patients	Single	18	24
Sandhu	2010	N	✓	✓			Unclear		Yes	Arrests / code calls	Before & after study	Retrospective	UK	Tertiary / specialist	*Unclear*	Single	Unclear	3
Ambati	2014	N				✓	N/A		Yes	RRT/MET calls	Before & after study	Unclear	US	Tertiary / specialist	*Unclear*	Single	12	36
Duns	2014	N	✓				In-house activation criteria ("Between the Flags")		Yes	RRT/MET calls	Before & after study	Prospective	Australia	Tertiary / specialist	*Unclear*	Single	24	24
Hanson	2010	Y	✓	✓		✓	In-house activation criteria		No	Arrests / code calls	Interrupted Time Series	Retrospective	US	Tertiary / specialist	All in-patients	Single	11	13
Heyden	2012	N	✓	✓			In-house activation criteria		No	Arrests / code calls	Before & after study	Unclear	US	Tertiary / specialist	All in-patients	Single	24	48
Hunt	2008	Y	✓	✓			In-house activation criteria		No	Arrests / code calls	Before & after study	Prospective	US	Tertiary / specialist	All in-patients	Single	12	12
Sornberg	2013	N	✓	✓			Unclear		Unclear	Arrests / code calls	Before & after study	Unclear	US	Tertiary / specialist	*Unclear*	Single	Unclear	
Ocholi	2014	N	✓		✓		Bedside PEWS	Unclear	Unclear	Acuity at PICU admission	Before & after study	Unclear	UK	Tertiary / specialist	*Unclear*	Single	6	6
Parshuram	2011	Y	✓		✓	✓	Bedside PEWS		No	"Clinical deterioration" events	Before & after study	Prospective	Canada	Community / DGH / non-specialist	All in-patients	Single	3	5
Norville	2013	N	✓		✓	✓	Paediatric Advance Warning Score (PAWS)	Modified escalation algorithm	Yes	Arrests / code calls	Before & after study	Unclear	US	Tertiary / specialist	Specialist ward only (Bone Marrow Transplant Unit)	Single	12	11
Bonafide	2014	Y	✓	✓	✓		Bedside PEWS		No	"Critical deterioration" events	Interrupted Time Series	Prospective	US	Tertiary / specialist	All in-patients	Single	32	27
Kotsakis	2011	Y	✓	✓		✓	Melbourne Activation Criteria	Removed items	No	Arrests / code calls	Before & after study	Prospective	Canada	Tertiary / specialist	All in-patients	Multi	24	24
Mistry	2006	Y	✓	✓		✓	In-house activation criteria		No	Arrests / code calls	Before & after study	Prospective	US	Tertiary / specialist	All in-patients	Single	6	5
Randhawa	2011	Y	✓		✓	✓	Brighton PEWS*	Modified escalation algorithm	Yes	Arrests / code calls	Before & after study	Prospective	US	Tertiary / specialist	All in-patients	Single	12	12
Sharek	2007	Y	✓	✓		✓	Melbourne Activation Criteria	Removed items; modified thresholds /	No	Mortality; Arrests / code calls	Before & after study	Prospective	US	Tertiary / specialist	All in-patients	Single	67	17

For peer review only

								definitions										
Tibballs	2005	Y	✓	✓		✓	Melbourne Activation Criteria		No	Mortality; Arrests / code calls	Before & after study	Prospective	Australia	Tertiary / specialist	All in -patients	Single	41	12
Tibballs	2009	Y	✓	✓		✓	Melbourne Activation Criteria		No	Mortality; Arrests / code calls	Before & after study	Prospective	Australia	Tertiary / specialist	All in-patients	Single	41	48
Anwar	2010	Y	✓	✓			In-house calling criteria		No	Arrests / call codes	Before & after study	Retrospective	Pakistan	Tertiary / specialist	All in-patients	Single	9	9
Zenker	2007	Y	✓	✓			In-house calling criteria		No	Mortality; Arrests / code calls	Before & after study	Prospective	US	Tertiary / specialist	All in –patients	Single	23	11
Sefton	2014	Y	✓		✓	✓	Bristol PEWS		No	Acuity at PICU admission	Before & after study	Retrospective	UK	Tertiary / specialist	All in-patients	Single	12	12
Douglas	2016	Y	✓		✓	✓	Brighton PEWS	Modified thresholds / definitions	Yes	RRT/MET calls	Before & after study	Retrospective	US	Tertiary / specialist	All in-patients	Single	12	12

For peer review only

Supplementary table 4 - findings of PTTS validation studies

First author	Year	Full text?	Type of PTTS	Based on	Score / trigger	Study design / analysis	Country	Setting	Study population	Single / multi centre	Bench tested / implemented	Sample size	No of cases / adverse events	Outcome							Performance								
														Mortality	Arrest / code call	PICU / PHDU transfer	RRT / MET call	Critical intervention	Senior review	Other	Threshold reported	AUROC	Sensitivity	Specificity	PPV	NPV	IRR	Adherence	Notes
Duncan	2006	Y	Paediatric Early Warning System score		Score	Case-control	Canada	Tertiary / specialist centre	All in-patients	Single	Bench tested	215	128		✓						5	0.90	78.0	95.0	4.2	-		Yes	
Bell	2013	Y	Texas Children's Hospital (TCH) PAWS	Brighton PEWS	Score	Chart review	US	Tertiary / specialist centre	General medicine & two specialist wards	Single	Implemented	150									5						Y	No	Reliability statistics only
Edwards	2008	Y	Cardiff & Vale PEWS	Melbourne Activation Criteria	Score	Cohort	UK	Tertiary / specialist centre	All in-patients	Single	Bench tested	1,000	16	✓	✓	✓					2	0.86	69.5	89.9	5.9	99.7	-	Yes	
Egdell	2008	Y	Paediatric Advance Warning Score (PAWS)		Score	Case-control	UK	Tertiary / specialist centre			Bench tested					✓					3	0.86	70.0	90.0				No	
Fuijkschot (Study 1)	2015	Y	Modified PEWS	Bedside PEWS	Score		Netherlands				Implemented	118	15					✓	✓	✓	8				73.0			Yes	
Fuijkschot (Study 2)	2015	Y	Modified PEWS	Bedside PEWS	Score		Netherlands				Implemented	Unclear	24			✓					8		66.6					No	
Fuijkschot (Study 3)	2015	Y	Modified PEWS	Bedside PEWS	Score		Netherlands				Implemented	Unclear	14					✓			8		100.0					No	
Clayson	2014	N	Adjusted PEWT	Bristol PEWT	Trigger	Cohort	UK	Tertiary / specialist centre	Specialist ward only (cardiac)	Single	Implemented	126									-				12.5	97.0		No	
Rahman	2014	N	Burn-specific PEWS	Brighton PEWS	Score	Case / chart review	US	Tertiary / specialist centre	Specialist ward only (burns unit)	Single	Unclear	50																Yes	Descriptive statistics only
Wright	2011	N	Paediatric Early Warning Tool (PEWT)	Bristol PEWT	Trigger	Case / chart review	UK	Tertiary / specialist centre	All in-patients	Single	Implemented	55			✓						-		49.1					Yes	
Hopkins	2013	N	Modified PEWS	Bedside PEWS	Score	Case-control	US	Tertiary / specialist centre	All in-patients	Single	Unclear	66	47			✓		✓										No	Descriptive statistics only
Gawronski	2013	N	Bedside PEWS		Score	Case-control	Italy	Tertiary / specialist centre	Specialist ward only (bone marrow transp	Single	Bench tested	11	10			✓												No	Descriptive statistics only

For peer review only

									lant unit)																				
Haines	2006	Y	Bristol PEWT		Trigger	Case-control (only cases analysed)	UK	Tertiary / specialist centre	All in-patients	Single	Bench tested	360	180	✓	✓	✓												No	Descriptive statistics only
Garlick	2013	N	Modified PEWS	Brighton PEWS	Score	Case / chart review	US	Tertiary / specialist centre	All in-patients	Single	Unclear	267				✓												No	Descriptive statistics only
Ahmed	2012	N	Burton PEWS	Unclear	Score	Case / chart review	UK	Unclear	Unclear	Single	Bench tested	23	23			✓							93.0					No	
Akre	2010	Y	Paediatric Early Warning Score	Brighton PEWS	Score	Case / chart review	US	Tertiary / specialist centre	All in-patients	Single	Bench tested	186	186		✓		✓						85.5					Yes	
Edwards	2011	Y	Melbourne Activation Criteria		Trigger	Cohort	UK	Tertiary / specialist centre	All in-patients	Single	Bench tested	1,000	16	✓	✓	✓					-	0.79	68.3	83.2	3.6	99.7		Yes	
McLellan	2013	Y	Cardiac CHEWS	Brighton PEWS	Score	Pilot study – descriptive	US	Tertiary / specialist centre	Specialist unit only (cardiac)	Single	Implemented																		Experience of implementing, no formal evaluation
McLellan	2014	Y	Cardiac CHEWS	Brighton PEWS	Score	Case-control	US	Tertiary / specialist centre	Specialist unit only (cardiac)	Single	Implemented	312	64		✓	✓					3	0.86	95.3	76.2	50.8	98.4	Y	No	
Skaletzky	2012	Y	Modified Paediatric Early Warning Score	Brighton PEWS	Score	Case-control	US	Tertiary / specialist centre	Medical-surgical ward only	Single	Bench tested	350	100		✓	✓	✓				2.5	0.81	62.0	89.0				No	
Zhai	2014	Y	Prediction algorithm		Score	Case-control	US	Tertiary / specialist centre	All in-patients	Single	Bench tested	7,298 (measurements)	526 (measurements)			✓					-	0.91	84.9	85.9	4.8			Yes	
Zhai	2014	Y	Brighton PEWS	Brighton PEWS	Score	Case-control	US	Tertiary / specialist centre	All in-patients	Single	Bench tested	7,298 (measurements)	526 (measurements)			✓					2	0.74	68.4	81.6	2.3			Yes	
Zhai	2014	Y	Bedside PEWS	Bedside PEWS	Score	Case-control	US	Tertiary / specialist centre	All in-patients	Single	Bench tested	7,298 (measurements)	526 (measurements)			✓					7	0.82	73.6	71.7	2.1			Yes	
Bonafide	2012	Y	7-item score (non-vital signs)		Score	Case-control	US	Tertiary / specialist centre	All in-patients	Single	Bench tested	564	141		✓	✓						0.78						No	
Sefton	2014	N	Modified Bristol PEWT	Bristol PEWT	Score	Chart review	UK	Tertiary / specialist centre	Unclear	Single	Unclear	Unclear	Unclear	✓	✓	✓												No	Individual components odds ratios
Parshuram	2011	Y	Bedside PEWS	Bedside PEWS	Score	Case-control	UK / Canada	Tertiary / specialist centre	All in-patients	Multi	Bench tested	2,074	686		✓	✓					7	0.87	64.0	91.0				Yes	

Parshuram	2009	Y	Bedside PEWS	Paediatric Early Warning System (PEWS) score	Score	Case-control	Canada	Tertiary / specialist centre	All in-patients	Single	Bench tested	180	60			✓					8	0.91	82.0	93.0				Yes	
Tucker	2009	Y	Modified Paediatric Early Warning Score	Brighton PEWS	Score	Cohort	US	Tertiary / specialist centre	General medical unit only	Single	Implemented	2,979	51			✓					3	0.89	90.2	74.4	5.8	99.8	Y	No	
Tume	2007	Y	Bristol PEWT		Trigger	Chart review	UK	Tertiary / specialist centre	Unplanned PICU/ PHDU transfers only	Single	Bench tested	29	29			✓							88.0					No	
Tume	2007	Y	Melbourne Activation Criteria		Trigger	Chart review	UK	Tertiary / specialist centre	Unplanned PICU/ PHDU transfers only	Single	Bench tested	29	29			✓							88.0					No	
O'Loughlin	2012	N	Paediatric Early Warning Tool	Bristol PEWT	Trigger	Cohort	UK	Tertiary / specialist centre	All in-patients	Single	Implemented	331	7			✓					-	0.91	100.0	81.0	11.2			No	
Robson	2013	Y	Paediatric Early Warning Tool	Bristol PEWT	Trigger	Case-control	US	Tertiary / specialist centre	All in-patients	Single	Bench tested	192	96		✓						-	0.75	76.3	61.5					
Robson	2013	Y	PEW System Score		Score	Case-control	US	Tertiary / specialist centre	All in-patients	Single	Bench tested	192	96		✓						5	0.85	86.6	72.2					
Robson	2013	Y	Bedside PEWS		Score	Case-control	US	Tertiary / specialist centre	All in-patients	Single	Bench tested	192	96		✓						7	0.73	56.3	78.1					
Dean	2015	N	Unclear	Unclear	Unclear	Cohort	US	Unclear	General medical	Single	Unclear	Unclear	Unclear			✓		✓			Unclear	0.83							
Fenix	2015	Y	Modified Paediatric Early Warning Score	Brighton PEWS	Score	Case-control	US	Tertiary / specialist centre	All in-patients, except: haematology - oncology, surgical and cardiac	Single	Implemented	97	51			✓		✓			3		80.0	43.0					
Lin	2015	N	Automated EHR-based tool		Score	Case-control	US	Tertiary / specialist centre	All in-patients	Single	Bench tested	2,310	701		✓	✓			✓		0.45/1.0	0.93	97.0	85.0	15.0			No	
Mandell	2015	Y	Modified Paediatric Early Warning Score	Brighton PEWS	Score	Case-control	US	Tertiary / specialist centre	PICU discharge patients only	Single	Implemented	189	38							✓	2	0.77	71.0	58.0				No	

For peer review only

Medar	2015	N	Unclear	Unclear	Unclear	Case / chart review	Unclear	Unclear	All in-patients with RRT calls	Single	Bench tested	61	61				✓				3		57.3					No	
Ross	2015	N	Bedside PEWS		Score	Case-control	US	Tertiary / specialist centre	All in-patients	Single	Bench tested	4628	848			✓					Unclear		70.0	84.0				No	Did not use resp data
Gawronski	2016	Y	Bedside PEWS	7-item	Score	Case-control (nested)	Italy	Tertiary / specialist centre	Specialist unit only (stem-cell transplant)	Single	Bench tested	99	19	✓		✓	✓				6		79.0	97.5				No	
Mason	2016	Y	NHS III PEWS		Score	Cohort	UK	Tertiary / specialist centre	All in-patients	Single	Bench tested	1,000	16	✓	✓	✓					2	0.83	73.2	75.2	2.6	99.7		Yes	

For peer review only

Supplementary table 5 – findings of effectiveness studies

			Intervention														Reported outcomes						
First author	Year	Full text?	New PTTS	New RRT / MET	New escalation process linked to PTTS	Staff training / education package	Type of PTTS	Modification from original version	Existing RRT / MET?	Study design / analysis	Country	Setting	Study population	Single / multi centre	Pre-intervention period (mths)	Post-intervention period (mths)	Mortality	Arrests / code calls	PICU / PHDU transfers	Acuity on PICU admission	Critical interventions	RRT/MET calls	Denominator
Camacho	2011	N	✓				Brighton PEWS	Modified thresholds / definitions	Unclear	Before & after study	US	Tertiary / specialist	Specialist ward only (cardiac & renal)	Single	3	5							
Demmel	2010	Y	✓		✓	✓	Brighton PEWS	Modified escalation algorithm; added "exception algorithm"	Yes	Before & after study	US	Tertiary / specialist	Specialist ward only (haematology & oncology)	Single	Unclear	8							
Panesar	2014	Y			✓		Brighton PEWS	Modified thresholds / definitions; mandated RRT call for high scores	No	Before & after study	US	Tertiary / specialist	All in-patients	Single	18	24							
Sandhu	2010	N	✓	✓			Unclear		Yes	Before & after study	UK	Tertiary / specialist	*Unclear*	Single	Unclear	3							
Ambati	2014	N				✓	N/A		Yes	Before & after study	US	Tertiary / specialist	*Unclear*	Single	12	36							
Duns	2014	N	✓				In-house activation criteria ("Between the Flags")		Yes	Before & after study	Australia	Tertiary / specialist	*Unclear*	Single	24	24	↓					↑	Per 1,000 ward admissions
Hanson	2010	Y	✓	✓		✓	In-house activation criteria		No	Interrupted Time Series	US	Tertiary / specialist	All in-patients	Single	11	13	↓	↓					Per 1,000 ward admissions
Heyden	2012	N	✓	✓			In-house activation criteria		No	Before & after study	US	Tertiary / specialist	All in-patients	Single	24	48		↓** (arrests)				↑	None used
Hunt	2008	Y	✓	✓			In-house activation criteria		No	Before & after study	US	Tertiary / specialist	All in-patients	Single	12	12		↓* (respiratory arrests only)					Per 1,000 patient-days
Sornberg	2013	N	✓	✓			Unclear		Unclear	Observational study	US	Non-specialist	*Unclear*	Single	Unclear								
Ocholi	2014	N	✓		✓		Bedside PEWS	Unclear	Unclear	Before & after study	UK	Tertiary / specialist	*Unclear*	Single	6	6							
Parshuram	2011	Y	✓		✓	✓	Bedside PEWS		No	Before & after study	Canada	Non-specialist	All in-patients	Single	3	5			↑* (transfers to external specialist centre)		↓** (critical intervention prior to transfer)		Per 1,000 patient-days
Norville	2013	N	✓		✓	✓	Brighton PEWS	Modified thresholds / definitions; modified escalation algorithm	Yes	Before & after study	US	Tertiary / specialist	Specialist ward only (Bone Marrow Transplant Unit)	Single	12	11							
Bonafide	2014	Y	✓	✓	✓		Bedside PEWS		No	Interrupted Time Series	US	Tertiary / specialist	All in-patients	Single	32	27		↓	↑		↓** (critical intervention within 12hrs of PICU admission)		Per 1,000 patient-days
Kotsakis	2011	Y	✓	✓		✓	Melbourne Activation Criteria	Removed items; modified escalation	No	Before & after study	Canada	Tertiary / specialist	All in-patients	Multi	24	24		↓** (code blue calls but not actual arrests)					Per 1,000 ward admissions

								algorithm															
Mistry	2006	Y	✓	✓		✓	In-house activation criteria		No	Before & after study	US	Tertiary / specialist	All in-patients	Single	6	5	↓** (non PICU inpatient deaths)	↓** (cardiac arrests)					None used
Randhawa	2011	Y	✓		✓	✓	Brighton PEWS*	Modified escalation algorithm	Yes	Before & after study	US	Tertiary / specialist	All in-patients	Single	24			↓					Per 1,000 patient-days
Sharek	2007	Y	✓	✓		✓	Melbourne Activation Criteria	Removed items; modified thresholds / definitions	No	Before & after study	US	Tertiary / specialist	All in-patients	Single	67	17	↓** (hospital-wide mortality rate)	↓** (code call requiring critical intervention)					Per 1,000 patient-days (codes); Per 100 discharges (deaths)
Tibballs	2005	Y	✓	✓		✓	Melbourne Activation Criteria		No	Before & after study	Australia	Tertiary / specialist	All in -patients	Single	41	12	↓	↓	↑				
Tibballs	2009	Y	✓	✓		✓	Melbourne Activation Criteria		No	Before & after study	Australia	Tertiary / specialist	All in-patients	Single	41	48	↓** (total hospital deaths)	↓* ('preventable' cardiac arrests only)					Per 1,000 admissions
Anwar	2010	Y	✓	✓			In-house calling criteria		No	Before & after study	Pakistan	Tertiary / specialist	All in-patients	Single	9	9							
Zenker	2007	Y	✓	✓			In-house calling criteria		No	Before & after study	US	Tertiary / specialist	All in –patients	Single	23	11		↓					Per 1,000 discharges
Sefton	2014	Y	✓		✓	✓	Bristol PEWT		No	Before & after study	UK	Tertiary / specialist	All in-patients	Single	12	12				↓** (PIM2 median)	↓** (critical intervention during PICU admission)		Percentage of all PICU admissions
Douglas	2016	Y	✓		✓	✓	Brighton PEWS	Modified thresholds / definitions	Yes	Before & after study	US	Tertiary / specialist	All in-patients	Single	12	12		↓				↑** (RRT calls)	Per 1,000 patient days

For peer review only

PRISMA 2009 Checklist

Section/topic	#	Checklist item	Reported on page #
**TITLE**			
Title	1	Identify the report as a systematic review, meta-analysis, or both.	1
**ABSTRACT**			
Structured summary	2	Provide a structured summary including, as applicable: background; objectives; data sources; study eligibility criteria, participants, and interventions; study appraisal and synthesis methods; results; limitations; conclusions and implications of key findings; systematic review registration number.	3-4
**INTRODUCTION**			
Rationale	3	Describe the rationale for the review in the context of what is already known.	4-5
Objectives	4	Provide an explicit statement of questions being addressed with reference to participants, interventions, comparisons, outcomes, and study design (PICOS).	5
**METHODS**			
Protocol and registration	5	Indicate if a review protocol exists, if and where it can be accessed (e.g., Web address), and, if available, provide registration information including registration number.	6
Eligibility criteria	6	Specify study characteristics (e.g., PICOS, length of follow-up) and report characteristics (e.g., years considered, language, publication status) used as criteria for eligibility, giving rationale.	6
Information sources	7	Describe all information sources (e.g., databases with dates of coverage, contact with study authors to identify additional studies) in the search and date last searched.	6-7
Search	8	Present full electronic search strategy for at least one database, including any limits used, such that it could be repeated.	Supp table 1
Study selection	9	State the process for selecting studies (i.e., screening, eligibility, included in systematic review, and, if applicable, included in the meta-analysis).	7
Data collection process	10	Describe method of data extraction from reports (e.g., piloted forms, independently, in duplicate) and any processes for obtaining and confirming data from investigators.	7
Data items	11	List and define all variables for which data were sought (e.g., PICOS, funding sources) and any assumptions and simplifications made.	7
Risk of bias in individual studies	12	Describe methods used for assessing risk of bias of individual studies (including specification of whether this was done at the study or outcome level), and how this information is to be used in any data synthesis.	7
Summary measures	13	State the principal summary measures (e.g., risk ratio, difference in means).	NA
Synthesis of results	14	Describe the methods of handling data and combining results of studies, if done, including measures of consistency (e.g., I^2) for each meta-analysis.	NA

For peer review only - http://bmjopen.bmj.com/site/about/guidelines.xhtml

PRISMA 2009 Checklist

Section/topic	#	Checklist item	Reported on page #
Risk of bias across studies	15	Specify any assessment of risk of bias that may affect the cumulative evidence (e.g., publication bias, selective reporting within studies).	NA
Additional analyses	16	Describe methods of additional analyses (e.g., sensitivity or subgroup analyses, meta-regression), if done, indicating which were pre-specified.	NA
**RESULTS**			
Study selection	17	Give numbers of studies screened, assessed for eligibility, and included in the review, with reasons for exclusions at each stage, ideally with a flow diagram.	8
Study characteristics	18	For each study, present characteristics for which data were extracted (e.g., study size, PICOS, follow-up period) and provide the citations.	8
Risk of bias within studies	19	Present data on risk of bias of each study and, if available, any outcome level assessment (see item 12).	Supp table 2/3
Results of individual studies	20	For all outcomes considered (benefits or harms), present, for each study: (a) simple summary data for each intervention group (b) effect estimates and confidence intervals, ideally with a forest plot.	Supp table 2/3
Synthesis of results	21	Present results of each meta-analysis done, including confidence intervals and measures of consistency.	NA
Risk of bias across studies	22	Present results of any assessment of risk of bias across studies (see Item 15).	NA
Additional analysis	23	Give results of additional analyses, if done (e.g., sensitivity or subgroup analyses, meta-regression [see Item 16]).	NA
**DISCUSSION**			
Summary of evidence	24	Summarize the main findings including the strength of evidence for each main outcome; consider their relevance to key groups (e.g., healthcare providers, users, and policy makers).	15
Limitations	25	Discuss limitations at study and outcome level (e.g., risk of bias), and at review-level (e.g., incomplete retrieval of identified research, reporting bias).	15-19
Conclusions	26	Provide a general interpretation of the results in the context of other evidence, and implications for future research.	15-19
**FUNDING**			
Funding	27	Describe sources of funding for the systematic review and other support (e.g., supply of data); role of funders for the systematic review.	19

From: Moher D, Liberati A, Tetzlaff J, Altman DG, The PRISMA Group (2009). Preferred Reporting Items for Systematic Reviews and Meta-Analyses: The PRISMA Statement. PLoS Med 6(7): e1000097. doi:10.1371/journal.pmed1000097

For more information, visit: www.prisma-statement.org.

For peer review only - http://bmjopen.bmj.com/site/about/guidelines.xhtml

BMJ Open

Validity and effectiveness of paediatric early warning systems and track and trigger tools for identifying and reducing clinical deterioration in hospitalised children: a systematic review

Journal:	*BMJ Open*
Manuscript ID	bmjopen-2018-022105.R1
Article Type:	Research
Date Submitted by the Author:	20-Sep-2018
Complete List of Authors:	Trubey, Rob; Cardiff University, Centre for Trials Research<br>Huang, Chao; Cardiff University, Centre for Trials Research<br>Lugg-Widger, Fiona; Cardiff University, Centre for Trials Research<br>Hood, Kerenza; Cardiff University, Centre for Trials Research<br>Allen, Davina; Cardiff University, School of Healthcare Sciences<br>Edwards, Dawn; Morriston Hospital, Department of Paediatrics<br>Lacy, David; Wirral Unviersity Teaching Hospital<br>Lloyd, Amy; Cardiff University, Centre for Trials Research<br>Mann, Mala; Cardiff University, University Library Services<br>Mason, Brendan; Public Health Wales<br>Oliver, Alison; Noah's Ark Children's Hospital for Wales, Department of Paediatric Intensive Care<br>Roland, Damian; Paediatric Emergency Medicine Leicester Academic (PEMLA) Group, Emergency Department<br>Sefton, G.; Alder Hey Childrens NHS Fdn Trust,<br>Skone, Richard; Noah's Ark Children's Hospital for Wales, Department of Paediatric Intensive Care<br>Thomas-Jones, Emma; Cardiff University, Centre for Trials Research<br>Tume, Lyvonne N.; University of the West of England Bristol, Faculty of Health and Applied Sciences (HAS)<br>Powell, Colin; Children's Hospital for Wales, Paediatrics; Sidra Medical and Research Center, Department of Pediatric Emergency Medicine
**Primary Subject Heading**:	Paediatrics
Secondary Subject Heading:	Paediatrics, Nursing, Intensive care, Health policy, Diagnostics
Keywords:	Quality in health care < HEALTH SERVICES ADMINISTRATION & MANAGEMENT, Paediatric intensive & critical care < INTENSIVE & CRITICAL CARE, PAEDIATRICS

SCHOLARONE™
Manuscripts

Validity and effectiveness of paediatric early warning systems and track and trigger tools for identifying and reducing clinical deterioration in hospitalised children: a systematic review

Authors: Rob Trubey[1]†, Chao Huang[1], Fiona Lugg-Widger[1], Kerenza Hood[1], Davina Allen[2], Dawn Edwards[3], David Lacy[4], Amy Lloyd[1], Mala Mann[5], Brendan Mason[6], Alison Oliver[7], Damian Roland[8], Gerri Sefton[9], Richard Skone[7], Emma Thomas-Jones[1], Lyvonne Tume[9], Colin Powell[10,11]

1 Centre for Trials Research, Cardiff University, Heath Park, Cardiff, UK
2 School of Healthcare Sciences, Cardiff University, Eastgate House, Cardiff, UK
3 Department of Paediatrics, Morriston Hospital, Swansea, UK
4 Wirral University Teaching Hospital, Wirral, UK
5 Specialist Unit for Review Evidence (SURE), Cardiff University, Heath Park, Cardiff, UK
6 Public Health Wales, Cardiff, UK
7 Department of Paediatric Intensive Care, Children's Hospital for Wales, Cardiff, UK
8 Paediatric Emergency Medicine Leicester Academic (PEMLA) Group, Emergency Department, Infirmary Square, Leicester, UK
9 Paediatric Intensive Care Unit, Alder Hey Children's NHS Foundation Trust, Liverpool, UK
10 Department of Child Health, Children's Hospital for Wales, Division of Population Medicine School of Medicine, Cardiff University, Cardiff, UK,
11 Department of Pediatric Emergency Medicine, Sidra Medicine, Doha, Qatar

Corresponding author:
Dr. Rob Trubey, Centre for Trials Research, Cardiff University, Heath Park, Cardiff, UK
trubeyrj@cf.ac.uk

Keywords: PEWS, track and trigger scores, early warning scores, clinical deterioration, children, systematic review

For peer review only - http://bmjopen.bmj.com/site/about/guidelines.xhtml

ABSTRACT

Objectives

To assess (1) how well validated existing paediatric track and trigger tools (PTTT) are for predicting in-patient deterioration, and (2) how effective paediatric early warning systems (with or without a PTTT) are at reducing mortality and morbidity outcomes in hospitalised children.

Methods

A systematic search was carried out through May 2018, across several databases. Supplementary searches were carried out to identify published and unpublished literature. Studies reporting on the development and validation of PTTT and effectiveness of broader early warning systems were eligible for inclusion. Study selection, data extraction and quality assessment were conducted by two independent reviewers and disagreements resolved by discussion. PROSPERO registration number CRD42015015326.

Results

36 validation studies and 30 effectiveness studies were included, with 27 unique PTTT identified. Outcome measures varied considerably for both research questions. Validation studies were largely retrospective, case-control studies or chart reviews: some PTTT demonstrated very good or excellent diagnostic accuracy across multiple studies (primarily for predicting PICU transfers) but PPV values were consistently low, suggesting the potential for alarm fatigue. No studies accounted for the co-occurrence of routine clinical intervention when assessing the relationship between PTTT scores and subsequent deterioration. Effectiveness studies were predominantly uncontrolled before-after studies. Overall, there was limited evidence of paediatric early warning system interventions leading to reduced rates of deterioration. Some studies reported significant decreases in mortality, arrests or code calls, but were limited by methodological concerns.

Conclusion

There are a number of fundamental methodological limitations in the PTTT literature, and a predominance of single-site studies carried out in specialist centres limits generalisability. With limited evidence of effectiveness, we would argue that calls to make PTTT mandatory

For peer review only

across all paediatric units are not supported by the evidence base. More work is needed to understand the impact of PTTT implementation on the wider clinical microsystem.

For peer review only - http://bmjopen.bmj.com/site/about/guidelines.xhtml

STRENGTHS AND LIMITATIONS OF THIS STUDY

- Paediatric early warning systems and paediatric track and trigger tools (PTTT) are increasingly used by paediatric units across Europe, North America, Australia and elsewhere – this study is a timely review of the evidence for their validity and effectiveness
- A comprehensive search was carried out across multiple databases and included published as well as grey literature
- The review highlights methodological weaknesses and gaps in the current evidence base and makes suggestions for future research
- Heterogeneity in study populations, study designs and outcome measures make it difficult to compare and synthesise findings across the wide range of early warning systems and PTTT being used in practice
- The review is limited in scope to quantitative validation and effectiveness studies, so must be considered alongside wider literature reflecting on potential secondary benefits of early warning systems and PTTT for communication, teamwork and empowerment

For peer review only

For peer review only - http://bmjopen.bmj.com/site/about/guidelines.xhtml

BACKGROUND

Failure to recognise and respond to clinical deterioration in hospitalised children is a major safety concern in healthcare. The underlying causes of this problem are clearly multi-factorial[1–3] but paediatric 'early warning systems' have been strongly advocated as one approach to improving recognition of deterioration in paediatric units[1,2,4].

A paediatric 'early warning system' can be considered any patient safety initiative or programme which aims to monitor, detect and respond to signs of deterioration in hospitalised children in order to avert adverse outcomes and premature death. Such systems are often multi-faceted and may include the use of rapid response teams (RRT) or medical emergency teams (MET), education or training to improve clinical staff's ability to identify deterioration or strategies aimed at improving staff communication and situational awareness.

An increasingly commonplace paediatric 'early warning system' initiative is the use of a 'track and trigger tool': these tools, also commonly used in adult care, provide a formal framework for evaluating routine physiological, clinical and observational data for early indicators of patient deterioration. They are typically integrated into routine observation charts or electronic health records and compare patient observations to pre-defined 'normal' thresholds. When one or more observation is considered abnormal, staff are directed to various clinical actions, including but not limited to altered frequency of observations, review by senior staff or more appropriate treatment or management. Tools may be paper based or electronic and monitoring may be automated or manually undertaken by staff.

These tools have been referred to in the literature using a number of different terms: paediatric early warning scores (PEWS); paediatric early warning tools (PEWT), track and trigger tools (TTT) and many others. Here, we refer to the tools themselves using the term 'paediatric track and trigger tools' (PTTT). A variety of PTTT have been developed, typically by teams based in specialist paediatric centres and often used as a means of triggering a dedicated response team. Their advocacy has recently led to widespread uptake across a variety of different paediatric units, including many non-specialist centres where patient populations and resources may differ. In the United Kingdom (UK), a recent cross-sectional survey found that 85% of paediatric units were using some form of PTTT, most of which were non-specialist centres without a dedicated response team[5]. Despite their widespread use, recent reviews have questioned the evidence-base for their effectiveness in improving patient outcomes [6,7]. The current review aimed to build on this work, assessing in depth the evidence

For peer review only - http://bmjopen.bmj.com/site/about/guidelines.xhtml

base for both the validity of PTTT for predicting in-patient deterioration and the effectiveness of broader ‘early warning systems’ at reducing instances of mortality and morbidity in paediatric settings:

- Question 1: How well validated are existing paediatric track and trigger tools (PTTT) and their component parts for predicting in-patient deterioration?
- Question 2: How effective are paediatric early warning systems (with or without a PTTT) at reducing mortality and critical events?

For peer review only

For peer review only - http://bmjopen.bmj.com/site/about/guidelines.xhtml

METHODS

This systematic review was conducted and reported in accordance with the Preferred Reporting Items for Systematic Review and Meta-Analyses (PRISMA) guidelines[8]. Our review protocol is registered with the PROSPERO database CRD42015015326.

Search strategy

A comprehensive search was conducted across a range of databases to identify relevant studies in the English language. Published and unpublished literature was considered where publicly available, as were studies in press. The following databases were searched through May 2018: British Nursing Index, CINAHL (Cumulative Index of Nursing and Allied Health Literature), Cochrane Central Register of Controlled Trials, Database of Abstracts of Reviews of Effectiveness, EMBASE, HMIC (Health Management Information Centre), Medline, Medline in Process, Scopus and Web of Knowledge (Science Citation Indexes). To identify additional papers, published, unpublished or research reported in the grey literature a range of relevant websites and trial registers were searched including Clinical Trials.gov. To identify published papers that had not yet been catalogued in the electronic databases, recent editions of key journals were hand-searched. The search terms included ‘early warning scores’, ‘alert criteria’, ‘rapid response’, ‘track and trigger’ and ‘early medical intervention’. (Supplementary Table 1)

Eligibility screening and study selection

PICOS parameters guided inclusion criteria for the validation and effectiveness studies (Supplementary Table 2). Papers reporting development of validation of a PTTT were included for Question 1, whereas papers reporting the implementation of any broader ‘paediatric early warning system’ (with or without a PTTT) were eligible for Question 2. Both research questions were limited to studies that involved in-patients aged 0-18. Outcome measures considered were mortality and critical events, including: unplanned admission to a higher level of care, cardiac arrest, respiratory arrest, medical emergencies requiring immediate assistance, children reviewed by Paediatric Intensive Care Unit (PICU) staff on the ward (in specialist centres) or reviewed by external PICU staff (for non-specialist centres), acuity at PICU admission and PICU outcomes. A range of study designs were considered for both questions.

For peer review only - http://bmjopen.bmj.com/site/about/guidelines.xhtml

Two of the review authors independently screened the titles and abstracts yielded in the search. Full texts were reviewed independently by six reviewers against the above eligibility criteria and were assigned to the relevant review question if included. Reasons for exclusion were recorded. Separate data extraction forms were developed for validation and effectiveness studies. The forms had common elements (study design, country, setting, study population, description of the PTTT or early warning system, statistical techniques used, outcomes assessed). Additional data items for validation studies included the items in the PTTT, modifications to the PTTT from previous versions, predictive ability of individual items and the overall tool, sensitivity and specificity and inter and intra-rater reliability. Effectiveness studies included an assessment of outcomes in terms of mortality and various morbidity variables. Data extraction was carried out by two reviewers and discrepancies were resolved by discussion. Risk ratios (RR) and P-values were reported or calculated for all effectiveness outcomes, where available.

Quality appraisal

Methodological quality and risk of bias was assessed for each included study using a modified version of the Downs and Black rating scale[9] (templates shown in Supplementary Table 3).

Patient and Public Involvement

This review was conducted as part of a larger mixed-methods study (ISRCTN94228292), which used a formal, facilitated parental advisory group. The group comprised parents of children who had experienced an unexpected adverse event in a paediatric unit and provided input which helped to shape the broader research questions and outcome measures. The results of the review will be disseminated to parents through this group.

For peer review only - http://bmjopen.bmj.com/site/about/guidelines.xhtml

REVIEW RESULTS

Figure 1 shows the PRISMA flow diagram for both research questions.

[FIGURE 1]

Study characteristics

Table 1 summarises the study characteristics of the 36 validation (Question 1) and 30 effectiveness (Question 2) papers included in the review.

Validation studies (n=36)			Effectiveness studies (n=30)		
	N	%		n	%
**Type**			**Type**		
Full text	22	61.1	Full text	21	70.0
Abstract	14	38.9	Abstract	9	30.0
**Country**			**Country**		
United States	15	41.7	United States	18	60.0
United Kingdom	12	33.3	United Kingdom	3	10.0
Canada	2	5.5	Canada	2	6.7
Australia	0	0.0	Australia	3	10.0
Other	5	13.9	Other	3	10.0
Multiple	1	2.8	Multiple	1	3.3
Unclear	1	2.8	Unclear	0	0.0
**Year of study**			**Year of study**		
Pre-2012	10	27.8	Pre-2012	15	50.0
2012	3	8.3	2012	1	3.3
2013	6	16.7	2013	2	6.7
2014	5	13.9	2014	6	20.0
2015	7	19.4	2015	0	0.0
2016	2	5.6	2016	2	6.7
2017	3	8.3	2017	1	3.3
2018	0	0.0	2018	3	10.0
**Setting**			**Setting**		
Specialist / tertiary	33	91.7	Specialist / tertiary	29	96.7
Non-specialist / community	0	0.0	Non-specialist / community	1	3.3
Unclear	3	8.3	Unclear	0	0.0
**Single / multi-centre**			**Single / multi-centre**		
Single-centre	35	97.2	Single-centre	28	93.3
Multi-centre	1	2.8	Multi-centre	2	6.7
**Study population**			**Study population**		
General in-patients	23	63.9	General in-patients	20	66.6
Specialist population	11	30.6	Specialist population	5	16.7
Unclear	2	5.6	Unclear	5	16.7
**Study design**			**Study design**		
Cohort	7	19.4	Uncontrolled before-after	26	86.7

For peer review only - http://bmjopen.bmj.com/site/about/guidelines.xhtml

Case-control	18	50.0	Controlled before-after	1	3.3
Case / chart review	10	27.8	Interrupted Time Series	2	6.7
Pilot study	1	2.8	Cluster randomised trial	1	3.3

Table 1: Summary study characteristics of validation and effectiveness papers in the review

Types of PTTS and components

Across 66 studies, we identified 27 unique PTTT (Table 2). Twenty PTTTs were based on one of four different tools: Monaghan's Brighton PEWS[10], the Bedside PEWS[11], the Bristol PEWT[12] and the Melbourne Activation Criteria[13]. Other PTTT described in the literature included the National Health Service Institute for Innovation and Improvement (NHS III) PEWS[14] (the second most commonly used PTTT in United Kingdom paediatric settings[5]), RRT and MET activation criteria[15–18], and one prediction algorithm developed from a large dataset of electronic health data[19].

[TABLE 2]

Table 2 illustrates the range of physiological and behavioural parameters underpinning PTTT. Common parameters included heart rate (present in 26 out of 27 PTTT), respiratory rate (24), respiratory effort (24) and level of consciousness or behavioural state (24). All PTTT required at least six different parameters to be collected.

Question 1 – How well validated are PTTT and component parts for predicting in-patient deterioration?

Nine validation papers meeting inclusion criteria were excluded from analysis: eight did not report any performance characteristics of the PTTT for predicting deterioration[20–27] and one study calculated incorrect sensitivity/specificity outcomes[12] (Supplementary Table 4). The remaining 27 validation studies, evaluating the performance of 18 unique PTTT, are described in Table 3. Four studies evaluated multiple PTTTs[3,19,28,29] and one paper described three separate studies of the same PTTT[30].

[TABLE 3]

Five cohort studies were included[14,31–34], three based on the same dataset. All other studies were either case-control or chart reviews. Thirteen papers implemented the PTTT in practice[23,30,31,34–43], while the remaining studies 'bench tested' the PTTT – researchers retrospectively calculated the score based on data abstracted from medical charts and records. All studies were conducted in specialist centres with only one multi-centre study reported[44].

Outcome measures

For peer review only - http://bmjopen.bmj.com/site/about/guidelines.xhtml

For peer review only

PTTT were evaluated for their ability to predict a wide range of clinical outcomes. Composite measures were used in eight studies[14,23,29,32,33,37,45,46], cardiac/respiratory arrest or a "code call" was used (singularly or part of a composite outcome) in six studies[23,28,29,37,45,47], while 22 studies used transfer to a to PICU or PHDU as the main outcome[3,11,19,23,28–34,36,37,39,41–44,46,48,49].

Predictive ability of individual PTTT components

Three validation papers reported on the performance characteristics of individual components of the tool for predicting adverse outcomes[11,33,42]. Parshuram and colleagues, for instance, reported AUROC values for individual PTTT items of a pilot version of the Bedside PEWS: ranging from 0.54 (bolus fluid) to 0.81 (heart rate), compared to 0.91 for the overall PTTT[11]. All other studies reported outcomes for the PTTT as a whole.

Paediatric Early Warning System (PEWS) score

The predictive ability of the 16-item PEWS score was assessed by one internal[47] (AUROC=0.90) and two external case-control studies[28,29] (AUROC range =0.82-0.88) with a range of outcome measures and scoring thresholds. One case-control study used an observed prevalence rate to calculate a PPV of 4.2% for the tool in predicting code calls[47] (for every 1,000 patients triggering the PTTT, 42 would be expected to deteriorate).

Bedside PEWS and derivatives

The Bedside PEWS was evaluated in one internal[11] (AUROC=0.91) and five external case-control studies[19,28,29,44,46] (AUROC range=0.73-0.90) for a range of different outcome measures and at different scoring thresholds. One case-control study calculated a PPV of 2.1% for identifying children requiring urgent PICU transfer within 24 hours of admission, based on locally observed prevalence rates[19]. A modified version of the Bedside PEWS (with temperature added) demonstrated an AUROC of 0.86 in an external case-control study with a composite outcome of death, arrest or unplanned PICU transfer[29].

Brighton PEWS and derivatives

Six different PTTT based on the original Brighton PEWS were evaluated across 11 studies. Tucker and colleagues reported a large prospective cohort study of their Modified Brighton PEWS (a) to test prediction of PICU transfers (AUROC=0.92, PPV=5.8%)[31]. An external case-control study tested the same score for predicting urgent PICU transfers within 24 hours of admission (AUROC=0.74, PPV= 2.1%)[19].

For peer review only - http://bmjopen.bmj.com/site/about/guidelines.xhtml

An external case-control study used a composite measure of death, arrest or PICU transfer to evaluate Akre *et al.*'s Modified Brighton PEWS (b) (AUROC=0.79) and Skaltezky's Modified Brighton PEWS (d) (AUROC=0.74)[29]. Skaletzky and colleagues also evaluated their own tool in an internal case-control study for predicting PICU transfer (AUROC=0.82) [48].

The Children's Hospital Early Warning Score (CHEWS) had a reported AUROC of 0.90 for predicting PICU transfers or arrests in a large internal case-control study[50]. A version of the same tool modified for cardiac patients, the Cardiac CHEWS (C-CHEWS) was evaluated by one internal study on a cardiac unit[37] (AUROC = 0.90) looking at arrests or unplanned PICU transfers, and two external studies of oncology / haematology units[41,42] for the same outcome (AUROC=0.95). Finally, the Children's Hospital Los Angeles (CHLA) PEWS was evaluated by in a small internal case-control study for prediction of re-admission to PICU after initial PICU discharge[40] (AUROC=0.71).

Melbourne Activation Criteria (MAC) and derivatives

The MAC was assessed by one external case-control study with an outcome of death, arrest or unplanned PICU transfer[29] (AUROC=0.71) and a large external cohort study with an outcome of death or unplanned PICU or HDU transfer[33] (AUROC=0.79, PPV=3.6%). A derivative of the MAC using an aggregate score, the Cardiff & Vale PEWS (C&VPEWS), was tested using the same cohort and outcome measures in an earlier external study (AUROC=0.86, PPV=5.9%)[32] and was the best performing PTTT in an external case-control study evaluating multiple PTTT[29] (AUROC=0.89).

Bristol PEWT

The Bristol PEWT was evaluated by five external validation studies: two chart review studies[3,35] (no AUROC), one small cohort study of PICU transfers[34] (AUROC=0.91, PPV=11%), and two case-control studies looking at code calls[28] (AUROC=0.75) and a composite of death, arrests and PICU transfers[29] (AUROC=0.62).

Other PTTT

The NHS Institute for Improvement and Innovation (NHS III) PEWS was tested by one external cohort study looking at a composite of death or unplanned transfers to PICU or HDU[14] (AUROC=0.88, PPV=4.3%) and one external case-control study looking at a composite of death, arrests and PICU transfers[29] (AUROC=0.82). Zhai and colleagues developed and retrospectively evaluated a logistic regression algorithm in an internal case-

For peer review only - http://bmjopen.bmj.com/site/about/guidelines.xhtml

control study looking at urgent PICU transfers in the first 24 hours of admission[19] (AUROC =0.91, PPV=4.8%).

Across PTTT, studies reporting performance characteristics of a tool at a range of different scoring thresholds demonstrate the expected interaction and trade-off between sensitivity and specificity – at lower triggering thresholds, sensitivity is high but specificity is low; at higher thresholds, the opposite is true.

Inter-rater reliability and completeness of data

Accurate assessment of the ability of a PTTT to predict clinical deterioration is contingent on accuracy and reliability of tool scoring (whether by bedside nurses in practice or by researchers abstracting data) and the availability of underpinning observations. Only five papers made reference to accuracy or reliability of scoring[28,31,37,42,45], with mixed results: Tucker *et al.* implemented their PTTT and reported that two nurses scored 55 patients on their modification of the Brighton PEWS[31], achieving an intra-class coefficient of 0.92, but McLellan *et al*., reported only 67% agreement in scoring on their C-CHEWS tool between a study nurse and the bedside nurse[37]. Completeness of data was reported in 11 studies[11,14,19,29,30,32,33,42,44,45,47]. Fuijkschot *et al.*[30] reported that in 59% of cases reviewed "the PEWS was correctly performed and could be used for inclusion in the study", Edwards *et al.*[33] bench-tested the C&VPEWS and found an average completeness rate of 44% for the seven different parameters, while Parshuram *et al.*[44] reported in a multi-centre study of the Bedside PEWS that "only 5.1% [of observation sets] had measurements on all 7 items".

Question 2 – how effective are early warning systems at reducing mortality and critical events in hospitalised children?

Eleven papers meeting inclusion criteria were excluded from analysis for providing insufficient statistical information (e.g., denominator data, absolute numbers of events) to calculate effect sizes[39,51–59]. Further details on papers excluded from analysis are provided in Supplementary Table 5. Findings from the 19 studies included in the analysis are summarised in Table 4.

[TABLE 4]

Type of early warning system interventions

Seventeen interventions involved the introduction of a new PTTT, one intervention introduced a mandatory triggering element to an existing PTTT[60], and one study reported a

For peer review only - http://bmjopen.bmj.com/site/about/guidelines.xhtml

large, multi-centre analysis of MET introduction with no details on PTTT use[61]. Twelve interventions included the introduction of a new MET or RRT[13,15–18,62–68], while four further interventions introduced a new PTTT in a hospital with an existing MET or RRT. Only three studies therefore evaluated a PTTT in the absence of a dedicated response team[69–71]. A staff education programme was explicitly described in ten nterventions[13,15,17,63,65,67,69–72].

Of the 18 studies that used a PTTT, only seven used a tool that had been formally evaluated for validity: three used the Bedside PEWS[62,65,70], two used the MAC[13,67], one used the Modified Brighton PEWS (b)[72] and one used the C-CHEWS[71]. One study did not report the PTTT used[63], while ten studies used a variety of calling criteria and local modifications to validated tools that had not been evaluated for validity[15–18,60,64,66,68,69,73].

Mortality (ward or hospital wide)

Two uncontrolled before-after studies (both with MET/RRT) reported significant mortality rate reductions post intervention: one in hospital wide deaths per 100 discharges[17] (RR=0.82, 95% CI=0.70-0.95) and one in total hospital deaths per 1,000 admissions (RR=0.65, 0.57-0.75) and deaths on the ward ('unexpected deaths') per 1,000 admissions[67] (RR=0.35, 0.13-0.92). Seven studies found no reductions in mortality, including two high quality multi-centre studies[13,15,61,62,64,65,68]. Parshuram and colleagues conducted a cluster randomised trial and found no difference in all-cause hospital mortality rates between 10 hospitals randomly selected to receive an intervention centred around use of the Bedside PEWS and 11 usual care hospitals, one year post intervention (OR=1.01, 0.61-1.69)[65]. Kutty *et al.* assessed the impact of MET implementation in 38 US paediatric hospitals with an interrupted time series study, and reported no difference in the slope of hospital mortality rates five years post intervention and the expected slope based on pre-implementation trends (OR = 0.94, 0.93-0.95)[61].

PICU mortality

Two uncontrolled before-after studies (both with MET/RRT) reported a significant post-intervention reduction in rates of PICU mortality among ward transfers (RR=0.31, 0.13-0.72)[18], and PICU mortality rates among patients readmitted within 48 hours (RR=0.43, 0.17-0.99)[64]. Six studies (including a high quality cluster randomised trial and interrupted time series study) reported no post-intervention change in PICU mortality using a variety of metrics[62,65,66,69,71,73].

Cardiac and respiratory arrests

For peer review only - http://bmjopen.bmj.com/site/about/guidelines.xhtml

Two uncontrolled before-after studies (both with RRT/MET) reported significant post-intervention rate reductions in sub-categories of cardiac arrests: one in ‘near cardiopulmonary arrests’[64] (RR=0.54, 0.52-0.57) but not ‘actual cardiopulmonary arrests’ and one in ‘preventable cardiac arrests’[67] (RR=0.45, 0.20-0.97) but not ‘unexpected cardiac arrests’. One uncontrolled before-after study (with RRT/MET) reported a significant post intervention reduction in rates of ward respiratory arrests per 1,000 patient-days[16] (RR=0.27, 0.05-1.01). Seven studies (including one high quality cluster randomised trial and one high quality interrupted time series study) found no change in cardiac arrest rates using a variety of metrics[13,15,16,62,63,65] or cardiac and respiratory arrests combined[68].

Calls for urgent review / assistance

Three uncontrolled before-after studies (all with RRT/MET) reported significant post-intervention reductions in rates of code calls[15,17,64] (RR=0.42, 0.17-1.03; RR=0.29, 0.10-0.65; RR=0.71, 0.61-0.83) while two studies found no change in rates of code calls[18,72]. One uncontrolled before-after study in a community hospital (without RRT/MET) found significant post intervention reductions in rates of urgent calls to the in-house paediatrician (RR=0.23, 0.11-0.46) and respiratory therapist[70] (RR=0.36, 0.13-0.95). Two uncontrolled before-after studies (with RRT/MET) found increases in rates of RRT calls[72] (RR=1.59, 1.33-1.90) and outreach team calls[73] (RR=1.92, 1.79-2.07). One study found no change in rates of RRT calls[60].

PICU transfers

One uncontrolled before-after study (without RRT/MET) found a significant post-intervention decrease in the rate of unplanned PICU transfers per 1,000 patient-days[71] (RR=0.70, 0.56-0.88). One uncontrolled before-after study (without RRT/MET) found a significant post-intervention increase in the rate of transfers to external specialist units (RR=1.37, 0.51-3.63), but with a significant decrease in the rate of ‘clinical deterioration events’ on the ward (RR=0.18, 0.02-1.97)[70]. Three studies (including one high quality cluster randomised trial and one high quality interrupted time series study) found no change in rates of PICU admissions post intervention[62,65,73].

PICU outcomes

Two studies, one interrupted time series and one multi-centre cluster randomised trial (both with RRT/MET), found significant reductions in rates of ‘critical deterioration events’ (life-sustaining interventions administered within 12 hours of PICU admission) relative to pre-

For peer review only - http://bmjopen.bmj.com/site/about/guidelines.xhtml

implementation trends and relative to control hospitals respectively (IRR=0.38, 0.20-0.75; OR=0.77, 0.61-0.97)[62,65]. One controlled before-after study (without RRT/MET) reported a significant reduction in rates of invasive ventilation given to emergency PICU admissions post intervention (RR=0.83, 0.72-0.97) with no significant change observed in a control group of patients admitted to PICU from outside of the hospital[69]. One uncontrolled before-after study reported a significant post-intervention decrease in rates of PICU admissions receiving mechanical ventilation (RR=0.85, 0.73-0.99) but an increase in rates of early intubation (RR=1.87, 1.33-2.62)[66].

Implementation outcomes

Only three studies reported outcomes relating to the quality of implementation of the intervention. Parshuram and colleagues reported 99% of audited observation sets of the Bedside PEWS had at least 5 vital signs present post-intervention, up from 76% pre-intervention (no change in control hospitals)[65]. A previous Bedside PEWS study reported 3% of audited cases had used the incorrect age chart but reported an intra-class coefficient of 0.90 for agreement between bedside nurses scoring the PTTT in practice and research nurses retrospectively assigned scores[70]. Finally, Agulnik and colleagues evaluated error rates in C-CHEWS scoring, observing an initial 47% rate of errors that reduced to below 10% by the end of the study[71].

DISCUSSION

This paper reviewed the published PTTT and early warning system literature in order to assess the validity of PTTT for predicting in-patient deterioration (Question 1) and the effectiveness of early warning system interventions (with or without PTTT) for reducing mortality and morbidity outcomes in hospitalised children (Question 2). We believe that the consideration of broader 'early warning systems' differentiates this paper from previous reviews, as does the inclusion of two recently published high-quality effectiveness studies[61,65].

How well validated are existing tools for predicting in-patient deterioration?

Given a growing understanding and emphasis on the importance of local context in healthcare interventions, it is perhaps not surprising that such a wide range of PTTT have been developed and evaluated internationally, and modifications to existing PTTT are common. The result, however, is that a large number of different PTTT have been narrowly validated, but none have been broadly validated across a variety of different settings and

For peer review only - http://bmjopen.bmj.com/site/about/guidelines.xhtml

populations. With only one exception[44], all studies evaluating the validity of PTTT have been single-centre reports from specialist units, greatly limiting the generalisability of the findings.

PTTT such as the Bedside PEWS, C&VPEWS, NHS III PEWS and C-CHEWS have demonstrated very good (AUROC ≥0.80) or excellent (AUROC ≥ 0.90) diagnostic accuracy, typically for predicting PICU transfers, in internal and external validation studies[11,14,19,32,37,42,44,74]. However, methodological issues common to the validation studies mean that such results need to be interpreted with a degree of caution. Firstly, each of the studies was conducted in a clinical setting where paediatric in-patients are subject to various forms of routine clinical intervention throughout their admission. This automatically complicates the relationship between patients' PTTT scores at different time points and the subsequent occurrence (or not) of deterioration – across the study window, patients will have received a variety of different clinical interventions which will have impacted their chances of deteriorating. Indeed, the majority of outcomes used in the validation studies are clinical interventions themselves (e.g., PICU transfer). There are numerous statistical modelling techniques which can account for co-occurrence of clinical interventions and the longitudinal nature of the predictors[75,76], but none of these were used in the validation studies and so estimates of predictive ability are likely to be distorted. Secondly, while it understandable that a majority of studies 'bench-tested' the PTTT rather than implement it into practice before evaluation, the process of abstracting PTTT scores retrospectively from patient charts and medical records introduces a number of sources of potential bias or inaccuracy. For instance, several studies reported either high levels of missing data (i.e., some of the observations required to populate the PTTT score being evaluated were not routinely collected or recorded and so were scored as 'normal')[11,19,32,44,45] or difficulty in abstracting certain descriptive or subjective PTTT components[19,28,41,49]. Assuming missing values are normal, or excluding some PTTT items for analysis are both likely to skew the results. Finally, studies which evaluated a PTTT that had been implemented in practice are at risk of overestimating the ability of PTTT to predict proxy outcomes such as PICU transfer, inasmuch as high PTTT scores or triggers automatically direct staff towards escalation of care, or clinical actions which make escalation of care more likely.

The findings reported in several PTTT studies point towards two potential challenges for some centres in implementing and sustaining a PTTT in clinical practice. As noted above, a number of studies that retrospectively 'bench-tested' a PTTT reported that the observations that were required to score the tool were not always routinely collected or recorded in their

For peer review only - http://bmjopen.bmj.com/site/about/guidelines.xhtml

centre. It may be that the introduction of a PTTT into practice would help create a framework to ensure that core vital signs and observations were collected more routinely (as demonstrated by Parshuram and colleagues[65]) but this would obviously have resource implications that could be a potential barrier for some centres. Such considerations are important, as evidence from the adult literature points to the potential for tools to inadvertently mask deterioration when core observations are missing[77]. Secondly, PPV values reported in cohort studies, and case-control studies that adjusted for outcome prevalence, were uniformly low (between 2.3%-5.9%)[14,19,31–33,47]. They demonstrate that even PTTT which demonstrate good predictive performance are likely to generate a large amount of 'false alarms' because adverse outcomes are so rare. For some centres, these issues may be mitigated to some extent by dedicated response teams or other available resources, but other hospitals may not be able to sustain the increased workload of responding to PTTT triggers.

How effective are early warning systems for reducing mortality and morbidity?

We found limited evidence for early warning system interventions reducing mortality or arrest rates in hospitalised children. While some effectiveness papers did report significant reductions in rates of mortality (on the ward or in PICU) or cardiac arrests after implementation of different early warning system interventions[16–18,64,67], they were all uncontrolled before-after studies which have inherent limitations in terms of establishing causality. They do not preclude the possibility that outcome rates would have improved over time regardless of the intervention[78] or changes were caused by other factors, and their inclusion is accordingly discouraged by some Cochrane review groups[79]. Three high quality multi-centre studies - two interrupted time series studies and a recent cluster randomised trial – found no changes in rates or trends of mortality or arrests post intervention[61,62,65].

There was also limited evidence for early warning systems reducing PICU transfers or calls for urgent review. Again, a small number of uncontrolled before-after studies reported significant reductions post-intervention[15,17,64], but several other studies reported significant increases in transfers or calls for review[70,72,73] or no post-intervention changes. We did find moderate evidence across four studies – including a controlled before-after study, a multi-centre interrupted time series study and a multi-centre cluster randomised trial - for early warning system interventions reducing rates of early critical interventions in children transferred to PICU[62,65,66,69]. Such results are promising, but corresponding reductions in hospital or PICU mortality rates have not yet been reported.

For peer review only - http://bmjopen.bmj.com/site/about/guidelines.xhtml

Implementing complex interventions in a healthcare setting is challenging and evidence from the adult literature points to challenges and barriers to successfully implement TTT in practice[80–82]. However, given so few effectiveness studies reported on implementation outcomes, it is difficult to know whether negative findings reflect poor effectiveness or implementation of early warning systems. Again, effectiveness studies were predominantly carried out in specialist centres – and in all but three cases, involved the use of a dedicated response team – greatly limiting generalisability outside of these contexts.

Limitations of the review

There are sevral limitations of the current review. Firstly, despite purposely widening the scope of the effectiveness review question to include paediatric 'early warning systems' with or without a PTTT, we identified very few studies that did not employ a PTTT as part of the intervention . In part, this likely reflects the fact that PTTT have become almost synonymous with early warning systems, but it is also possible that our search strategy may have missed some broader early warning system initiatives that were not explicitly labelled as such. Secondly, our inclusion criteria for study selection were deliberately broad and so resulted in our including several validation and effectiveness studies that were subsequently excluded from analysis due to insufficient statistical detail or methodological issues. Finally, the scope of the current review was limited to consideration of quantitative validation and effectiveness studies. We are mindful of research suggesting that implementing PTTT in practice may confer secondary benefits including, but not limited to improvements in communication, teamwork and empowerment of junior staff to call for assistance[83–85].

Conclusion

The PTTT literature is currently characterised by an 'absence of evidence' rather than an 'evidence of absence'. PTTT seem like a logical tool for helping staff detect and respond to deteriorating patients, but the existing evidence base is too limited to form clear judgements of their utility. We would argue that there has been too much confidence placed in the statistical findings of validation studies of PTTT, given methodological limitations in the study designs. There is evidence of consistently high false-alarm rates and bench-testing studies point to unreliable availability of many underlying observations: as such there is reason for caution in considering the viability of PTTT for all hospitals. Almost all of the early warning systems and PTTT reported in the literature have been developed and evaluated in specialist centres, typically in units with access to dedicated response teams –

For peer review only - http://bmjopen.bmj.com/site/about/guidelines.xhtml

yet PTTT appear to be commonly adopted by non-specialist units with little modification. With limited current evidence for 'early warning systems' that use a PTTT reducing deterioration or death in practice, we would urge caution among policymakers in calling for their use to become mandatory across all hospitals. We acknowledge the potential for PTTT to confer a range of secondary benefits in areas such as communication, teamwork and empowerment of junior staff. Indeed, we would argue that more work is required to understand the wider impact of PTTT implementation in different clinical settings before it is possible to evaluate their overall contribution to the wider safety mechanisms and systems aimed at identifying and responding to deteriorating in paediatric patients.

For peer review only

FOOTNOTES

Contributors

RT: screening and review of papers, contribution to design of work, preparation of manuscript; CH: screening and review of papers, contribution to concept and design of work, review of manuscript; FL: contribution to design of work, screening and review of papers, review of manuscript; KH: contribution to concept and design of work, screening and review of papers, review of manuscript; CP, DR, BM, AO, DE, RS, GS, DL, LT, DA, AL, ETJ: contribution to concept and design of work, screening and review of papers, review of manuscript; MM: information specialist, review of manuscript.

Acknowledgements

We would like to acknowledge the contribution of Dr James Bunn to the review.

Competing interests

None declared.

Funding

This study is funded by the National Institute for Health Research (NIHR) Health Services and Delivery Research (HS&DR) programme (12/178/17).

Data sharing statement

No additional data are available.

For peer review only - http://bmjopen.bmj.com/site/about/guidelines.xhtml

FIGURE LEGENDS

Figure 1 – PRISMA flow diagram of study inclusion

For peer review only

For peer review only - http://bmjopen.bmj.com/site/about/guidelines.xhtml

REFERENCES

1. Pearson, G. a (Ed). *Why Children Die: A Pilot Study 2006; England (South West, North East and West Midlands), Wales and Northern Ireland.* (2008). doi:ISBN: 978-0-9558055-0-9

2. Joint Commission. 2009 National Patient Safety Goals, Requirements and Rationales. *Jt. Comm. Perspect. Patient Saf.* **8,** 8–12 (2009).

3. Tume, L. The deterioration of children in ward areas in a specialist children's hospital. *Nurs. Crit. Care* **12,** 12–19 (2007).

4. NICE. Acutely ill patients in hospital: Recognition of and response to acute illness in adults in hospital. *NICE Clin. Guidel. 50* 1–107 (2007). doi:10.1136/bmj.39272.679688.47

5. Roland, D., Oliver, A., Edwards, E. D., Mason, B. W. & Powell, C. V. E. Use of paediatric early warning systems in Great Britain: has there been a change of practice in the last 7 years? *Arch. Dis. Child.* **99,** 26–9 (2014).

6. Chapman, S. M., Wray, J., Oulton, K. & Peters, M. J. Systematic review of paediatric track and trigger systems for hospitalised children. *Resuscitation* **109,** 87–109 (2016).

7. Lambert, V., Matthews, A., MacDonell, R. & Fitzsimons, J. Paediatric early warning systems for detecting and responding to clinical deterioration in children: a systematic review. *BMJ Open* **7,** e014497 (2017).

8. Moher, D., Liberati, A., Tetzlaff, J. & Altman, D. G. Preferred reporting items for systematic reviews and meta-analyses: the PRISMA statement. *BMJ* **339,** (2009).

9. Downs, S. H. & Black, N. The feasibility of creating a checklist for the assessment of the methodological quality both of randomised and non-randomised studies of health care interventions. *J. Epidemiol. Community Health* **52,** 377–384 (1998).

10. Monaghan, A. Detecting and managing deterioration in children. *Paediatr. Nurs.* **17,** 32–35 (2005).

11. Parshuram, C. S., Hutchison, J. & Middaugh, K. Development and initial validation of the Bedside Paediatric Early Warning System score. *Crit. Care* **13,** R135–R135 (2009).

12. Haines, C., Perrott, M. & Weir, P. Promoting care for acutely ill children - Development and evaluation of a Paediatric Early Warning Tool. *Intensive Crit. Care Nurs.* **22,** 73–81 (2006).

13. Tibballs, J., Kinney, S., Duke, T., Oakley, E. & Hennessy, M. Reduction of paediatric in-patient cardiac arrest and death with a medical emergency team: preliminary results. *Arch. Dis. Child.* **90,** 1148–1152 (2005).

14. Mason, B. W., Edwards, E. D., Oliver, A. & Powell, C. V. E. Cohort study to test the predictability of the NHS Institute for Innovation and Improvement Paediatric Early Warning System. *Arch. Dis. Child.* archdischild–2015–308465 (2016). doi:10.1136/archdischild-2015-308465

15. Brilli, R. J. *et al.* Implementation of a medical emergency team in a large pediatric teaching hospital prevents respiratory and cardiopulmonary arrests outside the intensive care unit. *Pediatric Critical Care Medicine* **8,** 236–246 (2007).

16. Hunt, E. A. *et al.* Transition from a traditional code team to a medical emergency team

For peer review only - http://bmjopen.bmj.com/site/about/guidelines.xhtml

and categorization of cardiopulmonary arrests in a children's center. *Arch. Pediatr. Adolesc. Med.* **162,** 117–122 (2008).

17. Sharek, P. J. *et al.* Effect of a rapid response team on hospital-wide mortality and code rates outside the ICU in a Children's Hospital. *JAMA* **298,** 2267–2274 (2007).
18. Anwar-Ul-Haque, Saleem, A. F., Zaidi, S. & Haider, S. R. Experience of pediatric rapid response team in a tertiary care hospital in Pakistan. *Indian J. Pediatr.* **77,** 273–276 (2010).
19. Zhai, H. *et al.* Developing and evaluating a machine learning based algorithm to predict the need of pediatric intensive care unit transfer for newly hospitalized children. *Resuscitation* **85,** 1065–1071 (2014).
20. Garlick, J. *et al.* Modified pediatric early warning scores, a predictor of appropriate transfer to higher level of care. *Crit. Care Med.* **41,** A166–A167 (2013).
21. Medar, S., Weingarten-Arams, J. & Katyal, C. Pediatric early warning score (PEWS) is a reliable predictor of rapid response team (RRT) call. *Crit. Care Med.* **43,** 228 (2015).
22. Bell, D. *et al.* The texas children's hospital pediatric advanced warning score as a predictor of clinical deterioration in hospitalized infants and children: A modification of the pews tool. *J. Pediatr. Nurs.* **28,** (2013).
23. McLellan, M. C. & Connor, J. A. The Cardiac Children's Hospital Early Warning Score (C-CHEWS). *J. Pediatr. Nurs.* **28,** 171–178 (2013).
24. Rahman, Z. H. *et al.* Application of the burn center Pediatric Early Warning Score (PEWS) tool to clinical practice: A pilot validation study. *J. Burn Care Res.* **35,** S187 (2014).
25. Hopkins, M., Rowan, C., Rigby, M. & Tori, A. PEWS score predicts the need for PICU transfer and critical care interventions. *Crit. Care Med.* **Conference,** 43rd Critical Care Congress of the Society of Crit (2013).
26. Gawronski, O. *et al.* Bedside pews in an Italian pediatric bone marrow transplant unit: Preliminary results of a longitudinal retrospective chart review. *Intensive Care Med.* **39,** S130 (2013).
27. Sefton, G., Lane, S. & Carrol, E. D. Performance of individual predictors of deterioration used in a paediatric early warning score. *Pediatr. Crit. Care Med.* **15,** 29–30 (2014).
28. Robson, M.-A. J., Cooper, C. L., Medicus, L. a, Quintero, M. J. & Zuniga, S. a. Comparison of three acute care pediatric early warning scoring tools. *J. Pediatr. Nurs.* **28,** e33–41 (2013).
29. Chapman, S. M. *et al.* 'The Score Matters': wide variations in predictive performance of 18 paediatric track and trigger systems. *Arch. Dis. Child.* archdischild–2016–311088 (2017). doi:10.1136/archdischild-2016-311088
30. Fuijkschot, J., Vernhout, B., Lemson, J., Draaisma, J. M. T. & Loeffen, J. L. C. M. Validation of a paediatric early warning score: First results and implications of usage. *Eur. J. Pediatr.* **174,** 15–21 (2015).
31. Tucker, K. M., Brewer, T. L., Baker, R. B., Demeritt, B. & Vossmeyer, M. T. Prospective evaluation of a pediatric inpatient early warning scoring system. *J. Spec. Pediatr. Nurs.* **14,** 79–85 (2009).

For peer review only - http://bmjopen.bmj.com/site/about/guidelines.xhtml

32. Edwards, E. D., Powell, C. V, Mason, B. W. & Oliver, A. Prospective cohort study to test the predictability of the Cardiff and Vale paediatric early warning system. *Arch Dis Child* **94,** 602–606 (2009).

33. Edwards, E. D., Mason, B. W., Oliver, A. & Powell, C. V. E. Cohort study to test the predictability of the Melbourne criteria for activation of the medical emergency team. *Arch. Dis. Child.* **96,** 174–179 (2011).

34. O'Loughlin, K. *et al.* The effectiveness of the paediatric early warning tool (PEWT) in identifying children requiring admission to a critical care unit. *Arch. Dis. Child.* **97,** A154 (2012).

35. Wright, D., Sefton, G. & Horan, M. Charted observations and the use of a paediatric early warning tool did not predict the majority of cardio-respiratory arrest calls in a paediatric hospital. *Arch. Dis. Child.* **96,** A59 (2011).

36. Ahmed, M. *et al.* Burton paediatric early warning system score. *Arch. Dis. Child.* **97,** A421 (2012).

37. Mclellan, M. C., Gauvreau, K. & Connor, J. A. Validation of the cardiac children's hospital early warning score: An early warning scoring tool to prevent cardiopulmonary arrests in children with heart disease. *Congenit. Heart Dis.* **9,** 194–202 (2014).

38. Clayson, R., Selby, A. & Tume, L. Is a paediatric early warning tool (PEWT) useful in cardiac patients? *Pediatr. Crit. Care Med.* **15,** 35–36 (2014).

39. Fenix, J. B., Gillespie, C. W., Levin, A. & Dean, N. Comparison of Pediatric Early Warning Score to Physician Opinion for Deteriorating Patients. *Hosp. Pediatr.* **5,** 474–479 (2015).

40. Mandell, I. M. *et al.* Pediatric Early Warning Score and unplanned readmission to the pediatric intensive care unit. *J. Crit. Care* **30,** 1090–1095 (2015).

41. A., A., P.W., F., N., S. & C., R.-G. Validation of a Pediatric Early Warning Score in Hospitalized Pediatric Oncology and Hematopoietic Stem Cell Transplant Patients. *Pediatr. Crit. Care Med.* **17,** e146–e153 (2016).

42. Agulnik, A. *et al.* Validation of a pediatric early warning system for hospitalized pediatric oncology patients in a resource-limited setting. *Cancer* **123,** 4903–4913 (2017).

43. Blackstone, B., Sharmila, A., Pienaar, A., Bevan, R. & Cheung, R. G453(P) 'Between the flags' paediatric early warning system is a sensitive tool to detect deterioration in children from district general hospitals admitted to paediatric intensive care. *Arch. Dis. Child.* **102,** A178 LP – A179 (2017).

44. Parshuram, C. S. *et al.* Multicentre validation of the bedside paediatric early warning system score: a severity of illness score to detect evolving critical illness in hospitalised children. *Crit Care* **15,** R184 (2011).

45. Akre, M. *et al.* Sensitivity of the pediatric early warning score to identify patient deterioration. *Pediatrics* **125,** e763–9 (2010).

46. Gawronski, O. *et al.* Accuracy of Bedside Paediatric Early Warning System (BedsidePEWS) in a Pediatric Stem Cell Transplant Unit. *J. Pediatr. Oncol. Nurs.* **33,** 249–256 (2016).

47. Duncan, H., Hutchison, J. & Parshuram, C. S. The pediatric early warning system score: A severity of illness score to predict urgent medical need in hospitalized

For peer review only - http://bmjopen.bmj.com/site/about/guidelines.xhtml

children. *J. Crit. Care* **21,** 271–278 (2006).

48. Skaletzky, S. M., Raszynski, A. & Totapally, B. R. Validation of a modified pediatric early warning system score: a retrospective case-control study. *Clin. Pediatr. (Phila).* **51,** 431–5 (2012).

49. Ross, C. *et al.* Sensitivity and specificity of a pediatric early warning system using data-driven vital signs. *Crit. Care Med.* **43,** 39 (2015).

50. McLellan, M., Gauvreau, K. & Connor, J. A. Validation of the children's hospital early warning scoring system for identifying hospitalized children at risk for arrest or ICU transfer. *Pediatr. Crit. Care Med.* **15,** 30 (2014).

51. Mistry, K. P., Turi, J., Hueckel, R., Mericle, J. M. & Meliones, J. N. Pediatric Rapid Response Teams in the Academic Medical Center. *Clin. Pediatr. Emerg. Med.* **7,** 241–247 (2006).

52. Demmel, K. M., Williams, L. & Flesch, L. Implementation of the pediatric early warning scoring system on a pediatric hematology/oncology unit. *J. Pediatr. Oncol. Nurs.* **27,** 229–240 (2010).

53. Sandhu, S. *et al.* 726 The Paediatric Early Warning Tool in Combination with a Nurse-Led Outreach Team: Impact on Outcomes for Critically Ill Children. *Pediatr Res* **68,** 368 (2010).

54. Randhawa, S., Roberts-Turner, R., Woronick, K. & DuVal, J. Implementing and Sustaining Evidence-Based Nursing Practice to Reduce Pediatric Cardiopulmonary Arrest. *West. J. Nurs. Res.* **33,** 443–456 (2010).

55. Camacho, E. A., Holman, D., Badgett, V., St. Peters, J. & Southworth, B. Modification of a Pediatric Early Warning Tool for Use With Patients in a Specialized Cardiac/Renal Unit. *J. Pediatr. Nurs.* **26,** e2–e3 (2011).

56. Heyden, M. V, Mudge, B. & Casella, S. Initiation of a dedicated pediatric rapid response team reduces cardiopulmonary arrests outside of the intensive care unit in a small children's hospital. *Crit. Care Med.* **40,** 147 (2012).

57. Norville, R., Wills-Bagnato, P., Staton, S. & Kennedy-Nasser, A. Improvement in early recognition of deteriorating pediatric bone marrow transplant patients. *Biol. Blood Marrow Transplant.* **19,** S173 (2013).

58. Ambati, S. R., Sweberg, T., Silver, P. & Gangadharan, S. Effect of simulation based curriculum on the utilization of rapid response team activations (RRT). *Crit. Care Med.* **42,** A1432 (2014).

59. Ocholi, T., Downing, E., Rzeskiewicz, A., Pang, R. & Broughton, S. Impact of the Bedside PEWS on early detection of deteriorating patients. *Arch. Dis. Child.* **99,** A89–A90 (2014).

60. Panesar, R. *et al.* Characteristics and outcomes of pediatric rapid response teams before and after mandatory triggering by an elevated Pediatric Early Warning System (PEWS) score. *Hosp. Pediatr.* **4,** 135–140 (2014).

61. Kutty, S. *et al.* Association of Pediatric Medical Emergency Teams With Hospital Mortality. *Circulation* **137,** 38–46 (2018).

62. Bonafide, C. P. *et al.* Impact of Rapid Response System Implementation on Critical Deterioration Events in Children. *JAMA Pediatr.* **168,** 25 (2014).

63. Hanson, C. C. *et al.* A reduction in cardiac arrests and duration of clinical instability

For peer review only - http://bmjopen.bmj.com/site/about/guidelines.xhtml

after implementation of a paediatric rapid response system. *Postgrad. Med. J.* **86,** 314–318 (2010).

64. Kotsakis, A. *et al.* Implementation of a multicenter rapid response system in pediatric academic hospitals is effective. *Pediatrics* **128,** 72–78 (2011).

65. CS, P., Dryden-Palmer, K., Farrell, C. & al, et. Effect of a pediatric early warning system on all-cause mortality in hospitalized pediatric patients: The epoch randomized clinical trial. *JAMA* **319,** 1002–1012 (2018).

66. Kolovos, N. S., Gill, J., Michelson, P. H., Doctor, A. & Hartman, M. E. Reduction in Mortality Following Pediatric Rapid Response Team Implementation. *Pediatr. Crit. Care Med.* 1 (2018). doi:10.1097/PCC.0000000000001519

67. Tibballs, J. & Kinney, S. Reduction of hospital mortality and of preventable cardiac arrest and death on introduction of a pediatric medical emergency team. *Pediatr. Crit. Care Med.* **10,** 306–312 (2009).

68. Zenker, P. *et al.* Implementation and impact of a rapid response team in a children's hospital. *Jt. Comm. J. Qual. Patient Saf.* **33,** 418–425 (2007).

69. Sefton, G. *et al.* What impact did a Paediatric Early Warning system have on emergency admissions to the paediatric intensive care unit? An observational cohort study. *Intensive Crit. care Nurs.* **31,** 91–99 (2015).

70. Parshuram, C. S., Bayliss, A., Reimer, J., Middaugh, K. & Blanchard, N. Implementing the Bedside Paediatric Early Warning System in a community hospital: A prospective observational study. *Paediatr. Child Health* **16,** e18–22 (2011).

71. Agulnik, A. *et al.* Improved outcomes after successful implementation of a pediatric early warning system (PEWS) in a resource-limited pediatric oncology hospital. *Cancer* **123,** 2965–2974 (2017).

72. Douglas, K., Collado, J. C. & Keller, S. Implementation of a Pediatric Early Warning Scoring System at an Academic Medical Center. *Crit. Care Nurs. Q.* **39,** 363–370 (2016).

73. Duns, N. *et al.* Introduction of a track and trigger system is associated with increased paediatric intensive care outreach utilisation and a trend towards improved patient outcomes in a tertiary children's hospital. *Aust. Crit. Care* **27,** 61 (2014).

74. Chapman, S. M. *et al.* 'The Score Matters': Wide variations in predictive performance of 18 paediatric track and trigger systems. *Arch. Dis. Child.* **102,** 487–495 (2017).

75. Guerra, M. W., Shults, J., Amsterdam, J. & Ten-Have, T. The analysis of binary longitudinal data with time-dependent covariates. *Stat. Med.* **31,** 931–948 (2012).

76. Selig, J. P., Preacher, K. J. & Little, T. D. Modeling Time-Dependent Association in Longitudinal Data: A Lag as Moderator Approach. *Multivariate Behav. Res.* **47,** 697–716 (2012).

77. Clifton, D. A. *et al.* 'Errors' and omissions in paper-based early warning scores: the association with changes in vital signs-a database analysis. *BMJ Open* **5,** e007376 (2015).

78. Joffe, A. R., Anton, N. R. & Burkholder, S. C. Reduction in hospital mortality over time in a hospital without a pediatric medical emergency team: limitations of before-and-after study designs. *Arch. Pediatr. Adolesc. Med.* **165,** 419–423 (2011).

79. Goodacre, S. Uncontrolled before-after studies: Discouraged by Cochrane and the

For peer review only - http://bmjopen.bmj.com/site/about/guidelines.xhtml

EMJ. *Emergency Medicine Journal* **32,** 507–508 (2015).

80. Niegsch, M., Fabritius, M. L. & Anh??j, J. Imperfect Implementation of an Early Warning Scoring System in a Danish Teaching Hospital: A Cross-Sectional Study. *PLoS One* **8,** (2013).

81. Hands, C. *et al.* Patterns in the recording of vital signs and early warning scores: Compliance with a clinical escalation protocol. *BMJ Qual. Saf.* **22,** 719–726 (2013).

82. Lydon, S., Byrne, D., Offiah, G. & Gleeson, L. A mixed-methods investigation of health professionals' perceptions of a physiological track and trigger system. *BMJ Qual.* 688–695 (2015). doi:10.1136/bmjqs-2015-004261

83. Bonafide, C. P. *et al.* Beyond statistical prediction: Qualitative evaluation of the mechanisms by which pediatric early warning scores impact patient safety. *J. Hosp. Med.* **8,** 248–253 (2013).

84. Ennis, L. Paediatric early warning scores on a children's ward: a quality improvement initiative. *Nurs. Child. Young People* **26,** 25–31 (2014).

85. Kaul, M. *et al.* Implementation of the Bedside Paediatric Early Warning System (BedsidePEWS) for nurse identification of deteriorating patients. *J. Spec. Pediatr. Nurs.* **19,** 339–349 (2014).

86. Monaghan, A. Detecting and managing deterioration in children. *Paediatr. Care* **17,** 32–35 (2005).

For peer review only

Table 2– summary of PTTTs

PTTT name	Refs (first author, year)	Development / modification details	Score / trigger	Thresholds based on	Age-dependent thresholds?	No. of items in the tool*	Underlying components															
							Respiratory rate	Heart rate	Respiratory effort / distress	Level of consciousness / behaviour	Oxygen saturation	Capillary refill time	Oxygen therapy	Systolic blood pressure	Pain	Staff concern	Skin colour	Airway obstruction / abnormality	Temperature	Pulses	Family concern	Other items
Paediatric Early Warning System (PEWS) score	Duncan 2006[47]<br><br>Robson 2013[28]	Initial development by Duncan *et al.* for use in Canadian tertiary centre.<br><br>47 candidate items generated by focus groups with nurses; 22 items removed by separate focus group and consensus / Delphi methods with nursing and clinical staff; evaluated remaining items using a clinical dataset of cases (code blue call, n=87) and controls (n=128) – 4 items removed due to insufficient data, 5 removed due to poor discriminatory ability. 16 items remained (9 static items, 7 dynamic).<br><br>Development and validation datasets not independent.	Score	Expert opinion	Yes	16	✓	✓		✓	✓	✓	✓	✓				✓	✓	✓		Bolus fluid, medications, home oxygen, any previous admission to an ICU, central venous line in situ, transplant recipient, severe cerebral palsy, gastrostomy tube, greater than 3 medical specialties involved in care

For peer review only - http://bmjopen.bmj.com/site/about/guidelines.xhtml

Bedside Paediatric Early Warning Score (PEWS)	Parshuram 2009[11]<br>Parshuram 2011[44]<br>Parshuram 2011[70]†<br>Robson 2013[28]<br>Hopkins 2013[25]<br>Gawronski 2013[26]<br>Bonafide 2014[62]†<br>Zhai 2014[19]<br>Ocholi 2014[59]†<br>Gawronski 2016[46]<br>Parshuram 2018[65]†	Initial development by Parshuram *et al*. for use in US tertiary centre.<br>11 candidate items (all dynamic) identified based on inclusion in routine clinical assessment at local setting; discriminatory ability of individual items assessed using clinical dataset of cases (PICU admission, n=60) and controls (n=120) , 3 items removed and 3 candidate scores (using combinations of remaining 8 items) evaluated on same clinical dataset. Evaluation lead to selection of 7 item score.<br>Development and validation set not independent.	Score	Expert opinion	Yes	7	✓	✓	✓		✓	✓	✓	✓								
Modified Bedside PEWS (a)	Fuijkschot 2015[30]	Modification to Bedside PEWS for use in Dutch tertiary centre.<br>Added temperature; modified wording of respiratory effort and oxygen therapy items	Score	Expert opinion	Yes	8	✓	✓	✓		✓	✓	✓	✓					✓			
Modified Bedside PEWS (b)	Ross 2015[49]	Modification to Bedside PEWS for use in US tertiary centre.<br>Changed normal	Score	Data driven (HR and	Yes	7	✓	✓	✓		✓	✓	✓	✓								

For peer review only - http://bmjopen.bmj.com/site/about/guidelines.xhtml

		thresholds for HR and RR based on analysis of local clinical data.		RR)																		
Brighton PEWS	Monaghan 2005[86]<br><br>Randhawa 2010[54]†	Initial development by Monaghan *et al*. for use in UK tertiary centre.<br><br>Based on existing adult scores, but with some items (e.g. blood pressure) removed due to local consensus and some non-vital sign items added to reduce need to develop multiple age-adjusted charts. Persistent vomiting added and tachycardia added as scoring items after pilot implementation.<br><br>Initial audit of 30 patients who had scored at least 4 on the tool – little statistical detail given.	Score	Expert opinion	No	3	✓	✓	✓	✓		✓	✓				✓					¼ hourly nebulisers, persistent vomiting post-surgery
Modified Brighton PEWS (a)	Tucker 2008[31]<br><br>Zhai 2014[19]<br><br>Fenix 2015[39]	Modification of Brighton PEWS by Tucker *et al*.for use in general medical ward of a US tertiary centre.<br><br>Altered thresholds for oxygen therapy; changed wording for respiratory effort; modified escalation algorithm.	Score	Expert opinion	No	3	✓	✓	✓	✓		✓	✓				✓					¼ hourly nebulisers, persistent vomiting post-surgery
Modified Brighton PEWS (b)	Akre 2010[45]<br><br>Douglas 2016[72]†	Modification of Brighton PEWS by Akre *et al* for use in US tertiary centre.<br><br>Added age-dependent	Score	Expert opinion	Yes	3	✓	✓	✓	✓		✓	✓				✓					¼ hourly nebulisers, persistent vomiting post-

For peer review only

For peer review only - http://bmjopen.bmj.com/site/about/guidelines.xhtml

		thresholds for HR and RR.																				surgery
Modified Brighton PEWS (c)	Demmel 2010[52]†	Modification of Brighton PEWS for use in a US haematology / oncology unit.<br><br>Altered thresholds for oxygen therapy; changed wording for respiratory effort; modified escalation algorithm; removed nebulisers and persistent vomiting items; added parental concern.<br><br>No formal validation study reported in the literature.	Score	Expert opinion	No	3	✓	✓	✓	✓		✓	✓				✓				✓	
Modified Brighton PEWS (d)	Skaletzky 2012[48]	Modification of Brighton PEWS for use in a US tertiary centre.<br><br>Modified wording of Behaviour component, added age-dependent thresholds for HR and RR; removed nebulisers and persistent vomiting items	Score	Expert opinion	Yes	3	✓	✓	✓	✓		✓	✓				✓					
Modified Brighton PEWS (e)	Panesar 2014[60]†	Modification of Brighton PEWS for use in a US tertiary centre.<br><br>Modified wording of Behaviour component; altered thresholds for oxygen therapy; changed wording for respiratory effort; removed nebulisers and persistent vomiting items.	Score	Expert opinion	No	3	✓	✓	✓	✓		✓	✓				✓					

For peer review only - http://bmjopen.bmj.com/site/about/guidelines.xhtml

		No formal validation study reported in the literature.																				
Texas Children's Hospital (TCH) PAWS	Bell 2013[22]	Modification of Brighton PEWS for use in a US tertiary centre.<br><br>Modified wording of Behaviour component; added oxygen saturations to Respiratory component; added diaphoresis to Cardiovascular component; changed ¼ hourly nebulisers to hourly; changed oxygen therapy thresholds; modified escalation algorithm	Score	Expert opinion	No	3	✓	✓	✓	✓	✓	✓	✓				✓					Hourly respiratory treatments; persistent vomiting post-surgery
Children's Hospital Early Warning Score (CHEWS)	McLellan 2014[50]	Modification of Brighton PEWS for use in a US tertiary centre.<br><br>Altered thresholds for oxygen therapy; changed wording for Behaviour and Respiratory components; added staff concern and family concern; removed nebulisers and persistent vomiting items; modified escalation algorithm	Score	Expert opinion	No	5	✓	✓	✓	✓		✓	✓			✓	✓				✓	
Children's Hospital Cardiac Early Warning Score (C-CHEWS)	McLellan 2013[23]<br><br>Agulnik 2016[41]<br><br>Agulnik 2017[42]<br><br>Agulnik	Modification of Brighton PEWS by McLellan *et al.*, for use in a cardiac ward of a US tertiary centre.<br><br>Altered thresholds for oxygen therapy; added multiple items to Behaviour, Respiratory and	Score	Expert opinion	Yes	5	✓	✓	✓	✓	✓	✓	✓		✓	✓	✓			✓	✓	

For peer review only - http://bmjopen.bmj.com/site/about/guidelines.xhtml

	2017[71]†	Cardiovascular components; added family and staff concern; added age-dependent thresholds; removed nebulisers and persistent vomiting items; changed required actions according to certain scoring cut-offs.																				
Burn-specific PEWS	Rahman 2014[24]	Modification of Brighton PEWS, for use in a specialist Burn Centre of a US tertiary centre.<br><br>Added temperature; added intake and output scoring items; added Skin component	Score	Expert opinion	No	6	✓	✓	✓	✓	✓	✓	✓				✓			✓		Intake; outputs; skin
Children's Hospital Los Angeles (CHLA) PEWS	Mandell 2015[40]	Modification of Brighton PEWS for use in a US tertiary centre.<br><br>Added medical history as scoring item; added single ventricle physiology as scoring item; changed thresholds for oxygen therapy; added any assisted ventilation to Respiratory component.	Score	Expert opinion		4	✓	✓	✓	✓		✓	✓				✓					RRT, code blue, or transfer from/to PICU in past 2 weeks; single ventricle physiology; any assisted ventilation
Melbourne Activation Criteria (MAC)	Tibballs 2005[13]†<br><br>Tume 2007[3]<br><br>Tibballs 2009[67]†<br><br>Edwards	Initial development by Tibballs *et al*., for use in an Australian tertiary centre to activate a MET.<br><br>"The pediatric MET calling criteria were adapted from adult MET calling criteria with the addition of age-	Trigger	Expert opinion	Yes	9	✓	✓	✓	✓	✓			✓		✓		✓				Cardiac or respiratory arrest

For peer review only - http://bmjopen.bmj.com/site/about/guidelines.xhtml

	2011[33]	related abnormal recordings of heart rate, respiratory rate, and blood pressure" (Tibballs 2009).<br><br>No formal validation reported by Tibballs *et al.*																				
Modified MAC	Kotsakis 2011[64]†	Modification of MAC for use in a Canadian tertiary centre, to activate a RRS.<br><br>Removed cardiac / respiratory arrest outcome.<br><br>No formal validation reported in the literature.	Trigger	Expert opinion	Yes	8	✓	✓	✓	✓	✓			✓		✓		✓				
Cardiff & Vale Paediatric Early Warning Score (C&VPEWS)	Edwards 2009[32]<br><br>Edwards 2011[33]	Modification of MAC for evaluation in a UK tertiary centre.<br><br>Removed cardiac / respiratory arrest outcome; altered thresholds of some items; evaluated as aggregate score rather than single-item trigger.	Score	Expert opinion	Yes	8	✓	✓	✓	✓	✓			✓		✓		✓				
Bristol Paediatric Early Warning Tool (PEWT)	Haines 2006[12]<br><br>Tume 2007[3]<br><br>Wright 2011[35]<br><br>O'Loughlin 2012[34]<br><br>Robson 2013[28]	Initial development by Haines *et al.* for use in a UK tertiary centre.<br><br>Candidate items generated by reference to an un-validated tool developed in Plymouth, "with modifications from criteria developed at Melbourne Children's Hospital and similar adult systems" and expert opinion of local	Trigger	APLS values	Yes	14	✓	✓		✓	✓	✓	✓	✓	✓	✓		✓				Required nebulised adrenaline ; hyperkalaemia; suspected meningoccocus; diabetic ketoacidosis; persistent

For peer review only - http://bmjopen.bmj.com/site/about/guidelines.xhtml

		team.<br><br>Study team identified patients who would have triggered any of the candidate items over 6-month period (n=360). Several candidate items removed or modified based on discriminative ability in predicting adverse events (if at least 50% of patients triggering on any individual item subsequently required HDU/PICU transfer or death, items were retained).<br><br>Development and validation dataset not independent.																				convulsion
Modified Bristol PEWT (a)	Sefton 2015[69]†	Modification of Bristol PEWT for use in a UK tertiary centre.<br><br>Adjusted wording of Airway parameters; added effort of breathing and respiratory depression parameters; allowed for AVPU evaluation in addition to GCS; removed suspected meingococcus and diabetic ketoacidosis; added ph<7.2 and unresolved pain parameters.<br><br>No formal validation study reported in the literature.	Trigger	APLS values	Yes	14	✓	✓	✓	✓	✓	✓	✓	✓	✓	✓		✓				Required nebulised adrenaline or no improvement after nebulisers; pH<7.2; unresolved pain or current analgesic therapy; fitting

For peer review only - http://bmjopen.bmj.com/site/about/guidelines.xhtml

Modified Bristol PEWT (b)	Clayson 2014[38]	Modification of Bristol PEWT for use in a cardiac ward of a UK tertiary centre.<br><br>Amended HR and RR thresholds. Adjusted wording of Airway parameters; added effort of breathing and respiratory depression parameters; allowed for AVPU evaluation in addition to GCS; removed suspected meingococcus and diabetic ketoacidosis; added ph<7.2 and unresolved pain parameters.	Trigger	HR and RR thresh olds change d to eviden ce-based centile charts	Yes	14	✓	✓	✓	✓	✓	✓	✓	✓	✓	✓		✓				Required nebulised adrenaline or no improvem ent after nebulisers; pH<7.2; unresolve d pain or current analgesic therapy; fitting
NHS Institute for Innovation and Improvemen t (NHS III) PEWS	Mason 2016[14]	Designed as part of a NHS Institute fellowship project. Adapted from adult scores and Brighton PEWS.<br><br>No formal development or internal validation study published.	Score	APLS values	Yes	6	✓	✓	✓	✓			✓			✓						
Paediatric Medical Emergency Team (PMET) triggering criteria (a)	Brilli 2007[15]†	Initial development by Brilli *et al.*, for use in a US tertiary centre to activate a MET.<br><br>Retrospective chart review of patients requiring code call non-ICU areas (n=44) used to generate 10 most commonly recorded variables in preceding 4 hours before event.<br><br>Several combinations of	Trigger	Expert opinion	No	4			✓	✓	✓					✓	✓				✓	Worsening retractions ; cyanosis

For peer review only - http://bmjopen.bmj.com/site/about/guidelines.xhtml

		variables ("trigger sets") evaluated against case data – eventual triggers selected based on "the clinical judgment of experts, balancing each trigger set's calculated rank against the ease of measure and detection, anticipated false alarm rate, and practical considerations regarding their effective use by hospital staff"<br><br>No formal validation of final tool reported.																				
Paediatric Medical Emergency Team (PMET) triggering criteria (b)	Hunt 2008[16]†	Initial development by Hunt *et al.*, for use in a US tertiary centre to activate a MET.<br><br>Minimal description of tool development. Authors explain that they "deliberately created broad criteria to encourage calls and described categories of illness rather than using specific vital sign parameters, because the wards had children of varying ages and no single set of vital sign parameters would be appropriate".<br><br>No formal validation study reported in the literature.	Trigger	Expert opinion	Unclear	12	✓	✓	✓	✓	✓					✓					✓	Cardiac or respiratory arrest; seizures with apnoea; progressive lethargy; circulatory compromise/acute shock syndrome
Paediatric Rapid	Sharek 2007[17]†	Initial development by Sharek *et al.* for use in a	Trigger	Expert opinion	No	6	✓	✓		✓	✓			✓		✓						

For peer review only

For peer review only - http://bmjopen.bmj.com/site/about/guidelines.xhtml

Response Team (PRRT) triggering criteria (a)		US tertiary centre, to activate a RRT.<br><br>Triggering items elected through expert consensus locally – reference to similarity to Melbourne Activation Criteria and PMET triggering criteria (a).<br><br>No formal validation study reported in the literature.																				
Paediatric Rapid Response Team (PRRT) triggering criteria (b)	Anwar-al-Haque, 2010[18]†	Initial description by Anwar-al-Haque *et al.*, for use in calling RRT team in a tertiary centre in Pakistan.<br><br>Minimal explanation for selection of calling criteria. No formal validation study reported in the literature.	Trigger	Unclea r	Yes	8	✓	✓	✓	✓	✓			✓		✓						Convulsion
Logistic regression algorithm	Zhai 2014[19]	Development of an algorithm tool, based on a data mining exercise of a large, electronic health record dataset in the authors' US-based tertiary centre.<br><br>Extracted 24 hours of clinical data from children transferred to PICU within 24hrs of admission (n=526) and controls not transferred to PICU (n=6,722).<br><br>Identified 400 most commonly recorded clinical elements in health records, and then expert	Score	Expert opinion	Yes	29	✓	✓	✓	✓	✓	✓		✓	✓				✓			Acuity level (local measure); tissue perfusion and oxygenatio n

For peer review only - http://bmjopen.bmj.com/site/about/guidelines.xhtml

		consensus used to select 16 most useful clinical parameters. Created 36 variables from these 16 parameters, including maximum, minimum and mean values for some parameters over the 24hr period.<br><br>Developed a logistic regression model using 36 items, on a subset of the cases (n=473) and controls (n=473). Decided to retain 29 items (relating to 13 clinical parameters) in the final model. Final model was tested on remaining cases (n=53) and controls (n=6,299).																				

* Multiple parameters are often required to be collected for each scoring item/category, e.g., scoring the 'Cardiovascular' category in the Brighton PEWS requires collection / evaluation of heart rate, skin colour and capillary refill time

† Denotes a study included in the effectiveness review

PTTS: Paediatric Track and Trigger Tool; PICU: Paediatric Intensive Care Unit; PHDU: Paediatric High-Dependency Unit; HR: Heart rate; RR: Respiratory rate; APLS: Advanced Paediatric Life Support; AVPU: Alert, Voice, Pain, Responsive ; GCS: Glasgow Coma Scale

For peer review only

For peer review only - http://bmjopen.bmj.com/site/about/guidelines.xhtml

Table 3 – summary of PTTT validation study outcomes

PTTT	Ref (first author, year)	Country	Study population	Study design	Number of centres	PTTT used in practice?	Internal / external validation study?	Outcome measures	Sample size	Score or trigger?	Score tested / maximum score	Which score used (frequency of scoring)?*	AUROC	Sensitivity	Specificity	PPV	NPV	Notes on accuracy / reliability of scoring and missing data	Quality score (max = 24)
Paediatric Early Warning System (PEWS) score	Duncan 2006[47]	Canada	All in-patients	Case-control study (retrospective)	1	No	Int	Code blue call for actual or impending cardiopulmonary arrest	215 (87 cases)	S	5 / 26	Max 24hrs before event (hourly)	0.90	78.0	95.0	4.2†		No details on data abstraction. Data for four items from the tool could not be reliably abstracted and were excluded from analysis. 13% of eligible cases and 84% of eligible controls excluded due to incomplete clinical data. Missing data assumed to be normal.	14

For peer review only - http://bmjopen.bmj.com/site/about/guidelines.xhtml

	Robson 2013[28]	US	All in-patients	Case-control study (retrospective)	1	No	Ext	Code blue call	192 (96 cases)	S	5 / 32	Max 24hrs before event (6 hourly)	0.85	86.6	72.2			Four researchers independently scored PTTT from 20 charts, with inter-rater reliability of 0.95. Individually scored for rest of study. Interpretation of subjective variables agreed in advance within research team.<br><br>No details on extent of missing data. Missing data assumed to be normal.	8
	Chapman 2017[6]	UK	All in-patients	Case-control study (retrospective)	1	No	Ext	Death, arrest or unplanned PICU transfer	608 (297 cases)	S	7 / 32	Max 48hrs before event (per usual practice)	0.82	70.0	75.0	*72.6*	*72.0*	Data abstraction completed by single researcher.<br><br>36% of observation sets contained HR, RR, O2 Sats, systolic BP, temperature and assessment of consciousness. Missing data assumed to be normal.	17
Bedside PEWS	Parshuram 2009[11]	Canada	All in-patients	Case-control study (retrospective)	1	No	Int	Urgent PICU transfer (without code blue call)	180 (60 cases)	S	8 / 26	Max 24hrs before event (hourly)	0.91	82.0	93.0			Accuracy of data abstraction not assessed.<br><br>Availability of scoring items in medical records varied from 27% (cap refill time) to 93% (oxygen therapy). Missing data assumed to be normal.	21

For peer review only

For peer review only - http://bmjopen.bmj.com/site/about/guidelines.xhtml

	Parshuram 2011[44]	Canada & UK	All in-patients	Case-control study (prospective)	4	No	Ext	Urgent PICU transfer or immediate call to resuscitation team	2,074 (686 cases)	S	7 / 26	Max 24hrs before event (hourly)	0.87	64.0	91.0			PTTT scores calculated electronically after abstraction from charts by single research nurse. No details on accuracy / reliability of scoring.<br><br>5.1% of observation sets had all 7 items recorded, 31% had at least 5 items.	22
	Robson 2013[28]	US	All in-patients	Case-control study (retrospective)	1	No	Ext	Code blue call	192 (96 cases)	S	7 / 26	Max 24hrs before event (6 hourly)	0.73	56.3	78.1			See above.	8
	Zhai 2014[19]	US	All in-patients	Case-control study (retrospective)	1	No	Ext	Urgent PCU transfer within 24 hrs of admission	6,352 (53 cases)	S	7 / 26	Max 24 hrs before event (hourly)	0.82	73.6	71.7	2.1†		Data directly extracted from electronic health records.<br><br>Authors reported minimal missing data. Excluded two items of Bedside PEWS (oxygen therapy and respiratory effort) from analysis due to difficulty abstracting from health records.	17

For peer review only - http://bmjopen.bmj.com/site/about/guidelines.xhtml

	Gawronski 2016[46]	Italy	Stem Cell Transplant Unit	Case-control study (retrospective)	1	No	Ext	Unexpected death, urgent consult with RRT or urgent PICU transfer	99 (19 cases)	S	6 / 26	Score 4hrs before event	0.90	79.0	97.5			Data abstracted by research nurses. No details on accuracy / reliability.<br><br>No details on extent of missing data. Conflicting or missing observations resolved by interviews with clinical staff.	15
	Chapman 2017[74]	UK	All in-patients	Case-control study (retrospective)	1	No	Ext	Death, arrest or PICU transfer	608 (297 cases)	S	6 / 26	Max 48hrs before event (per usual practice)	0.88	72.0	89.0	*86.0*	*77.0*	See above.	17
Modified Bedside PEWS (a)	Fuijkschot 2015[30] (study 1)	Netherlands	Oncology ward	Case-cohort study (retrospective)	1	Yes	Int	Emergency medical intervention or reviewed by PICU staff or clinical condition considered 'sick' by staff	118 (15 cases)	S	8 / 28	Unclear (minimum 8 hourly)				*73.0*		PTTT used in practice – no details on accuracy / reliability of scoring.<br><br>41% of admissions excluded from study due to incomplete PTTT scores.	10
	Fuijkschot 2015[30] (study 2)	Netherlands	All in-patients	Case-cohort study (retrospective)	1	Yes	Int	PICU transfer	Unclear (24 cases)	S	8 / 28	Score 2-6hrs before event (minimum 8 hourly)		66.6				PTTT used in practice – no details on accuracy / reliability of scoring.<br><br>High rate of exclusions reported due to missing data.	10

For peer review only - http://bmjopen.bmj.com/site/about/guidelines.xhtml

	Fuijkschot 2015[30] (study 3)	Netherlands	All in-patients	Case-cohort study (prospective)	1	Yes	Int	Emergency medical intervention	Unclear (14 cases)	S	8 / 28	Unclear (minimum 8 hourly)		100				PTTT used in practice – no details on accuracy / reliability of scoring.<br><br>No details on missing data.	10
	Chapman 2017[74]	UK	All in-patients	Case-control study (retrospective)	1	No	Ext	Death, arrest or PICU transfer	608 (297 cases)	S	7 / 28	Max 48hrs before event (per usual practice)	0.87	69.0	91.0	*87.9*	*79.0*	See above.	17
Modified Bedside PEWS (b)	Ross 2015[49]	US	All in-patients	Case-control study (retrospective)	1	No	Int	Urgent PICU transfer	4628 (848 cases)	S	8 / 26	Max during admission		70.0	84.0			No details on data abstraction.<br><br>Respiratory effort category excluded due to difficulty abstracting from local charts. No details on missing data.	9
Modified Brighton PEWS (a)	Tucker 2008[31]	US	General medical unit	Cohort study (prospective)	1	Yes	Int	PICU transfer	2,979 (51 cases)	S	3 / 11	Max during admission (4 hourly)	0.89	90.2	74.4	5.8	99.8	PTTT used in practice by bedside nurses. Two bedside nurses independently scored 55 patients, within minutes of each other. Intraclass coefficient of 0.92 reported.<br><br>No discussion of missing data.	14

For peer review only - http://bmjopen.bmj.com/site/about/guidelines.xhtml

	Zhai 2014[19]	US	All in-patients	Case-control study (retrospective)	1	No	Ext	Urgent PCU transfer within 24 hrs of admission	6,352 (53 cases)	S	2 / 11	Max 24hrs before event (hourly)	0.74	68.4	81.6	2.3		Data directly extracted from electronic health records.<br><br>Only included observation sets with a complete Brighton PEWS score (tool used in practice locally): 64% of eligible cases and 51% of eligible controls were excluded due to missing data.	17
	Fenix 2015[39]	US	PICU transfers among all in-patients (excluding haematology oncology, surgical and cardiac wards)	Case-control study (retrospective)	1	Yes	Ext	Non-elective PICU transfer followed by any intubation, inotropes, HFNC, non-invasive mechanical ventilation, or aggressive fluid resuscitation (>60 mL/kg) within 12 hours of transfer	97 PICU transfers (51 cases of PICU transfer followed by ‘deterioration event’)	S	3 / 11	Max during admission		80.0	43.0	*61.0*	*67.0*	PTTT used in practice – no details on accuracy / reliability of scoring.<br><br>No details on missing data.	15

For peer review only - http://bmjopen.bmj.com/site/about/guidelines.xhtml

Modified Brighton PEWS (b)	Akre 2010[45]	US	All in-patients	Chart review study (retrospective)	1	No	Int	Rapid response team call or code blue call	186 cases (170 RRT calls, 16 code calls)	S	4 / 13	Max 24 hrs before event (minimum 4 hourly)		85.5				Scores abstracted from charts by single nurse, initially working with advanced practice nurse. Independently scored same charts "until interrater reliability was achieved on a sample of 10 records".<br><br>Categories scored as missing unless all necessary data from charts available. 25% of charts missing behavioural state and 26% missing cardiovascular colour. No details on how missing data was handled.	14
	Chapman 2017[74]	UK	All in-patients	Case-control study (retrospective)	1	No	Ext	Death, arrest or PICU transfer	608 (297 cases)	S	4 / 13	Max 48hrs before event (per usual practice)	0.79	61.0	84.0	*78.4*	*69.0*	See above.	17

For peer review only

Modified Brighton PEWS (d)	Skaletzky 2012[48]	US	Medical surgical wards	Case-control study (retrospective)	1	No	Int	PICU transfer	350 (100 cases)	S	2.5 / 9	Max 48hrs before event (4 hourly)	0.81	62.0	89.0			Data abstracted from medial charts and notes. No details on reliability / validity of scoring. Score for behavioural category derived from level of consciousness assessment in charts, authors describe process as "subject to varying interpretations".<br><br>No details on missing data.	15
	Chapman 2017[74]	UK	All in-patients	Case-control study (retrospective)	1	No	Ext	Death, arrest or PICU transfer	608 (297 cases)	S	4 / 9	Max 48hrs before event (per usual practice)	0.74	46.0	90.0	*81.3*	*63.0*	See above.	17
Children's Hospital Early Warning Score (CHEWS)	McLellan 2014[50]	US	All in-patients	Case-control study (retrospective)	1	Yes	Int	Arrest or unplanned PICU transfer	1,136 (360 cases)	S	4 / 12	Max in admission (4 hourly)	0.90	84.2	80.9			PTTT used in practice. No details on accuracy / reliability of scoring.<br><br>No details on missing data.	10

For peer review only

For peer review only - http://bmjopen.bmj.com/site/about/guidelines.xhtml

Children's Hospital Cardiac Early Warning Score (C-CHEWS)	McLellan 2013[23]	US	Cardiovascular unit	Case-control study (retrospective)	1	Yes	Int	Arrest or unplanned PICU transfer	312 (64 cases)	S	3 / 12	Max 18hrs before event (4 hourly)	0.86	95.3	76.2	*50.8*	*98.4*	PTTT used in practice. Study nurse and bedside nurses independently assessed scores for 37 patients, with 67% agreement.<br><br>No details on missing data.	9
	Agulnik 2016[41]	US	Oncology unit	Case-control study (retrospective)	1	Yes	Ext	Unplanned PICU transfer	330 (110 cases)	S	4 / 12	Max 24 hours before event (4 hourly)	0.96	86.0	95.0			PTTT used in practice, documented by nursing staff. No details about accuracy /reliability of scoring.<br><br>Did not abstract instances where vital signs were present but no PTTT score calculated by nurse. No details on missing data.	14

For peer review only

	Agulnik 2017[42]	Guatemala	Oncology unit	Case-control study (retrospective)	1	Yes	Ext	Unplanned PICU transfer	258 (129 cases)	S	4 / 12	Max 24hrs before event (3 hourly)		91.0	88.0			PTTT used in practice, documented by nursing staff. Researcher evaluated charts and calculated scores, reporting 14% error rate (PTTT score calculated incorrectly) and 3% omission rate (vital signs recorded but no PTTT score calculated).<br><br>1 out of 130 cases excluded due to missing PTTT documentation.	16
Children's Hospital Los Angeles (CHLA) PEWS	Mandell 2015[40]	US	In-patients discharged from PICU to ward	Case-control study (retrospective)	1	Yes	Int	Early unplanned re-admission to PICU (within 48 hours of discharge from PICU to ward)	189 (38 cases)	S	2 / 10	First score assigned on ward, post PICU discharge	0.71	76.0	56.0			PTTT used in practice. No details on accuracy / reliability of scoring.<br><br>No details on missing data.	12
Melbourne Activation Criteria (MAC)	Tume 2007[3]	UK	In-patients with an unplanned PICU transfer	Chart review study (retrospective)	1	No	Ext	Unplanned PICU transfer	33 cases	T	NA	Unclear		87.8				Data abstracted by two reviewers. No details on accuracy / reliability of scoring.<br><br>Reference to "large number of missing records and observation charts".	11

For peer review only

	Tume 2007[3]	UK	In-patients with an unplanned PHDU transfer	Chart review study (retrospective)	1	No	Ext	Unplanned PHDU transfer	32 cases	T	N/A	Unclear		87.5				See above.	11
	Edwards 2011[33]	UK	All in-patients	Cohort study (retrospective)	1	No	Ext	Death or unplanned PICU or HDU transfer	1,000 (16 cases)	T	N/A	Any trigger over admission (per usual practice)	0.79	68.3	83.2	3.6	99.7	Observation charts altered to include all PTTT parameters but PTTT not used in practice. Data abstracted and scores calculated by researcher. No detail on reliability / accuracy of scoring.<br><br>56% of records reviewed were missing at least one required PTTT criteria. Missing data assumed to be normal.	17
	Chapman 2017[74]	UK	All in-patients	Case-control study (retrospective)	1	No	Ext	Death, arrest or PICU transfer	608 (297 cases)	T	NA	Max 48hrs before event (per usual practice)	0.71	93.0	49.0	*64.0*	*88.0*	See above.	17

For peer review only

For peer review only - http://bmjopen.bmj.com/site/about/guidelines.xhtml

Cardiff & Vale Paediatric Early Warning Score (C&VPEWS)	Edwards 2009[32]	UK	All in-patients	Cohort study (prospective)	1	No	Int	Death or unplanned PICU or HDU transfer	1,000 (16 cases)	S	2 / 8	Max score during admission (per usual practice)	0.86	69.5	89.9	5.9	99.7	Observation charts altered to include all PTTT parameters but PTTT not used in practice. Data abstracted and scores calculated by researcher. No detail on reliability / accuracy of scoring.<br><br>56% of records reviewed were missing at least one required PTTT criteria. Missing data assumed to be normal.	18
	Chapman 2017[74]	UK	All in-patients	Case-control study (retrospective)	1	No	Ext	Death, arrest or PICU transfer	608 (297 cases)	S	3 / 8	Max 48hrs before event (per usual practice)	0.89	80.0	86.0	*84.0*	*82.0*	See above.	17
Bristol Paediatric Early Warning Tool (PEWT)	Tume 2007[3]	UK	In-patients with an unplanned PICU transfer	Chart review (retrospective)	1	No	Ext	Unplanned PICU transfer	33 cases	T	N/A	Unclear		87.8				Data abstracted by two reviewers. No details on accuracy / reliability of scoring.<br><br>Reference to "large number of missing records and observation charts".	11
	Tume 2007[3]	UK	In-patients with an unplanned PHDU transfer	Chart review (retrospective)	1	No	Ext	Unplanned PHDU transfer	32 cases	T	N/A	Unclear		84.4				See above.	11

For peer review only

For peer review only - http://bmjopen.bmj.com/site/about/guidelines.xhtml

	Wright 2011[35]	UK	All in-patients	Chart review (retrospective)	1	Yes	Ext	Cardiac arrest	55 cases	T	N/A	If triggered 24hrs before event		49.1				PTTT used in practice. No details on accuracy / reliability of scoring.<br><br>One case excluded due to missing notes. No details on missing data.	11
	O'Loughlin 2012[34]	UK	All in-patients	Cohort study (prospective)	1	Yes	Ext	PICU transfer	331 (7 cases)	T	N/A	Triggered during admission (12hrly)	0.91	100	81.0	11.0		PTTT used in practice. No details on accuracy / reliability of scoring.<br><br>No details on missing data.	6
	Robson 2013[28]	US	All in-patients	Case-control study (retrospective)	1	No	Ext	Code blue call	192 (96 cases)	T	N/A	Triggered 24hrs before event (6hrly)	0.75	76.3	61.5			See above.	8
	Chapman 2017[74]	UK	All in-patients	Case-control study (retrospective)	1	No	Ext	Death, arrest or PICU transfer	608 (297 cases)	T	N/A	If triggered 48hrs before event (per usual practice)	0.62	96.0	28.0	*56.0*	*88.0*	See above.	17
Modified Bristol Paediatric Early Warning Tool (PEWT) (b)	Clayson 2014[38]	UK	Cardiac ward	Cohort study (prospective)	1	Yes	Int	'A deteriorating patient'	126 (unclear number of cases)	T	N/A	Unclear				*12.5*	*97.0*	PTTT used in practice. No details on accuracy / reliability of scoring.<br><br>No details on missing data.	5

For peer review only - http://bmjopen.bmj.com/site/about/guidelines.xhtml

NHS Institute for Innovation and Improvement (NHS III) PEWS	Mason 2016[14]	UK	All in-patients	Cohort study (retrospective)	1	No	Ext	Death or unplanned PICU or HDU transfer	1,000 (16 cases)	S	2 / 7	Max score over admission (per usual practice)	0.88	80.0	81.0	4.3	99.7	Observation charts altered to include all PTTT parameters but PTTT not used in practice. Data abstracted and scores calculated by researcher. No detail on reliability / accuracy of scoring.<br><br>56% of records reviewed were missing at least one required PTTT criteria. Missing data assumed to be normal.	15
	Chapman 2017[74]	UK	All in-patients	Case-control study (retrospective)	1	No	Ext	Death, arrest or PICU transfer	608 (297 cases)	S	2 / 7	Max 48hrs before event (per usual practice)	0.82	83.0	65.0	*69.6*	*80.0*	See above.	17
Logistic regression algorithm	Zhai 2014[19]	US	All in-patients	Case-control study (retrospective)	1	No	Ext	Urgent PICU transfer within 24 hrs of admission	6,352 (53 cases)	S	> 0.5	Max 24hrs before event (hourly)	0.91	84.9	85.9	4.8		Data directly extracted from electronic health records.<br><br>Missing values assigned separate category in regression model. No details on extent of missing data but authors report that "missing data was a major cause of incorrect prediction".	17

For peer review only - http://bmjopen.bmj.com/site/about/guidelines.xhtml

Burton Paediatric Early Warning Score (BPEWS)	Ahmed 2012[36]	UK	PICU admissions only	Chart review (retrospective)	1	Yes	Int	PICU admission	23	S	4 / 19	Max 24hrs before event (unclear)		93.0				Data extracted from case notes by two reviewers. No details on reliability / accuracy of scoring.<br><br>No details on missing data.	4
'Between the Flags' Paediatric Early Warning System (PEWS)	Blackstone 2017[43]	UK	Urgent PICU admissions only	Chart review (retrospective)	1	Yes	Ext	Urgent PICU admission	100	T	NA	Unclear		91.0				Data extracted from health records.<br><br>No details on missing data.	8

All studies conducted in a specialist / tertiary centre.

PPV and NPV values in italics represent results from case-control studies – these values are misleading in isolation because they assume that the wider prevalence rate of the adverse event is equal to the case to control ratio used in the research study (e.g., if the researchers studied 300 cases and 300 controls, the prevalence rate of adverse events for the calculation of PPV is 50%). As per the cohort studies, prevalence rates of critical events are typically far lower among hospitalised paediatric populations than the case/control ratios used in studies, and so PPV values would be considerably lower in clinical practice.

Studies classified as internal validation if the setting for the study was the same hospital and same research team as those who developed the score. Studies classified as external validation if the score was tested in a different centre and by a different research team to those who developed it.

* Typically, study researchers collected or abstracted multiple PTTT scores for each patient at different time points, but can only use one score per patient for the analysis of the tool's predictive ability. This column specifies which score the researchers used. In most cases, the study team used the maximum PTTT score recorded for each patient in a given study window – e.g., 24 hours prior to a critical event for case patients. The text in parentheses describes the frequency with which scores were assessed or abstracted for each patient, if this information was described in the paper.

† Case-control study, but PPV value calculated based on clinical prevalence of event as measured at local centre during the study

PTTT, paediatric track and trigger tool; S, score; T, trigger; AUROC, area under the receiver operating characteristic curve; PPV, positive predictive value, NPV, negative predictive value; PICU, paediatric intensive care unit; PHDU, paediatric high-dependency unit; RRT, rapid response team; HFNC, high flow nasal cannula; UK, United Kingdom; US, United States; Int, Internal validation; Ext, external validation

For peer review only

For peer review only - http://bmjopen.bmj.com/site/about/guidelines.xhtml

Table 4 – summary of early warning system effectiveness study outcomes

Outcome	Refs (first author, year)	Intervention: Implemented a new PTTT	Intervention: Implemented new RRT / MET	Intervention: Modified escalation process	Intervention: Staff training / education	PTTT	Country	Number of centres	Specialist unit?	Existing RRT / MET?	Population	Study design	Study duration in months	Events before, *n* (rate)	Events after, *n* (rate)	Effect size (95% CI)	P Value	Description of findings	Quality score (max = 26)
**MORTALITY**																			
Deaths on ward (per 1,000 admissions)	Tibballs 2005[13]	✓	✓		✓	Melbourne Activation Criteria (MAC)	Australia	1	Y	N	All in-patients	Uncontrolled before-after study (prospective)	53 (41 before, 12 after)	13 (0.12)	2 (0.06)	RR = 0.45 (0.10-1.99) †	0.29	No change in rate of deaths on ward, post intervention	10
Hospital-wide deaths (per 100 discharges)	Sharek 2007[17]	✓	✓		✓	Paediatric Rapid Response Team (PRRT) triggering criteria	US	1	Y	N	All in-patients	Uncontrolled before-after study (prospective)	84 (67 before, 17 after)	547 (1.01)	158 (0.83)	RR = 0.82 (0.70-0.95)	**.007**	**Significant reduction in rate of deaths per 100 dischargers post intervention**	15
Hospital wide deaths, excluding neonate ICU and ED (per 1,000 discharges)	Zenker 2007[68]	✓	✓			RRT activation criteria*	US	1	Y	N	All in-patients	Uncontrolled before-after study (prospective)	34 (23 before, 11 after)	97 (4.30)	52 (4.45)	RR=1.04 (0.74-1.45) †	.57	No change in rate of hospital-wide deaths, post intervention	12

For peer review only - http://bmjopen.bmj.com/site/about/guidelines.xhtml

Deaths outside ICU (per 1,000 non-ICU patient-days)	Brilli 2007[15]	✓	✓		✓	Paediatric Medical Emergency Team (PMET) triggering criteria (a)	US	1	Y	N	All in-patients	Uncontrolled before-after study (prospective)	27 (15 before, 12 after)	9 (0.10)	2 (0.04)	RR=0.39 (0.08-1.80) †	.13	Non-significant trend towards reduced rate of mortality outside of ICU, post intervention	14
Ward death rate (per 1,000 ward admissions)	Hanson 2010[63]	✓	✓		✓	*Not described*	US	1	Y	N	All in-patients	Uncontrolled before-after study (retrospective)	36 (24 before, 12 after)	13 (1.50)	2 (0.45)	RR = 0.30 (0.07–1.31) †	.07	Non-significant trend towards reduced rate of ward deaths, post intervention	18
Total hospital deaths (per 1,000 admissions)	Tibballs 2009[67]	✓	✓		✓	Melbourne Activation Criteria (MAC)	Australia	1	Y	N	All in-patients	Uncontrolled before-after study (prospective)	89 (41 before, 48 after)	459 (4.38)	398 (2.87)	RR = 0.65 (0.57-0.75)	**< .0001**	**Significant reduction in rate of total hospital deaths, post intervention**	15
Deaths on ward (per 1,000 admissions)	Tibballs 2009[67]	✓	✓		✓	Melbourne Activation Criteria (MAC)	Australia	1	Y	N	All in-patients	Uncontrolled before-after study (prospective)	89 (41 before, 48 after)	13 (0.12)	6 (0.04)	RR = 0.35 (0.13-0.92)	**.03**	**Significant reduction in rate of deaths on ward ('unexpected deaths'), post intervention**	15
All-cause hospital mortality (per 1,000 admissions)	Kotsakis 2011[64]	✓	✓			Modified MAC	Canada	4	Y	N	All in-patients	Uncontrolled before-after study (prospective)	48 (24 before, 24 after)	553 (9.97)	540 (9.65)	RR = 0.97 (0.83-1.12)	.65	No change in all-cause hospital mortality rate post intervention	18
All cause hospital mortality (per 1,000 discharges)	Parshuram 2018[65]	✓	✓		✓	Bedside PEWS	Belgium, Ireland, Netherlands, England, Italy, Canada, New Zealand	21	Y	N	All in-patients	Cluster randomised trial (prospective)	18 (6 pre, 12 post)	Con: 61 (1.31)<br>Int: 52 (1.95)	Con: 147 (1.56)<br>Int: 97 (1.93)	OR=1.01 (0.61-1.69)	.96	No significant difference in pre and post intervention mortality rates between intervention and usual care hospitals	23

For peer review only - http://bmjopen.bmj.com/site/about/guidelines.xhtml

Hospital mortality (per 1,000 admissions)	Kutty 2018[61]		✓			NR	US	38	Y	N	All in-patients	Interrupted Time Series (retrospective)	180 (60 before, 120 after)	NA	NA	OR=0.94 (0.93-0.95)	.98	No significant difference in trend of adjusted mortality rate five years post intervention, compared to expected trend based on pre-implementation trajectory	20
**PICU MORTALITY**																			
PICU mortality after PICU admission from ward (per PICU admission)	Anwar-al-Haque, 2010[18]	✓	✓			Paediatric Rapid Response Team (PRRT) triggering criteria (b)	Pakistan	1	Y	N	All in-patients	Uncontrolled before-after study (retrospective)	18 (9 before, 9 after)	23 (51.11)	5 (15.63)	RR = 0.31 (0.13-0.72) †	**.007†**	**Significant reduction in mortality rate among patients admitted to PICU from ward, post intervention**	6
PICU mortality after PICU readmission within 48 hrs of discharge (per 1,000 admissions)	Kotsakis 2011[64]	✓	✓			Modified MAC	Canada	4	Y	N	All in-patients	Uncontrolled before-after study (prospective)	48 (24 before, 24 after)	16 (0.29)	7 (0.13)	RR = 0.43 (0.17-0.99)	**<.05**	**Significant reduction in mortality rate among patients readmitted to PICU within 48hrs of discharge, post intervention**	18
PICU mortality after urgent PICU admission from ward (per 1,000 admissions)	Kotsakis 2011[64]	✓	✓			Modified MAC	Canada	4	Y	N	All in-patients	Uncontrolled before-after study (prospective)	48 (24 before, 24 after)	70 (1.3)	61 (1.1)	RR = 0.90 (0.70-1.00)	.25	No change in PICU mortality rate after urgent PICU admission, post intervention	18

For peer review only

For peer review only - http://bmjopen.bmj.com/site/about/guidelines.xhtml

Death prior to discharge (per unplanned PICU transfer)	Bonafide 2014[62]	✓	✓			Bedside PEWS	US	1	Y	N	All in-patients	Interrupted Time Series study (prospective)	59 (32 before, 27 after)	51 (6.3)	56 (6.5)	RR = 1.03 (0.72-1.49) †	.99	No change in death rate prior to discharge among unplanned PICU transfers post intervention	23
PICU mortality (per PICU admission)	Duns 2014[73]	✓				Between the Flags (BTS) tool*	Australia	1	Y	Y	All in-patients	Uncontrolled before-after study (prospective)	48 (24 before, 24 after)	30 (8.57)	20 (5.49)	RR=0.64 (0.37-1.11) †	.14	Non-significant trend towards reduction in rate of PICU mortality after PICU admission, post intervention	7
Death in PICU (per 1,000 patient-days)	Agulnik 2017[71]	✓			✓	Children's Hospital Cardiac Early Warning Score (C-CHEWS)	Guatemala	1	Y	N	Oncology unit	Uncontrolled before-after study (retrospective)	24 (12 before, 12 after)	21 (1.25)	22 (1.10)	RR=0.89 (0.49-1.61) †	.76	No change in rate of deaths in PICU, post intervention	19
Death in PICU (per emergency PICU admission)	Sefton 2015[69]	✓		✓	✓	Modified Bristol PEWT (a)	UK	1	Y	N	All PICU admissions	Controlled before-after study (retrospective)	24 (12 before, 12 after)	17 (10.8)	14 (8.4)	RR = 0.78 (0.40-1.53) †	.47	No change in PICU mortality rate among PICU emergency admissions, post intervention	16
Deaths in PICU (per unplanned PICU admission)	Kolovos, 2018	✓	✓			RRT activation criteria*	US	1	Y	N	All unplanned PICU admissions	Uncontrolled before-after study (retrospective)	78 (42 before, 36 after)	54† (4.9)	40† (3.8)	RR = 0.77 (0.52–1.15) †	.20†	No change in PICU mortality rate among PICU admissions, post interventions	12
PICU mortality (per 1,000 discharges)	Parshuram 2018[65]	✓	✓		✓	Bedside PEWS	Belgium, Ireland, Netherlands, England, Italy, Canada, New Zealand	21	Y	N	All in-patients	Cluster randomised trial (prospective)	18 (6 pre, 12 post)	Con: 34 (0.73)<br>Int: 33 (1.24)	Con: 91 (0.96)<br>Int: 56 (1.12)	OR=0.95 (0.48-1.86)	.88	No significant difference in pre and post intervention PICU mortality rate between intervention and usual care hospitals	23
**CARDIAC ARREST**																			

For peer review only

For peer review only - http://bmjopen.bmj.com/site/about/guidelines.xhtml

Cardiac arrests on ward (per 1,000 admissions)	Tibballs 2005[13]	✓	✓		✓	Melbourne Activation Criteria (MAC)	Australia	1	Y	N	All in-patients	Uncontrolled before-after study (prospective)	53 (41 before, 12 after)	20 (0.19)	4 (0.11)	RR = 0.58 (0.20-1.70)	.33	No change in rate of cardiac arrests on ward, post intervention	10
Cardiopulmonary arrests (per 1,000 non-ICU patient-days)	Brilli 2007[15]	✓	✓		✓	Paediatric Medical Emergency Team (PMET) triggering criteria (a)	US	1	Y	N	All in-patients	Uncontrolled before-after study (prospective)	27 (15 before, 12 after)	7 (0.08)	2 (0.04)	RR=0.50 (0.10-2.42) †	.11	Non-significant trend towards reduction in rate of cardiopulmonary arrests, post intervention	14
Ward cardiac arrest rate (per 1,000 ward admissions)	Hanson 2010[63]	✓	✓		✓	*Not described*	US	1	Y	N	All in-patients	Uncontrolled before-after study (retrospective)	36 (24 before, 12 after)	11 (1.27)	2 (0.45)	RR = 0.35 (0.08–1.58) †	.13	Non-significant trend towards reduced cardiac arrest rate, post intervention	18
Ward cardiopulmonary arrests (per 1,000 patient-days)	Hunt 2008[16]	✓	✓			Paediatric Medical Emergency Team (PMET) triggering criteria	US	1	Y	N	All in-patients	Uncontrolled before-after study (prospective)	24 (12 before, 12 after)	5 (0.10)	5 (0.10)	RR = 0.98 (0.22–4.24)	.97	No change in cardiopulmonary arrest rate, post intervention	17
Preventable cardiac arrests (per 1,000 admissions)	Tibballs 2009[67]	✓	✓		✓	Melbourne Activation Criteria (MAC)	Australia	1	Y	N	All in-patients	Uncontrolled before-after study (prospective)	89 (41 before, 48 after)	17 (0.16)	10 (0.07)	RR = 0.45 (0.20-0.97)	**.04**	**Significant reduction in rate of 'preventable cardiac arrests', post intervention**	15
Unexpected cardiac arrests (per 1,000 admissions)	Tibballs 2009[67]	✓	✓		✓	Melbourne Activation Criteria (MAC)	Australia	1	Y	N	All in-patients	Uncontrolled before-after study (prospective)	89 (41 before, 48 after)	20 (0.19)	24 (0.17)	RR = 0.91 (0.50-1.64)	.75	No change in rate of 'unexpected cardiac arrests', post intervention	15

For peer review only - http://bmjopen.bmj.com/site/about/guidelines.xhtml

Actual cardiopulmonary arrests (per 1,000 ward admissions)	Kotsakis 2011[64]	✓	✓			Modified MAC	Canada	4	Y	N	All in-patients	Uncontrolled before-after study (prospective)	48 (24 before, 24 after)	69 (1.9)	66 (1.8)	RR = 0.95 (0.76-1.96)	.68	No change in rate of cardiopulmonary arrests, post intervention	18
Near cardiopulmonary arrests (per 1,000 admissions)	Kotsakis 2011[64]	✓	✓			Modified MAC	Canada	4	Y	N	All in-patients	Uncontrolled before-after study (prospective)	48 (24 before, 24 after)	123 (3.4)	67 (1.9)	RR = 0.54 (0.52-0.57)	**<.001**	**Significant reduction in rate of 'near cardiopulmonary arrests' post intervention**	18
Cardiac arrests on ward (per 1,000 non-ICU patient-days)	Bonafide 2014[62]	✓	✓			Bedside PEWS	US	1	Y	N	All in-patients	Interrupted Time Series study (prospective)	59 (32 before, 27 after)	6† (0.03)	2† (0.01)	RR = 0.36 (0.07-1.78) †	.21	No significant difference in rate of ward cardiac arrests, post intervention	23
Cardiac arrests (per 1,000 patient-days)	Parshuram 2018[65]	✓	✓		✓	Bedside PEWS	Belgium, Ireland, Netherlands, England, Italy, Canada, New Zealand	21	Y	N	All in-patients	Cluster randomised trial (prospective)	18 (6 pre, 12 post)	Con: 18 (0.11)<br>Int: 15 (0.12)	Con: 32 (0.10)<br>Int: 27 (0.11)	RR=1.02 (0.65-1.62)	.92	No significant difference in pre and post intervention cardiac arrest rate between intervention and usual care hospitals	23
**RESPIRATORY ARREST**																			
Ward respiratory arrests (per 1,000 patient-days)	Hunt 2008[16]	✓	✓			Paediatric Medical Emergency Team (PMET) triggering criteria	US	1	Y	N	All in-patients	Uncontrolled before-after study (prospective)	24 (12 before, 12 after)	11 (0.23)	3 (0.06)	RR = 0.27 (0.05-1.01)	**.03**	**Significant reduction in respiratory arrest rate, post intervention**	17
**CARDIAC OR RESPIRATORY ARREST**																			

For peer review only - http://bmjopen.bmj.com/site/about/guidelines.xhtml

Cardiac or respiratory arrest (per 1,000 discharges)	Zenker 2007[68]	✓	✓			RRT activation criteria*	US	1	Y	N	All in-patients	Uncontrolled before-after study (prospective)	34 (23 before, 11 after)	180 (7.98)	60 (5.13)	RR=0.64 (0.48-0.86) †	.19	Non-significant trend towards reduction in rate of cardiac and respiratory arrests, post intervention	12
**CALLS FOR URGENT REVIEW / ASSISTANCE**																			
Code calls (per 1,000 non-ICU patient-days)	Brilli 2007[15]	✓	✓		✓	Paediatric Medical Emergency Team (PMET) triggering criteria (a)	US	1	Y	N	All in-patients	Uncontrolled before-after study (prospective)	27 (15 before, 12 after)	25 (0.27)	6 (0.11)	RR=0.42 (0.17-1.03) †	**.03**	**Significant reduction in rate of code calls, post intervention**	14
Code calls (per 1,000 non-ICU patient-days)	Sharek 2007[17]	✓	✓		✓	Paediatric Rapid Response Team (PRRT) triggering criteria	US	1	Y	N	All in-patients	Uncontrolled before-after study (prospective)	84 (67 before, 17 after)	53 (0.52)	5 (0.15)	RR = 0.29 (0.10-0.65)	**.008**	**Significant reduction in rate of code calls, post intervention**	15
Code calls (per 1,000 admissions)	Anwar-al-Haque 2010[18]	✓	✓			Paediatric Rapid Response Team (PRRT) triggering criteria (b)	Pakistan	1	Y	N	All in-patients	Uncontrolled before-after study (retrospective)	18 (9 before, 9 after)	26 (5.25)	12 (2.73)	RR = 0.52 (0.26-1.03)	.06	Non-significant trend towards reduction in rate of code calls, post intervention	6
Urgent calls to respiratory therapist (per 1,000 patient-days)	Parshuram 2011[70]	✓		✓	✓	Bedside PEWS	Canada	1	N	N	All in-patients	Uncontrolled before-after study (prospective)	8 (3 before, 5 after)	8 (9.5)	8 (3.4)	RR = 0.36 (0.13-0.95) †	**<.0001**	**Significant reduction in rate of urgent calls to respiratory therapist, post intervention**	23

For peer review only - http://bmjopen.bmj.com/site/about/guidelines.xhtml

Urgent calls to paediatrician (per 1,000 patient-days)	Parshuram 2011[70]	✓		✓	✓	Bedside PEWS	Canada	1	N	N	All in-patients	Uncontrolled before-after study (prospective)	8 (3 before, 5 after)	19 (22.6)	12 (5.1)	RR = 0.23 (0.11-0.46) †	**<.0001**	**Significant decrease in rate of urgent calls to paediatrician, post intervention**	23
Code blue calls on the ward (per 1,000 admissions)	Kotsakis 2011[64]	✓	✓			Modified MAC	Canada	4	Y	N	All in-patients	Uncontrolled before-after study (prospective)	48 (24 before, 24 after)	210 (3.75)	150 (2.70)	RR = 0.71 (0.61-0.83)	**<.0001**	**Significant reduction in rate of code blue calls, post intervention**	18
Urgent calls to outreach team (per 1,000 admissions)	Duns 2014[73]	✓				Between the Flags (BTS) tool*	Australia	1	Y	Y	All in-patients	Uncontrolled before-after study (prospective)	48 (24 before, 24 after)	1,058 (39.5)	2,120 (76.0)	RR=1.92 (1.79-2.07) †	**.02**	**Significant increase in rate of urgent calls to outreach team, post intervention**	7
RRT calls (per 1,000 patient-days)	Panesar 2014[60]			✓		Modified Brighton PEWS (e)	US	1	Y	Y	All in-patients	Uncontrolled before-after study (retrospective)	42 (18 before, 24 after)	44 (3.14)	69 (4.23)	RR = 1.35 (0.92-1.96) †	.11	Non-significant trend towards increased RRT calls, post intervention	15
RRT calls (per 1,000 patient days)	Douglas 2016[72]	✓		✓	✓	Modified Brighton PEWS (b)	US	1	Y	Y	All in-patients	Uncontrolled before-after study (retrospective)	24 (12 before, 12 after)	194 (6.17)	292 (9.80)	RR = 1.59 (1.33-.1.90) †	**<.001**	**Significant increase in rate of RRT calls, post intervention**	12
Code calls (per 1,000 patient days)	Douglas 2016[72]	✓		✓	✓	Modified Brighton PEWS (b)	US	1	Y	Y	All in-patients	Uncontrolled before-after study (retrospective)	24 (12 before, 12 after)	31 (0.98)	20 (0.67)	RR = 0.68 (0.39-1.19) †	.21	No change in rate of code calls, post intervention	12
**PICU TRANSFERS**																			

For peer review only - http://bmjopen.bmj.com/site/about/guidelines.xhtml

Transfers from ward to other specialist units (per 1,000 patient-days)	Parshuram 2011[70]	✓		✓	✓	Bedside PEWS	Canada	1	N	N	All in-patients	Uncontrolled before-after study (prospective)	8 (3 before, 5 after)	5 (5.9)	19 (8.1)	RR = 1.37 (0.51-3.63) †	.04	**Significant increase in rate of transfers from ward to specialist unit, post intervention**	23
Clinical deterioration events on ward prior to transfer to specialist unit (per 1,000 patient-days)	Parshuram 2011[70]	✓		✓	✓	Bedside PEWS	Canada	1	N	N	All in-patients	Uncontrolled before-after study (prospective)	8 (3 before, 5 after)	2 (2.4)	1 (0.43)	RR = 0.18 (0.02-1.97) †	.01	**Significant reduction in rate of clinical deterioration events on ward, post intervention**	23
PICU transfers (per 1,000 admissions)	Duns 2014[73]	✓				Between the Flags (BTS) tool*	Australia	1	Y	Y	All in-patients	Uncontrolled before-after study (prospective)	48 (24 before, 24 after)	350 (13.1)	364 (13.1)	RR=1.00 (0.86-1.16) †	.98	No significant change in rate of PICU transfers, post intervention	7
Unplanned PICU transfers from ward (per 1,000 non-ICU patient-days)	Bonafide 2014[62]	✓	✓			Bedside PEWS	US	1	Y	N	All in-patients	Interrupted Time Series study (prospective)	59 (32 before, 27 after)	874 (4.54)	936 (5.25)	IRR = 0.73 (0.46–1.14)	.16	No significant difference between trend in adjusted rate of unplanned transfers post intervention and expected trend based on pre-intervention trajectory	23
Unplanned transfers to PICU from ward (per 1,000 patient-days)	Agulnik 2017[71]	✓			✓	Children's Hospital Cardiac Early Warning Score (C-CHEWS)	Guatemala	1	Y	N	Oncology unit	Uncontrolled before-after study (retrospective)	24 (12 before, 12 after)	157 (9.3)	130 (6.5)	RR = 0.70 (0.56-0.88) †	**.003**	**Significant reduction in rate of unplanned transfers to PICU, post intervention**	19

For peer review only

For peer review only - http://bmjopen.bmj.com/site/about/guidelines.xhtml

Urgent PICU admissions (per 1,000 patient-days)	Parshuram 2018[65]	✓	✓		✓	Bedside PEWS	Belgium, Ireland, Netherlands, England, Italy, Canada, New Zealand	21	Y	N	All in-patients	Cluster randomised trial (prospective)	18 (6 pre, 12 post)	Con: 652 (4.01)<br>Int: 469 (3.62)	Con: 1178 (3.83)<br>Int: 828 (3.29)	RR=0.95 (0.82-1.09)	.45	No significant difference in pre and post intervention urgent PICU admission rate between intervention and usual care hospitals	23
**PICU OUTCOMES**																			
Critical deterioration events after PICU transfer (per 1,000 non-ICU patient-days)	Bonafide 2014[62]	✓	✓			Bedside PEWS	US	1	Y	N	All in-patients	Interrupted Time Series study (prospective)	59 (32 before, 27 after)	260† (1.35)	282† (1.58)	IRR = 0.38 (0.20-0.75)	**.01**	**Significant downward trend in adjusted rate of critical deterioration events post intervention, compared to expected trend based on pre-intervention trajectory**	23
Mechanical ventilation within 1hr of unplanned PICU transfer (per unplanned transfer to PICU)	Bonafide 2014[62]	✓	✓			Bedside PEWS	US	1	Y	N	All in-patients	Interrupted Time Series study (prospective)	59 (32 before, 27 after)	45 (5.1)	42 (4.5)	RR = 0.87 (0.58-1.31) †	.51	No significant difference in unadjusted rate of mechanical ventilation within 1hr of PICU admission, post intervention	23

For peer review only - http://bmjopen.bmj.com/site/about/guidelines.xhtml

Mechanical ventilation within 12hrs of unplanned PICU transfer (per unplanned transfer to PICU)	Bonafide 2014[62]	✓	✓			Bedside PEWS	US	1	Y	N	All in-patients	Interrupted Time Series study (prospective)	59 (32 before, 27 after)	112 (12.8)	103 (11.0)	IRR = 0.17 (0.07-0.44)	**<0.001**	**Significant reduction in adjusted trend of rate of mechanical ventilation within 12hrs of PICU admission, compared to expected trend based on pre-intervention trajectory**	23
Vasopressor within 1hr of unplanned PICU transfer (per unplanned transfer to PICU)	Bonafide 2014[62]	✓	✓			Bedside PEWS	US	1	Y	N	All in-patients	Interrupted Time Series study (prospective)	59 (32 before, 27 after)	41 (4.7)	16 (1.7)	RR = 0.36 (0.21-0.64) †	**<0.001**	**Significant reduction in unadjusted rate of vasopressors within 1hr of PICU admission, post intervention**	23
Vasopressors within 12hrs of unplanned PICU transfer (per unplanned transfer to PICU)	Bonafide 2014[62]	✓	✓			Bedside PEWS	US	1	Y	N	All in-patients	Interrupted Time Series study (prospective)	59 (32 before, 27 after)	71 (8.1)	57 (6.1)	IRR = 0.20 (0.06-0.62)	**.006**	**Significant reduction in adjusted trend of rate of vasopressors within 12hrs of PICU admission, compared to expected trend based on pre-intervention trajectory**	23
Invasive ventilation in PICU (per emergency PICU admission)	Sefton 2015[69]	✓		✓	✓	Modified Bristol PEWT (a)	UK	1	Y	N	All PICU admissions	Controlled before-after study (retrospective)	24 (12 before, 12 after)	118 (75.2)	104 (62.7)	RR = 0.83 (0.72-0.97) †	**.002**	**Significant reduction in rate of PICU emergency admissions given invasive ventilation, post intervention**	16

For peer review only - http://bmjopen.bmj.com/site/about/guidelines.xhtml

Inotropes in PICU (per emergency PICU admission)	Sefton 2015[69]	✓		✓	✓	Modified Bristol PEWT (a)	UK	1	Y	N	All PICU admissions	Controlled before-after study (retrospective)	24 (12 before, 12 after)	50 (31.8)	40 (24.1)	RR = 0.76 (0.53-1.08) †	.12	Non-significant trend towards reduced rate of PICU emergency admissions given inotropes, post intervention	16
Intubation within 24hrs of PICU admission (per 1,000 patient-days)	Agulnik 2017[71]	✓			✓	Children's Hospital Cardiac Early Warning Score (C-CHEWS)	Guatemala	1	Y	N	Oncology unit	Uncontrolled before-after study (retrospective)	24 (12 before, 12 after)	11 (0.65)	18 (0.90)	RR=1.38 (0.65-2.92) †	.46	No change in rate of PICU admissions receiving intubation within 24 hours, post intervention	19
Vasopressors within 24hrs of PICU admission (per 1,000 patient-days)	Agulnik 2017[71]	✓			✓	Children's Hospital Cardiac Early Warning Score (C-CHEWS)	Guatemala	1	Y	N	Oncology unit	Uncontrolled before-after study (retrospective)	24 (12 before, 12 after)	29 (1.72)	37 (1.86)	RR=1.08 (0.66-1.75) †	.60	No change in rate of PICU admissions receiving vasopressors within 24 hours, post intervention	19
Mechanical ventilation during PICU admission (per PICU admission)	Kolovos 2018[66]	✓	✓			RRT activation criteria*	US	1	Y	N	All unplanned PICU admissions	Uncontrolled before-after study (retrospective)	78 (42 before, 36 after)	285 (25.98)	233 (22.09)	RR = 0.85 (0.73-0.99) †	**.03†**	**Significant reduction in rate of PICU admissions receiving mechanical ventilation, post intervention**	12
Intubation within 1hr of PICU admission (per PICU admission)	Kolovos 2018[66]	✓	✓			RRT activation criteria*	US	1	Y	N	All unplanned PICU admissions	Uncontrolled before-after study (retrospective)	78 (42 before, 36 after)	49 (4.47)	88 (8.34)	RR = 1.87 (1.33-2.62)	**.0003**	**Significant increase in rate of PICU admissions intubated within 1hr of admission, post intervention**	12
Significant clinical deterioration events	Parshuram 2018[65]	✓	✓		✓	Bedside PEWS	Belgium, Ireland, Netherlands,	21	Y	N	All in-patients	Cluster randomised trial (prospective)	18 (6 pre, 12 post)	Con: 144 (0.89)	Con: 259 (0.84)	RR=0.77 (0.61-0.97)	**.03**	**Significant reduction in rate of clinical deterioration**	23

For peer review only - http://bmjopen.bmj.com/site/about/guidelines.xhtml

(per 1,000 patient-days)							England, Italy, Canada, New Zealand							Int: 80 (0.62)	Int: 127 (0.50)			**events in intervention hospitals, compared to control hospitals**	

A critical deterioration event is defined as transfer to the intensive care unit (ICU) followed by non-invasive or invasive mechanical ventilation or vasopressor infusion within 12 hours[62]

*Indicates a PTTT not described or validated in the published literature

† Data calculated by research team, based on data presented in the journal article. All data calculated via https://www.medcalc.org.

PTTT, paediatric track and trigger tool; RRT, rapid response team; MET, medical emergency team; PICU, paediatric intensive care unit; RR, relative risk; OR, odds ratio; ED, emergency department

For peer review only

For peer review only - http://bmjopen.bmj.com/site/about/guidelines.xhtml

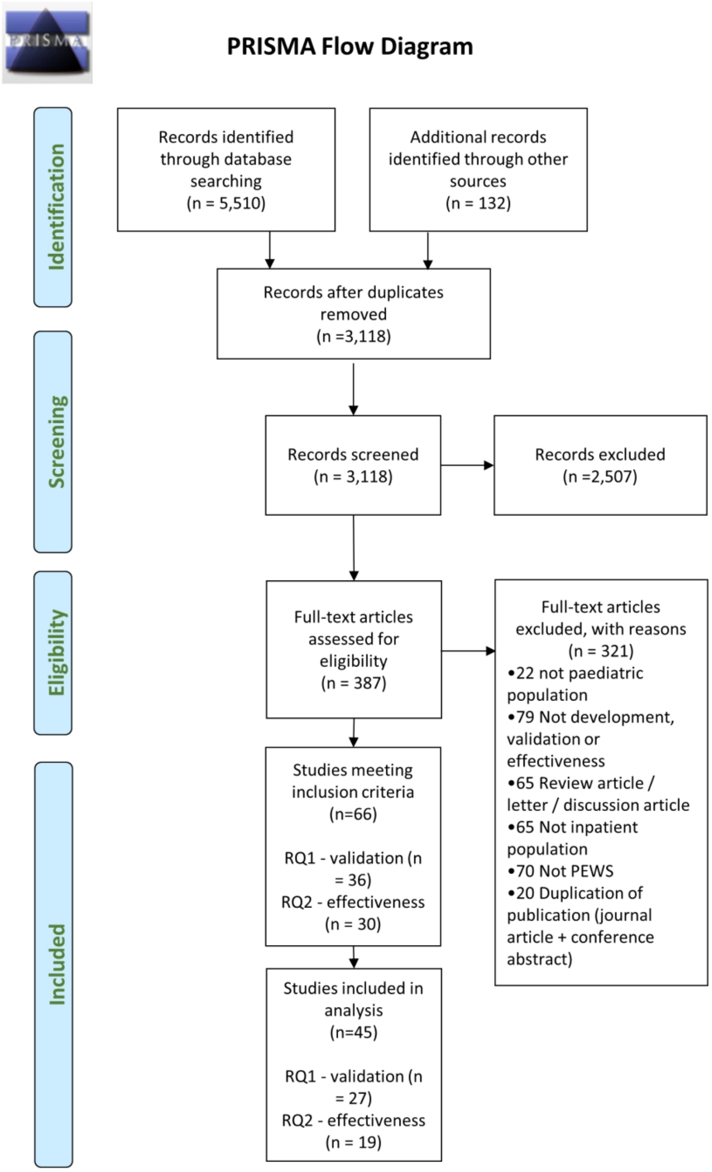

Figure 1: PRISMA flow diagram of study inclusion

190x254mm (300 x 300 DPI)

Supplementary Table 1 – Details of search strategy

Database Search

The search was across a range of databases from their inception to January 2015 then an update was carried out in September 2016 and the second update May 2018.

A preliminary search strategy was developed using a set of key papers known to the group for Ovid Medline using both text words and Medical subject headings. The search strategy was modified according to the indexing systems of the other databases.

Databases and Database platform	Original search results January 2015	Update September 2016	Update May 2018
British Nursing Index (Proquest)	19	12	25
CINAHL (Cumulative Index of Nursing and Allied Health Literature) (Ebsco)	206	17	29
Cochrane Central Register of Controlled Trials (Wiley)	43	4	30
EMBASE (OVID)	1065	206	431
HMIC (Health Management Information Centre) (OVID)	70	1	75
Medline (OVID)	943	135	328
Medline in Process (OVID)	43	69	45
Scopus (Elsevier)	747	85	234
Web of Knowledge (Science Science Citation Indexes) (Thomson Reuter)	400	82	166
**Total**	**3536** *(prior to removing duplicates and irrelevant studies)*	**611** *(prior to removing duplicates and irrelevant studies)*	**1363** *(prior to removing duplicates and irrelevant studies)*

Supplementary search

For peer review only

PUMA Search Information

Supplementary search

NB. Restricted each of the below searches by dates: 01/01/2016 – 16/05/2018

Trials Registers	Hits January 2015	Update September 2016	Update June 2018
ClinicalTrials.gov https://clinicaltrials.gov/	6	4	0
UK Clinical Trials Gateway http://www.ukctg.nihr.ac.uk/default.aspx	3 (duplicates)	5 (1 duplicate)	0
The WHO trial search portal for studies worldwide: http://apps.who.int/trialsearch	1 (duplicate)	0	0
Journal site	Hits		
Archives of Disease in Childhood http://adc.bmj.com/	14	4	7
BMJ http://www.bmj.com/theBMJ	1	0	1
BMJ Quality and safety http://qualitysafety.bmj.com/	7	4	2
JAMA Pediatrics http://archpedi.jamanetwork.com/journal.aspx	1	0	0
Journal of Critical Care http://www.jccjournal.org/	3	1	0
Journal of Pediatrics (American) http://www.jpeds.com/	1	0	2
Journal of Paediatrics and Child Health (Australian) http://onlinelibrary.wiley.com/journal/10.1111/(ISSN)1440-1754	2	2	0

For peer review only

Lancet<br>http://www.thelancet.com/	0	0	0
New England Journal of Medicine<br>http://www.nejm.org/	0	0	0
Pediatrics<br>http://pediatrics.aappublications.org/	6	2	0
Pediatric Critical Care Medicine<br>http://journals.lww.com/pccmjournal/pages/default.aspx	14	6	3
Websites and organisations	HITS		
American Society of Anesthesiologists<br>https://www.asahq.org/	1	0	0
American Academy of Pediatrics<br>http://www.aap.org/en-us/Pages/Default.aspx	1		0
Association of Anaesthetists of Great Britain and Ireland<br>http://www.aagbi.org/	0	0	0
Australian Medical Council<br>http://www.amc.org.au/	1	0	0
Royal College of Paediatrics and Child Health<br>http://www.rcpch.ac.uk/	1	0	4
Paediatric Nursing Association Europe<br>http://www.rcn.org.uk/	9		0
European Federation of Critical Care Nursing Associations<br>http://www.efccna.org/	No Search Option	No Search Option	No Search Option
Royal Australasian College of Physicians (Division of Child Health)<br>https://www.racp.edu.au/page/paed-policy	0	0	0
Royal College of Physicians (inclusive of National Clinical Guideline Centre)<br>https://www.rcplondon.ac.uk/	2	0	0
The NHS Institute for Innovation and Improvement<br>http://www.institute.nhs.uk/	4	Site cease to exist	Site cease to exist
NICE: Eyes on Evidence	4	1	1

For peer review only

https://www.evidence.nhs.uk/about-evidence-services/bulletins-and-alerts/eyes-on-evidence			
**TOTAL**	82	30	20

Total = 112

Search Strategies

BNI
"Paediatric Early Warning" OR ("pediatric early warning" OR "pediatric rapid response") OR ("paediatric rapid response" OR "Bedside paediatric early warning") OR ("Pediatric Advanced Warning Score" OR "Paediatric Advanced Warning Score")

CENTRAL
Search Name: PUMA update
Last Saved: 16/05/2018 11:39:08.703
Description:

ID	Search
#1	"early warning score*"
#2	"early warning system*"
#3	"early warning tool*"
#4	"VitalPAC Early Warning Score"
#5	"activation criteria"
#6	"Rapid Response Team"
#7	"Rapid Response system*"
#8	"Track and trigger"
#9	"trigger tools"
#10	"calling criteria"

For peer review only

#11	"Alert criteria"
#12	"Rapid Response"
#13	#1 or #2 or #3 or #4 or #5 or #6 or #7 or #8 or #9 or #10 or #11 or #12
#14	pediatric* or paediatric* or infant* or child* or baby or toddler or babies or teen* or adolescent*
#15	#13 and #14
#16	"Pediatric Early Warning"
#17	"Paediatric Early Warning"
#18	"p?ediatric alert"
#19	"Pediatric Rapid Response"
#20	"Pediatric Advanced Warning Score*"
#21	"Paediatric Advanced Warning Score*"
#22	"infant early warning"
#23	"Bedside PEWS"
#24	"Bedside paediatric early warning"
#25	#16 or #17 or #18 or #19 or #20 or #21 or #22 or #23 or #24
#26	#15 or #25 Publication Year from 2016 to 2018

CINAHL via EBSCO

	Search ID#	Search Terms
☐	S11	S7 OR S10
☐	S10	S1 AND S8
☐	S9	S2 AND S8
☐	S8	S3 AND S4
☐	S7	S5 OR S6
☐	S6	TX "infant early warning" OR TX "bedside PEWS" OR TX "Bedside paediatric early warning"
☐	S5	TX "p?ediatric early warning system" OR TX "P?ediatric Early Warning" OR TX "p?ediatric early warning score" OR TX "p?ediatric risk of mortality" OR TX "P?ediatric Rapid Response Team" OR TX "P?ediatric alert"
☐	S4	AB pediatric* or paediatric* or infant*1 or child* or baby or toddler or babies or teen* or adolescent*

For peer review only

☐	S3	TX "track-and-trigger" OR TX "VitalPAC Early Warning Score" OR TX "activation criteria". OR TX "trigger tool*" OR TX "Rapid Response" OR TX "activation criteria". OR TX "early warning" OR TX "Alert criteria" OR TX outreach N3 emergency
☐	S2	Detecting W3 deterioration
☐	S1	"early warning"

DARE
(Paediatric early warning) OR (pediatric early warning) OR (Paediatric Rapid Response) IN DARE
(early warning) OR (track-and-trigger system) OR (Rapid Response) IN DARE
(emergency team) AND (early warning) IN DARE

Embase
Database: EMBASE <1947-Present>
Search Strategy:
----
1 ("early warning" adj5 scor*).ab,ti. (568)
2 ("early warning" adj5 system* adj5 (deteriorat* or mortality or death or outcome* or harm* or safety)).ab,ti. (51)
3 "acute illness severity".mp. (38)
4 early intervention/ and ((prevent* or reduc* or improv*) adj5 (deteriorat* or mortality or death or outcome* or harm* or safety)).ab,ti. (1185)
5 ("early medical intervention" adj5 (tool* or scor* or index* or indicator* or indice* or assessment* or guide* or instrument* or criteria or parameter* or deteriorat* or mortality or death or monitor* or outcome* or harm* or safety)).ab,ti. (10)
6 *"severity of illness index"/ and ((tool* or scor* or index* or indicator* or indice* or assessment* or instrument* or criteria or parameter*) adj5 ((prevent* or reduc* or improv*) adj5 (deteriorat* or mortality or death or outcome* or harm* or safety))).ab,ti. (3)
7 exp Health Status Indicators/ and ((tool* or scor* or index* or indicator* or indice* or assessment* or instrument* or criteria or parameter*) adj3 ((prevent* or reduc* or improv*) adj3 (deteriorat* or mortality or death or outcome* or harm* or safety))).ab,ti. (7)
8 rapid response team/ (849)
9 "alarm monitor"/ and (prevent* or reduc* or improv*).mp. (245)
10 ("clinical alarm" adj5 (prevent* or reduc* or improv*)).mp. (2)
11 (outreach adj3 emergency).tw. (46)
12 VitalPAC Early Warning Score.tw. (15)
13 medical emergency team.tw. (395)
14 Rapid Response Systems.mp. (140)

For peer review only

15 ("rapid response" adj5 (prevent* or reduc* or improv*)).tw. (191)
16 ("medical device" adj3 (prevent* or reduc* or improv*)).mp. (187)
17 (((Detecting or managing) adj3 deterioration) and warning).tw. (11)
18 track-and-trigger system.tw. (24)
19 (Track adj trigger).tw. (4)
20 (Track and trigger).tw. (241)
21 trigger tools.tw. (47)
22 ("alert criteria" or "activation criteria" or "calling criteria").tw. (209)
23 SBAR technique*.mp. (5)
24 (score adj3 severity of illness).tw. (393)
25 or/1-24 (4295)
26 limit 25 to (infant <to one year> or child <unspecified age> or preschool child <1 to 6 years> or school child <7 to 12 years> or adolescent <13 to 17 years>) (533)
27 P?ediatric Early Warning.mp. (120)
28 p?ediatric alert.tw. (7)
29 p?ediatric early warning systems.mp. (4)
30 p?ediatric risk of mortality.tw. (527)
31 P?ediatric Rapid Response Team.tw. (14)
32 Point-of-Care Systems/ and ((paediatric or pediatric) adj3 (improve or identify or detect* or outcome or early or critical or emergency)).tw. (23)
33 P?ediatric Advanced Warning Score.tw. (3)
34 neonatal early warning.tw. (1)
35 infant early warning.tw. (0)
36 p?ediatric rapid response.tw. (31)
37 Bedside paediatric early warning.tw. (5)
38 Bedside PEWS.tw. (7)
39 or/27-38 (707)
40 26 or 39 (1155)
41 limit 40 to human (1065)

HMIC
Database: HMIC Health Management Information Consortium
Search Strategy:

For peer review only

1 ("early warning" adj5 scor*).ab,ti. (23)
2 ("early warning" adj5 system* adj5 (deteriorat* or mortality or death or outcome* or harm* or safety)).ab,ti. (6)
3 "acute illness severity".mp. (3)
4 "early medical intervention"/ and ((prevent* or reduc* or improv*) adj5 (deteriorat* or mortality or death or outcome* or harm* or safety)).ab,ti. (0)
5 ("early medical intervention" adj5 (tool* or scor* or index* or indicator* or indice* or assessment* or guide* or instrument* or criteria or parameter* or deteriorat* or mortality or death or monitor* or outcome* or harm* or safety)).ab,ti. (0)
6 Health Status Indicators.mp. and ((tool* or scor* or index* or indicator* or indice* or assessment* or instrument* or criteria or parameter*) adj3 ((prevent* or reduc* or improv*) adj3 (deteriorat* or mortality or death or outcome* or harm* or safety))).ab,ti. (0)
7 exp "Severity of illness index"/ and ((tool* or scor* or index* or indicator* or indice* or assessment* or instrument* or criteria or parameter*) adj5 ((prevent* or reduc* or improv*) adj5 (deteriorat* or mortality or death or outcome* or harm* or safety))).ab,ti. (0)
8 "activation criteria".ab,ti. (2)
9 exp Rapid response teams/ (39)
10 Clinical Alarms.mp. (0)
11 (outreach adj3 emergency).tw. (2)
12 VitalPAC Early Warning Score.tw. (0)
13 medical emergency team.tw. (15)
14 Rapid Response Systems.mp. (8)
15 Rapid Response Team.tw. (27)
16 ((Detecting or managing) adj3 deterioration).tw. (1)
17 track-and-trigger system.tw. (2)
18 (Track adj trigger).tw. (1)
19 (Track and trigger).tw. (8)
20 trigger tools.tw. (4)
21 Calling criteria.tw. (1)
22 Alert criteria.mp. (1)
23 Rapid response.tw. (111)
24 (score adj3 severity of illness).tw. (3)
25 or/1-24 (171)
26 (pediatric* or paediatric* or infant*1 or child* or baby or toddler or babies or teen* or adolescent*).mp. (40161)
27 25 and 26 (14)
28 p?ediatric alert.tw. (0)
29 p?ediatric early warning systems.mp. (1)
30 p?ediatric risk of mortality.tw. (4)

For peer review only

31 Pediatric Rapid Response Team.tw. (0)
32 Point-of-Care.mp. and ((paediatric or pediatric) adj3 (improve or identify or detect* or outcome or early or critical or emergency)).tw. (0)
33 Pediatric Advanced Warning Score.tw. (0)
34 neonatal early warning.tw. (0)
35 infant early warning.tw. (0)
36 paediatric rapid response.tw. (1)
37 pediatric rapid response.tw. (0)
38 Bedside paediatric early warning.tw. (0)
39 Bedside PEWS.tw. (0)
40 p?ediatric early warning.mp. (2)
41 care.mp. and ((paediatric or pediatric) adj3 (improve or identify or detect* or outcome or early or critical or emergency)).tw. [mp=title, other title, abstract, heading words] (57)
42 or/28-41 (59)
43 27 or 42 (70)

Medline
Database: Ovid MEDLINE(R) <1946 to January Week 2 2015>
Search Strategy:
----
1 ("early warning" adj5 scor*).ab,ti. (260)
2 ("early warning" adj5 system* adj5 (deteriorat* or mortality or death or outcome* or harm* or safety)).ab,ti. (24)
3 "acute illness severity".mp. (21)
4 "early medical intervention"/ and ((prevent* or reduc* or improv*) adj5 (deteriorat* or mortality or death or outcome* or harm* or safety)).ab,ti. (99)
5 ("early medical intervention" adj5 (tool* or scor* or index* or indicator* or indice* or assessment* or guide* or instrument* or criteria or parameter* or deteriorat* or mortality or death or monitor* or outcome* or harm* or safety)).ab,ti. (7)
6 exp Health Status Indicators/ and ((tool* or scor* or index* or indicator* or indice* or assessment* or instrument* or criteria or parameter*) adj3 ((prevent* or reduc* or improv*) adj3 (deteriorat* or mortality or death or outcome* or harm* or safety))).ab,ti. (166)
7 "Severity of Illness Index"/ and ((tool* or scor* or index* or indicator* or indice* or assessment* or instrument* or criteria or parameter*) adj5 ((prevent* or reduc* or improv*) adj5 (deteriorat* or mortality or death or outcome* or harm* or safety))).ab,ti. (274)
8 exp Hospitals/ and ((Detecting or managing) adj3 deterioration).tw. (2)
9 ("medical device" adj3 (prevent* or reduc* or improv*)).mp. (58)
10 ("alert criteria" or "activation criteria" or "calling criteria").tw. (121)

For peer review only

11 Hospital Rapid Response Team/ (334)
12 Clinical Alarms/ (332)
13 (outreach adj3 emergency).tw. (32)
14 VitalPAC Early Warning Score.tw. (10)
15 medical emergency team.tw. (247)
16 Rapid Response Systems.mp. (87)
17 Rapid Response Team.tw. (185)
18 (((Detecting or managing) adj3 deterioration) and warning).tw. (8)
19 track-and-trigger system.tw. (14)
20 (Track adj trigger).tw. (2)
21 (Track and trigger).tw. (137)
22 trigger tools.tw. (22)
23 SBAR technique*.mp. (3)
24 ("rapid response" adj5 (prevent* or reduc* or improv*)).tw. (117)
25 (score adj3 severity of illness).tw. (243)
26 or/1-25 (2286)
27 limit 26 to (humans and "all child (0 to 18 years)") (453)
28 P?ediatric Early Warning.mp. (38)
29 p?ediatric alert.tw. (5)
30 p?ediatric early warning systems.mp. (3)
31 p?ediatric risk of mortality.tw. (400)
32 P?ediatric Rapid Response Team.tw. (6)
33 Point-of-Care Systems/ and ((paediatric or pediatric) adj3 (improve or identify or detect* or outcome or early or critical or emergency)).tw. (79)
34 P?ediatric Advanced Warning Score.tw. (2)
35 neonatal early warning.tw. (0)
36 infant early warning.tw. (0)
37 p?ediatric rapid response.tw. (20)
38 Bedside paediatric early warning.tw. (2)
39 Bedside PEWS.tw. (2)
40 or/28-39 (542)
41 27 or 40 (943)

Scopus

For peer review only

(TITLE-ABS-KEY ("Paediatric Early Warning" OR "Pediatric Early Warning" OR "Pediatric Advanced Warning Score" OR "Paediatric Advanced Warning Score" OR "neonatal early warning" OR "infant early warning" OR "pediatric rapid response" OR "Paedatric rapid response")) OR (((TITLE-ABS-KEY ("early warning" W/5 scor*)) OR (TITLE-ABS-KEY ("Rapid Response")) OR (TITLE-ABS-KEY ("track-and-trigger system")) OR (TITLE-ABS-KEY ("track and trigger")) OR (TITLE-ABS-KEY ("trigger tool*")) OR (TITLE-ABS-KEY ("alert criteria")) OR (TITLE-ABS-KEY ("activation criteria")) OR (TITLE-ABS-KEY ("VitalPAC Early Warning Score"))) AND (TITLE-ABS-KEY (pediatric* OR paediatric* OR infant* OR child* OR baby OR toddler OR babies OR teen* OR adolescent*))) AND (LIMIT-TO (SUBJAREA , "MEDI") OR LIMIT-TO (SUBJAREA , "NURS") OR LIMIT-TO (SUBJAREA , "NEUR"))

Web of Science

# 19	400	#17 OR #1<br>**Refined by:** [excluding] **WEB OF SCIENCE CATEGORIES:** (PARASITOLOGY OR PUBLIC ENVIRONMENTAL OCCUPATIONAL HEALTH OR BIOCHEMISTRY MOLECULAR BIOLOGY OR OPTICS OR HEALTH CARE SCIENCES SERVICES OR MYCOLOGY OR MANAGEMENT OR LINGUISTICS OR INSTRUMENTS INSTRUMENTATION OR MICROBIOLOGY OR INFORMATION SCIENCE LIBRARY SCIENCE OR MATHEMATICAL COMPUTATIONAL BIOLOGY OR GERIATRICS GERONTOLOGY OR ENGINEERING BIOMEDICAL OR FOOD SCIENCE TECHNOLOGY OR ENVIRONMENTAL STUDIES OR ENGINEERING ENVIRONMENTAL OR ENGINEERING ELECTRICAL ELECTRONIC OR HEALTH POLICY SERVICES OR TOXICOLOGY OR EDUCATION EDUCATIONAL RESEARCH OR NUTRITION DIETETICS OR SUBSTANCE ABUSE OR ECONOMICS OR MEDICINE RESEARCH EXPERIMENTAL OR STATISTICS PROBABILITY OR DEVELOPMENTAL BIOLOGY OR MEDICAL INFORMATICS OR SOCIOLOGY OR DENTISTRY ORAL SURGERY MEDICINE OR PSYCHOLOGY EXPERIMENTAL OR COMPUTER SCIENCE ARTIFICIAL INTELLIGENCE OR METEOROLOGY ATMOSPHERIC SCIENCES OR CHEMISTRY ANALYTICAL OR MEDICAL LABORATORY TECHNOLOGY OR CELL BIOLOGY OR DEMOGRAPHY OR BUSINESS FINANCE OR COMPUTER SCIENCE INTERDISCIPLINARY APPLICATIONS OR AUDIOLOGY SPEECH LANGUAGE PATHOLOGY OR PSYCHOLOGY DEVELOPMENTAL OR COMPUTER SCIENCE INFORMATION SYSTEMS OR PLANNING DEVELOPMENT)<br>Indexes=SCI-EXPANDED, SSCI, CPCI-S, CPCI-SSH Timespan=1900-2015
# 18	499	#17 OR #1<br>Indexes=SCI-EXPANDED, SSCI, CPCI-S, CPCI-SSH Timespan=1900-2015
#	487	#16 AND #15

For peer review only

17		Indexes=SCI-EXPANDED, SSCI, CPCI-S, CPCI-SSH Timespan=1900-2015
# 16	8,044	#14 OR #13 OR #12 OR #11 OR #10 OR #9 OR #8 OR #7 OR #6 OR #5 OR #4 OR #3 OR #2<br>Indexes=SCI-EXPANDED, SSCI, CPCI-S, CPCI-SSH Timespan=1900-2015
# 15	1,689,232	**TOPIC:** ((pediatric* OR paediatric* OR infant* OR child* OR baby OR toddler OR babies OR teen* OR adolescent*))<br>Indexes=SCI-EXPANDED, SSCI, CPCI-S, CPCI-SSH Timespan=1900-2015
# 14	130	**TOPIC:** ("Severity of Illness Index" and ((tool* or scor* or index* or indicator* or indice* or assessment* or instrument* or criteria or parameter*) SAME ((prevent* or reduc* or improv*) SAME (deteriorat* or mortality or death or outcome* or harm* or safety))))<br>Indexes=SCI-EXPANDED, SSCI, CPCI-S, CPCI-SSH Timespan=1900-2015
# 13	63	**TOPIC:** (("early medical intervention" SAME (tool* or scor* or index* or indicator* or indice* or assessment* or guide* or instrument* or criteria or parameter* or deteriorat* or mortality or death or monitor* or outcome* or harm* or safety)))<br>Indexes=SCI-EXPANDED, SSCI, CPCI-S, CPCI-SSH Timespan=1900-2015
# 12	28	**TOPIC:** ("early medical intervention" and ((prevent* or reduc* or improv*) SAME (deteriorat* or mortality or death or outcome* or harm* or safety)))<br>Indexes=SCI-EXPANDED, SSCI, CPCI-S, CPCI-SSH Timespan=1900-2015
# 11	1,206	**TOPIC:** ("early warning" SAME system* SAME (deteriorat* or mortality or death or outcome* or harm* or safety))<br>Indexes=SCI-EXPANDED, SSCI, CPCI-S, CPCI-SSH Timespan=1900-2015
# 10	2	**TOPIC:** ("SBAR technique")<br>Indexes=SCI-EXPANDED, SSCI, CPCI-S, CPCI-SSH Timespan=1900-2015
# 9	7	**TOPIC:** ("VitalPAC Early Warning Score")<br>Indexes=SCI-EXPANDED, SSCI, CPCI-S, CPCI-SSH Timespan=1900-2015
# 8	123	**TOPIC:** ("activation criteria")<br>Indexes=SCI-EXPANDED, SSCI, CPCI-S, CPCI-SSH Timespan=1900-2015
# 7	16	TS=("alert criteria")<br>Indexes=SCI-EXPANDED, SSCI, CPCI-S, CPCI-SSH Timespan=1900-2015
# 6	159	TS=("trigger tool*")<br>Indexes=SCI-EXPANDED, SSCI, CPCI-S, CPCI-SSH Timespan=1900-2015
#	45	TS=("track and trigger")

For peer review only

5 Indexes=SCI-EXPANDED, SSCI, CPCI-S, CPCI-SSH Timespan=1900-2015

15 TS=("track-and-trigger system")

4 Indexes=SCI-EXPANDED, SSCI, CPCI-S, CPCI-SSH Timespan=1900-2015

6,100 TS=("Rapid Response")

3 Indexes=SCI-EXPANDED, SSCI, CPCI-S, CPCI-SSH Timespan=1900-2015

604 TS=("early warning" SAME scor*)

2 Indexes=SCI-EXPANDED, SSCI, CPCI-S, CPCI-SSH Timespan=1900-2015

88 TS=("Paediatric Early Warning" OR "Pediatric Early Warning" OR "Pediatric Advanced Warning Score"

1 OR "Paediatric Advanced Warning Score" OR "neonatal early warning" OR "infant early warning" OR "pediatric rapid response" OR "Paedatric rapid response")

Indexes=SCI-EXPANDED, SSCI, CPCI-S, CPCI-SSH Timespan=1900-2015

PUMA Supplementary searches

Search terms to use:

"Pediatric Early warning"
"Paediatric Early warning"
“Pediatric Rapid Response Team”
“Paediatric Rapid Response Team”
PEWS
“Paediatric trigger tools”
“Pediatric trigger tools”

For peer review only

Supplementary Table 2 - PICOS criteria for inclusion of studies

Question 1 – development / validation studies

Parameter	Inclusion criteria	Exclusion criteria
Patients	Children aged 0-18 who are in-patients in a hospital	Adult patients; children in emergency departments or neonatal unit
Intervention	Development or validation of a PTTT	Acuity or triage tools, tools developed for use in emergency departments
Comparator	Not applicable	
Outcomes	Mortality and critical events including: arrests, code calls, transfer to higher level of care (e.g., ICU/HDU), senior review, RRT/MET activation	
Study design	Chart or case reviews; cohort studies; case-control studies, observational studies	Reviews, editorials or opinion pieces

Question 2 – effectiveness studies

Parameter	Inclusion criteria	Exclusion criteria
Patients	Children aged 0-18 who are in-patients in a hospital	Adult patients<br>Children in emergency departments or neonatal unit
Intervention	Implementation of any 'paediatric early warning system' intervention (with or without a PTTT) – including implementing a new PTTT, RRT/MET implementation, educational initiatives or communications tools aimed at improving identification of deteriorating in-patients	Acuity or triage tools, tools developed for use in emergency departments, interventions whose purpose was not identification of deteriorating in-patients
Comparator	Not applicable	
Outcomes	Mortality and critical events including: arrests, code calls, transfer to higher level of care (e.g., ICU/HDU), senior review, RRT/MET activation	
Study design	Randomised controlled trials, non-randomised controlled trials, before-after studies (controlled or uncontrolled); interrupted time series studies	Reviews, editorials or opinion pieces

For peer review only

Supplementary Table 3 – Template Quality Assessment Forms

QUALITY ASSESSMENT FOR DEVELOPMENT AND VALIDATION STUDIES

Criteria		Yes (2)	Partial (1)	No (0)	N/A	Score
1	Is the hypothesis / aim / objective of the study clearly described?	Easily identified in introduction / method.	Vague / incomplete or found in other parts of paper (than introduction/method)	Aim / Objective no reported		
2	Was the score developed comprehensively?	Evidence base / Expert opinion / Delphi method	Decided within research team	No info / unclear		
3	Are the characteristics of the patients in the study clearly described?	Reproducible criteria used to categorise participants	Poorly define criteria / incomplete information	No baseline / demographic info		
4	Is the study design well described and appropriate?	Well described, easy to find in paper	Design not clearly described / design only partially answers the question	Design poorly described or does not answer study question		
5	Are the study sample representative of the intended population?	A full description of the target population is given with the sample selected in a non-biased manner	Sample selected from a known population however, selection strategy likely introduces bias but not enough to seriously distort results	Sample recruited from an unknown population in an opportunistic fashion		
6	Are population characteristics controlled for and adequately described?	Appropriate control at design/analysis stage	Incomplete control/description or not considered but unlikely to seriously influence results	Not controlled for and likely to seriously influence results		
7	Was compliance/use of the PEWS reliable?	Compliance / use was well described and reliably implemented	Compliance / use was not well described or not reliably implemented	Compliance / use was not reported		
8	Was consideration given for data collected at different times / sites	Well described reason why data was collected at different time points	Data was collected at different times due to specific opportunity	No explanation for data collection at different time points	Data was collected at the same time point	
9	Are the main findings clearly described?	Simple outcome data reported for all major findings	Incomplete or inappropriate descriptive statistics	No/inadequate descriptive statistics		
10	Are methods of analysis adequately described and appropriate?	Described and appropriate	Not reported but probably appropriate or some tests appropriate, some not	Methods not described and cannot be determined		
11	Are the conclusions supported by the results	All conclusions supported by data	Some of the major conclusions are supported by the data; some are not or speculative interpretations are not indicated as such	None/few of major conclusions supported by the data		
12	How was missing data handled	Missing data was reported and handled appropriately	Missing data was reported but unable to determine how it was handled or it wasn't handled appropriately	Missing data was not reported	No missing data	
					**Total**	

MAX. Score: 24

QUALITY ASSESSMENT FOR EFFECTIVENESS STUDIES

Criteria		Yes (2)	Partial (1)	No (0)	N/A	Score
1	Is the hypothesis / aim / objective of the study clearly described?	Easily identified in introduction / method.	Vague / incomplete or found in other parts of paper (than introduction/method)	Aim / Objective no reported		
2	Was the score developed comprehensively?	Evidence base / Expert opinion / Delphi method	Decided within research team	No info / unclear		
3	Are the characteristics of the patients in the study clearly described?	Reproducible criteria used to categorise participants	Poorly define criteria / incomplete information	No baseline / demographic info		
4	Is the study design well described and appropriate?	Well described, easy to find in paper	Design not clearly described / design only partially answers the question	Design poorly described or does not answer study question		
5	Are the study sample representative of the intended population?	A full description of the target population is given with the sample selected in a non-biased manner	Sample selected from a known population however, selection strategy likely introduces bias but not enough to seriously distort results	Sample recruited from an unknown population in an opportunistic fashion		
6	Was the PEWS well implemented?	Implementation was well reported and appropriately applied	Implementation was not well reported or not appropriate	No info / unclear		
7	Are population characteristics controlled for and adequately described?	Appropriate control at design/analysis stage	Incomplete control/description or not considered but unlikely to seriously influence results	Not controlled for and likely to seriously influence results		
8	Was compliance/use of the PEWS reliable?	Compliance / use was well described and reliably implemented	Compliance / use was not well described or not reliably implemented	Compliance / use was not reported		
9	Was consideration given for data collected at different times / sites	Well described reason why data was collected at different time points	Data was collected at different times due to specific opportunity	No explanation for data collection at different time points	Data was collected at the same time point	
10	Are the main findings clearly described?	Simple outcome data reported for all major findings	Incomplete or inappropriate descriptive statistics	No/inadequate descriptive statistics		
11	Are methods of analysis adequately described and appropriate?	Described and appropriate	Not reported but probably appropriate or some tests appropriate, some not	Methods not described and cannot be determined		
12	Are the conclusions supported by the results	All conclusions supported by data	Some of the major conclusions are supported by the data; some are not or speculative interpretations are not indicated as such	None/few of major conclusions supported by the data		
13	How was missing data handled	Missing data was reported and handled appropriately	Missing data was reported but unable to determine how it was handled or it wasn't handled appropriately	Missing data was not reported	No missing data	
					**Total**	

MAX. Score: 26

Supplementary Table 4 –Validation papers excluded from analysis

PTTT	Refs	Country	Study population	Study design	Number of centres	PTTT used in practice?	Internal / external validation study?	Outcome measures	Sample size	Score or trigger?	Study overview and reason for exclusion from validation results	Quality score (max = 24)
Modified Brighton PEWS (a)	Garlick 2013[20]	US	All in-patients (MET calls only)	Case-control study (retrospective)	1	N	Ext	Transfer to PICU	267 (116 cases)	S	Describes review of MET calls (n=267) to evaluate predictive ability of Modified Brighton PEWS tool for identifying children requiring transfer to PICU (n=116). Results presented in terms of association between PEWS and odds of transfer to higher level of care – no evaluation of performance characteristics such as AUROC, sensitivity or specificity.	8
	Medar 2015[21]	Unclear	RRT calls only	Chart review (retrospective)	1	NR	Ext	RRT call	61	S	Describes retrospective review of RRT calls (n=61) to evaluate Modified Brighton PEWS at time of admission and time of RRT call. Report higher median PEWS score for patients at time of RRT call compared to admission. No evaluation of performance characteristics such as AUROC, sensitivity or specificity.	6
Texas Children's Hospital (TCH) PAWS	Bell 2013[22]	US	General medical ward & two specialist units	Chart review (retrospective)	1	Y	Int	Other validated scales (e.g., Glasgow Coma Scale)	150	S	Describes development and implementation of the TCH PAWS tool in three wards of a specialist paediatric unit in the US. TCH PAWS amended locally from the Brighton PEWS. Reports on internal reliability (correlation coefficients between 3 categories of the score) and inter-rater reliability of scoring among nurses. Also compares scores on sub-categories to other measures, e.g., the Behavioural sub-score is compared to the Glasgow Coma Scale. No evaluation of performance characteristics such as AUROC, sensitivity or specificity.	12
Cardiac Children's Hospital Early Warning Score (C-CHEWS)	McLellan 2013[23]	US	Cardiac unit	Tool development	1	Y	Int	Cardiac ICU transfer	27	S	Describes the development and implementation of a modified version of the Children's Hospital Early Warning score for cardiac patients. Results focus on tool modification and implementation challenges – no evaluation of performances characteristics such as AUROC, sensitivity or specificity. Validation of the tool described in a separate paper.	9
Burn-specific PEWS	Rahman 2014[24]	US	Specialist burn unit	Chart review (retrospective)	1	Y	Int	Burn injuries	50	S	Conference abstract only. Describes development and implementation of a modified version of the Brighton PEWS, for use with in-patients with burn injuries. Analysis of 50 randomly selected charts – results focus on compliance with scoring and relationship between PTTT score and extent of burn injuries. No evaluation of performance characteristics such as AUROC, sensitivity or specificity.	13

Bedside PEWS	Hopkins 2013[25]	US	All in-patients (code blue and RRT calls only)	Chart review (retrospective)	1	N	Ext	PICU transfer and critical intervention in PICU among RRT and code calls	113 (64 cases)	S	Conference abstract only. Describes retrospective chart review of code blue and RRT calls over a year – Bedside PEWS scores calculated and comparisons drawn between patients eventually transferred to PICU and those who stayed on ward. Preliminary analysis given in terms of mean PEWS scores for different groups – no evaluation of performance characteristics such as AUROC, sensitivity or specificity.	6
	Gawronski 2013[26]	Italy	Bone marrow transplant unit	Case-control study (retrospective)	1	N	Ext	Urgent PICU transfer, PICU consult or death	21 (11 cases)	S	Conference abstract only. Describes case-control study evaluating Bedside PEWS in an Italian bone marrow transplant unit, in relation to urgent PICU transfers or consultations. Preliminary analysis only – comparison of mean PTTT scores for cases and controls. No evaluation of performance characteristics such as AUROC, sensitivity or specificity.	6
Bristol PEWT	Haines 2006[12]	UK	All in-patients	Chart review (retrospective)	1	Y	Int	Transfer to PICU or HDU	360 (180 cases)	T	Describes development and piloting of the Bristol PEWT in a UK tertiary centre. Only included children who would have triggered the pilot version of the tool (n=360) and then identified PICU or HDU transfers from this population. Paper presents specificity and sensitivity outcomes but they are incorrectly calculated, so results not included in analysis.	9
Modified Bristol PEWT (a)	Sefton 2014[27]	UK	All in-patients	Chart review (retrospective)	1	Y	Int	Transfer to PICU, cardiac / respiratory arrest or unexpected death	Unclear	T	Conference abstract only. Describes a retrospective review of 5 years of data from locally implemented PTTT in a UK tertiary centre, presenting a multiple regression model identifying seven components (including age) most strongly associated with subsequent adverse event if triggered. Of the six clinical elements, all were associated with increased odds of an adverse event, except nurse concern which was significantly associated with decreased odds of an adverse event. No evaluation of overall PTTT performance characteristics such as AUROC, sensitivity or specificity.	10

PTTT names refer to those described in Table 2

All studies conducted in a specialist / tertiary centre.

Studies classified as internal validation if the setting for the study was the same hospital and same research team as those who developed the score. Studies classified as external validation if the score was tested in a different centre and by a different research team to those who developed it.

PTTT, paediatric track and trigger tool; S, score; T, trigger; AUROC, area under the receiver operating characteristic curve; PPV, positive predictive value, NPV, negative predictive value; PICU, paediatric intensive care unit; PHDU, paediatric high-dependency unit; RRT, rapid response team; HFNC, high flow nasal cannula; UK, United Kingdom; US, United States; Int, Internal validation; Ext, external validation

For peer review only

Supplementary Table 5 – Effectiveness papers excluded from analysis

Reference	Intervention				PTTT	Country	Number of centres	Specialist unit?	Existing RRT / MET?	Population	Study design	Study duration in months (before & after intervention)	Description and reason for excluding from analysis	Quality score (max = 26)
	Implemented a new PTTT	Implemented new RRT / MET	Modified escalation process	Staff training / education										
Mistry 2006[51]	✓	✓		✓	PRRT activation criteria*	US	1	Y	N	All in-patients	Uncontrolled before-after study (prospective)	11 (6 before, 5 after)	Describes implementation of a PRRT with calling criteria (not defined). Looked at impact on mortality, cardiac arrests and PICU outcomes among PICU transfers. Reports absolute decreases in numbers of deaths and arrests post-intervention, but no denominator data provided or further statistical details given.	3
Demmel 2010[52]	✓				Modified Brighton PEWS (e)	US	1	Y	Y	Haematology / oncology patients	Uncontrolled before-after study (prospective)	Unclear (unclear, 8 after)	Implemented a locally modified version of the Brighton PEWS in a specialist haematology / oncology unit. Discusses challenges in the development and implementation of the tool. Refers to number of days between cardiopulmonary arrests being 299 immediately before implementation, and 1,053 days eight months after implementation – however, no denominator data or further statistical details given.	8
Sandhu 2010[53]		✓			Unclear	UK	1	Y	N	Unclear	Uncontrolled before-after study (retrospective)	Unclear (unclear, 3 months)	Conference abstract only. Reported implementing an 'outreach response team' alongside an existing 'paediatric early warning tool' (unclear which tool) in a UK tertiary centre. Reference to comparable triggering rate of PTTT before (28% of patients) and after (28% of patients) piloting the outreach team, and 2 arrests before piloting, and 0 after – but no denominator data or further statistical details given.	8
Randhawa 2011[54]	✓		✓	✓	Brighton PEWS	US	1	Y	Y	All in-patients	Uncontrolled before-after study (prospective)	Unclear	Describes implementation of the Brighton PEWS in a specialist paediatric centre. Details various cycles of change during implementation of the tool across different wards, and efforts at staff education. Reports reduction in rate of cardiopulmonary arrests post-intervention, but no absolute numbers, denominator data or further statistical details given.	12

Camacho 2011[55]	✓				Modified Brighton PEWS (a) †	US	1	Y	NR	Cardiac and renal patients	Uncontrolled before-after study (prospective)	8 (3 before, 5 after)	Conference abstract only. Reported piloting and modifying Tucker's modified Brighton PEWS for specialist cardiac and renal population. Unclear if RRT/MET in place. Referred to there being 5 code calls in the quarter (3 months) before implementation, and 0 in the following 5 months. However, no denominator data or further statistical details given.	8
Heyden 2012[56]	✓	✓			PRRT activation criteria*	US	1	Y	N	All in-patients	Uncontrolled before-after study (retrospective)	72 (24 before, 48 after)	Conference abstract only. Describes implementation of an RRT in a US tertiary centre, with an associated 'broad calling criteria' (limited details given). Reports number of cardiac arrests on ward and PICU before and after intervention, and refers to increase in RRT calls over time. No denominator data or further statistical details given.	7
Somberg 2013	✓	✓			Unclear	US	1	N	N	All in-patients	Uncontrolled before-after study (unclear)	Unclear	Conference abstract only. Reported developing and implementing a PTTT (tool not named) and RRT for a paediatric unit in a community hospital. Reference to no intubation or code calls since intervention, but no pre-intervention comparison, time frames, denominator data or further statistical details given.	2
Norville 2013[57]	✓				Texas Children's Hospital (TCH) Paediatric Advanced Warning Score (PAWS)†	US	1	Y	Y	Bone marrow transplant patients	Uncontrolled before-after study (unclear)	23 (12 before, 11 after)	Conference abstract only. Describes implementation of TCH PAWS, with amended algorithm for specialist bone marrow transplant unit. Looked at impact on code calls and RRT calls – refers to 3 code calls and 18 RRT calls pre-intervention, compared to 0 codes and 25 RRT calls post-intervention. No denominator data or further statistical details given.	5
Ambati 2014[58]				✓	Not applicable	US	1	Y	Y	Unclear	Uncontrolled before-after study (unclear)	48 (12 before, 36 after)	Conference abstract only. Reported effect of implementing a "simulation based curriculum" for clinical staff on subsequent RRT utilisation. Reference to increase in RRT calls year on year post implementation, but no denominator data or further statistical details given.	3
Ocholi 2014[59]	✓				Bedside PEWS	UK	1	Y	N	Unclear	Uncontrolled before-after study (unclear)	12 months (6 before, 6 after)	Conference abstract only. Describes implementation of Bedside PEWS in a UK tertiary centre. Looked at impact of intervention on ward outcomes and outcomes of children transferred to PICU. Reference to impact of tool on number of 'adverse incidents' (not defined) on the ward and median length of stay in PICU among PICU transfers, but no denominator data or further statistical details given.	6
Fenix 2016[39]	✓			✓	Unclear	US	1	Y	NR	Two general paediatric wards	Uncontrolled before-after study (retrospective)	46 months (16 before, 30 after)	Conference abstract only. Describes implementation of a 'Situational Awareness' tool, with integrated PTTT (unclear which tool) in a tertiary centre. Retrospective review of rates of Critical Deterioration (CD) events on two of seven general paediatric wards. Reports a significant decrease in trend and trajectory of CD events post-implementation, but no event numbers, denominator data or further statistical details given.	6

For peer review only

PTTT names refer to those described in Table 2

* Indicates PTTT not fully described or validated in the published literature

† PTTT modified by local team, but exact modifications not described

PTTT, paediatric track and trigger tool; RRT, rapid response team; MET, medical emergency team; PICU, paediatric intensive care unit; US, United States; UK, United Kingdom

For peer review only

BMJ Open

Validity and effectiveness of paediatric early warning systems and track and trigger tools for identifying and reducing clinical deterioration in hospitalised children: a systematic review

Journal:	*BMJ Open*
Manuscript ID	bmjopen-2018-022105.R2
Article Type:	Research
Date Submitted by the Author:	23-Jan-2019
Complete List of Authors:	Trubey, Rob; Cardiff University, Centre for Trials Research<br>Huang, Chao; Cardiff University, Centre for Trials Research<br>Lugg-Widger, Fiona; Cardiff University, Centre for Trials Research<br>Hood, Kerenza; Cardiff University, Centre for Trials Research<br>Allen, Davina; Cardiff University, School of Healthcare Sciences<br>Edwards, Dawn; Morriston Hospital, Department of Paediatrics<br>Lacy, David; Wirral Unviersity Teaching Hospital<br>Lloyd, Amy; Cardiff University, Centre for Trials Research<br>Mann, Mala; Cardiff University, University Library Services<br>Mason, Brendan; Public Health Wales<br>Oliver, Alison; Noah's Ark Children's Hospital for Wales, Department of Paediatric Intensive Care<br>Roland, Damian; Paediatric Emergency Medicine Leicester Academic (PEMLA) Group, Emergency Department<br>Sefton, G.; Alder Hey Childrens NHS Fdn Trust,<br>Skone, Richard; Noah's Ark Children's Hospital for Wales, Department of Paediatric Intensive Care<br>Thomas-Jones, Emma; Cardiff University, Centre for Trials Research<br>Tume, Lyvonne N.; University of the West of England Bristol, Faculty of Health and Applied Sciences (HAS)<br>Powell, Colin; Children's Hospital for Wales, Paediatrics; Sidra Medical and Research Center, Department of Pediatric Emergency Medicine
**Primary Subject Heading**:	Paediatrics
Secondary Subject Heading:	Paediatrics, Nursing, Intensive care, Health policy, Diagnostics
Keywords:	Quality in health care < HEALTH SERVICES ADMINISTRATION & MANAGEMENT, Paediatric intensive & critical care < INTENSIVE & CRITICAL CARE, PAEDIATRICS

SCHOLARONE™ Manuscripts

Validity and effectiveness of paediatric early warning systems and track and trigger tools for identifying and reducing clinical deterioration in hospitalised children: a systematic review

Authors: Rob Trubey[1†], Chao Huang[1], Fiona Lugg-Widger[1], Kerenza Hood[1], Davina Allen[2], Dawn Edwards[3], David Lacy[4], Amy Lloyd[1], Mala Mann[5], Brendan Mason[6], Alison Oliver[7], Damian Roland[8], Gerri Sefton[9], Richard Skone[7], Emma Thomas-Jones[1], Lyvonne Tume[9], Colin Powell[10,11]

1 Centre for Trials Research, Cardiff University, Heath Park, Cardiff, UK
2 School of Healthcare Sciences, Cardiff University, Eastgate House, Cardiff, UK
3 Department of Paediatrics, Morriston Hospital, Swansea, UK
4 Wirral University Teaching Hospital, Wirral, UK
5 Specialist Unit for Review Evidence (SURE), Cardiff University, Heath Park, Cardiff, UK
6 Public Health Wales, Cardiff, UK
7 Department of Paediatric Intensive Care, Children's Hospital for Wales, Cardiff, UK
8 Paediatric Emergency Medicine Leicester Academic (PEMLA) Group, Emergency Department, Infirmary Square, Leicester, UK
9 Paediatric Intensive Care Unit, Alder Hey Children's NHS Foundation Trust, Liverpool, UK
10 Department of Child Health, Children's Hospital for Wales, Division of Population Medicine School of Medicine, Cardiff University, Cardiff, UK,
11 Department of Pediatric Emergency Medicine, Sidra Medicine, Doha, Qatar

Corresponding author:
Dr. Rob Trubey, Centre for Trials Research, Cardiff University, Heath Park, Cardiff, UK
trubeyrj@cf.ac.uk

Keywords: PEWS, track and trigger scores, early warning scores, clinical deterioration, children, systematic review

For peer review only - http://bmjopen.bmj.com/site/about/guidelines.xhtml

ABSTRACT

Objective

To assess (1) how well validated existing paediatric track and trigger tools (PTTT) are for predicting adverse outcomes in hospitalised children, and (2) how effective broader paediatric early warning systems are at reducing adverse outcomes in hospitalised children.

Design

Systematic review.

Data sources

British Nursing Index, CINAHL, Cochrane Central Register of Controlled Trials, Database of Abstracts of Reviews of Effectiveness, EMBASE, HMIC, Medline, Medline in Process, Scopus and Web of Knowledge searched through May 2018.

Eligibility criteria

We included (1) papers reporting on the development or validation of a PTTT or (2) the implementation of a broader early warning system in paediatric units (age 0-18), where adverse outcome metrics were reported. Several study designs were considered. PROSPERO registration number CRD42015015326.

Data extraction and synthesis

Data extraction was conducted by two independent reviewers using template forms. Studies were quality assessed using a modified Downs and Black rating scale.

Results

36 validation studies and 30 effectiveness studies were included, with 27 unique PTTT identified. Validation studies were largely retrospective case-control studies or chart reviews, while effectiveness studies were predominantly uncontrolled before-after studies. Metrics of adverse outcomes varied considerably. Some PTTT demonstrated good diagnostic accuracy in retrospective case-control studies (primarily for predicting PICU transfers) but positive predictive value was consistently low, suggesting potential for alarm fatigue. A small number of effectiveness studies reported significant decreases in mortality, arrests or code calls, but

For peer review only

were limited by methodological concerns. Overall, there was limited evidence of paediatric early warning system interventions leading to reductions in deterioration.

Conclusion

There are several fundamental methodological limitations in the PTTT literature, and the predominance of single-site studies carried out in specialist centres greatly limits generalisability. With limited evidence of effectiveness, calls to make PTTT mandatory across all paediatric units are not supported by the evidence base.

For peer review only

For peer review only - http://bmjopen.bmj.com/site/about/guidelines.xhtml

STRENGTHS AND LIMITATIONS OF THIS STUDY

- Paediatric early warning systems and paediatric track and trigger tools (PTTT) are increasingly used by paediatric units across Europe, North America, Australia and elsewhere – this study is a timely review of the evidence for their validity and effectiveness
- A comprehensive search was carried out across multiple databases and included published as well as grey literature
- The review highlights methodological weaknesses and gaps in the current evidence base and makes suggestions for future research
- Heterogeneity in study populations, study designs and outcome measures make it difficult to compare and synthesise findings across the wide range of early warning systems and PTTT being used in practice
- The review is limited in scope to quantitative validation and effectiveness studies, so must be considered alongside wider literature reflecting on potential secondary benefits of early warning systems and PTTT for communication, teamwork and empowerment

For peer review only

For peer review only - http://bmjopen.bmj.com/site/about/guidelines.xhtml

BACKGROUND

Failure to recognise and respond to clinical deterioration in hospitalised children is a major safety concern in healthcare. The underlying causes of this problem are clearly multi-factorial[1–3] but paediatric 'early warning systems' have been strongly advocated as one approach to improving recognition of deterioration in paediatric units[1,2,4].

A paediatric 'early warning system' can be considered any patient safety initiative or programme which aims to monitor, detect and respond to signs of deterioration in hospitalised children in order to avert adverse outcomes and premature death. Such systems are often multi-faceted and may include the use of rapid response teams (RRT) or medical emergency teams (MET), education or training to improve clinical staff's ability to identify deterioration or strategies aimed at improving staff communication and situational awareness.

An increasingly commonplace paediatric 'early warning system' initiative is the use of a 'track and trigger tool': these tools, also commonly used in adult care, provide a formal framework for evaluating routine physiological, clinical and observational data for early indicators of patient deterioration. They are typically integrated into routine observation charts or electronic health records and compare patient observations to pre-defined 'normal' thresholds. When one or more observation is considered abnormal, staff are directed to various clinical actions, including but not limited to altered frequency of observations, review by senior staff or more appropriate treatment or management. Tools may be paper based or electronic and monitoring may be automated or manually undertaken by staff.

These tools have been referred to in the literature using a number of different terms: paediatric early warning scores (PEWS); paediatric early warning tools (PEWT), track and trigger tools (TTT) and many others. Here, we refer to the tools themselves using the term 'paediatric track and trigger tools' (PTTT). A variety of PTTT have been developed, typically by teams based in specialist paediatric centres and often used as a means of triggering a dedicated response team. Their advocacy has recently led to widespread uptake across a variety of different paediatric units, including many non-specialist centres where patient populations and resources may differ. In the United Kingdom (UK), a recent cross-sectional survey found that 85% of paediatric units were using some form of PTTT, most of which were non-specialist centres without a dedicated response team[5]. Despite their widespread use, recent reviews have questioned the evidence-base for their effectiveness in improving patient outcomes [6,7]. The current review aimed to build on this work, assessing in depth the evidence

For peer review only - http://bmjopen.bmj.com/site/about/guidelines.xhtml

base for both the validity of PTTT for predicting in-patient deterioration and the effectiveness of broader 'early warning systems' at reducing instances of mortality and morbidity in paediatric settings:

- Question 1: How well validated are existing paediatric track and trigger tools (PTTT) and their component parts for predicting in-patient deterioration?
- Question 2: How effective are paediatric early warning systems (with or without a PTTT) at reducing mortality and critical events?

For peer review only

For peer review only - http://bmjopen.bmj.com/site/about/guidelines.xhtml

METHODS

This systematic review is reported in accordance with the Preferred Reporting Items for Systematic Review and Meta-Analyses (PRISMA) guidelines[8]. Our review protocol is registered with the PROSPERO database CRD42015015326.

Search strategy

A comprehensive search was conducted across a range of databases to identify relevant studies in the English language. Published and unpublished literature was considered where publicly available, as were studies in press. The following databases were searched through May 2018: British Nursing Index, CINAHL (Cumulative Index of Nursing and Allied Health Literature), Cochrane Central Register of Controlled Trials, Database of Abstracts of Reviews of Effectiveness, EMBASE, HMIC (Health Management Information Centre), Medline, Medline in Process, Scopus and Web of Knowledge (Science Citation Indexes). To identify additional papers, published, unpublished or research reported in the grey literature a range of relevant websites and trial registers were searched including Clinical Trials.gov. To identify published papers that had not yet been catalogued in the electronic databases, recent editions of key journals were hand-searched. The search terms included 'early warning scores', 'alert criteria', 'rapid response', 'track and trigger' and 'early medical intervention'. (Supplementary Table 1)

Eligibility screening and study selection

PICOS parameters guided inclusion criteria for the validation and effectiveness studies (Supplementary Table 2). Papers reporting development of validation of a PTTT were included for Question 1, whereas papers reporting the implementation of any broader 'paediatric early warning system' (with or without a PTTT) were eligible for Question 2. Both research questions were limited to studies that involved in-patients aged 0-18. Outcome measures considered were mortality and critical events, including: unplanned admission to a higher level of care, cardiac arrest, respiratory arrest, medical emergencies requiring immediate assistance, children reviewed by Paediatric Intensive Care Unit (PICU) staff on the ward (in specialist centres) or reviewed by external PICU staff (for non-specialist centres), acuity at PICU admission and PICU outcomes. A range of study designs were considered for both questions.

For peer review only - http://bmjopen.bmj.com/site/about/guidelines.xhtml

Two of the review authors independently screened the titles and abstracts yielded in the search. Full texts were reviewed independently by six reviewers against the above eligibility criteria and were assigned to the relevant review question if included. Reasons for exclusion were recorded. Separate data extraction forms were developed for validation and effectiveness studies. The forms had common elements (study design, country, setting, study population, description of the PTTT or early warning system, statistical techniques used, outcomes assessed). Additional data items for validation studies included the items in the PTTT, modifications to the PTTT from previous versions, predictive ability of individual items and the overall tool, sensitivity and specificity and inter and intra-rater reliability. Effectiveness studies included an assessment of outcomes in terms of mortality and various morbidity variables. Data extraction was carried out by two reviewers and discrepancies were resolved by discussion. For effectiveness studies, effect sizes and 95% confidence intervals (CI) were calculated or reported as risk ratios (RR) or odds ratios (OR) as appropriate, with p-values reported to assess statistical significance. Data analysis was conducted using an online medical statistics tool.

Quality appraisal

Methodological quality and risk of bias was assessed for each included study using a modified version of the Downs and Black rating scale[9] (templates shown in Supplementary Table 3).

Patient and Public Involvement

This review was conducted as part of a larger mixed-methods study (ISRCTN94228292), which used a formal, facilitated parental advisory group. The group comprised parents of children who had experienced an unexpected adverse event in a paediatric unit and provided input which helped to shape the broader research questions and outcome measures. The results of the review will be disseminated to parents through this group.

For peer review only - http://bmjopen.bmj.com/site/about/guidelines.xhtml

REVIEW RESULTS

Figure 1 shows the PRISMA flow diagram for both research questions.

[FIGURE 1]

Study characteristics

Table 1 summarises the study characteristics of the 36 validation (Question 1) and 30 effectiveness (Question 2) papers included in the review.

Validation studies (n=36)			Effectiveness studies (n=30)		
	N	%		n	%
**Type**			**Type**		
Full text	22	61.1	Full text	21	70.0
Abstract	14	38.9	Abstract	9	30.0
**Country**			**Country**		
United States	15	41.7	United States	18	60.0
United Kingdom	12	33.3	United Kingdom	3	10.0
Canada	2	5.5	Canada	2	6.7
Australia	0	0.0	Australia	3	10.0
Other	5	13.9	Other	3	10.0
Multiple	1	2.8	Multiple	1	3.3
Unclear	1	2.8	Unclear	0	0.0
**Year of study**			**Year of study**		
Pre-2012	10	27.8	Pre-2012	15	50.0
2012	3	8.3	2012	1	3.3
2013	6	16.7	2013	2	6.7
2014	5	13.9	2014	6	20.0
2015	7	19.4	2015	0	0.0
2016	2	5.6	2016	2	6.7
2017	3	8.3	2017	1	3.3
2018	0	0.0	2018	3	10.0
**Setting**			**Setting**		
Specialist / tertiary	33	91.7	Specialist / tertiary	29	96.7
Non-specialist / community	0	0.0	Non-specialist / community	1	3.3
Unclear	3	8.3	Unclear	0	0.0
**Single / multi-centre**			**Single / multi-centre**		
Single-centre	35	97.2	Single-centre	28	93.3
Multi-centre	1	2.8	Multi-centre	2	6.7
**Study population**			**Study population**		
General in-patients	23	63.9	General in-patients	20	66.6
Specialist population	11	30.6	Specialist population	5	16.7
Unclear	2	5.6	Unclear	5	16.7
**Study design**			**Study design**		
Case-control	18	50.0	Uncontrolled before-after	26	86.7

For peer review only - http://bmjopen.bmj.com/site/about/guidelines.xhtml

Case / chart review	10	27.8	Controlled before-after	1	3.3
Cohort	7	19,4	Interrupted Time Series	2	6.7
Pilot study	1	2.8	Cluster randomised trial	1	3.3

Table 1: Summary study characteristics of validation and effectiveness papers in the review

Types of PTTS and components

Across 66 studies, we identified 27 unique PTTT (Table 2). Twenty PTTTs were based on one of four different tools: Monaghan's Brighton PEWS[10], the Bedside PEWS[11], the Bristol PEWT[12] and the Melbourne Activation Criteria[13]. Other PTTT described in the literature included the National Health Service Institute for Innovation and Improvement (NHS III) PEWS[14] (the second most commonly used PTTT in United Kingdom paediatric settings[5]), RRT and MET activation criteria[15–18], and one prediction algorithm developed from a large dataset of electronic health data[19].

[TABLE 2]

Table 2 illustrates the range of physiological and behavioural parameters underpinning PTTT. Common parameters included heart rate (present in 26 out of 27 PTTT), respiratory rate (24), respiratory effort (24) and level of consciousness or behavioural state (24). All PTTT required at least six different parameters to be collected.

Question 1 – How well validated are PTTT and component parts for predicting in-patient deterioration?

Nine validation papers meeting inclusion criteria were excluded from analysis: eight did not report any performance characteristics of the PTTT for predicting deterioration[20–27] and one study calculated incorrect sensitivity/specificity outcomes[12] (Supplementary Table 4). The remaining 27 validation studies, evaluating the performance of 18 unique PTTT, are described in Table 3. Four studies evaluated multiple PTTTs[3,19,28,29] and one paper described three separate studies of the same PTTT[30].

[TABLE 3]

Five cohort studies were included[14,31–34], three based on the same dataset. All other studies were either case-control or chart reviews. Thirteen papers implemented the PTTT in practice[23,30,31,34–43], while the remaining studies 'bench tested' the PTTT – researchers retrospectively calculated the score based on data abstracted from medical charts and records. All studies were conducted in specialist centres with only one multi-centre study reported[44].

Outcome measures

For peer review only - http://bmjopen.bmj.com/site/about/guidelines.xhtml

PTTT were evaluated for their ability to predict a wide range of clinical outcomes. Composite measures were used in eight studies[14,23,29,32,33,37,45,46], cardiac/respiratory arrest or a "code call" was used (singularly or part of a composite outcome) in six studies[23,28,29,37,45,47], while 22 studies used transfer to a to PICU or Paediatric High-Dependency Unit (PHDU) as the main outcome[3,11,19,23,28–34,36,37,39,41–44,46,48,49].

Predictive ability of individual PTTT components

Three validation papers reported on the performance characteristics of individual components of the tool for predicting adverse outcomes[11,33,42]. Parshuram and colleagues, for instance, reported Area Under the Receiver Operating Characteristic curve (AUROC) values for individual PTTT items of a pilot version of the Bedside PEWS: ranging from 0.54 (bolus fluid) to 0.81 (heart rate), compared to 0.91 for the overall PTTT[11]. All other studies reported outcomes for the PTTT as a whole.

Paediatric Early Warning System (PEWS) score

The predictive ability of the 16-item PEWS score was assessed by one internal[47] (AUROC=0.90) and two external case-control studies[28,29] (AUROC range =0.82-0.88) with a range of outcome measures and scoring thresholds. One case-control study used an observed prevalence rate to calculate a positive predictive value (PPV) of 4.2% for the tool in predicting code calls[47] (for every 1,000 patients triggering the PTTT, 42 would be expected to deteriorate).

Bedside PEWS and derivatives

The Bedside PEWS was evaluated in one internal[11] (AUROC=0.91) and five external case-control studies[19,28,44,46,50] (AUROC range=0.73-0.90) for a range of different outcome measures and at different scoring thresholds. One case-control study calculated a PPV of 2.1% for identifying children requiring urgent PICU transfer within 24 hours of admission, based on locally observed prevalence rates[19]. A modified version of the Bedside PEWS (with temperature added) demonstrated an AUROC of 0.86 in an external case-control study with a composite outcome of death, arrest or unplanned PICU transfer[29].

Brighton PEWS and derivatives

Six different PTTT based on the original Brighton PEWS were evaluated across 11 studies[19,31,37,39–42,45,48,50,51]. The Modified Brighton PEWS (a) was evaluated for its ability to predict PICU transfers in one large prospective cohort study (AUROC=0.92, PPV=5.8%)[31],

For peer review only - http://bmjopen.bmj.com/site/about/guidelines.xhtml

and an external case-control study tested the same score for predicting urgent PICU transfers within 24 hours of admission (AUROC=0.74, PPV= 2.1%)[19].

An external case-control study used a composite measure of death, arrest or PICU transfer to evaluate the Modified Brighton PEWS (b) (AUROC=0.79) and the Modified Brighton PEWS (d) (AUROC=0.74)[29]. The latter tool was evaluated in a further internal case-control study for predicting PICU transfer (AUROC=0.82) [48].

The Children's Hospital Early Warning Score (CHEWS) had a reported AUROC of 0.90 for predicting PICU transfers or arrests in a large internal case-control study[51]. A modification for cardiac patients, the Cardiac CHEWS (C-CHEWS) was evaluated by one internal study on a cardiac unit[37] (AUROC = 0.90) looking at arrests or unplanned PICU transfers, and two external studies of oncology / haematology units[41,42] for the same outcome (AUROC=0.95). Finally, the Children's Hospital Los Angeles (CHLA) PEWS was evaluated by in a small internal case-control study for prediction of re-admission to PICU after initial PICU discharge[40] (AUROC=0.71).

Melbourne Activation Criteria (MAC) and derivatives

The MAC was assessed by one external case-control study with an outcome of death, arrest or unplanned PICU transfer[29] (AUROC=0.71) and a large external cohort study with an outcome of death or unplanned PICU or HDU transfer[33] (AUROC=0.79, PPV=3.6%). A derivative of the MAC using an aggregate score, the Cardiff & Vale PEWS (C&VPEWS), was tested using the same cohort and outcome measures in an earlier external study (AUROC=0.86, PPV=5.9%)[32] and was the best performing PTTT in an external case-control study evaluating multiple PTTT[29] (AUROC=0.89).

Bristol PEWT

The Bristol PEWT was evaluated by five external validation studies: two chart review studies[3,35] (no AUROC), one small cohort study of PICU transfers[34] (AUROC=0.91, PPV=11%), and two case-control studies looking at code calls[28] (AUROC=0.75) and a composite of death, arrests and PICU transfers[29] (AUROC=0.62).

Other PTTT

The NHS Institute for Improvement and Innovation (NHS III) PEWS was tested by one external cohort study looking at a composite of death or unplanned transfers to PICU or HDU[14] (AUROC=0.88, PPV=4.3%) and one external case-control study looking at a

For peer review only - http://bmjopen.bmj.com/site/about/guidelines.xhtml

composite of death, arrests and PICU transfers[29] (AUROC=0.82). Zhai and colleagues developed and retrospectively evaluated a logistic regression algorithm in an internal case-control study looking at urgent PICU transfers in the first 24 hours of admission[19] (AUROC =0.91, PPV=4.8%).

Across PTTT, studies reporting performance characteristics of a tool at a range of different scoring thresholds demonstrate the expected interaction and trade-off between sensitivity and specificity – at lower triggering thresholds, sensitivity is high but specificity is low; at higher thresholds, the opposite is true.

Inter-rater reliability and completeness of data

Accurate assessment of the ability of a PTTT to predict clinical deterioration is contingent on accuracy and reliability of tool scoring (whether by bedside nurses in practice or by researchers abstracting data) and the availability of underpinning observations. Only five papers made reference to accuracy or reliability of scoring[28,31,37,42,45], with mixed results: for example, two nurses separately scoring a sub-set of patients on the Modified Brighton PEWS (a) achieved an intra-class coefficient of 0.92[31], but a study nurse and bedside nurse achieved only 67% agreement in scoring the C-CHEWS tool[37]. Completeness of data was reported in 11 studies[11,14,19,29,30,32,33,42,44,45,47]. An evaluation of the Modified Bedside PEWS (a) reported that “the PEWS was correctly performed and could be used for inclusion in the study” in 59% of cases[30], a prospective study bench-testing the C&VPEWS found an average completeness rate of 44% for the seven different parameters in daily practice[32], while a multi-centre study of the Bedside PEWS reported that “only 5.1% [of observation sets] had measurements on all 7 items”[44].

Question 2 – how effective are early warning systems at reducing mortality and critical events in hospitalised children?

Eleven papers meeting inclusion criteria were excluded from analysis for providing insufficient statistical information (e.g., denominator data, absolute numbers of events) to calculate effect sizes[39,52–60]. Further details on papers excluded from analysis are provided in Supplementary Table 5. Findings from the 19 studies included in the analysis are summarised in Table 4.

[TABLE 4]

Type of early warning system interventions

For peer review only - http://bmjopen.bmj.com/site/about/guidelines.xhtml

Seventeen interventions involved the introduction of a new PTTT[13,15–18,61–73], one intervention introduced a mandatory triggering element to an existing PTTT[72], and one study reported a large, multi-centre analysis of MET introduction with no details on PTTT use[74]. Twelve interventions included the introduction of a new MET or RRT[13,15–18,61–66,70], while four further interventions introduced a new PTTT in a hospital with an existing MET or RRT. Only three studies therefore evaluated a PTTT in the absence of a dedicated response team[68,69,71]. A staff education programme was explicitly described in ten interventions[13,15,17,62,63,65,68,69,71,73].

Of the 18 studies that used a PTTT, only seven used a tool that had been formally evaluated for validity: three used the Bedside PEWS[65,66,71], two used the MAC[13,63], one used the Modified Brighton PEWS (b)[73] and one used the C-CHEWS[68]. One study did not report the PTTT used[62], while ten studies used a variety of calling criteria and local modifications to validated tools that had not been evaluated for validity[15–18,61,64,67,69,70,72].

Mortality (ward or hospital wide)

Two uncontrolled before-after studies (both with MET/RRT) reported significant mortality rate reductions post intervention: one in hospital wide deaths per 100 discharges[17] (RR=0.82, 95% CI=0.70-0.95) and one in total hospital deaths per 1,000 admissions (RR=0.65, 0.57-0.75) and deaths on the ward ('unexpected deaths') per 1,000 admissions[63] (RR=0.35, 0.13-0.92). Seven studies found no reductions in mortality, including two high quality multi-centre studies[13,15,61,64–66,74]. Parshuram and colleagues conducted a cluster randomised trial and found no difference in all-cause hospital mortality rates between 10 hospitals randomly selected to receive an intervention centred around use of the Bedside PEWS and 11 usual care hospitals, one year post intervention (OR=1.01, 0.61-1.69)[65]. Kutty *et al.* assessed the impact of MET implementation in 38 US paediatric hospitals with an interrupted time series study, and reported no difference in the slope of hospital mortality rates five years post intervention and the expected slope based on pre-implementation trends (OR = 0.94, 0.93-0.95)[74].

PICU mortality

Two uncontrolled before-after studies (both with MET/RRT) reported a significant post-intervention reduction in rates of PICU mortality among ward transfers (RR=0.31, 0.13-0.72)[18], and PICU mortality rates among patients readmitted within 48 hours (RR=0.43, 0.17-0.99)[64]. Six studies (including a high quality cluster randomised trial and interrupted time

For peer review only - http://bmjopen.bmj.com/site/about/guidelines.xhtml

series study) reported no post-intervention change in PICU mortality using a variety of metrics[65–70].

Cardiac and respiratory arrests

Two uncontrolled before-after studies (both with RRT/MET) reported significant post-intervention rate reductions in sub-categories of cardiac arrests: one in 'near cardiopulmonary arrests'[64] (RR=0.54, 0.52-0.57) but not 'actual cardiopulmonary arrests' and one in 'preventable cardiac arrests'[63] (RR=0.45, 0.20-0.97) but not 'unexpected cardiac arrests'. One uncontrolled before-after study (with RRT/MET) reported a significant post intervention reduction in rates of ward respiratory arrests per 1,000 patient-days[16] (RR=0.27, 0.07-0.95). Seven studies (including one high quality cluster randomised trial and one high quality interrupted time series study) found no change in cardiac arrest rates using a variety of metrics[13,15,16,62,65,66] or cardiac and respiratory arrests combined[61].

Calls for urgent review / assistance

Two uncontrolled before-after studies (all with RRT/MET) reported significant post-intervention reductions in rates of code calls[17,64] (RR=0.29, 0.10-0.65; RR=0.71, 0.61-0.83) while three studies found no change in rates of code calls[15,18,73]. One uncontrolled before-after study in a community hospital (without RRT/MET) found significant post intervention reductions in rates of urgent calls to the in-house paediatrician (RR=0.23, 0.11-0.46) and respiratory therapist[71] (RR=0.36, 0.13-0.95). Two uncontrolled before-after studies (with RRT/MET) found increases in rates of RRT calls[73] (RR=1.59, 1.33-1.90) and outreach team calls[67] (RR=1.92, 1.79-2.07). One study found no change in rates of RRT calls[72].

PICU transfers

One uncontrolled before-after study (without RRT/MET) found a significant post-intervention decrease in the rate of unplanned PICU transfers per 1,000 patient-days[68] (RR=0.70, 0.56-0.88). Four studies (including one high quality cluster randomised trial and one high quality interrupted time series study) found no change in rates of PICU admissions post intervention[65–67,71].

PICU outcomes

Two studies, one interrupted time series and one multi-centre cluster randomised trial (both with RRT/MET), found significant reductions in rates of 'critical deterioration events' (life-sustaining interventions administered within 12 hours of PICU admission) relative to pre-

For peer review only - http://bmjopen.bmj.com/site/about/guidelines.xhtml

implementation trends and relative to control hospitals respectively (IRR=0.38, 0.20-0.75; OR=0.77, 0.61-0.97)[65,66]. One controlled before-after study (without RRT/MET) reported a significant reduction in rates of invasive ventilation given to emergency PICU admissions post intervention (RR=0.83, 0.72-0.97) with no significant change observed in a control group of patients admitted to PICU from outside of the hospital[69]. One uncontrolled before-after study reported a significant post-intervention decrease in rates of PICU admissions receiving mechanical ventilation (RR=0.85, 0.73-0.99) but an increase in rates of early intubation (RR=1.87, 1.33-2.62)[70].

Implementation outcomes

Only three studies reported outcomes relating to the quality of implementation of the intervention. One study reported 99% of audited observation sets of the Bedside PEWS had at least 5 vital signs present post-intervention, up from 76% pre-intervention (no change in control hospitals)[65]. A previous study of the same PTTT reported 3% of audited cases had used the incorrect age chart but reported an intra-class coefficient of 0.90 for agreement between bedside nurses scoring the PTTT in practice and research nurses retrospectively assigned scores[71]. Finally, error rates in C-CHEWS scoring were reported to have reduced from an initial 47% to below 10% by the end of the study[68].

DISCUSSION

This paper reviewed the published PTTT and early warning system literature in order to assess the validity of PTTT for predicting in-patient deterioration (Question 1) and the effectiveness of early warning system interventions (with or without PTTT) for reducing mortality and morbidity outcomes in hospitalised children (Question 2). We believe that the consideration of broader 'early warning systems' differentiates this paper from previous reviews, as does the inclusion of two recently published high-quality effectiveness studies[65,74].

How well validated are existing tools for predicting in-patient deterioration?

Given a growing understanding and emphasis on the importance of local context in healthcare interventions, it is perhaps not surprising that such a wide range of PTTT have been developed and evaluated internationally, and modifications to existing PTTT are common. The result, however, is that a large number of different PTTT have been narrowly validated, but none have been broadly validated across a variety of different settings and

For peer review only - http://bmjopen.bmj.com/site/about/guidelines.xhtml

populations. With only one exception[44], all studies evaluating the validity of PTTT have been single-centre reports from specialist units, greatly limiting the generalisability of the findings.

PTTT such as the Bedside PEWS, C&VPEWS, NHS III PEWS and C-CHEWS have demonstrated very good (AUROC ≥0.80) or excellent (AUROC ≥ 0.90) diagnostic accuracy, typically for predicting PICU transfers, in internal and external validation studies[11,14,19,32,37,42,44,50]. However, methodological issues common to the validation studies mean that such results need to be interpreted with a degree of caution. Firstly, each of the studies was conducted in a clinical setting where paediatric in-patients are subject to various forms of routine clinical intervention throughout their admission.. . There are numerous statistical modelling techniques which can account for co-occurrence of clinical interventions and the longitudinal nature of the predictors[75,76], but none of these were used in the validation studies and so estimates of predictive ability are likely to be distorted. Indeed, the majority of outcomes used in the validation studies are clinical interventions themselves (e.g., PICU transfer). Secondly, while it understandable that a majority of studies ‘bench-tested’ the PTTT rather than implement it into practice before evaluation, the process of abstracting PTTT scores retrospectively from patient charts and medical records introduces a number of sources of potential bias or inaccuracy. For instance, several studies reported either high levels of missing data (i.e., some of the observations required to populate the PTTT score being evaluated were not routinely collected or recorded and so were scored as ‘normal’)[11,19,32,44,45] or difficulty in abstracting certain descriptive or subjective PTTT components[19,28,41,49]. Assuming missing values are normal, or excluding some PTTT items for analysis are both likely to result in underscoring of the PTTT and skew the results. Finally, studies which evaluated a PTTT that had been implemented in practice are at risk of overestimating the ability of PTTT to predict proxy outcomes such as PICU transfer, inasmuch as high PTTT scores or triggers automatically direct staff towards escalation of care, or clinical actions which make escalation of care more likely.

The findings reported in several PTTT studies point towards two potential challenges for some centres in implementing and sustaining a PTTT in clinical practice. As noted above, a number of studies that retrospectively ‘bench-tested’ a PTTT reported that the observations that were required to score the tool were not always routinely collected or recorded in their centre. It may be that the introduction of a PTTT into practice would help create a framework to ensure that core vital signs and observations were collected more routinely (as demonstrated by Parshuram and colleagues[65]) but this would obviously have resource

For peer review only - http://bmjopen.bmj.com/site/about/guidelines.xhtml

implications that could be a potential barrier for some centres. Such considerations are important, as evidence from the adult literature points to the potential for tools to inadvertently mask deterioration when core observations are missing[77]. Secondly, PPV values reported in cohort studies, and case-control studies that adjusted for outcome prevalence, were uniformly low (between 2.3%-5.9%)[14,19,31–33,47]. They demonstrate that even PTTT which demonstrate good predictive performance are likely to generate a large amount of 'false alarms' because adverse outcomes are so rare. For some centres, these issues may be mitigated to some extent by dedicated response teams or other available resources, but other hospitals may not be able to sustain the increased workload of responding to PTTT triggers.

How effective are early warning systems for reducing mortality and morbidity?

We found limited evidence for early warning system interventions reducing mortality or arrest rates in hospitalised children. While some effectiveness papers did report significant reductions in rates of mortality (on the ward or in PICU) or cardiac arrests after implementation of different early warning system interventions[16–18,63,64], they were all uncontrolled before-after studies which have inherent limitations in terms of establishing causality. They do not preclude the possibility that outcome rates would have improved over time regardless of the intervention[78] or changes were caused by other factors, and their inclusion is accordingly discouraged by some Cochrane review groups[79]. Three high quality multi-centre studies - two interrupted time series studies and a recent cluster randomised trial – found no changes in rates or trends of mortality or arrests post intervention[65,66,74].

There was also limited evidence for early warning systems reducing PICU transfers or calls for urgent review. Again, a small number of uncontrolled before-after studies reported significant reductions post-intervention[15,17,64], but several other studies reported significant increases in transfers or calls for review[67,73] or no post-intervention changes. We did find moderate evidence across four studies – including a controlled before-after study, a multi-centre interrupted time series study and a multi-centre cluster randomised trial - for early warning system interventions reducing rates of early critical interventions in children transferred to PICU[65,66,69,70]. Such results are promising, but corresponding reductions in hospital or PICU mortality rates have not yet been reported.

Implementing complex interventions in a healthcare setting is challenging and evidence from the adult literature points to challenges and barriers to successfully implement TTT in practice[80–82]. However, given so few effectiveness studies reported on implementation outcomes, it is difficult to know whether negative findings reflect poor effectiveness or

For peer review only - http://bmjopen.bmj.com/site/about/guidelines.xhtml

implementation of early warning systems. Again, effectiveness studies were predominantly carried out in specialist centres – and in all but three cases[68,69,71], involved the use of a dedicated response team – which greatly limits the generalisability of findings outside of these contexts.

Limitations of the review

There are several limitations of the current review. Firstly, despite purposely widening the scope of the effectiveness review question to include paediatric 'early warning systems' with or without a PTTT, we identified very few studies that did not employ a PTTT as part of the intervention . In part, this likely reflects the fact that PTTT have become almost synonymous with early warning systems, but it is also possible that our search strategy may have missed some broader early warning system initiatives that were not explicitly labelled as such. Secondly, our inclusion criteria for study selection were deliberately broad and so resulted in our including several validation and effectiveness studies that were subsequently excluded from analysis due to insufficient statistical detail or methodological issues. Thirdly, the scope of the current review was limited to consideration of quantitative validation and effectiveness studies. We are mindful of research suggesting that implementing PTTT in practice may confer secondary benefits including, but not limited to improvements in communication, teamwork and empowerment of junior staff to call for assistance[83–85]. Finally, we opted not to conduct a meta-analysis of effectiveness findings due to the heterogeneity of outcome metrics, interventions and study designs, populations and settings. Given the large sample sizes required to detect changes in rare adverse events, we believe further work is needed to harmonise outcome measures used to evaluate early warning system interventions internationally, in order to facilitate pooling of findings across studies.

Conclusion

The PTTT literature is currently characterised by an 'absence of evidence' rather than an 'evidence of absence'. PTTT seem like a logical tool for helping staff detect and respond to deteriorating patients, but the existing evidence base is too limited to form clear judgements of their utility. We would argue that there has been too much confidence placed in the statistical findings of validation studies of PTTT, given methodological limitations in the study designs. There is evidence of consistently high false-alarm rates and bench-testing studies point to many PTTT parameters not being reliably recorded in practice: as such there is reason for caution in considering the viability of PTTT for all hospitals. Almost all of the

For peer review only - http://bmjopen.bmj.com/site/about/guidelines.xhtml

early warning systems and PTTT reported in the literature have been developed and evaluated in specialist centres, typically in units with access to dedicated response teams – yet PTTT appear to be commonly adopted by non-specialist units with little modification. There is currently limited evidence that 'early warning systems' incorporating a PTTT reduce deterioration or death in practice. As such, we would urge caution among policymakers in calling for their use to become mandatory across all hospitals. We acknowledge the potential for PTTT to confer a range of secondary benefits in areas such as communication, teamwork and empowerment of junior staff. More work is required to understand the wider impact of PTTT implementation in different clinical settings before it is possible to evaluate their overall contribution to the wider safety mechanisms and systems aimed at identifying and responding to deteriorating in paediatric patients.

For peer review only

FOOTNOTES

Contributors

RT: screening and review of papers, contribution to design of work, preparation of manuscript; CH: screening and review of papers, contribution to concept and design of work, review of manuscript; FL: contribution to design of work, screening and review of papers, review of manuscript; KH: contribution to concept and design of work, screening and review of papers, review of manuscript; CP, DR, BM, AO, DE, RS, GS, DL, LT, DA, AL, ETJ: contribution to concept and design of work, screening and review of papers, review of manuscript; MM: information specialist, review of manuscript.

Acknowledgements

We would like to acknowledge the contribution of Dr James Bunn to the review.

Competing interests

None declared.

Funding

This study is funded by the National Institute for Health Research (NIHR) Health Services and Delivery Research (HS&DR) programme (12/178/17).

Data sharing statement

No additional data are available.

For peer review only

FIGURE LEGENDS

Figure 1 – PRISMA flow diagram of study inclusion

For peer review only

For peer review only - http://bmjopen.bmj.com/site/about/guidelines.xhtml

REFERENCES

1. Pearson, G. a (Ed). *Why Children Die: A Pilot Study 2006; England (South West, North East and West Midlands), Wales and Northern Ireland.* (2008). doi:ISBN: 978-0-9558055-0-9

2. Joint Commission. 2009 National Patient Safety Goals, Requirements and Rationales. *Jt. Comm. Perspect. Patient Saf.* **8,** 8–12 (2009).

3. Tume, L. The deterioration of children in ward areas in a specialist children's hospital. *Nurs. Crit. Care* **12,** 12–19 (2007).

4. NICE. Acutely ill patients in hospital: Recognition of and response to acute illness in adults in hospital. *NICE Clin. Guidel. 50* 1–107 (2007). doi:10.1136/bmj.39272.679688.47

5. Roland, D., Oliver, A., Edwards, E. D., Mason, B. W. & Powell, C. V. E. Use of paediatric early warning systems in Great Britain: has there been a change of practice in the last 7 years? *Arch. Dis. Child.* **99,** 26–9 (2014).

6. Chapman, S. M., Wray, J., Oulton, K. & Peters, M. J. Systematic review of paediatric track and trigger systems for hospitalised children. *Resuscitation* **109,** 87–109 (2016).

7. Lambert, V., Matthews, A., MacDonell, R. & Fitzsimons, J. Paediatric early warning systems for detecting and responding to clinical deterioration in children: a systematic review. *BMJ Open* **7,** e014497 (2017).

8. Moher, D., Liberati, A., Tetzlaff, J. & Altman, D. G. Preferred reporting items for systematic reviews and meta-analyses: the PRISMA statement. *BMJ* **339,** (2009).

9. Downs, S. H. & Black, N. The feasibility of creating a checklist for the assessment of the methodological quality both of randomised and non-randomised studies of health care interventions. *J. Epidemiol. Community Health* **52,** 377–384 (1998).

10. Monaghan, A. Detecting and managing deterioration in children. *Paediatr. Nurs.* **17,** 32–35 (2005).

11. Parshuram, C. S., Hutchison, J. & Middaugh, K. Development and initial validation of the Bedside Paediatric Early Warning System score. *Crit. Care* **13,** R135–R135 (2009).

12. Haines, C., Perrott, M. & Weir, P. Promoting care for acutely ill children - Development and evaluation of a Paediatric Early Warning Tool. *Intensive Crit. Care Nurs.* **22,** 73–81 (2006).

13. Tibballs, J., Kinney, S., Duke, T., Oakley, E. & Hennessy, M. Reduction of paediatric in-patient cardiac arrest and death with a medical emergency team: preliminary results. *Arch. Dis. Child.* **90,** 1148–1152 (2005).

14. Mason, B. W., Edwards, E. D., Oliver, A. & Powell, C. V. E. Cohort study to test the predictability of the NHS Institute for Innovation and Improvement Paediatric Early Warning System. *Arch. Dis. Child.* archdischild–2015–308465 (2016). doi:10.1136/archdischild-2015-308465

15. Brilli, R. J. *et al.* Implementation of a medical emergency team in a large pediatric teaching hospital prevents respiratory and cardiopulmonary arrests outside the intensive care unit. *Pediatric Critical Care Medicine* **8,** 236–246 (2007).

16. Hunt, E. A. *et al.* Transition from a traditional code team to a medical emergency team

For peer review only - http://bmjopen.bmj.com/site/about/guidelines.xhtml

and categorization of cardiopulmonary arrests in a children's center. *Arch. Pediatr. Adolesc. Med.* **162,** 117–122 (2008).

17. Sharek, P. J. *et al.* Effect of a rapid response team on hospital-wide mortality and code rates outside the ICU in a Children's Hospital. *JAMA* **298,** 2267–2274 (2007).
18. Anwar-Ul-Haque, Saleem, A. F., Zaidi, S. & Haider, S. R. Experience of pediatric rapid response team in a tertiary care hospital in Pakistan. *Indian J. Pediatr.* **77,** 273–276 (2010).
19. Zhai, H. *et al.* Developing and evaluating a machine learning based algorithm to predict the need of pediatric intensive care unit transfer for newly hospitalized children. *Resuscitation* **85,** 1065–1071 (2014).
20. Garlick, J. *et al.* Modified pediatric early warning scores, a predictor of appropriate transfer to higher level of care. *Crit. Care Med.* **41,** A166–A167 (2013).
21. Medar, S., Weingarten-Arams, J. & Katyal, C. Pediatric early warning score (PEWS) is a reliable predictor of rapid response team (RRT) call. *Crit. Care Med.* **43,** 228 (2015).
22. Bell, D. *et al.* The texas children's hospital pediatric advanced warning score as a predictor of clinical deterioration in hospitalized infants and children: A modification of the pews tool. *J. Pediatr. Nurs.* **28,** (2013).
23. McLellan, M. C. & Connor, J. A. The Cardiac Children's Hospital Early Warning Score (C-CHEWS). *J. Pediatr. Nurs.* **28,** 171–178 (2013).
24. Rahman, Z. H. *et al.* Application of the burn center Pediatric Early Warning Score (PEWS) tool to clinical practice: A pilot validation study. *J. Burn Care Res.* **35,** S187 (2014).
25. Hopkins, M., Rowan, C., Rigby, M. & Tori, A. PEWS score predicts the need for PICU transfer and critical care interventions. *Crit. Care Med.* **Conference,** 43rd Critical Care Congress of the Society of Crit (2013).
26. Gawronski, O. *et al.* Bedside pews in an Italian pediatric bone marrow transplant unit: Preliminary results of a longitudinal retrospective chart review. *Intensive Care Med.* **39,** S130 (2013).
27. Sefton, G., Lane, S. & Carrol, E. D. Performance of individual predictors of deterioration used in a paediatric early warning score. *Pediatr. Crit. Care Med.* **15,** 29–30 (2014).
28. Robson, M.-A. J., Cooper, C. L., Medicus, L. a, Quintero, M. J. & Zuniga, S. a. Comparison of three acute care pediatric early warning scoring tools. *J. Pediatr. Nurs.* **28,** e33–41 (2013).
29. Chapman, S. M. *et al.* 'The Score Matters': wide variations in predictive performance of 18 paediatric track and trigger systems. *Arch. Dis. Child.* archdischild–2016–311088 (2017). doi:10.1136/archdischild-2016-311088
30. Fuijkschot, J., Vernhout, B., Lemson, J., Draaisma, J. M. T. & Loeffen, J. L. C. M. Validation of a paediatric early warning score: First results and implications of usage. *Eur. J. Pediatr.* **174,** 15–21 (2015).
31. Tucker, K. M., Brewer, T. L., Baker, R. B., Demeritt, B. & Vossmeyer, M. T. Prospective evaluation of a pediatric inpatient early warning scoring system. *J. Spec. Pediatr. Nurs.* **14,** 79–85 (2009).

For peer review only - http://bmjopen.bmj.com/site/about/guidelines.xhtml

32. Edwards, E. D., Powell, C. V, Mason, B. W. & Oliver, A. Prospective cohort study to test the predictability of the Cardiff and Vale paediatric early warning system. *Arch Dis Child* **94,** 602–606 (2009).

33. Edwards, E. D., Mason, B. W., Oliver, A. & Powell, C. V. E. Cohort study to test the predictability of the Melbourne criteria for activation of the medical emergency team. *Arch. Dis. Child.* **96,** 174–179 (2011).

34. O'Loughlin, K. *et al.* The effectiveness of the paediatric early warning tool (PEWT) in identifying children requiring admission to a critical care unit. *Arch. Dis. Child.* **97,** A154 (2012).

35. Wright, D., Sefton, G. & Horan, M. Charted observations and the use of a paediatric early warning tool did not predict the majority of cardio-respiratory arrest calls in a paediatric hospital. *Arch. Dis. Child.* **96,** A59 (2011).

36. Ahmed, M. *et al.* Burton paediatric early warning system score. *Arch. Dis. Child.* **97,** A421 (2012).

37. Mclellan, M. C., Gauvreau, K. & Connor, J. A. Validation of the cardiac children's hospital early warning score: An early warning scoring tool to prevent cardiopulmonary arrests in children with heart disease. *Congenit. Heart Dis.* **9,** 194–202 (2014).

38. Clayson, R., Selby, A. & Tume, L. Is a paediatric early warning tool (PEWT) useful in cardiac patients? *Pediatr. Crit. Care Med.* **15,** 35–36 (2014).

39. Fenix, J. B., Gillespie, C. W., Levin, A. & Dean, N. Comparison of Pediatric Early Warning Score to Physician Opinion for Deteriorating Patients. *Hosp. Pediatr.* **5,** 474–479 (2015).

40. Mandell, I. M. *et al.* Pediatric Early Warning Score and unplanned readmission to the pediatric intensive care unit. *J. Crit. Care* **30,** 1090–1095 (2015).

41. A., A., P.W., F., N., S. & C., R.-G. Validation of a Pediatric Early Warning Score in Hospitalized Pediatric Oncology and Hematopoietic Stem Cell Transplant Patients. *Pediatr. Crit. Care Med.* **17,** e146–e153 (2016).

42. Agulnik, A. *et al.* Validation of a pediatric early warning system for hospitalized pediatric oncology patients in a resource-limited setting. *Cancer* **123,** 4903–4913 (2017).

43. Blackstone, B., Sharmila, A., Pienaar, A., Bevan, R. & Cheung, R. G453(P) 'Between the flags' paediatric early warning system is a sensitive tool to detect deterioration in children from district general hospitals admitted to paediatric intensive care. *Arch. Dis. Child.* **102,** A178 LP – A179 (2017).

44. Parshuram, C. S. *et al.* Multicentre validation of the bedside paediatric early warning system score: a severity of illness score to detect evolving critical illness in hospitalised children. *Crit Care* **15,** R184 (2011).

45. Akre, M. *et al.* Sensitivity of the pediatric early warning score to identify patient deterioration. *Pediatrics* **125,** e763–9 (2010).

46. Gawronski, O. *et al.* Accuracy of Bedside Paediatric Early Warning System (BedsidePEWS) in a Pediatric Stem Cell Transplant Unit. *J. Pediatr. Oncol. Nurs.* **33,** 249–256 (2016).

47. Duncan, H., Hutchison, J. & Parshuram, C. S. The pediatric early warning system score: A severity of illness score to predict urgent medical need in hospitalized

For peer review only - http://bmjopen.bmj.com/site/about/guidelines.xhtml

children. *J. Crit. Care* **21,** 271–278 (2006).

48. Skaletzky, S. M., Raszynski, A. & Totapally, B. R. Validation of a modified pediatric early warning system score: a retrospective case-control study. *Clin. Pediatr. (Phila).* **51,** 431–5 (2012).

49. Ross, C. *et al.* Sensitivity and specificity of a pediatric early warning system using data-driven vital signs. *Crit. Care Med.* **43,** 39 (2015).

50. Chapman, S. M. *et al.* 'The Score Matters': Wide variations in predictive performance of 18 paediatric track and trigger systems. *Arch. Dis. Child.* **102,** 487–495 (2017).

51. McLellan, M., Gauvreau, K. & Connor, J. A. Validation of the children's hospital early warning scoring system for identifying hospitalized children at risk for arrest or ICU transfer. *Pediatr. Crit. Care Med.* **15,** 30 (2014).

52. Mistry, K. P., Turi, J., Hueckel, R., Mericle, J. M. & Meliones, J. N. Pediatric Rapid Response Teams in the Academic Medical Center. *Clin. Pediatr. Emerg. Med.* **7,** 241–247 (2006).

53. Demmel, K. M., Williams, L. & Flesch, L. Implementation of the pediatric early warning scoring system on a pediatric hematology/oncology unit. *J. Pediatr. Oncol. Nurs.* **27,** 229–240 (2010).

54. Sandhu, S. *et al.* 726 The Paediatric Early Warning Tool in Combination with a Nurse-Led Outreach Team: Impact on Outcomes for Critically Ill Children. *Pediatr Res* **68,** 368 (2010).

55. Randhawa, S., Roberts-Turner, R., Woronick, K. & DuVal, J. Implementing and Sustaining Evidence-Based Nursing Practice to Reduce Pediatric Cardiopulmonary Arrest. *West. J. Nurs. Res.* **33,** 443–456 (2010).

56. Camacho, E. A., Holman, D., Badgett, V., St. Peters, J. & Southworth, B. Modification of a Pediatric Early Warning Tool for Use With Patients in a Specialized Cardiac/Renal Unit. *J. Pediatr. Nurs.* **26,** e2–e3 (2011).

57. Heyden, M. V, Mudge, B. & Casella, S. Initiation of a dedicated pediatric rapid response team reduces cardiopulmonary arrests outside of the intensive care unit in a small children's hospital. *Crit. Care Med.* **40,** 147 (2012).

58. Norville, R., Wills-Bagnato, P., Staton, S. & Kennedy-Nasser, A. Improvement in early recognition of deteriorating pediatric bone marrow transplant patients. *Biol. Blood Marrow Transplant.* **19,** S173 (2013).

59. Ambati, S. R., Sweberg, T., Silver, P. & Gangadharan, S. Effect of simulation based curriculum on the utilization of rapid response team activations (RRT). *Crit. Care Med.* **42,** A1432 (2014).

60. Ocholi, T., Downing, E., Rzeskiewicz, A., Pang, R. & Broughton, S. Impact of the Bedside PEWS on early detection of deteriorating patients. *Arch. Dis. Child.* **99,** A89–A90 (2014).

61. Zenker, P. *et al.* Implementation and impact of a rapid response team in a children's hospital. *Jt. Comm. J. Qual. Patient Saf.* **33,** 418–425 (2007).

62. Hanson, C. C. *et al.* A reduction in cardiac arrests and duration of clinical instability after implementation of a paediatric rapid response system. *Postgrad. Med. J.* **86,** 314–318 (2010).

63. Tibballs, J. & Kinney, S. Reduction of hospital mortality and of preventable cardiac

For peer review only - http://bmjopen.bmj.com/site/about/guidelines.xhtml

arrest and death on introduction of a pediatric medical emergency team. *Pediatr. Crit. Care Med.* **10,** 306–312 (2009).

64. Kotsakis, A. *et al.* Implementation of a multicenter rapid response system in pediatric academic hospitals is effective. *Pediatrics* **128,** 72–78 (2011).

65. CS, P., Dryden-Palmer, K., Farrell, C. & al, et. Effect of a pediatric early warning system on all-cause mortality in hospitalized pediatric patients: The epoch randomized clinical trial. *JAMA* **319,** 1002–1012 (2018).

66. Bonafide, C. P. *et al.* Impact of Rapid Response System Implementation on Critical Deterioration Events in Children. *JAMA Pediatr.* **168,** 25 (2014).

67. Duns, N. *et al.* Introduction of a track and trigger system is associated with increased paediatric intensive care outreach utilisation and a trend towards improved patient outcomes in a tertiary children's hospital. *Aust. Crit. Care* **27,** 61 (2014).

68. Agulnik, A. *et al.* Improved outcomes after successful implementation of a pediatric early warning system (PEWS) in a resource-limited pediatric oncology hospital. *Cancer* **123,** 2965–2974 (2017).

69. Sefton, G. *et al.* What impact did a Paediatric Early Warning system have on emergency admissions to the paediatric intensive care unit? An observational cohort study. *Intensive Crit. care Nurs.* **31,** 91–99 (2015).

70. Kolovos, N. S., Gill, J., Michelson, P. H., Doctor, A. & Hartman, M. E. Reduction in Mortality Following Pediatric Rapid Response Team Implementation. *Pediatr. Crit. Care Med.* 1 (2018). doi:10.1097/PCC.0000000000001519

71. Parshuram, C. S., Bayliss, A., Reimer, J., Middaugh, K. & Blanchard, N. Implementing the Bedside Paediatric Early Warning System in a community hospital: A prospective observational study. *Paediatr. Child Health* **16,** e18–22 (2011).

72. Panesar, R. *et al.* Characteristics and outcomes of pediatric rapid response teams before and after mandatory triggering by an elevated Pediatric Early Warning System (PEWS) score. *Hosp. Pediatr.* **4,** 135–140 (2014).

73. Douglas, K., Collado, J. C. & Keller, S. Implementation of a Pediatric Early Warning Scoring System at an Academic Medical Center. *Crit. Care Nurs. Q.* **39,** 363–370 (2016).

74. Kutty, S. *et al.* Association of Pediatric Medical Emergency Teams With Hospital Mortality. *Circulation* **137,** 38–46 (2018).

75. Guerra, M. W., Shults, J., Amsterdam, J. & Ten-Have, T. The analysis of binary longitudinal data with time-dependent covariates. *Stat. Med.* **31,** 931–948 (2012).

76. Selig, J. P., Preacher, K. J. & Little, T. D. Modeling Time-Dependent Association in Longitudinal Data: A Lag as Moderator Approach. *Multivariate Behav. Res.* **47,** 697–716 (2012).

77. Clifton, D. A. *et al.* 'Errors' and omissions in paper-based early warning scores: the association with changes in vital signs-a database analysis. *BMJ Open* **5,** e007376 (2015).

78. Joffe, A. R., Anton, N. R. & Burkholder, S. C. Reduction in hospital mortality over time in a hospital without a pediatric medical emergency team: limitations of before-and-after study designs. *Arch. Pediatr. Adolesc. Med.* **165,** 419–423 (2011).

79. Goodacre, S. Uncontrolled before-after studies: Discouraged by Cochrane and the

For peer review only - http://bmjopen.bmj.com/site/about/guidelines.xhtml

EMJ. *Emergency Medicine Journal* **32,** 507–508 (2015).

80. Niegsch, M., Fabritius, M. L. & Anh??j, J. Imperfect Implementation of an Early Warning Scoring System in a Danish Teaching Hospital: A Cross-Sectional Study. *PLoS One* **8,** (2013).

81. Hands, C. *et al.* Patterns in the recording of vital signs and early warning scores: Compliance with a clinical escalation protocol. *BMJ Qual. Saf.* **22,** 719–726 (2013).

82. Lydon, S., Byrne, D., Offiah, G. & Gleeson, L. A mixed-methods investigation of health professionals' perceptions of a physiological track and trigger system. *BMJ Qual.* 688–695 (2015). doi:10.1136/bmjqs-2015-004261

83. Bonafide, C. P. *et al.* Beyond statistical prediction: Qualitative evaluation of the mechanisms by which pediatric early warning scores impact patient safety. *J. Hosp. Med.* **8,** 248–253 (2013).

84. Ennis, L. Paediatric early warning scores on a children's ward: a quality improvement initiative. *Nurs. Child. Young People* **26,** 25–31 (2014).

85. Kaul, M. *et al.* Implementation of the Bedside Paediatric Early Warning System (BedsidePEWS) for nurse identification of deteriorating patients. *J. Spec. Pediatr. Nurs.* **19,** 339–349 (2014).

For peer review only

For peer review only - http://bmjopen.bmj.com/site/about/guidelines.xhtml

Table 2– summary of PTTTs

PTTT name (references)	Development / modification details	Score / trigger	Choice of thresholds / parameters	Age-dependent thresholds?	No. of items in the tool*	PTTT parameters															
						Respiratory rate	Heart rate	Respiratory effort / distress	LOC / behaviour	Oxygen saturation	Capillary refill time	Oxygen therapy	Systolic blood pressure	Pain	Staff concern	Skin colour	Airway problems	Temperature	Pulses	Family concern	Other items
Paediatric Early Warning System (PEWS) score[28,47]	Developed for use in Canadian tertiary centre[47]. Nurse-generated candidate items reduced by focus groups/Delphi and evaluation with clinical dataset (code blue calls, n=87; controls, n=128). Development and validation datasets not independent.	Score	Expert opinion	Yes	16	✓	✓		✓	✓	✓	✓	✓				✓	✓	✓		Bolus fluid, medications, home oxygen, any previous admission to an ICU, central venous line in situ, transplant recipient, severe cerebral palsy, gastrostomy tube, greater than 3 medical specialties involved in care
Bedside Paediatric Early Warning Score (PEWS)[11,19,25,26,28,44,46,60,65,66,71]	Developed for use in US tertiary centre[11]. Routinely collected items assessed for discriminatory ability using clinical dataset (PICU admission, n=60; controls, n=120). Development and validation set not independent.	Score	Expert opinion	Yes	7	✓	✓	✓		✓	✓	✓	✓								
Modified Bedside PEWS (a)[30]	Modification to Bedside PEWS for use in Dutch tertiary centre. Added temperature; modified wording of respiratory effort and oxygen therapy items.	Score	Expert opinion	Yes	8	✓	✓	✓		✓	✓	✓	✓					✓			
Modified Bedside PEWS (b)[49]	Modification to Bedside PEWS for use in US tertiary centre. Changed normal thresholds for HR and RR based on analysis of local clinical data.	Score	HR / RR data driven	Yes	7	✓	✓	✓		✓	✓	✓	✓								
Brighton PEWS[10,55]	Initial development for use in UK tertiary centre. Adapted from existing adult scores, but amended based on local clinical consensus. Small audit of patients (n=30) described but no formal validation.	Score	Expert opinion	No	5	✓	✓	✓	✓		✓	✓				✓					¼ hourly nebulisers, persistent vomiting post-surgery

Modified Brighton PEWS (a)[19,31,39]	Modification of Brighton PEWS for use in general medical ward of a US tertiary centre. Altered thresholds for oxygen therapy; changed wording for respiratory effort; modified escalation algorithm.	Score	Expert opinion	No	5	✓	✓	✓	✓		✓	✓				✓					¼ hourly nebulisers, persistent vomiting post-surgery
Modified Brighton PEWS (b)[45,73]	Modification of Brighton PEWS for use in US tertiary centre. Added age-dependent thresholds for HR and RR.	Score	Expert opinion	Yes	5	✓	✓	✓	✓		✓	✓				✓					¼ hourly nebulisers, persistent vomiting post-surgery
Modified Brighton PEWS (c)[53]	Modification of Brighton PEWS for use in a US haematology / oncology unit. Altered thresholds; changed respiratory effort wording; modified escalation algorithm; added and removed items. No formal validation study reported.	Score	Expert opinion	No	3	✓	✓	✓	✓		✓	✓				✓				✓	
Modified Brighton PEWS (d)[48]	Modification of Brighton PEWS for use in a US tertiary centre. Modified wording of Behaviour component, added age-dependent thresholds for HR and RR; removed nebulisers and persistent vomiting.	Score	Expert opinion	Yes	3	✓	✓	✓	✓		✓	✓				✓					
Modified Brighton PEWS (e)[72]	Modification of Brighton PEWS for use in a US tertiary centre. Modified wording of Behaviour and respiratory effort items; altered thresholds for O2 therapy; removed nebs and persistent vomiting items. No formal validation study reported.	Score	Expert opinion	No	3	✓	✓	✓	✓		✓	✓				✓					
Texas Children's Hospital (TCH) PAWS[22]	Modification of Brighton PEWS for use in a US tertiary centre. Modified wording of Behaviour category; added scoring items to Resp. and Cardiovascular categories; changed O2 therapy thresholds; modified escalation algorithm.	Score	Expert opinion	No	5	✓	✓	✓	✓	✓	✓	✓				✓					Hourly respiratory treatments; persistent vomiting post-surgery
Children's Hospital Early Warning Score (CHEWS)[51]	Modification of Brighton PEWS for use in a US tertiary centre. Altered thresholds for O2 therapy; changed wording for Behaviour and Resp. categories; added staff and family concern; removed nebs and vomiting; modified escalation algorithm.	Score	Expert opinion	No	5	✓	✓	✓	✓		✓	✓			✓	✓				✓	

For peer review only - http://bmjopen.bmj.com/site/about/guidelines.xhtml

Children's Hospital Cardiac Early Warning Score (C-CHEWS) [23,41,42,68]	Modification of Brighton PEWS for cardiac ward of a US tertiary centre. Altered O2 therapy thresholds; added items to Behaviour, Resp. and Cardiovascular categories; added family & staff concern; added age-related thresholds; removed nebs and vomiting items; modified escalation algorithm.	Score	Expert opinion	Yes	5	✓	✓	✓	✓	✓	✓	✓		✓	✓	✓			✓	✓	
Burn-specific PEWS [24]	Modification of Brighton PEWS, for use in a specialist Burn Centre of a US tertiary centre. Added temperature; added intake and output scoring items; added Skin component.	Score	Expert opinion	No	6	✓	✓	✓	✓	✓	✓	✓				✓			✓		Intake; outputs; skin
Children's Hospital Los Angeles (CHLA) PEWS [40]	Modification of Brighton PEWS for use in a US tertiary centre. Added medical history scoring item; added single ventricle physiology scoring item; changed O2 therapy thresholds; added items to Resp. category.	Score	Expert opinion		4	✓	✓	✓	✓		✓	✓				✓					RRT, code blue, or transfer from/to PICU in past 2 weeks; single ventricle physiology; any assisted ventilation
Melbourne Activation Criteria (MAC) [3,13,33,63]	Initial development for use in an Australian tertiary centre to activate MET. Adapted from adult MET calling criteria, using age-appropriate thresholds. No formal validation study reported.	Trigger	Expert opinion	Yes	9	✓	✓	✓	✓	✓			✓		✓		✓				Cardiac or respiratory arrest
Modified MAC [64]	Modification of MAC for use in a Canadian tertiary centre, to activate a RRS. Removed cardiac / respiratory arrest outcome. No formal validation study reported.	Trigger	Expert opinion	Yes	8	✓	✓	✓	✓	✓			✓		✓		✓				
Cardiff & Vale Paediatric Early Warning Score (C&VPEWS) [32,33]	Modification of MAC for evaluation in a UK tertiary centre. Removed cardiac / respiratory arrest outcome; altered thresholds of some items; evaluated as aggregate score rather than single-item trigger.	Score	Expert opinion	Yes	8	✓	✓	✓	✓	✓			✓		✓		✓				
Bristol Paediatric Early Warning Tool (PEWT) [3,12,28,34,35]	Initial development for use in a UK tertiary centre. Initial candidate items drawn from un-validated Plymouth tool – retrospectively evaluated for ability to predict adverse events among cases (n=360, HDU or PICU transfers). Development and validation dataset not independent.	Trigger	APLS values	Yes	14	✓	✓		✓	✓	✓	✓	✓	✓	✓		✓				Required nebulised adrenaline; hyperkalaemia; suspected meningococcus; diabetic ketoacidosis; persistent convulsion

For peer review only - http://bmjopen.bmj.com/site/about/guidelines.xhtml

Modified Bristol PEWT (a)[69]	Modification of Bristol PEWT for a UK tertiary centre. Adjusted wording of Airway parameters; added respiratory items; added AVPU evaluation; removed suspected meingococcus and diabetic ketoacidosis; added ph<7.2 and unresolved pain. No formal validation study reported.	Trigger	APLS values	Yes	14	✓	✓	✓	✓	✓	✓	✓	✓	✓	✓		✓				Required nebulised adrenaline or no improvement after nebulisers; pH<7.2; unresolved pain or current analgesic therapy; fitting
Modified Bristol PEWT (b)[38]	Modification of Bristol PEWT for cardiac ward of a UK tertiary centre. Amended HR and RR thresholds. Adjusted wording of Airway parameters; added respiratory items; added AVPU evaluation; removed suspected meingococcus and diabetic ketoacidosis; added ph<7.2 and unresolved pain	Trigger	HR / RR data driven	Yes	14	✓	✓	✓	✓	✓	✓	✓	✓	✓	✓		✓				Required nebulised adrenaline or no improvement after nebulisers; pH<7.2; unresolved pain or current analgesic therapy; fitting
NHS Institute for Innovation and Improvement (NHS III) PEWS[14]	Designed as part of a NHS Institute fellowship project. Adapted from adult scores and Brighton PEWS. No formal development or internal validation study published.	Score	APLS values	Yes	6	✓	✓	✓	✓			✓			✓						
Paediatric Medical Emergency Team (PMET) triggering criteria (a)[15]	Initial development for use in a US tertiary centre to activate a MET. Retrospective chart review of case patients (n-44, code calls) used to generate candidate items. Clinical judgement used to select final items. No formal validation of final tool reported.	Trigger	Expert opinion	No	4			✓	✓	✓					✓	✓				✓	Worsening retractions; cyanosis
Paediatric Medical Emergency Team (PMET) triggering criteria (b)[16]	Initial development for use in a US tertiary centre to activate a MET. Minimal description of tool development – authors deliberately chose broad criteria and categories of illness rather than specific vital signs. No formal validation study reported.	Trigger	Expert opinion	Unclear	12	✓	✓	✓	✓	✓					✓					✓	Cardiac or respiratory arrest; seizures with apnoea; progressive lethargy; circulatory compromise/acute shock syndrome
Paediatric Rapid Response Team (PRRT) triggering criteria (a)[17]	Initial development for use in a US tertiary centre, to activate a RRT. Triggering items elected through expert consensus locally – reference to similarity to MAC and PMET triggering criteria (a). No formal validation study reported.	Trigger	Expert opinion	No	6	✓	✓		✓	✓			✓		✓						

For peer review only - http://bmjopen.bmj.com/site/about/guidelines.xhtml

Paediatric Rapid Response Team (PRRT) triggering criteria (b)[18]	Initial development for use in calling RRT team in a tertiary centre in Pakistan. Minimal explanation for selection of calling criteria. No formal validation study reported in the literature.	Trigger	Unclear	Yes	8	✓	✓	✓	✓	✓			✓		✓						Convulsion
Logistic regression algorithm[19]	Initial development based on data mining of electronic health records in US tertiary-centre. Extracted 24 hours of clinical data from inpatients (n=6,722 controls, 526 PICU transfers) and used logistic regression model to select 29 item tool. Validation performed on subset of development dataset.	Score	Expert opinion	Yes	29	✓	✓	✓	✓	✓	✓		✓	✓				✓			Acuity level (local measure); tissue perfusion and oxygenation

* Multiple parameters are often required to be collected for each scoring item/category, e.g., scoring the 'Cardiovascular' category in the Brighton PEWS requires collection / evaluation of heart rate, skin colour and capillary refill time

† Denotes a study included in the effectiveness review

PTTS: Paediatric Track and Trigger Tool; PICU: Paediatric Intensive Care Unit; PHDU: Paediatric High-Dependency Unit; HR: Heart rate; RR: Respiratory rate; APLS: Advanced Paediatric Life Support; AVPU: Alert, Voice, Pain, Responsive ; GCS: Glasgow Coma Scale; LOC: Level of consciousness

For peer review only

For peer review only - http://bmjopen.bmj.com/site/about/guidelines.xhtml

Table 3 – summary of PTTT validation study outcomes

PTTT	First author, year	Country	Study population	Study design	Number of centres	PTTT used in practice?	Internal / external validation study?	Outcome measures	Sample size	Score or trigger?	Score tested / maximum score	Which score used (frequency of scoring)?*	AUROC	Sensitivity	Specificity	PPV	NPV	Notes on accuracy / reliability of scoring and missing data	Quality score (max = 24)
Paediatric Early Warning System (PEWS) score	Duncan 2006[47]	Canada	All in-patients	Case-control study (retrospective)	1	No	Int	Code blue call for actual or impending cardiopulmonary arrest	215 (87 cases)	S	5 / 26	Max 24hrs before event (hourly)	0.90	78.0	95.0	4.2†		No details on data abstraction. 13% of eligible cases and 84% of eligible controls excluded due to incomplete clinical data.	14
	Robson 2013[28]	US	All in-patients	Case-control study (retrospective)	1	No	Ext	Code blue call	192 (96 cases)	S	5 / 32	Max 24hrs before event (6 hourly)	0.85	86.6	72.2			Four researchers scored PTTT from 20 charts, inter-rater reliability of 0.95. No details on extent of missing data.	8
	Chapman 2017[50]	UK	All in-patients	Case-control study (retrospective)	1	No	Ext	Death, arrest or unplanned PICU transfer	608 (297 cases)	S	7 / 32	Max 48hrs before event (per usual practice)	0.82	70.0	75.0	*72.6*	*72.0*	Data abstraction by single researcher. 36% of observation sets contained HR, RR, O2 Sats, systolic BP, temperature and assessment of consciousness.	17
Bedside PEWS	Parshuram 2009[11]	Canada	All in-patients	Case-control study (retrospective)	1	No	Int	Urgent PICU transfer (without code blue call)	180 (60 cases)	S	8 / 26	Max 24hrs before event (hourly)	0.91	82.0	93.0			Availability of scoring items in medical records varied from 27% (cap refill time) to 93% (oxygen therapy).	21

	Parshuram 2011[44]	Canada & UK	All in-patients	Case-control study (prospective)	4	No	Ext	Urgent PICU transfer or immediate call to resuscitation team	2,074 (686 cases)	S	7 / 26	Max 24hrs before event (hourly)	0.87	64.0	91.0			PTTT scores calculated electronically after abstraction by research nurse. 5.1% of records had all 7 items recorded, 31% had at least 5 items.	22
	Robson 2013[28]	US	All in-patients	Case-control study (retrospective)	1	No	Ext	Code blue call	192 (96 cases)	S	7 / 26	Max 24hrs before event (6 hourly)	0.73	56.3	78.1			See above.	8
	Zhai 2014[19]	US	All in-patients	Case-control study (retrospective)	1	No	Ext	Urgent PCU transfer within 24 hrs of admission	6,352 (53 cases)	S	7 / 26	Max 24 hrs before event (hourly)	0.82	73.6	71.7	2.1†		Data extracted from electronic health records. Excluded two items of Bedside PEWS (oxygen therapy and respiratory effort) due to difficulty abstracting.	17
	Gawronski 2016[46]	Italy	Stem Cell Transplant Unit	Case-control study (retrospective)	1	No	Ext	Unexpected death, urgent consult with RRT or urgent PICU transfer	99 (19 cases)	S	6 / 26	Score 4hrs before event	0.90	79.0	97.5			Data abstracted by research nurses. No details on extent of missing data. Conflicting / missing observations resolved by interviews with clinical staff.	15
	Chapman 2017[50]	UK	All in-patients	Case-control study (retrospective)	1	No	Ext	Death, arrest or PICU transfer	608 (297 cases)	S	6 / 26	Max 48hrs before event (per usual practice)	0.88	72.0	89.0	*86.0*	*77.0*	See above.	17

For peer review only

Modified Bedside PEWS (a)	Fuijkschot 2015[30] (study 1)	Netherlands	Oncology ward	Case-cohort study (retrospective)	1	Yes	Int	Emergency medical intervention or reviewed by PICU staff or staff concern	118 (15 cases)	S	8 / 28	Unclear (minimum 8 hourly)				*73.0*		41% of admissions excluded from study due to incomplete PTTT scores.	10
	Fuijkschot 2015[30] (study 2)	Netherlands	All in-patients	Case-cohort study (retrospective)	1	Yes	Int	PICU transfer	Unclear (24 cases)	S	8 / 28	Score 2-6hrs before event (minimum 8 hourly)		66.6				High rate of exclusions reported due to missing data.	10
	Fuijkschot 2015[30] (study 3)	Netherlands	All in-patients	Case-cohort study (prospective)	1	Yes	Int	Emergency medical intervention	Unclear (14 cases)	S	8 / 28	Unclear (minimum 8 hourly)		100				No details on missing data.	10
	Chapman 2017[50]	UK	All in-patients	Case-control study (retrospective)	1	No	Ext	Death, arrest or PICU transfer	608 (297 cases)	S	7 / 28	Max 48hrs before event (per usual practice)	0.87	69.0	91.0	*87.9*	*79.0*	See above.	17
Modified Bedside PEWS (b)	Ross 2015[49]	US	All in-patients	Case-control study (retrospective)	1	No	Int	Urgent PICU transfer	4628 (848 cases)	S	8 / 26	Max during admission		70.0	84.0			No details on data abstraction. Respiratory effort category excluded due to difficulty abstracting. No details on missing data.	9
Modified Brighton PEWS (a)	Tucker 2008[31]	US	General medical unit	Cohort study (prospective)	1	Yes	Int	PICU transfer	2,979 (51 cases)	S	3 / 11	Max during admission (4 hourly)	0.89	90.2	74.4	5.8	99.8	Intraclass coefficient of 0.92 reported for two bedside nurses scoring 55 patients. No details on missing data.	14

For peer review only

	Zhai 2014[19]	US	All in-patients	Case-control study (retrospective)	1	No	Ext	Urgent PCU transfer within 24 hrs of admission	6,352 (53 cases)	S	2 / 11	Max 24hrs before event (hourly)	0.74	68.4	81.6	2.3		Data extracted from electronic health records. Only included records with complete PEWS score: 64% of eligible cases and 51% of eligible controls excluded.	17
	Fenix 2015[39]	US	PICU transfers among all in-patients (excluding haematology oncology, surgical and cardiac wards)	Case-control study (retrospective)	1	Yes	Ext	Non-elective PICU transfer followed by deterioration event	97 PICU transfers (51 cases of PICU transfer followed by 'deterioration event')	S	3 / 11	Max during admission		80.0	43.0	*61.0*	*67.0*	No details on missing data.	15
Modified Brighton PEWS (b)	Akre 2010[45]	US	All in-patients	Chart review study (retrospective)	1	No	Int	Rapid response team call or code blue call	186 cases (170 RRT calls, 16 code calls)	S	4 / 13	Max 24 hrs before event (minimum 4 hourly)		85.5				Scores abstracted from charts by single nurse, having calibrated with advanced nurse practitioner. Categories scored missing if any items missing. 25% of charts missing behavioural state, 26% cardiovascular colour.	14
	Chapman 2017[50]	UK	All in-patients	Case-control study (retrospective)	1	No	Ext	Death, arrest or PICU transfer	608 (297 cases)	S	4 / 13	Max 48hrs before event (per usual practice)	0.79	61.0	84.0	*78.4*	*69.0*	See above.	17

For peer review only

Modified Brighton PEWS (d)	Skaletzky 2012[48]	US	Medical surgical wards	Case-control study (retrospective)	1	No	Int	PICU transfer	350 (100 cases)	S	2.5 / 9	Max 48hrs before event (4 hourly)	0.81	62.0	89.0			Data abstracted from medial charts and notes. Behaviour category abstracted from LOC. No details on missing data.	15
	Chapman 2017[50]	UK	All in-patients	Case-control study (retrospective)	1	No	Ext	Death, arrest or PICU transfer	608 (297 cases)	S	4 / 9	Max 48hrs before event (per usual practice)	0.74	46.0	90.0	*81.3*	*63.0*	See above.	17
Children's Hospital Early Warning Score (CHEWS)	McLellan 2014[51]	US	All in-patients	Case-control study (retrospective)	1	Yes	Int	Arrest or unplanned PICU transfer	1,136 (360 cases)	S	4 / 12	Max in admission (4 hourly)	0.90	84.2	80.9			No details on missing data.	10
Children's Hospital Cardiac Early Warning Score (C-CHEWS)	McLellan 2013[23]	US	Cardiovascular unit	Case-control study (retrospective)	1	Yes	Int	Arrest or unplanned PICU transfer	312 (64 cases)	S	3 / 12	Max 18hrs before event (4 hourly)	0.86	95.3	76.2	*50.8*	*98.4*	Study nurse and bedside nurses assessed scores for 37 patients, 67% agreement. No details on missing data.	9
	Agulnik 2016[41]	US	Oncology unit	Case-control study (retrospective)	1	Yes	Ext	Unplanned PICU transfer	330 (110 cases)	S	4 / 12	Max 24 hours before event (4 hourly)	0.96	86.0	95.0			PTTT scores abstracted by researcher. Did not abstract if vital signs were present but no PTTT score calculated by nurse. No details on missing data.	14

For peer review only

	Agulnik 2017[42]	Guatemala	Oncology unit	Case-control study (retrospective)	1	Yes	Ext	Unplanned PICU transfer	258 (129 cases)	S	4 / 12	Max 24hrs before event (3 hourly)		91.0	88.0			Researcher evaluated charts and calculated scores, reporting 14% error rate (PTTT score calculated incorrectly) and 3% omission rate (vital signs recorded but no PTTT score calculated). 1 out of 130 cases excluded due to missing PTTT documentation.	16
Children's Hospital Los Angeles (CHLA) PEWS	Mandell 2015[40]	US	In-patients discharged from PICU to ward	Case-control study (retrospective)	1	Yes	Int	Early unplanned re-admission to PICU (within 48 hours of discharge from PICU to ward)	189 (38 cases)	S	2 / 10	First score assigned on ward, post PICU discharge	0.71	76.0	56.0			No details on missing data.	12
Melbourne Activation Criteria (MAC)	Tume 2007[3]	UK	In-patients with an unplanned PICU transfer	Chart review study (retrospective)	1	No	Ext	Unplanned PICU transfer	33 cases	T	NA	Unclear		87.8				Data abstracted by two reviewers. Reference to "large number of missing records and observation charts".	11
	Tume 2007[3]	UK	In-patients with an unplanned PHDU transfer	Chart review study (retrospective)	1	No	Ext	Unplanned PHDU transfer	32 cases	T	N/A	Unclear		87.5				See above.	11

For peer review only

	Edwards 2011[33]	UK	All in-patients	Cohort study (retrospective)	1	No	Ext	Death or unplanned PICU or HDU transfer	1,000 (16 cases)	T	N/A	Any trigger over admission (per usual practice)	0.79	68.3	83.2	3.6	99.7	Observation charts altered to include all PTTT parameters. 56% of records missing at least one component. Missing data assumed to be normal.	17
	Chapman 2017[50]	UK	All in-patients	Case-control study (retrospective)	1	No	Ext	Death, arrest or PICU transfer	608 (297 cases)	T	NA	Max 48hrs before event (per usual practice)	0.71	93.0	49.0	*64.0*	*88.0*	See above.	17
Cardiff & Vale Paediatric Early Warning Score (C&VPEWS)	Edwards 2009[32]	UK	All in-patients	Cohort study (prospective)	1	No	Int	Death or unplanned PICU or HDU transfer	1,000 (16 cases)	S	2 / 8	Max score during admission (per usual practice)	0.86	69.5	89.9	5.9	99.7	Observation charts altered to include all PTTT parameters. 56% of records missing at least one component. Missing data assumed to be normal.	18
	Chapman 2017[50]	UK	All in-patients	Case-control study (retrospective)	1	No	Ext	Death, arrest or PICU transfer	608 (297 cases)	S	3 / 8	Max 48hrs before event (per usual practice)	0.89	80.0	86.0	*84.0*	*82.0*	See above.	17
Bristol Paediatric Early Warning Tool (PEWT)	Tume 2007[3]	UK	In-patients with an unplanned PICU transfer	Chart review (retrospective)	1	No	Ext	Unplanned PICU transfer	33 cases	T	N/A	Unclear		87.8				See above.	11
	Tume 2007[3]	UK	In-patients with an unplanned PHDU transfer	Chart review (retrospective)	1	No	Ext	Unplanned PHDU transfer	32 cases	T	N/A	Unclear		84.4				See above.	11

For peer review only - http://bmjopen.bmj.com/site/about/guidelines.xhtml

	Wright 2011[35]	UK	All in-patients	Chart review (retrospective)	1	Yes	Ext	Cardiac arrest	55 cases	T	N/A	If triggered 24hrs before event		49.1				One case excluded due to missing notes. No details on missing data.	11
	O'Loughlin 2012[34]	UK	All in-patients	Cohort study (prospective)	1	Yes	Ext	PICU transfer	331 (7 cases)	T	N/A	Triggered during admission (12hrly)	0.91	100	81.0	11.0		No details on missing data.	6
	Robson 2013[28]	US	All in-patients	Case-control study (retrospective)	1	No	Ext	Code blue call	192 (96 cases)	T	N/A	Triggered 24hrs before event (6hrly)	0.75	76.3	61.5			See above.	8
	Chapman 2017[50]	UK	All in-patients	Case-control study (retrospective)	1	No	Ext	Death, arrest or PICU transfer	608 (297 cases)	T	N/A	If triggered 48hrs before event (per usual practice)	0.62	96.0	28.0	*56.0*	*88.0*	See above.	17
Modified Bristol Paediatric Early Warning Tool (PEWT) (b)	Clayson 2014[38]	UK	Cardiac ward	Cohort study (prospective)	1	Yes	Int	'A deteriorating patient'	126 (unclear number of cases)	T	N/A	Unclear				*12.5*	*97.0*	No details on missing data.	5
NHS Institute for Innovation and Improvement (NHS III) PEWS	Mason 2016[14]	UK	All in-patients	Cohort study (retrospective)	1	No	Ext	Death or unplanned PICU or HDU transfer	1,000 (16 cases)	S	2 / 7	Max score over admission (per usual practice)	0.88	80.0	81.0	4.3	99.7	Observation charts altered to include all PTTT parameters. 56% of records missing at least one component. Missing data assumed to be normal.	15

For peer review only - http://bmjopen.bmj.com/site/about/guidelines.xhtml

	Chapman 2017[50]	UK	All in-patients	Case-control study (retrospective)	1	No	Ext	Death, arrest or PICU transfer	608 (297 cases)	S	2 / 7	Max 48hrs before event (per usual practice)	0.82	83.0	65.0	*69.6*	*80.0*	See above.	17
Logistic regression algorithm	Zhai 2014[19]	US	All in-patients	Case-control study (retrospective)	1	No	Ext	Urgent PICU transfer within 24 hrs of admission	6,352 (53 cases)	S	> 0.5	Max 24hrs before event (hourly)	0.91	84.9	85.9	4.8		Data extracted from electronic health records. No details on extent of missing data but authors report that "missing data was a major cause of incorrect prediction".	17
Burton Paediatric Early Warning Score (BPEWS)	Ahmed 2012[36]	UK	PICU admissions only	Chart review (retrospective)	1	Yes	Int	PICU admission	23	S	4 / 19	Max 24hrs before event (unclear)		93.0				Data extracted from case notes by two reviewers. No details on missing data.	4
'Between the Flags' Paediatric Early Warning System (PEWS)	Blackstone 2017[43]	UK	Urgent PICU admissions only	Chart review (retrospective)	1	Yes	Ext	Urgent PICU admission	100	T	NA	Unclear		91.0				Data extracted from health records. No details on missing data.	8

For peer review only - http://bmjopen.bmj.com/site/about/guidelines.xhtml

All studies conducted in a specialist / tertiary centre.

PPV and NPV values in italics represent results from case-control studies – these values are misleading in isolation because they assume that the wider prevalence rate of the adverse event is equal to the case to control ratio used in the research study (e.g., if the researchers studied 300 cases and 300 controls, the prevalence rate of adverse events for the calculation of PPV is 50%). As per the cohort studies, prevalence rates of critical events are typically far lower among hospitalised paediatric populations than the case/control ratios used in studies, and so PPV values would be considerably lower in clinical practice.

Studies classified as internal validation if the setting for the study was the same hospital and same research team as those who developed the score. Studies classified as external validation if the score was tested in a different centre and by a different research team to those who developed it.

* Typically, study researchers collected or abstracted multiple PTTT scores for each patient at different time points, but can only use one score per patient for the analysis of the tool's predictive ability. This column specifies which score the researchers used. In most cases, the study team used the maximum PTTT score recorded for each patient in a given study window – e.g., 24 hours prior to a critical event for case patients. The text in parentheses describes the frequency with which scores were assessed or abstracted for each patient, if this information was described in the paper.

† Case-control study, but PPV value calculated based on clinical prevalence of event as measured at local centre during the study

PTTT, paediatric track and trigger tool; S, score; T, trigger; AUROC, area under the receiver operating characteristic curve; PPV, positive predictive value, NPV, negative predictive value; PICU, paediatric intensive care unit; PHDU, paediatric high-dependency unit; RRT, rapid response team; HFNC, high flow nasal cannula; UK, United Kingdom; US, United States; Int, Internal validation; Ext, external validation

For peer review only

Table 4 – summary of early warning system effectiveness study outcome

Outcome	First author, year	Intervention				PTTT	Country	Number of centres	Specialist unit?	Existing RRT / MET?	Population	Study design	Study duration in months	Events before, *n* (rate)	Events after, *n* (rate)	Effect size (95% CI)	P Value	Quality score (max = 26)
		Implemented a new PTTT	Implemented new RRT / MET	Modified escalation process	Staff training / education													
**MORTALITY**																		
Deaths on ward (per 1,000 admissions)	Tibballs 2005[13]	✓	✓		✓	Melbourne Activation Criteria (MAC)	Australia	1	Y	N	All in-patients	Uncontrolled before-after study (prospective)	53 (41 before, 12 after)	13 (0.12)	2 (0.06)	RR = 0.45 (0.10-1.99) †	0.29	10
Hospital-wide deaths (per 100 discharges)	Sharek 2007[17]	✓	✓		✓	Paediatric Rapid Response Team (PRRT) triggering criteria	US	1	Y	N	All in-patients	Uncontrolled before-after study (prospective)	84 (67 before, 17 after)	547 (1.01)	158 (0.83)	RR = 0.82 (0.70-0.95)	**.007**	15
Hospital wide deaths, excluding neonate ICU and ED (per 1,000 discharges)	Zenker 2007[61]	✓	✓			RRT activation criteria*	US	1	Y	N	All in-patients	Uncontrolled before-after study (prospective)	34 (23 before, 11 after)	97 (4.30)	52 (4.45)	RR=1.04 (0.74-1.45) †	.57	12

review only

For peer review only - http://bmjopen.bmj.com/site/about/guidelines.xhtml

Deaths outside ICU (per 1,000 non-ICU patient-days)	Brilli 2007[15]	✓	✓		✓	Paediatric Medical Emergency Team (PMET) triggering criteria (a)	US	1	Y	N	All in-patients	Uncontrolled before-after study (prospective)	27 (15 before, 12 after)	9 (0.10)	2 (0.04)	RR=0.39 (0.08-1.80) †	.13	14
Ward death rate (per 1,000 ward admissions)	Hanson 2010[62]	✓	✓		✓	*Not described*	US	1	Y	N	All in-patients	Uncontrolled before-after study (retrospective)	36 (24 before, 12 after)	13 (1.50)	2 (0.45)	RR = 0.30 (0.07–1.31) †	.07	18
Total hospital deaths (per 1,000 admissions)	Tibballs 2009[63]	✓	✓		✓	Melbourne Activation Criteria (MAC)	Australia	1	Y	N	All in-patients	Uncontrolled before-after study (prospective)	89 (41 before, 48 after)	459 (4.38)	398 (2.87)	RR = 0.65 (0.57-0.75)	**< .0001**	15
Deaths on ward (per 1,000 admissions)	Tibballs 2009[63]	✓	✓		✓	Melbourne Activation Criteria (MAC)	Australia	1	Y	N	All in-patients	Uncontrolled before-after study (prospective)	89 (41 before, 48 after)	13 (0.12)	6 (0.04)	RR = 0.35 (0.13-0.92)	**.03**	15
All-cause hospital mortality (per 1,000 admissions)	Kotsakis 2011[64]	✓	✓			Modified MAC	Canada	4	Y	N	All in-patients	Uncontrolled before-after study (prospective)	48 (24 before, 24 after)	553 (9.97)	540 (9.65)	RR = 0.97 (0.83-1.12)	.65	18
All cause hospital mortality (per 1,000 discharges)	Parshuram 2018[65]	✓	✓		✓	Bedside PEWS	Belgium, Ireland, Netherlands, England, Italy, Canada, New Zealand	21	Y	N	All in-patients	Cluster randomised trial (prospective)	18 (6 pre, 12 post)	Con: 61 (1.31)<br>Int: 52 (1.95)	Con: 147 (1.56)<br>Int: 97 (1.93)	OR=1.01 (0.61-1.69)	.96	23
Hospital mortality (per 1,000 admissions)	Kutty 2018[74]		✓			NR	US	38	Y	N	All in-patients	Interrupted Time Series (retrospective)	180 (60 before, 120 after)	NA	NA	OR=0.94 (0.93-0.95)	.98	20
**PICU MORTALITY**																		

For peer review only - http://bmjopen.bmj.com/site/about/guidelines.xhtml

PICU mortality after PICU admission from ward (per PICU admission)	Anwar-al-Haque, 2010[18]	✓	✓			Paediatric Rapid Response Team (PRRT) triggering criteria (b)	Pakistan	1	Y	N	All in-patients	Uncontrolled before-after study (retrospective)	18 (9 before, 9 after)	23 (51.11)	5 (15.63)	RR = 0.31 (0.13-0.72) †	**.007†**	6
PICU mortality after PICU readmission within 48 hrs of discharge (per 1,000 admissions)	Kotsakis 2011[64]	✓	✓			Modified MAC	Canada	4	Y	N	All in-patients	Uncontrolled before-after study (prospective)	48 (24 before, 24 after)	16 (0.29)	7 (0.13)	RR = 0.43 (0.17-0.99)	**<.05**	18
PICU mortality after urgent PICU admission from ward (per 1,000 admissions)	Kotsakis 2011[64]	✓	✓			Modified MAC	Canada	4	Y	N	All in-patients	Uncontrolled before-after study (prospective)	48 (24 before, 24 after)	70 (1.3)	61 (1.1)	RR = 0.90 (0.70-1.00)	.25	18
Death prior to discharge (per unplanned PICU transfer)	Bonafide 2014[66]	✓	✓			Bedside PEWS	US	1	Y	N	All in-patients	Interrupted Time Series study (prospective)	59 (32 before, 27 after)	51 (6.3)	56 (6.5)	RR = 1.03 (0.72-1.49) †	.99	23
PICU mortality (per PICU admission)	Duns 2014[67]	✓				Between the Flags (BTS) tool*	Australia	1	Y	Y	All in-patients	Uncontrolled before-after study (prospective)	48 (24 before, 24 after)	30 (8.57)	20 (5.49)	RR=0.64 (0.37-1.11) †	.14	7
Death in PICU (per 1,000 patient-days)	Agulnik 2017[68]	✓			✓	Children's Hospital Cardiac Early Warning Score (C-CHEWS)	Guatemala	1	Y	N	Oncology unit	Uncontrolled before-after study (retrospective)	24 (12 before, 12 after)	21 (1.25)	22 (1.10)	RR=0.89 (0.49-1.61) †	.76	19

For peer review only

For peer review only - http://bmjopen.bmj.com/site/about/guidelines.xhtml

Death in PICU (per emergency PICU admission)	Sefton 2015[69]	✓		✓	✓	Modified Bristol PEWT (a)	UK	1	Y	N	All PICU admissions	Controlled before-after study (retrospective)	24 (12 before, 12 after)	17 (10.8)	14 (8.4)	RR = 0.78 (0.40-1.53) †	.47	16
Deaths in PICU (per unplanned PICU admission)	Kolovos, 2018	✓	✓			RRT activation criteria*	US	1	Y	N	All unplanned PICU admissions	Uncontrolled before-after study (retrospective)	78 (42 before, 36 after)	54† (4.9)	40† (3.8)	RR = 0.77 (0.52–1.15) †	.20†	12
PICU mortality (per 1,000 discharges)	Parshuram 2018[65]	✓	✓		✓	Bedside PEWS	Belgium, Ireland, Netherlands, England, Italy, Canada, New Zealand	21	Y	N	All in-patients	Cluster randomised trial (prospective)	18 (6 pre, 12 post)	Con: 34 (0.73)<br>Int: 33 (1.24)	Con: 91 (0.96)<br>Int: 56 (1.12)	OR=0.95 (0.48-1.86)	.88	23
**CARDIAC ARREST**																		
Cardiac arrests on ward (per 1,000 admissions)	Tibballs 2005[13]	✓	✓		✓	Melbourne Activation Criteria (MAC)	Australia	1	Y	N	All in-patients	Uncontrolled before-after study (prospective)	53 (41 before, 12 after)	20 (0.19)	4 (0.11)	RR = 0.58 (0.20-1.70)	.33	10
Cardiopulmonary arrests (per 1,000 non-ICU patient-days)	Brilli 2007[15]	✓	✓		✓	Paediatric Medical Emergency Team (PMET) triggering criteria (a)	US	1	Y	N	All in-patients	Uncontrolled before-after study (prospective)	27 (15 before, 12 after)	7 (0.08)	2 (0.04)	RR=0.50 (0.10-2.42) †	.11	14
Ward cardiac arrest rate (per 1,000 ward admissions)	Hanson 2010[62]	✓	✓		✓	*Not described*	US	1	Y	N	All in-patients	Uncontrolled before-after study (retrospective)	36 (24 before, 12 after)	11 (1.27)	2 (0.45)	RR = 0.35 (0.08–1.58) †	.13	18

For peer review only - http://bmjopen.bmj.com/site/about/guidelines.xhtml

Ward cardiopulmonary arrests (per 1,000 patient-days)	Hunt 2008[16]	✓	✓			Paediatric Medical Emergency Team (PMET) triggering criteria	US	1	Y	N	All in-patients	Uncontrolled before-after study (prospective)	24 (12 before, 12 after)	5 (0.10)	5 (0.10)	RR = 0.98 (0.22–4.24)	.97	17
Preventable cardiac arrests (per 1,000 admissions)	Tibballs 2009[63]	✓	✓		✓	Melbourne Activation Criteria (MAC)	Australia	1	Y	N	All in-patients	Uncontrolled before-after study (prospective)	89 (41 before, 48 after)	17 (0.16)	10 (0.07)	RR = 0.45 (0.20-0.97)	**.04**	15
Unexpected cardiac arrests (per 1,000 admissions)	Tibballs 2009[63]	✓	✓		✓	Melbourne Activation Criteria (MAC)	Australia	1	Y	N	All in-patients	Uncontrolled before-after study (prospective)	89 (41 before, 48 after)	20 (0.19)	24 (0.17)	RR = 0.91 (0.50-1.64)	.75	15
Actual cardiopulmonary arrests (per 1,000 ward admissions)	Kotsakis 2011[64]	✓	✓			Modified MAC	Canada	4	Y	N	All in-patients	Uncontrolled before-after study (prospective)	48 (24 before, 24 after)	69 (1.9)	66 (1.8)	RR = 0.95 (0.76-1.96)	.68	18
Near cardiopulmonary arrests (per 1,000 admissions)	Kotsakis 2011[64]	✓	✓			Modified MAC	Canada	4	Y	N	All in-patients	Uncontrolled before-after study (prospective)	48 (24 before, 24 after)	123 (3.4)	67 (1.9)	RR = 0.54 (0.52-0.57)	**<.001**	18
Cardiac arrests on ward (per 1,000 non-ICU patient-days)	Bonafide 2014[66]	✓	✓			Bedside PEWS	US	1	Y	N	All in-patients	Interrupted Time Series study (prospective)	59 (32 before, 27 after)	6† (0.03)	2† (0.01)	RR = 0.36 (0.07-1.78) †	.21	23
Cardiac arrests (per 1,000 patient-days)	Parshuram 2018[65]	✓	✓		✓	Bedside PEWS	Belgium, Ireland, Netherlands, England, Italy, Canada, New Zealand	21	Y	N	All in-patients	Cluster randomised trial (prospective)	18 (6 pre, 12 post)	Con: 18 (0.11)<br>Int: 15 (0.12)	Con: 32 (0.10)<br>Int: 27 (0.11)	RR=1.02 (0.65-1.62)	.92	23

For peer review only - http://bmjopen.bmj.com/site/about/guidelines.xhtml

**RESPIRATORY ARREST**																		
Ward respiratory arrests (per 1,000 patient-days)	Hunt 2008[16]	✓	✓			Paediatric Medical Emergency Team (PMET) triggering criteria	US	1	Y	N	All in-patients	Uncontrolled before-after study (prospective)	24 (12 before, 12 after)	11 (0.23)	3 (0.06)	RR = 0.27 (0.07-0.95)	**.04**	17
**CARDIAC OR RESPIRATORY ARREST**																		
Cardiac or respiratory arrest (per 1,000 discharges)	Zenker 2007[61]	✓	✓			RRT activation criteria*	US	1	Y	N	All in-patients	Uncontrolled before-after study (prospective)	34 (23 before, 11 after)	180 (7.98)	60 (5.13)	RR=0.64 (0.48-0.86) †	.19	12
Code calls (per 1,000 non-ICU patient-days)	Brilli 2007[15]	✓	✓		✓	Paediatric Medical Emergency Team (PMET) triggering criteria (a)	US	1	Y	N	All in-patients	Uncontrolled before-after study (prospective)	27 (15 before, 12 after)	25 (0.27)	6 (0.11)	RR=0.42 (0.17-1.03) †	.06†	14
Code calls (per 1,000 non-ICU patient-days)	Sharek 2007[17]	✓	✓		✓	Paediatric Rapid Response Team (PRRT) triggering criteria	US	1	Y	N	All in-patients	Uncontrolled before-after study (prospective)	84 (67 before, 17 after)	53 (0.52)	5 (0.15)	RR = 0.29 (0.10-0.65)	**.008**	15
Code calls (per 1,000 admissions)	Anwar-al-Haque 2010[18]	✓	✓			Paediatric Rapid Response Team (PRRT) triggering criteria (b)	Pakistan	1	Y	N	All in-patients	Uncontrolled before-after study (retrospective)	18 (9 before, 9 after)	26 (5.25)	12 (2.73)	RR = 0.52 (0.26-1.03)	.06	6
**CALLS FOR URGENT REVIEW / ASSISTANCE**																		

For peer review only - http://bmjopen.bmj.com/site/about/guidelines.xhtml

Urgent calls to respiratory therapist (per 1,000 patient-days)	Parshuram 2011[71]	✓		✓	✓	Bedside PEWS	Canada	1	N	N	All in-patients	Uncontrolled before-after study (prospective)	8 (3 before, 5 after)	8 (9.5)	8 (3.4)	RR = 0.36 (0.13-0.95) †	**.04†**	23
Urgent calls to paediatrician (per 1,000 patient-days)	Parshuram 2011[71]	✓		✓	✓	Bedside PEWS	Canada	1	N	N	All in-patients	Uncontrolled before-after study (prospective)	8 (3 before, 5 after)	19 (22.6)	12 (5.1)	RR = 0.23 (0.11-0.46) †	**<.0001**	23
Code blue calls on the ward (per 1,000 admissions)	Kotsakis 2011[64]	✓	✓			Modified MAC	Canada	4	Y	N	All in-patients	Uncontrolled before-after study (prospective)	48 (24 before, 24 after)	210 (3.75)	150 (2.70)	RR = 0.71 (0.61-0.83)	**<.0001**	18
Urgent calls to outreach team (per 1,000 admissions)	Duns 2014[67]	✓				Between the Flags (BTS) tool*	Australia	1	Y	Y	All in-patients	Uncontrolled before-after study (prospective)	48 (24 before, 24 after)	1,058 (39.5)	2,120 (76.0)	RR=1.92 (1.79-2.07) †	**.02**	7
RRT calls (per 1,000 patient-days)	Panesar 2014[72]			✓		Modified Brighton PEWS (e)	US	1	Y	Y	All in-patients	Uncontrolled before-after study (retrospective)	42 (18 before, 24 after)	44 (3.14)	69 (4.23)	RR = 1.35 (0.92-1.96) †	.11	15
RRT calls (per 1,000 patient days)	Douglas 2016[73]	✓		✓	✓	Modified Brighton PEWS (b)	US	1	Y	Y	All in-patients	Uncontrolled before-after study (retrospective)	24 (12 before, 12 after)	194 (6.17)	292 (9.80)	RR = 1.59 (1.33-.1.90) †	**<.001**	12
Code calls (per 1,000 patient days)	Douglas 2016[73]	✓		✓	✓	Modified Brighton PEWS (b)	US	1	Y	Y	All in-patients	Uncontrolled before-after study (retrospective)	24 (12 before, 12 after)	31 (0.98)	20 (0.67)	RR = 0.68 (0.39-1.19) †	.21	12
**PICU TRANSFERS**																		

For peer review only

For peer review only - http://bmjopen.bmj.com/site/about/guidelines.xhtml

Transfers from ward to other specialist units (per 1,000 patient-days)	Parshuram 2011[71]	✓		✓	✓	Bedside PEWS	Canada	1	N	N	All in-patients	Uncontrolled before-after study (prospective)	8 (3 before, 5 after)	5 (5.9)	19 (8.1)	RR = 1.37 (0.51-3.63) †	.54†	23
Clinical deterioration events on ward prior to transfer to specialist unit (per 1,000 patient-days)	Parshuram 2011[71]	✓		✓	✓	Bedside PEWS	Canada	1	N	N	All in-patients	Uncontrolled before-after study (prospective)	8 (3 before, 5 after)	2 (2.4)	1 (0.43)	RR = 0.18 (0.02-1.97) †	.16†	23
PICU transfers (per 1,000 admissions)	Duns 2014[67]	✓				Between the Flags (BTS) tool*	Australia	1	Y	Y	All in-patients	Uncontrolled before-after study (prospective)	48 (24 before, 24 after)	350 (13.1)	364 (13.1)	RR=1.00 (0.86-1.16) †	.98	7
Unplanned PICU transfers from ward (per 1,000 non-ICU patient-days)	Bonafide 2014[66]	✓	✓			Bedside PEWS	US	1	Y	N	All in-patients	Interrupted Time Series study (prospective)	59 (32 before, 27 after)	874 (4.54)	936 (5.25)	IRR = 0.73 (0.46–1.14)	.16	23
Unplanned transfers to PICU from ward (per 1,000 patient-days)	Agulnik 2017[68]	✓			✓	Children's Hospital Cardiac Early Warning Score (C-CHEWS)	Guatemala	1	Y	N	Oncology unit	Uncontrolled before-after study (retrospective)	24 (12 before, 12 after)	157 (9.3)	130 (6.5)	RR = 0.70 (0.56-0.88) †	**.003**	19
Urgent PICU admissions (per 1,000 patient-	Parshuram 2018[65]	✓	✓		✓	Bedside PEWS	Belgium, Ireland, Netherlands,	21	Y	N	All in-patients	Cluster randomised trial (prospective)	18 (6 pre, 12 post)	Con: 652 (4.01)	Con: 1178 (3.83)	RR=0.95 (0.82-1.09)	.45	23

For peer review only

days)							England, Italy, Canada, New Zealand							Int: 469 (3.62)	Int: 828 (3.29)			
**PICU OUTCOMES**																		
Critical deterioration events after PICU transfer (per 1,000 non-ICU patient-days)	Bonafide 2014[66]	✓	✓			Bedside PEWS	US	1	Y	N	All in-patients	Interrupted Time Series study (prospective)	59 (32 before, 27 after)	260† (1.35)	282† (1.58)	IRR = 0.38 (0.20-0.75)	**.01**	23
Mechanical ventilation within 1hr of unplanned PICU transfer (per unplanned transfer to PICU)	Bonafide 2014[66]	✓	✓			Bedside PEWS	US	1	Y	N	All in-patients	Interrupted Time Series study (prospective)	59 (32 before, 27 after)	45 (5.1)	42 (4.5)	RR = 0.87 (0.58-1.31) †	.51	23
Mechanical ventilation within 12hrs of unplanned PICU transfer (per unplanned transfer to PICU)	Bonafide 2014[66]	✓	✓			Bedside PEWS	US	1	Y	N	All in-patients	Interrupted Time Series study (prospective)	59 (32 before, 27 after)	112 (12.8)	103 (11.0)	IRR = 0.17 (0.07-0.44)	**<0.001**	23
Vasopressor within 1hr of unplanned PICU transfer (per unplanned transfer to PICU)	Bonafide 2014[66]	✓	✓			Bedside PEWS	US	1	Y	N	All in-patients	Interrupted Time Series study (prospective)	59 (32 before, 27 after)	41 (4.7)	16 (1.7)	RR = 0.36 (0.21-0.64) †	**<0.001**	23

For peer review only

For peer review only - http://bmjopen.bmj.com/site/about/guidelines.xhtml

Vasopressors within 12hrs of unplanned PICU transfer (per unplanned transfer to PICU)	Bonafide 2014[66]	✓	✓			Bedside PEWS	US	1	Y	N	All in-patients	Interrupted Time Series study (prospective)	59 (32 before, 27 after)	71 (8.1)	57 (6.1)	IRR = 0.20 (0.06-0.62)	**.006**	23
Invasive ventilation in PICU (per emergency PICU admission)	Sefton 2015[69]	✓		✓	✓	Modified Bristol PEWT (a)	UK	1	Y	N	All PICU admissions	Controlled before-after study (retrospective)	24 (12 before, 12 after)	118 (75.2)	104 (62.7)	RR = 0.83 (0.72-0.97) †	**.002**	16
Inotropes in PICU (per emergency PICU admission)	Sefton 2015[69]	✓		✓	✓	Modified Bristol PEWT (a)	UK	1	Y	N	All PICU admissions	Controlled before-after study (retrospective)	24 (12 before, 12 after)	50 (31.8)	40 (24.1)	RR = 0.76 (0.53-1.08) †	.12	16
Intubation within 24hrs of PICU admission (per 1,000 patient-days)	Agulnik 2017[68]	✓			✓	Children's Hospital Cardiac Early Warning Score (C-CHEWS)	Guatemala	1	Y	N	Oncology unit	Uncontrolled before-after study (retrospective)	24 (12 before, 12 after)	11 (0.65)	18 (0.90)	RR=1.38 (0.65-2.92) †	.46	19
Vasopressors within 24hrs of PICU admission (per 1,000 patient-days)	Agulnik 2017[68]	✓			✓	Children's Hospital Cardiac Early Warning Score (C-CHEWS)	Guatemala	1	Y	N	Oncology unit	Uncontrolled before-after study (retrospective)	24 (12 before, 12 after)	29 (1.72)	37 (1.86)	RR=1.08 (0.66-1.75) †	.60	19
Mechanical ventilation during PICU admission (per PICU admission)	Kolovos 2018[70]	✓	✓			RRT activation criteria*	US	1	Y	N	All unplanned PICU admissions	Uncontrolled before-after study (retrospective)	78 (42 before, 36 after)	285 (25.98)	233 (22.09)	RR = 0.85 (0.73-0.99) †	**.03†**	12

For peer review only

Intubation within 1hr of PICU admission (per PICU admission)	Kolovos 2018[70]	✓	✓			RRT activation criteria*	US	1	Y	N	All unplanned PICU admissions	Uncontrolled before-after study (retrospective)	78 (42 before, 36 after)	49 (4.47)	88 (8.34)	RR = 1.87 (1.33-2.62)	**.0003**	12
Significant clinical deterioration events (per 1,000 patient-days)	Parshuram 2018[65]	✓	✓		✓	Bedside PEWS	Belgium, Ireland, Netherlands, England, Italy, Canada, New Zealand	21	Y	N	All in-patients	Cluster randomised trial (prospective)	18 (6 pre, 12 post)	Con: 144 (0.89)<br>Int: 80 (0.62)	Con: 259 (0.84)<br>Int: 127 (0.50)	RR=0.77 (0.61-0.97)	**.03**	23

A critical deterioration event is defined as transfer to the intensive care unit (ICU) followed by non-invasive or invasive mechanical ventilation or vasopressor infusion within 12 hours[66]

*Indicates a PTTT not described or validated in the published literature

† Data calculated by research team, based on data presented in the journal article. All data calculated via https://www.medcalc.org.

PTTT, paediatric track and trigger tool; RRT, rapid response team; MET, medical emergency team; PICU, paediatric intensive care unit; RR, relative risk; OR, odds ratio; ED, emergency department

For peer review only

For peer review only - http://bmjopen.bmj.com/site/about/guidelines.xhtml

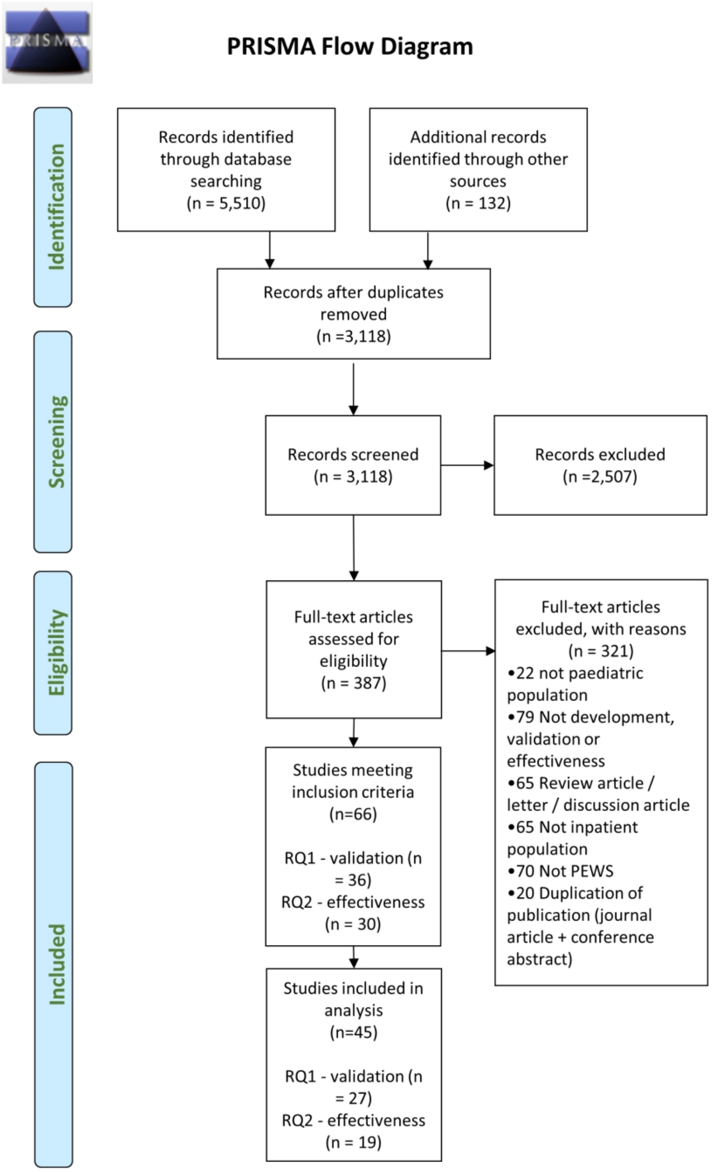

Figure 1: PRISMA flow diagram of study inclusion

190x254mm (300 x 300 DPI)

Supplementary Table 1 – Details of search strategy

Database Search

The search was across a range of databases from their inception to January 2015 then an update was carried out in September 2016 and the second update May 2018.

A preliminary search strategy was developed using a set of key papers known to the group for Ovid Medline using both text words and Medical subject headings. The search strategy was modified according to the indexing systems of the other databases.

Databases and Database platform	Original search results January 2015	Update September 2016	Update May 2018
British Nursing Index (Proquest)	19	12	25
CINAHL (Cumulative Index of Nursing and Allied Health Literature) (Ebsco)	206	17	29
Cochrane Central Register of Controlled Trials (Wiley)	43	4	30
EMBASE (OVID)	1065	206	431
HMIC (Health Management Information Centre) (OVID)	70	1	75
Medline (OVID)	943	135	328
Medline in Process (OVID)	43	69	45
Scopus (Elsevier)	747	85	234
Web of Knowledge (Science Science Citation Indexes) (Thomson Reuter)	400	82	166
**Total**	**3536** *(prior to removing duplicates and irrelevant studies)*	**611** *(prior to removing duplicates and irrelevant studies)*	**1363** *(prior to removing duplicates and irrelevant studies)*

Supplementary search

For peer review only

PUMA Search Information

Supplementary search

NB. Restricted each of the below searches by dates: 01/01/2016 – 16/05/2018

Trials Registers	Hits January 2015	Update September 2016	Update June 2018
ClinicalTrials.gov https://clinicaltrials.gov/	6	4	0
UK Clinical Trials Gateway http://www.ukctg.nihr.ac.uk/default.aspx	3 (duplicates)	5 (1 duplicate)	0
The WHO trial search portal for studies worldwide: http://apps.who.int/trialsearch	1 (duplicate)	0	0
**Journal site**	**Hits**		
Archives of Disease in Childhood http://adc.bmj.com/	14	4	7
BMJ http://www.bmj.com/theBMJ	1	0	1
BMJ Quality and safety http://qualitysafety.bmj.com/	7	4	2
JAMA Pediatrics http://archpedi.jamanetwork.com/journal.aspx	1	0	0
Journal of Critical Care http://www.jccjournal.org/	3	1	0
Journal of Pediatrics (American) http://www.jpeds.com/	1	0	2
Journal of Paediatrics and Child Health (Australian) http://onlinelibrary.wiley.com/journal/10.1111/(ISSN)1440-1754	2	2	0

For peer review only

Lancet<br>http://www.thelancet.com/	0	0	0
New England Journal of Medicine<br>http://www.nejm.org/	0	0	0
Pediatrics<br>http://pediatrics.aappublications.org/	6	2	0
Pediatric Critical Care Medicine<br>http://journals.lww.com/pccmjournal/pages/default.aspx	14	6	3
Websites and organisations	HITS		
American Society of Anesthesiologists<br>https://www.asahq.org/	1	0	0
American Academy of Pediatrics<br>http://www.aap.org/en-us/Pages/Default.aspx	1		0
Association of Anaesthetists of Great Britain and Ireland<br>http://www.aagbi.org/	0	0	0
Australian Medical Council<br>http://www.amc.org.au/	1	0	0
Royal College of Paediatrics and Child Health<br>http://www.rcpch.ac.uk/	1	0	4
Paediatric Nursing Association Europe<br>http://www.rcn.org.uk/	9		0
European Federation of Critical Care Nursing Associations<br>http://www.efccna.org/	No Search Option	No Search Option	No Search Option
Royal Australasian College of Physicians (Division of Child Health)<br>https://www.racp.edu.au/page/paed-policy	0	0	0
Royal College of Physicians (inclusive of National Clinical Guideline Centre)<br>https://www.rcplondon.ac.uk/	2	0	0
The NHS Institute for Innovation and Improvement<br>http://www.institute.nhs.uk/	4	Site cease to exist	Site cease to exist
NICE: Eyes on Evidence	4	1	1

For peer review only

https://www.evidence.nhs.uk/about-evidence-services/bulletins-and-alerts/eyes-on-evidence			
**TOTAL**	82	30	20

Total = 112

Search Strategies

BNI
"Paediatric Early Warning" OR ("pediatric early warning" OR "pediatric rapid response") OR ("paediatric rapid response" OR "Bedside paediatric early warning") OR ("Pediatric Advanced Warning Score" OR "Paediatric Advanced Warning Score")

CENTRAL
Search Name: PUMA update
Last Saved: 16/05/2018 11:39:08.703
Description:

ID	Search
#1	"early warning score*"
#2	"early warning system*"
#3	"early warning tool*"
#4	"VitalPAC Early Warning Score"
#5	"activation criteria"
#6	"Rapid Response Team"
#7	"Rapid Response system*"
#8	"Track and trigger"
#9	"trigger tools"
#10	"calling criteria"

For peer review only

#11	"Alert criteria"
#12	"Rapid Response"
#13	#1 or #2 or #3 or #4 or #5 or #6 or #7 or #8 or #9 or #10 or #11 or #12
#14	pediatric* or paediatric* or infant* or child* or baby or toddler or babies or teen* or adolescent*
#15	#13 and #14
#16	"Pediatric Early Warning"
#17	"Paediatric Early Warning"
#18	"p?ediatric alert"
#19	"Pediatric Rapid Response"
#20	"Pediatric Advanced Warning Score*"
#21	"Paediatric Advanced Warning Score*"
#22	"infant early warning"
#23	"Bedside PEWS"
#24	"Bedside paediatric early warning"
#25	#16 or #17 or #18 or #19 or #20 or #21 or #22 or #23 or #24
#26	#15 or #25 Publication Year from 2016 to 2018

CINAHL via EBSCO

	Search ID#	Search Terms
☐	S11	S7 OR S10
☐	S10	S1 AND S8
☐	S9	S2 AND S8
☐	S8	S3 AND S4
☐	S7	S5 OR S6
☐	S6	TX "infant early warning" OR TX "bedside PEWS" OR TX "Bedside paediatric early warning"
☐	S5	TX "p?ediatric early warning system" OR TX "P?ediatric Early Warning" OR TX "p?ediatric early warning score" OR TX "p?ediatric risk of mortality" OR TX "P?ediatric Rapid Response Team" OR TX "P?ediatric alert"
☐	S4	AB pediatric* or paediatric* or infant*1 or child* or baby or toddler or babies or teen* or adolescent*

For peer review only

☐	S3	TX "track-and-trigger" OR TX "VitalPAC Early Warning Score" OR TX "activation criteria". OR TX "trigger tool*" OR TX "Rapid Response" OR TX "activation criteria". OR TX "early warning" OR TX "Alert criteria" OR TX outreach N3 emergency
☐	S2	Detecting W3 deterioration
☐	S1	"early warning"

DARE
(Paediatric early warning) OR (pediatric early warning) OR (Paediatric Rapid Response) IN DARE
(early warning) OR (track-and-trigger system) OR (Rapid Response) IN DARE
(emergency team) AND (early warning) IN DARE

Embase
Database: EMBASE <1947-Present>
Search Strategy:
----
1 ("early warning" adj5 scor*).ab,ti. (568)
2 ("early warning" adj5 system* adj5 (deteriorat* or mortality or death or outcome* or harm* or safety)).ab,ti. (51)
3 "acute illness severity".mp. (38)
4 early intervention/ and ((prevent* or reduc* or improv*) adj5 (deteriorat* or mortality or death or outcome* or harm* or safety)).ab,ti. (1185)
5 ("early medical intervention" adj5 (tool* or scor* or index* or indicator* or indice* or assessment* or guide* or instrument* or criteria or parameter* or deteriorat* or mortality or death or monitor* or outcome* or harm* or safety)).ab,ti. (10)
6 *"severity of illness index"/ and ((tool* or scor* or index* or indicator* or indice* or assessment* or instrument* or criteria or parameter*) adj5 ((prevent* or reduc* or improv*) adj5 (deteriorat* or mortality or death or outcome* or harm* or safety))).ab,ti. (3)
7 exp Health Status Indicators/ and ((tool* or scor* or index* or indicator* or indice* or assessment* or instrument* or criteria or parameter*) adj3 ((prevent* or reduc* or improv*) adj3 (deteriorat* or mortality or death or outcome* or harm* or safety))).ab,ti. (7)
8 rapid response team/ (849)
9 "alarm monitor"/ and (prevent* or reduc* or improv*).mp. (245)
10 ("clinical alarm" adj5 (prevent* or reduc* or improv*)).mp. (2)
11 (outreach adj3 emergency).tw. (46)
12 VitalPAC Early Warning Score.tw. (15)
13 medical emergency team.tw. (395)
14 Rapid Response Systems.mp. (140)

For peer review only

15 ("rapid response" adj5 (prevent* or reduc* or improv*)).tw. (191)
16 ("medical device" adj3 (prevent* or reduc* or improv*)).mp. (187)
17 (((Detecting or managing) adj3 deterioration) and warning).tw. (11)
18 track-and-trigger system.tw. (24)
19 (Track adj trigger).tw. (4)
20 (Track and trigger).tw. (241)
21 trigger tools.tw. (47)
22 ("alert criteria" or "activation criteria" or "calling criteria").tw. (209)
23 SBAR technique*.mp. (5)
24 (score adj3 severity of illness).tw. (393)
25 or/1-24 (4295)
26 limit 25 to (infant <to one year> or child <unspecified age> or preschool child <1 to 6 years> or school child <7 to 12 years> or adolescent <13 to 17 years>) (533)
27 P?ediatric Early Warning.mp. (120)
28 p?ediatric alert.tw. (7)
29 p?ediatric early warning systems.mp. (4)
30 p?ediatric risk of mortality.tw. (527)
31 P?ediatric Rapid Response Team.tw. (14)
32 Point-of-Care Systems/ and ((paediatric or pediatric) adj3 (improve or identify or detect* or outcome or early or critical or emergency)).tw. (23)
33 P?ediatric Advanced Warning Score.tw. (3)
34 neonatal early warning.tw. (1)
35 infant early warning.tw. (0)
36 p?ediatric rapid response.tw. (31)
37 Bedside paediatric early warning.tw. (5)
38 Bedside PEWS.tw. (7)
39 or/27-38 (707)
40 26 or 39 (1155)
41 limit 40 to human (1065)

HMIC
Database: HMIC Health Management Information Consortium
Search Strategy:

For peer review only

1 ("early warning" adj5 scor*).ab,ti. (23)
2 ("early warning" adj5 system* adj5 (deteriorat* or mortality or death or outcome* or harm* or safety)).ab,ti. (6)
3 "acute illness severity".mp. (3)
4 "early medical intervention"/ and ((prevent* or reduc* or improv*) adj5 (deteriorat* or mortality or death or outcome* or harm* or safety)).ab,ti. (0)
5 ("early medical intervention" adj5 (tool* or scor* or index* or indicator* or indice* or assessment* or guide* or instrument* or criteria or parameter* or deteriorat* or mortality or death or monitor* or outcome* or harm* or safety)).ab,ti. (0)
6 Health Status Indicators.mp. and ((tool* or scor* or index* or indicator* or indice* or assessment* or instrument* or criteria or parameter*) adj3 ((prevent* or reduc* or improv*) adj3 (deteriorat* or mortality or death or outcome* or harm* or safety))).ab,ti. (0)
7 exp "Severity of illness index"/ and ((tool* or scor* or index* or indicator* or indice* or assessment* or instrument* or criteria or parameter*) adj5 ((prevent* or reduc* or improv*) adj5 (deteriorat* or mortality or death or outcome* or harm* or safety))).ab,ti. (0)
8 "activation criteria".ab,ti. (2)
9 exp Rapid response teams/ (39)
10 Clinical Alarms.mp. (0)
11 (outreach adj3 emergency).tw. (2)
12 VitalPAC Early Warning Score.tw. (0)
13 medical emergency team.tw. (15)
14 Rapid Response Systems.mp. (8)
15 Rapid Response Team.tw. (27)
16 ((Detecting or managing) adj3 deterioration).tw. (1)
17 track-and-trigger system.tw. (2)
18 (Track adj trigger).tw. (1)
19 (Track and trigger).tw. (8)
20 trigger tools.tw. (4)
21 Calling criteria.tw. (1)
22 Alert criteria.mp. (1)
23 Rapid response.tw. (111)
24 (score adj3 severity of illness).tw. (3)
25 or/1-24 (171)
26 (pediatric* or paediatric* or infant*1 or child* or baby or toddler or babies or teen* or adolescent*).mp. (40161)
27 25 and 26 (14)
28 p?ediatric alert.tw. (0)
29 p?ediatric early warning systems.mp. (1)
30 p?ediatric risk of mortality.tw. (4)

For peer review only

31 Pediatric Rapid Response Team.tw. (0)
32 Point-of-Care.mp. and ((paediatric or pediatric) adj3 (improve or identify or detect* or outcome or early or critical or emergency)).tw. (0)
33 Pediatric Advanced Warning Score.tw. (0)
34 neonatal early warning.tw. (0)
35 infant early warning.tw. (0)
36 paediatric rapid response.tw. (1)
37 pediatric rapid response.tw. (0)
38 Bedside paediatric early warning.tw. (0)
39 Bedside PEWS.tw. (0)
40 p?ediatric early warning.mp. (2)
41 care.mp. and ((paediatric or pediatric) adj3 (improve or identify or detect* or outcome or early or critical or emergency)).tw. [mp=title, other title, abstract, heading words] (57)
42 or/28-41 (59)
43 27 or 42 (70)

For peer review only

Medline
Database: Ovid MEDLINE(R) <1946 to January Week 2 2015>
Search Strategy:
----
1 ("early warning" adj5 scor*).ab,ti. (260)
2 ("early warning" adj5 system* adj5 (deteriorat* or mortality or death or outcome* or harm* or safety)).ab,ti. (24)
3 "acute illness severity".mp. (21)
4 "early medical intervention"/ and ((prevent* or reduc* or improv*) adj5 (deteriorat* or mortality or death or outcome* or harm* or safety)).ab,ti. (99)
5 ("early medical intervention" adj5 (tool* or scor* or index* or indicator* or indice* or assessment* or guide* or instrument* or criteria or parameter* or deteriorat* or mortality or death or monitor* or outcome* or harm* or safety)).ab,ti. (7)
6 exp Health Status Indicators/ and ((tool* or scor* or index* or indicator* or indice* or assessment* or instrument* or criteria or parameter*) adj3 ((prevent* or reduc* or improv*) adj3 (deteriorat* or mortality or death or outcome* or harm* or safety))).ab,ti. (166)
7 "Severity of Illness Index"/ and ((tool* or scor* or index* or indicator* or indice* or assessment* or instrument* or criteria or parameter*) adj5 ((prevent* or reduc* or improv*) adj5 (deteriorat* or mortality or death or outcome* or harm* or safety))).ab,ti. (274)
8 exp Hospitals/ and ((Detecting or managing) adj3 deterioration).tw. (2)
9 ("medical device" adj3 (prevent* or reduc* or improv*)).mp. (58)
10 ("alert criteria" or "activation criteria" or "calling criteria").tw. (121)

11 Hospital Rapid Response Team/ (334)
12 Clinical Alarms/ (332)
13 (outreach adj3 emergency).tw. (32)
14 VitalPAC Early Warning Score.tw. (10)
15 medical emergency team.tw. (247)
16 Rapid Response Systems.mp. (87)
17 Rapid Response Team.tw. (185)
18 (((Detecting or managing) adj3 deterioration) and warning).tw. (8)
19 track-and-trigger system.tw. (14)
20 (Track adj trigger).tw. (2)
21 (Track and trigger).tw. (137)
22 trigger tools.tw. (22)
23 SBAR technique*.mp. (3)
24 ("rapid response" adj5 (prevent* or reduc* or improv*)).tw. (117)
25 (score adj3 severity of illness).tw. (243)
26 or/1-25 (2286)
27 limit 26 to (humans and "all child (0 to 18 years)") (453)
28 P?ediatric Early Warning.mp. (38)
29 p?ediatric alert.tw. (5)
30 p?ediatric early warning systems.mp. (3)
31 p?ediatric risk of mortality.tw. (400)
32 P?ediatric Rapid Response Team.tw. (6)
33 Point-of-Care Systems/ and ((paediatric or pediatric) adj3 (improve or identify or detect* or outcome or early or critical or emergency)).tw. (79)
34 P?ediatric Advanced Warning Score.tw. (2)
35 neonatal early warning.tw. (0)
36 infant early warning.tw. (0)
37 p?ediatric rapid response.tw. (20)
38 Bedside paediatric early warning.tw. (2)
39 Bedside PEWS.tw. (2)
40 or/28-39 (542)
41 27 or 40 (943)

Scopus

For peer review only

(TITLE-ABS-KEY ("Paediatric Early Warning" OR "Pediatric Early Warning" OR "Pediatric Advanced Warning Score" OR "Paediatric Advanced Warning Score" OR "neonatal early warning" OR "infant early warning" OR "pediatric rapid response" OR "Paedatric rapid response")) OR (((TITLE-ABS-KEY ("early warning" W/5 scor*)) OR (TITLE-ABS-KEY ("Rapid Response")) OR (TITLE-ABS-KEY ("track-and-trigger system")) OR (TITLE-ABS-KEY ("track and trigger")) OR (TITLE-ABS-KEY ("trigger tool*")) OR (TITLE-ABS-KEY ("alert criteria")) OR (TITLE-ABS-KEY ("activation criteria")) OR (TITLE-ABS-KEY ("VitalPAC Early Warning Score"))) AND (TITLE-ABS-KEY (pediatric* OR paediatric* OR infant* OR child* OR baby OR toddler OR babies OR teen* OR adolescent*))) AND (LIMIT-TO (SUBJAREA , "MEDI") OR LIMIT-TO (SUBJAREA , "NURS") OR LIMIT-TO (SUBJAREA , "NEUR"))

Web of Science

# 19	400	#17 OR #1<br>**Refined by:** [excluding] **WEB OF SCIENCE CATEGORIES:** (PARASITOLOGY OR PUBLIC ENVIRONMENTAL OCCUPATIONAL HEALTH OR BIOCHEMISTRY MOLECULAR BIOLOGY OR OPTICS OR HEALTH CARE SCIENCES SERVICES OR MYCOLOGY OR MANAGEMENT OR LINGUISTICS OR INSTRUMENTS INSTRUMENTATION OR MICROBIOLOGY OR INFORMATION SCIENCE LIBRARY SCIENCE OR MATHEMATICAL COMPUTATIONAL BIOLOGY OR GERIATRICS GERONTOLOGY OR ENGINEERING BIOMEDICAL OR FOOD SCIENCE TECHNOLOGY OR ENVIRONMENTAL STUDIES OR ENGINEERING ENVIRONMENTAL OR ENGINEERING ELECTRICAL ELECTRONIC OR HEALTH POLICY SERVICES OR TOXICOLOGY OR EDUCATION EDUCATIONAL RESEARCH OR NUTRITION DIETETICS OR SUBSTANCE ABUSE OR ECONOMICS OR MEDICINE RESEARCH EXPERIMENTAL OR STATISTICS PROBABILITY OR DEVELOPMENTAL BIOLOGY OR MEDICAL INFORMATICS OR SOCIOLOGY OR DENTISTRY ORAL SURGERY MEDICINE OR PSYCHOLOGY EXPERIMENTAL OR COMPUTER SCIENCE ARTIFICIAL INTELLIGENCE OR METEOROLOGY ATMOSPHERIC SCIENCES OR CHEMISTRY ANALYTICAL OR MEDICAL LABORATORY TECHNOLOGY OR CELL BIOLOGY OR DEMOGRAPHY OR BUSINESS FINANCE OR COMPUTER SCIENCE INTERDISCIPLINARY APPLICATIONS OR AUDIOLOGY SPEECH LANGUAGE PATHOLOGY OR PSYCHOLOGY DEVELOPMENTAL OR COMPUTER SCIENCE INFORMATION SYSTEMS OR PLANNING DEVELOPMENT)<br>Indexes=SCI-EXPANDED, SSCI, CPCI-S, CPCI-SSH Timespan=1900-2015
# 18	499	#17 OR #1<br>Indexes=SCI-EXPANDED, SSCI, CPCI-S, CPCI-SSH Timespan=1900-2015
#	487	#16 AND #15

For peer review only

17		Indexes=SCI-EXPANDED, SSCI, CPCI-S, CPCI-SSH Timespan=1900-2015
# 16	8,044	#14 OR #13 OR #12 OR #11 OR #10 OR #9 OR #8 OR #7 OR #6 OR #5 OR #4 OR #3 OR #2<br>Indexes=SCI-EXPANDED, SSCI, CPCI-S, CPCI-SSH Timespan=1900-2015
# 15	1,689,232	**TOPIC:** ((pediatric* OR paediatric* OR infant* OR child* OR baby OR toddler OR babies OR teen* OR adolescent*))<br>Indexes=SCI-EXPANDED, SSCI, CPCI-S, CPCI-SSH Timespan=1900-2015
# 14	130	**TOPIC:** ("Severity of Illness Index" and ((tool* or scor* or index* or indicator* or indice* or assessment* or instrument* or criteria or parameter*) SAME ((prevent* or reduc* or improv*) SAME (deteriorat* or mortality or death or outcome* or harm* or safety))))<br>Indexes=SCI-EXPANDED, SSCI, CPCI-S, CPCI-SSH Timespan=1900-2015
# 13	63	**TOPIC:** (("early medical intervention" SAME (tool* or scor* or index* or indicator* or indice* or assessment* or guide* or instrument* or criteria or parameter* or deteriorat* or mortality or death or monitor* or outcome* or harm* or safety)))<br>Indexes=SCI-EXPANDED, SSCI, CPCI-S, CPCI-SSH Timespan=1900-2015
# 12	28	**TOPIC:** ("early medical intervention" and ((prevent* or reduc* or improv*) SAME (deteriorat* or mortality or death or outcome* or harm* or safety)))<br>Indexes=SCI-EXPANDED, SSCI, CPCI-S, CPCI-SSH Timespan=1900-2015
# 11	1,206	**TOPIC:** ("early warning" SAME system* SAME (deteriorat* or mortality or death or outcome* or harm* or safety))<br>Indexes=SCI-EXPANDED, SSCI, CPCI-S, CPCI-SSH Timespan=1900-2015
# 10	2	**TOPIC:** ("SBAR technique")<br>Indexes=SCI-EXPANDED, SSCI, CPCI-S, CPCI-SSH Timespan=1900-2015
# 9	7	**TOPIC:** ("VitalPAC Early Warning Score")<br>Indexes=SCI-EXPANDED, SSCI, CPCI-S, CPCI-SSH Timespan=1900-2015
# 8	123	**TOPIC:** ("activation criteria")<br>Indexes=SCI-EXPANDED, SSCI, CPCI-S, CPCI-SSH Timespan=1900-2015
# 7	16	TS=("alert criteria")<br>Indexes=SCI-EXPANDED, SSCI, CPCI-S, CPCI-SSH Timespan=1900-2015
# 6	159	TS=("trigger tool*")<br>Indexes=SCI-EXPANDED, SSCI, CPCI-S, CPCI-SSH Timespan=1900-2015
#	45	TS=("track and trigger")

For peer review only

5 Indexes=SCI-EXPANDED, SSCI, CPCI-S, CPCI-SSH Timespan=1900-2015

15 TS=("track-and-trigger system")

4 Indexes=SCI-EXPANDED, SSCI, CPCI-S, CPCI-SSH Timespan=1900-2015

6,100 TS=("Rapid Response")

3 Indexes=SCI-EXPANDED, SSCI, CPCI-S, CPCI-SSH Timespan=1900-2015

604 TS=("early warning" SAME scor*)

2 Indexes=SCI-EXPANDED, SSCI, CPCI-S, CPCI-SSH Timespan=1900-2015

88 TS=("Paediatric Early Warning" OR "Pediatric Early Warning" OR "Pediatric Advanced Warning Score"

1 OR "Paediatric Advanced Warning Score" OR "neonatal early warning" OR "infant early warning" OR "pediatric rapid response" OR "Paedatric rapid response")

Indexes=SCI-EXPANDED, SSCI, CPCI-S, CPCI-SSH Timespan=1900-2015

PUMA Supplementary searches

Search terms to use:

"Pediatric Early warning"

"Paediatric Early warning"

“Pediatric Rapid Response Team”

“Paediatric Rapid Response Team”

PEWS

“Paediatric trigger tools”

“Pediatric trigger tools”

For peer review only

Supplementary Table 2 - PICOS criteria for inclusion of studies

Question 1 – development / validation studies

Parameter	Inclusion criteria	Exclusion criteria
Patients	Children aged 0-18 who are in-patients in a hospital	Adult patients; children in emergency departments or neonatal unit
Intervention	Development or validation of a PTTT	Acuity or triage tools, tools developed for use in emergency departments
Comparator	Not applicable	
Outcomes	Mortality and critical events including: arrests, code calls, transfer to higher level of care (e.g., ICU/HDU), senior review, RRT/MET activation, acuity at PICU admission and critical interventions on the ward or PICU	
Study design	Chart or case reviews; cohort studies; case-control studies, observational studies	Reviews, editorials or opinion pieces

Question 2 – effectiveness studies

Parameter	Inclusion criteria	Exclusion criteria
Patients	Children aged 0-18 who are in-patients in a hospital	Adult patients<br>Children in emergency departments or neonatal unit
Intervention	Implementation of any 'paediatric early warning system' intervention (with or without a PTTT) – including implementing a new PTTT, RRT/MET implementation, educational initiatives or communications tools aimed at improving identification of deteriorating in-patients	Acuity or triage tools, tools developed for use in emergency departments, interventions whose purpose was not identification of deteriorating in-patients
Comparator	Not applicable	
Outcomes	Mortality and critical events including: arrests, code calls, transfer to higher level of care (e.g., ICU/HDU), senior review, RRT/MET activation, acuity at PICU admission and critical interventions on the ward or PICU	
Study design	Randomised controlled trials, non-randomised controlled trials, before-after studies (controlled or uncontrolled); interrupted time series studies	Reviews, editorials or opinion pieces

For peer review only

Supplementary Table 3 – Template Quality Assessment Forms

QUALITY ASSESSMENT FOR DEVELOPMENT AND VALIDATION STUDIES

Criteria		Yes (2)	Partial (1)	No (0)	N/A	Score
1	Is the hypothesis / aim / objective of the study clearly described?	Easily identified in introduction / method.	Vague / incomplete or found in other parts of paper (than introduction/method)	Aim / Objective no reported		
2	Was the score developed comprehensively?	Evidence base / Expert opinion / Delphi method	Decided within research team	No info / unclear		
3	Are the characteristics of the patients in the study clearly described?	Reproducible criteria used to categorise participants	Poorly define criteria / incomplete information	No baseline / demographic info		
4	Is the study design well described and appropriate?	Well described, easy to find in paper	Design not clearly described / design only partially answers the question	Design poorly described or does not answer study question		
5	Are the study sample representative of the intended population?	A full description of the target population is given with the sample selected in a non-biased manner	Sample selected from a known population however, selection strategy likely introduces bias but not enough to seriously distort results	Sample recruited from an unknown population in an opportunistic fashion		
6	Are population characteristics controlled for and adequately described?	Appropriate control at design/analysis stage	Incomplete control/description or not considered but unlikely to seriously influence results	Not controlled for and likely to seriously influence results		
7	Was compliance/use of the PEWS reliable?	Compliance / use was well described and reliably implemented	Compliance / use was not well described or not reliably implemented	Compliance / use was not reported		
8	Was consideration given for data collected at different times / sites	Well described reason why data was collected at different time points	Data was collected at different times due to specific opportunity	No explanation for data collection at different time points	Data was collected at the same time point	
9	Are the main findings clearly described?	Simple outcome data reported for all major findings	Incomplete or inappropriate descriptive statistics	No/inadequate descriptive statistics		
10	Are methods of analysis adequately described and appropriate?	Described and appropriate	Not reported but probably appropriate or some tests appropriate, some not	Methods not described and cannot be determined		
11	Are the conclusions supported by the results	All conclusions supported by data	Some of the major conclusions are supported by the data; some are not or speculative interpretations are not indicated as such	None/few of major conclusions supported by the data		
12	How was missing data handled	Missing data was reported and handled appropriately	Missing data was reported but unable to determine how it was handled or it wasn't handled appropriately	Missing data was not reported	No missing data	
					**Total**	

MAX. Score: 24

For peer review only

QUALITY ASSESSMENT FOR EFFECTIVENESS STUDIES

Criteria		Yes (2)	Partial (1)	No (0)	N/A	Score
1	Is the hypothesis / aim / objective of the study clearly described?	Easily identified in introduction / method.	Vague / incomplete or found in other parts of paper (than introduction/method)	Aim / Objective no reported		
2	Was the score developed comprehensively?	Evidence base / Expert opinion / Delphi method	Decided within research team	No info / unclear		
3	Are the characteristics of the patients in the study clearly described?	Reproducible criteria used to categorise participants	Poorly define criteria / incomplete information	No baseline / demographic info		
4	Is the study design well described and appropriate?	Well described, easy to find in paper	Design not clearly described / design only partially answers the question	Design poorly described or does not answer study question		
5	Are the study sample representative of the intended population?	A full description of the target population is given with the sample selected in a non-biased manner	Sample selected from a known population however, selection strategy likely introduces bias but not enough to seriously distort results	Sample recruited from an unknown population in an opportunistic fashion		
6	Was the PEWS well implemented?	Implementation was well reported and appropriately applied	Implementation was not well reported or not appropriate	No info / unclear		
7	Are population characteristics controlled for and adequately described?	Appropriate control at design/analysis stage	Incomplete control/description or not considered but unlikely to seriously influence results	Not controlled for and likely to seriously influence results		
8	Was compliance/use of the PEWS reliable?	Compliance / use was well described and reliably implemented	Compliance / use was not well described or not reliably implemented	Compliance / use was not reported		
9	Was consideration given for data collected at different times / sites	Well described reason why data was collected at different time points	Data was collected at different times due to specific opportunity	No explanation for data collection at different time points	Data was collected at the same time point	
10	Are the main findings clearly described?	Simple outcome data reported for all major findings	Incomplete or inappropriate descriptive statistics	No/inadequate descriptive statistics		
11	Are methods of analysis adequately described and appropriate?	Described and appropriate	Not reported but probably appropriate or some tests appropriate, some not	Methods not described and cannot be determined		
12	Are the conclusions supported by the results	All conclusions supported by data	Some of the major conclusions are supported by the data; some are not or speculative interpretations are not indicated as such	None/few of major conclusions supported by the data		
13	How was missing data handled	Missing data was reported and handled appropriately	Missing data was reported but unable to determine how it was handled or it wasn't handled appropriately	Missing data was not reported	No missing data	
					**Total**	

MAX. Score: 26

For peer review only

Supplementary Table 4 –Validation papers excluded from analysis

PTTT	First author, year	Country	Study population	Study design	Number of centres	PTTT used in practice?	Internal / external validation study?	Outcome measures	Sample size	Score or trigger?	Study overview and reason for exclusion from validation results	Quality score (max = 24)
Modified Brighton PEWS (a)	Garlick 2013[20]	US	All in-patients (MET calls only)	Case-control study (retrospective)	1	N	Ext	Transfer to PICU	267 (116 cases)	S	Describes review of MET calls (n=267) to evaluate predictive ability of Modified Brighton PEWS tool for identifying children requiring transfer to PICU (n=116). Results presented in terms of association between PEWS and odds of transfer to higher level of care – no evaluation of performance characteristics such as AUROC, sensitivity or specificity.	8
	Medar 2015[21]	Unclear	RRT calls only	Chart review (retrospective)	1	NR	Ext	RRT call	61	S	Describes retrospective review of RRT calls (n=61) to evaluate Modified Brighton PEWS at time of admission and time of RRT call. Report higher median PEWS score for patients at time of RRT call compared to admission. No evaluation of performance characteristics such as AUROC, sensitivity or specificity.	6
Texas Children's Hospital (TCH) PAWS	Bell 2013[22]	US	General medical ward & two specialist units	Chart review (retrospective)	1	Y	Int	Other validated scales (e.g., Glasgow Coma Scale)	150	S	Describes development and implementation of the TCH PAWS tool in three wards of a specialist paediatric unit in the US. TCH PAWS amended locally from the Brighton PEWS. Reports on internal reliability (correlation coefficients between 3 categories of the score) and inter-rater reliability of scoring among nurses. Also compares scores on sub-categories to other measures, e.g., the Behavioural sub-score is compared to the Glasgow Coma Scale. No evaluation of performance characteristics such as AUROC, sensitivity or specificity.	12
Cardiac Children's Hospital Early Warning Score (C-CHEWS)	McLellan 2013[23]	US	Cardiac unit	Tool development	1	Y	Int	Cardiac ICU transfer	27	S	Describes the development and implementation of a modified version of the Children's Hospital Early Warning score for cardiac patients. Results focus on tool modification and implementation challenges – no evaluation of performances characteristics such as AUROC, sensitivity or specificity. Validation of the tool described in a separate paper.	9
Burn-specific PEWS	Rahman 2014[24]	US	Specialist burn unit	Chart review (retrospective)	1	Y	Int	Burn injuries	50	S	Conference abstract only. Describes development and implementation of a modified version of the Brighton PEWS, for use with in-patients with burn injuries. Analysis of 50 randomly selected charts – results focus on compliance with scoring and relationship between PTTT score and extent of burn injuries. No evaluation of performance characteristics such as AUROC, sensitivity or specificity.	13

Bedside PEWS	Hopkins 2013[25]	US	All in-patients (code blue and RRT calls only)	Chart review (retrospective)	1	N	Ext	PICU transfer and critical intervention in PICU among RRT and code calls	113 (64 cases)	S	Conference abstract only. Describes retrospective chart review of code blue and RRT calls over a year – Bedside PEWS scores calculated and comparisons drawn between patients eventually transferred to PICU and those who stayed on ward. Preliminary analysis given in terms of mean PEWS scores for different groups – no evaluation of performance characteristics such as AUROC, sensitivity or specificity.	6
	Gawronski 2013[26]	Italy	Bone marrow transplant unit	Case-control study (retrospective)	1	N	Ext	Urgent PICU transfer, PICU consult or death	21 (11 cases)	S	Conference abstract only. Describes case-control study evaluating Bedside PEWS in an Italian bone marrow transplant unit, in relation to urgent PICU transfers or consultations. Preliminary analysis only – comparison of mean PTTT scores for cases and controls. No evaluation of performance characteristics such as AUROC, sensitivity or specificity.	6
Bristol PEWT	Haines 2006[12]	UK	All in-patients	Chart review (retrospective)	1	Y	Int	Transfer to PICU or HDU	360 (180 cases)	T	Describes development and piloting of the Bristol PEWT in a UK tertiary centre. Only included children who would have triggered the pilot version of the tool (n=360) and then identified PICU or HDU transfers from this population. Paper presents specificity and sensitivity outcomes but they are incorrectly calculated, so results not included in analysis.	9
Modified Bristol PEWT (a)	Sefton 2014[27]	UK	All in-patients	Chart review (retrospective)	1	Y	Int	Transfer to PICU, cardiac / respiratory arrest or unexpected death	Unclear	T	Conference abstract only. Describes a retrospective review of 5 years of data from locally implemented PTTT in a UK tertiary centre, presenting a multiple regression model identifying seven components (including age) most strongly associated with subsequent adverse event if triggered. Of the six clinical elements, all were associated with increased odds of an adverse event, except nurse concern which was significantly associated with decreased odds of an adverse event. No evaluation of overall PTTT performance characteristics such as AUROC, sensitivity or specificity.	10

PTTT names refer to those described in Table 2

All studies conducted in a specialist / tertiary centre.

Studies classified as internal validation if the setting for the study was the same hospital and same research team as those who developed the score. Studies classified as external validation if the score was tested in a different centre and by a different research team to those who developed it.

PTTT, paediatric track and trigger tool; S, score; T, trigger; AUROC, area under the receiver operating characteristic curve; PPV, positive predictive value, NPV, negative predictive value; PICU, paediatric intensive care unit; PHDU, paediatric high-dependency unit; RRT, rapid response team; HFNC, high flow nasal cannula; UK, United Kingdom; US, United States; Int, Internal validation; Ext, external validation

For peer review only

Supplementary Table 5 – Effectiveness papers excluded from analysis

First author, year	Intervention				PTTT	Country	Number of centres	Specialist unit?	Existing RRT / MET?	Population	Study design	Study duration in months (before & after intervention)	Description and reason for excluding from analysis	Quality score (max = 26)
	Implemented a new PTTT	Implemented new RRT / MET	Modified escalation process	Staff training / education										
Mistry 2006[51]	✓	✓		✓	PRRT activation criteria*	US	1	Y	N	All in-patients	Uncontrolled before-after study (prospective)	11 (6 before, 5 after)	Describes implementation of a PRRT with calling criteria (not defined). Looked at impact on mortality, cardiac arrests and PICU outcomes among PICU transfers. Reports absolute decreases in numbers of deaths and arrests post-intervention, but no denominator data provided or further statistical details given.	3
Demmel 2010[52]	✓				Modified Brighton PEWS (e)	US	1	Y	Y	Haematology / oncology patients	Uncontrolled before-after study (prospective)	Unclear (unclear, 8 after)	Implemented a locally modified version of the Brighton PEWS in a specialist haematology / oncology unit. Discusses challenges in the development and implementation of the tool. Refers to number of days between cardiopulmonary arrests being 299 immediately before implementation, and 1,053 days eight months after implementation – however, no denominator data or further statistical details given.	8
Sandhu 2010[53]		✓			Unclear	UK	1	Y	N	Unclear	Uncontrolled before-after study (retrospective)	Unclear (unclear, 3 months)	Conference abstract only. Reported implementing an ‘outreach response team’ alongside an existing ‘paediatric early warning tool’ (unclear which tool) in a UK tertiary centre. Reference to comparable triggering rate of PTTT before (28% of patients) and after (28% of patients) piloting the outreach team, and 2 arrests before piloting, and 0 after – but no denominator data or further statistical details given.	8
Randhawa 2011[54]	✓		✓	✓	Brighton PEWS	US	1	Y	Y	All in-patients	Uncontrolled before-after study (prospective)	Unclear	Describes implementation of the Brighton PEWS in a specialist paediatric centre. Details various cycles of change during implementation of the tool across different wards, and efforts at staff education. Reports reduction in rate of cardiopulmonary arrests post-intervention, but no absolute numbers, denominator data or further statistical details given.	12

Camacho 2011[55]	✓				Modified Brighton PEWS (a) †	US	1	Y	NR	Cardiac and renal patients	Uncontrolled before-after study (prospective)	8 (3 before, 5 after)	Conference abstract only. Reported piloting and modifying Tucker's modified Brighton PEWS for specialist cardiac and renal population. Unclear if RRT/MET in place. Referred to there being 5 code calls in the quarter (3 months) before implementation, and 0 in the following 5 months. However, no denominator data or further statistical details given.	8
Heyden 2012[56]	✓	✓			PRRT activation criteria*	US	1	Y	N	All in-patients	Uncontrolled before-after study (retrospective)	72 (24 before, 48 after)	Conference abstract only. Describes implementation of an RRT in a US tertiary centre, with an associated 'broad calling criteria' (limited details given). Reports number of cardiac arrests on ward and PICU before and after intervention, and refers to increase in RRT calls over time. No denominator data or further statistical details given.	7
Somberg 2013	✓	✓			Unclear	US	1	N	N	All in-patients	Uncontrolled before-after study (unclear)	Unclear	Conference abstract only. Reported developing and implementing a PTTT (tool not named) and RRT for a paediatric unit in a community hospital. Reference to no intubation or code calls since intervention, but no pre-intervention comparison, time frames, denominator data or further statistical details given.	2
Norville 2013[57]	✓				Texas Children's Hospital (TCH) Paediatric Advanced Warning Score (PAWS)†	US	1	Y	Y	Bone marrow transplant patients	Uncontrolled before-after study (unclear)	23 (12 before, 11 after)	Conference abstract only. Describes implementation of TCH PAWS, with amended algorithm for specialist bone marrow transplant unit. Looked at impact on code calls and RRT calls – refers to 3 code calls and 18 RRT calls pre-intervention, compared to 0 codes and 25 RRT calls post-intervention. No denominator data or further statistical details given.	5
Ambati 2014[58]				✓	Not applicable	US	1	Y	Y	Unclear	Uncontrolled before-after study (unclear)	48 (12 before, 36 after)	Conference abstract only. Reported effect of implementing a "simulation based curriculum" for clinical staff on subsequent RRT utilisation. Reference to increase in RRT calls year on year post implementation, but no denominator data or further statistical details given.	3
Ocholi 2014[59]	✓				Bedside PEWS	UK	1	Y	N	Unclear	Uncontrolled before-after study (unclear)	12 months (6 before, 6 after)	Conference abstract only. Describes implementation of Bedside PEWS in a UK tertiary centre. Looked at impact of intervention on ward outcomes and outcomes of children transferred to PICU. Reference to impact of tool on number of 'adverse incidents' (not defined) on the ward and median length of stay in PICU among PICU transfers, but no denominator data or further statistical details given.	6
Fenix 2016[39]	✓			✓	Unclear	US	1	Y	NR	Two general paediatric wards	Uncontrolled before-after study (retrospective)	46 months (16 before, 30 after)	Conference abstract only. Describes implementation of a 'Situational Awareness' tool, with integrated PTTT (unclear which tool) in a tertiary centre. Retrospective review of rates of Critical Deterioration (CD) events on two of seven general paediatric wards. Reports a significant decrease in trend and trajectory of CD events post-implementation, but no event numbers, denominator data or further statistical details given.	6

For peer review only

PTTT names refer to those described in Table 2

* Indicates PTTT not fully described or validated in the published literature

† PTTT modified by local team, but exact modifications not described

PTTT, paediatric track and trigger tool; RRT, rapid response team; MET, medical emergency team; PICU, paediatric intensive care unit; US, United States; UK, United Kingdom

For peer review only

PRISMA 2009 Checklist

Section/topic	#	Checklist item	Reported on page #
**TITLE**			
Title	1	Identify the report as a systematic review, meta-analysis, or both.	1
**ABSTRACT**			
Structured summary	2	Provide a structured summary including, as applicable: background; objectives; data sources; study eligibility criteria, participants, and interventions; study appraisal and synthesis methods; results; limitations; conclusions and implications of key findings; systematic review registration number.	2-3
**INTRODUCTION**			
Rationale	3	Describe the rationale for the review in the context of what is already known.	5-6
Objectives	4	Provide an explicit statement of questions being addressed with reference to participants, interventions, comparisons, outcomes, and study design (PICOS).	6
**METHODS**			
Protocol and registration	5	Indicate if a review protocol exists, if and where it can be accessed (e.g., Web address), and, if available, provide registration information including registration number.	7
Eligibility criteria	6	Specify study characteristics (e.g., PICOS, length of follow-up) and report characteristics (e.g., years considered, language, publication status) used as criteria for eligibility, giving rationale.	7-8; Supp table 2
Information sources	7	Describe all information sources (e.g., databases with dates of coverage, contact with study authors to identify additional studies) in the search and date last searched.	7
Search	8	Present full electronic search strategy for at least one database, including any limits used, such that it could be repeated.	Supp table 1
Study selection	9	State the process for selecting studies (i.e., screening, eligibility, included in systematic review, and, if applicable, included in the meta-analysis).	7-8
Data collection process	10	Describe method of data extraction from reports (e.g., piloted forms, independently, in duplicate) and any processes for obtaining and confirming data from investigators.	7-8
Data items	11	List and define all variables for which data were sought (e.g., PICOS, funding sources) and any assumptions and simplifications made.	7
Risk of bias in individual studies	12	Describe methods used for assessing risk of bias of individual studies (including specification of whether this was done at the study or outcome level), and how this information is to be used in any data synthesis.	8; Supp table 3
Summary measures	13	State the principal summary measures (e.g., risk ratio, difference in means).	8
Synthesis of results	14	Describe the methods of handling data and combining results of studies, if done, including measures of consistency (e.g., I^2) for each meta-analysis.	NA

For peer review only - http://bmjopen.bmj.com/site/about/guidelines.xhtml

PRISMA 2009 Checklist

Section/topic	#	Checklist item	Reported on page #
Risk of bias across studies	15	Specify any assessment of risk of bias that may affect the cumulative evidence (e.g., publication bias, selective reporting within studies).	NA
Additional analyses	16	Describe methods of additional analyses (e.g., sensitivity or subgroup analyses, meta-regression), if done, indicating which were pre-specified.	NA
**RESULTS**			
Study selection	17	Give numbers of studies screened, assessed for eligibility, and included in the review, with reasons for exclusions at each stage, ideally with a flow diagram.	9
Study characteristics	18	For each study, present characteristics for which data were extracted (e.g., study size, PICOS, follow-up period) and provide the citations.	9, Tables 2-4
Risk of bias within studies	19	Present data on risk of bias of each study and, if available, any outcome level assessment (see item 12).	Tables 2-4
Results of individual studies	20	For all outcomes considered (benefits or harms), present, for each study: (a) simple summary data for each intervention group (b) effect estimates and confidence intervals, ideally with a forest plot.	Tables 3-4
Synthesis of results	21	Present results of each meta-analysis done, including confidence intervals and measures of consistency.	NA
Risk of bias across studies	22	Present results of any assessment of risk of bias across studies (see Item 15).	NA
Additional analysis	23	Give results of additional analyses, if done (e.g., sensitivity or subgroup analyses, meta-regression [see Item 16]).	NA
**DISCUSSION**			
Summary of evidence	24	Summarize the main findings including the strength of evidence for each main outcome; consider their relevance to key groups (e.g., healthcare providers, users, and policy makers).	16-20
Limitations	25	Discuss limitations at study and outcome level (e.g., risk of bias), and at review-level (e.g., incomplete retrieval of identified research, reporting bias).	16-20
Conclusions	26	Provide a general interpretation of the results in the context of other evidence, and implications for future research.	16-20
**FUNDING**			
Funding	27	Describe sources of funding for the systematic review and other support (e.g., supply of data); role of funders for the systematic review.	21

From: Moher D, Liberati A, Tetzlaff J, Altman DG, The PRISMA Group (2009). Preferred Reporting Items for Systematic Reviews and Meta-Analyses: The PRISMA Statement. PLoS Med 6(7): e1000097. doi:10.1371/journal.pmed1000097

For more information, visit: www.prisma-statement.org.

For peer review only - http://bmjopen.bmj.com/site/about/guidelines.xhtml

PRISMA 2009 Checklist

For peer review only

BMJ Open

Validity and effectiveness of paediatric early warning systems and track and trigger tools for identifying and reducing clinical deterioration in hospitalised children: a systematic review

Journal:	*BMJ Open*
Manuscript ID	bmjopen-2018-022105.R3
Article Type:	Research
Date Submitted by the Author:	07-Mar-2019

Complete List of Authors:	Trubey, Rob; Cardiff University, Centre for Trials Research<br>Huang, Chao; Cardiff University, Centre for Trials Research<br>Lugg-Widger, Fiona; Cardiff University, Centre for Trials Research<br>Hood, Kerenza; Cardiff University, Centre for Trials Research<br>Allen, Davina; Cardiff University, School of Healthcare Sciences<br>Edwards, Dawn; Morriston Hospital, Department of Paediatrics<br>Lacy, David; Wirral Unviersity Teaching Hospital<br>Lloyd, Amy; Cardiff University, Centre for Trials Research<br>Mann, Mala; Cardiff University, University Library Services<br>Mason, Brendan; Public Health Wales<br>Oliver, Alison; Noah's Ark Children's Hospital for Wales, Department of Paediatric Intensive Care<br>Roland, Damian; Paediatric Emergency Medicine Leicester Academic (PEMLA) Group, Emergency Department<br>Sefton, G.; Alder Hey Childrens NHS Fdn Trust,<br>Skone, Richard; Noah's Ark Children's Hospital for Wales, Department of Paediatric Intensive Care<br>Thomas-Jones, Emma; Cardiff University, Centre for Trials Research<br>Tume, Lyvonne N.; University of the West of England Bristol, Faculty of Health and Applied Sciences (HAS)<br>Powell, Colin; Children's Hospital for Wales, Paediatrics; Sidra Medical and Research Center, Department of Pediatric Emergency Medicine

**Primary Subject Heading**:	Paediatrics
Secondary Subject Heading:	Paediatrics, Nursing, Intensive care, Health policy, Diagnostics
Keywords:	Quality in health care < HEALTH SERVICES ADMINISTRATION & MANAGEMENT, Paediatric intensive & critical care < INTENSIVE & CRITICAL CARE, PAEDIATRICS

SCHOLARONE™
Manuscripts

Validity and effectiveness of paediatric early warning systems and track and trigger tools for identifying and reducing clinical deterioration in hospitalised children: a systematic review

Authors: Rob Trubey[1†], Chao Huang[1], Fiona Lugg-Widger[1], Kerenza Hood[1], Davina Allen[2], Dawn Edwards[3], David Lacy[4], Amy Lloyd[1], Mala Mann[5], Brendan Mason[6], Alison Oliver[7], Damian Roland[8], Gerri Sefton[9], Richard Skone[7], Emma Thomas-Jones[1], Lyvonne Tume[9], Colin Powell[10,11]

1 Centre for Trials Research, Cardiff University, Heath Park, Cardiff, UK
2 School of Healthcare Sciences, Cardiff University, Eastgate House, Cardiff, UK
3 Department of Paediatrics, Morriston Hospital, Swansea, UK
4 Wirral University Teaching Hospital, Wirral, UK
5 Specialist Unit for Review Evidence (SURE), Cardiff University, Heath Park, Cardiff, UK
6 Public Health Wales, Cardiff, UK
7 Department of Paediatric Intensive Care, Children's Hospital for Wales, Cardiff, UK
8 Paediatric Emergency Medicine Leicester Academic (PEMLA) Group, Emergency Department, Infirmary Square, Leicester, UK
9 Paediatric Intensive Care Unit, Alder Hey Children's NHS Foundation Trust, Liverpool, UK
10 Department of Child Health, Children's Hospital for Wales, Division of Population Medicine School of Medicine, Cardiff University, Cardiff, UK,
11 Department of Pediatric Emergency Medicine, Sidra Medicine, Doha, Qatar

Corresponding author:
Dr. Rob Trubey, Centre for Trials Research, Cardiff University, Heath Park, Cardiff, UK
trubeyrj@cf.ac.uk

Keywords: PEWS, track and trigger scores, early warning scores, clinical deterioration, children, systematic review

For peer review only - http://bmjopen.bmj.com/site/about/guidelines.xhtml

ABSTRACT

Objective

To assess (1) how well validated existing paediatric track and trigger tools (PTTT) are for predicting adverse outcomes in hospitalised children, and (2) how effective broader paediatric early warning systems are at reducing adverse outcomes in hospitalised children.

Design

Systematic review.

Data sources

British Nursing Index, CINAHL, Cochrane Central Register of Controlled Trials, Database of Abstracts of Reviews of Effectiveness, EMBASE, HMIC, Medline, Medline in Process, Scopus and Web of Knowledge searched through May 2018.

Eligibility criteria

We included (1) papers reporting on the development or validation of a PTTT or (2) the implementation of a broader early warning system in paediatric units (age 0-18), where adverse outcome metrics were reported. Several study designs were considered.

Data extraction and synthesis

Data extraction was conducted by two independent reviewers using template forms. Studies were quality assessed using a modified Downs and Black rating scale.

Results

36 validation studies and 30 effectiveness studies were included, with 27 unique PTTT identified. Validation studies were largely retrospective case-control studies or chart reviews, while effectiveness studies were predominantly uncontrolled before-after studies. Metrics of adverse outcomes varied considerably. Some PTTT demonstrated good diagnostic accuracy in retrospective case-control studies (primarily for predicting PICU transfers) but positive predictive value was consistently low, suggesting potential for alarm fatigue. A small number of effectiveness studies reported significant decreases in mortality, arrests or code calls, but were limited by methodological concerns. Overall, there was limited evidence of paediatric early warning system interventions leading to reductions in deterioration.

For peer review only

Conclusion

There are several fundamental methodological limitations in the PTTT literature, and the predominance of single-site studies carried out in specialist centres greatly limits generalisability. With limited evidence of effectiveness, calls to make PTTT mandatory across all paediatric units are not supported by the evidence base.

PROSPERO registration number: CRD42015015326.

For peer review only

For peer review only - http://bmjopen.bmj.com/site/about/guidelines.xhtml

STRENGTHS AND LIMITATIONS OF THIS STUDY

- Paediatric early warning systems and paediatric track and trigger tools (PTTT) are increasingly used by paediatric units across Europe, North America, Australia and elsewhere – this study is a timely review of the evidence for their validity and effectiveness
- A comprehensive search was carried out across multiple databases and included published as well as grey literature
- The review highlights methodological weaknesses and gaps in the current evidence base and makes suggestions for future research
- Heterogeneity in study populations, study designs and outcome measures make it difficult to compare and synthesise findings across the wide range of early warning systems and PTTT being used in practice
- The review is limited in scope to quantitative validation and effectiveness studies, so must be considered alongside wider literature reflecting on potential secondary benefits of early warning systems and PTTT for communication, teamwork and empowerment

For peer review only

For peer review only - http://bmjopen.bmj.com/site/about/guidelines.xhtml

BACKGROUND

Failure to recognise and respond to clinical deterioration in hospitalised children is a major safety concern in healthcare. The underlying causes of this problem are clearly multi-factorial[1–3] but paediatric ‘early warning systems’ have been strongly advocated as one approach to improving recognition of deterioration in paediatric units[1,2,4].

A paediatric ‘early warning system’ can be considered any patient safety initiative or programme which aims to monitor, detect and respond to signs of deterioration in hospitalised children in order to avert adverse outcomes and premature death. Such systems are often multi-faceted and may include the use of rapid response teams (RRT) or medical emergency teams (MET), education or training to improve clinical staff’s ability to identify deterioration or strategies aimed at improving staff communication and situational awareness.

An increasingly commonplace paediatric ‘early warning system’ initiative is the use of a ‘track and trigger tool’: these tools, also commonly used in adult care, provide a formal framework for evaluating routine physiological, clinical and observational data for early indicators of patient deterioration. They are typically integrated into routine observation charts or electronic health records and compare patient observations to pre-defined ‘normal’ thresholds. When one or more observation is considered abnormal, staff are directed to various clinical actions, including but not limited to altered frequency of observations, review by senior staff or more appropriate treatment or management. Tools may be paper based or electronic and monitoring may be automated or manually undertaken by staff.

These tools have been referred to in the literature using a number of different terms: paediatric early warning scores (PEWS); paediatric early warning tools (PEWT), track and trigger tools (TTT) and many others. Here, we refer to the tools themselves using the term ‘paediatric track and trigger tools’ (PTTT). A variety of PTTT have been developed, typically by teams based in specialist paediatric centres and often used as a means of triggering a dedicated response team. Their advocacy has recently led to widespread uptake across a variety of different paediatric units, including many non-specialist centres where patient populations and resources may differ. In the United Kingdom (UK), a recent cross-sectional survey found that 85% of paediatric units were using some form of PTTT, most of which were non-specialist centres without a dedicated response team[5]. Despite their widespread use, recent reviews have questioned the evidence-base for their effectiveness in improving patient outcomes [6,7]. The current review aimed to build on this work, assessing in depth the evidence

For peer review only - http://bmjopen.bmj.com/site/about/guidelines.xhtml

base for both the validity of PTTT for predicting in-patient deterioration and the effectiveness of broader ‘early warning systems’ at reducing instances of mortality and morbidity in paediatric settings:

- Question 1: How well validated are existing paediatric track and trigger tools (PTTT) and their component parts for predicting in-patient deterioration?
- Question 2: How effective are paediatric early warning systems (with or without a PTTT) at reducing mortality and critical events?

For peer review only

For peer review only - http://bmjopen.bmj.com/site/about/guidelines.xhtml

METHODS

This systematic review is reported in accordance with the Preferred Reporting Items for Systematic Review and Meta-Analyses (PRISMA) guidelines[8]. Our review protocol is registered with the PROSPERO database CRD42015015326.

Search strategy

A comprehensive search was conducted across a range of databases to identify relevant studies in the English language. Published and unpublished literature was considered where publicly available, as were studies in press. The following databases were searched through May 2018: British Nursing Index, CINAHL (Cumulative Index of Nursing and Allied Health Literature), Cochrane Central Register of Controlled Trials, Database of Abstracts of Reviews of Effectiveness, EMBASE, HMIC (Health Management Information Centre), Medline, Medline in Process, Scopus and Web of Knowledge (Science Citation Indexes). To identify additional papers, published, unpublished or research reported in the grey literature a range of relevant websites and trial registers were searched including Clinical Trials.gov. To identify published papers that had not yet been catalogued in the electronic databases, recent editions of key journals were hand-searched. The search terms included 'early warning scores', 'alert criteria', 'rapid response', 'track and trigger' and 'early medical intervention'. (Supplementary Table 1)

Eligibility screening and study selection

PICOS parameters guided inclusion criteria for the validation and effectiveness studies (Supplementary Table 2). Papers reporting development of validation of a PTTT were included for Question 1, whereas papers reporting the implementation of any broader 'paediatric early warning system' (with or without a PTTT) were eligible for Question 2. Both research questions were limited to studies that involved in-patients aged 0-18. Outcome measures considered were mortality and critical events, including: unplanned admission to a higher level of care, cardiac arrest, respiratory arrest, medical emergencies requiring immediate assistance, children reviewed by Paediatric Intensive Care Unit (PICU) staff on the ward (in specialist centres) or reviewed by external PICU staff (for non-specialist centres), acuity at PICU admission and PICU outcomes. A range of study designs were considered for both questions.

For peer review only - http://bmjopen.bmj.com/site/about/guidelines.xhtml

Two of the review authors independently screened the titles and abstracts yielded in the search. Full texts were reviewed independently by six reviewers against the above eligibility criteria and were assigned to the relevant review question if included. Reasons for exclusion were recorded. Separate data extraction forms were developed for validation and effectiveness studies. The forms had common elements (study design, country, setting, study population, description of the PTTT or early warning system, statistical techniques used, outcomes assessed). Additional data items for validation studies included the items in the PTTT, modifications to the PTTT from previous versions, predictive ability of individual items and the overall tool, sensitivity and specificity and inter and intra-rater reliability. Effectiveness studies included an assessment of outcomes in terms of mortality and various morbidity variables. Data extraction was carried out by two reviewers and discrepancies were resolved by discussion. For effectiveness studies, effect sizes and 95% confidence intervals (CI) were calculated or reported as risk ratios (RR) or odds ratios (OR) as appropriate, with p-values reported to assess statistical significance. Data analysis was conducted using an online medical statistics tool.

Quality appraisal

Methodological quality and risk of bias was assessed for each included study using a modified version of the Downs and Black rating scale[9] (templates shown in Supplementary Table 3).

Patient and Public Involvement

This review was conducted as part of a larger mixed-methods study (ISRCTN94228292), which used a formal, facilitated parental advisory group. The group comprised parents of children who had experienced an unexpected adverse event in a paediatric unit and provided input which helped to shape the broader research questions and outcome measures. The results of the review will be disseminated to parents through this group.

For peer review only - http://bmjopen.bmj.com/site/about/guidelines.xhtml

REVIEW RESULTS

Figure 1 shows the PRISMA flow diagram for both research questions.

[FIGURE 1]

Study characteristics

Table 1 summarises the study characteristics of the 36 validation (Question 1) and 30 effectiveness (Question 2) papers included in the review.

Validation studies (n=36)			Effectiveness studies (n=30)		
	N	%		n	%
**Type**			**Type**		
Full text	22	61.1	Full text	21	70.0
Abstract	14	38.9	Abstract	9	30.0
**Country**			**Country**		
United States	15	41.7	United States	18	60.0
United Kingdom	12	33.3	United Kingdom	3	10.0
Canada	2	5.5	Canada	2	6.7
Australia	0	0.0	Australia	3	10.0
Other	5	13.9	Other	3	10.0
Multiple	1	2.8	Multiple	1	3.3
Unclear	1	2.8	Unclear	0	0.0
**Year of study**			**Year of study**		
Pre-2012	10	27.8	Pre-2012	15	50.0
2012	3	8.3	2012	1	3.3
2013	6	16.7	2013	2	6.7
2014	5	13.9	2014	6	20.0
2015	7	19.4	2015	0	0.0
2016	2	5.6	2016	2	6.7
2017	3	8.3	2017	1	3.3
2018	0	0.0	2018	3	10.0
**Setting**			**Setting**		
Specialist / tertiary	33	91.7	Specialist / tertiary	29	96.7
Non-specialist / community	0	0.0	Non-specialist / community	1	3.3
Unclear	3	8.3	Unclear	0	0.0
**Single / multi-centre**			**Single / multi-centre**		
Single-centre	35	97.2	Single-centre	28	93.3
Multi-centre	1	2.8	Multi-centre	2	6.7
**Study population**			**Study population**		
General in-patients	23	63.9	General in-patients	20	66.6
Specialist population	11	30.6	Specialist population	5	16.7
Unclear	2	5.6	Unclear	5	16.7
**Study design**			**Study design**		
Case-control	18	50.0	Uncontrolled before-after	26	86.7

For peer review only

For peer review only - http://bmjopen.bmj.com/site/about/guidelines.xhtml

Case / chart review	10	27.8	Controlled before-after	1	3.3
Cohort	7	19.4	Interrupted Time Series	2	6.7
Pilot study	1	2.8	Cluster randomised trial	1	3.3

Table 1: Summary study characteristics of validation and effectiveness papers in the review

Types of PTTS and components

Across 66 studies, we identified 27 unique PTTT (Table 2). Twenty PTTTs were based on one of four different tools: Monaghan's Brighton PEWS[10], the Bedside PEWS[11], the Bristol PEWT[12] and the Melbourne Activation Criteria[13]. Other PTTT described in the literature included the National Health Service Institute for Innovation and Improvement (NHS III) PEWS[14] (the second most commonly used PTTT in United Kingdom paediatric settings[5]), RRT and MET activation criteria[15–18], and one prediction algorithm developed from a large dataset of electronic health data[19].

[TABLE 2]

Table 2 illustrates the range of physiological and behavioural parameters underpinning PTTT. Common parameters included heart rate (present in 26 out of 27 PTTT), respiratory rate (24), respiratory effort (24) and level of consciousness or behavioural state (24). All PTTT required at least six different parameters to be collected.

Question 1 – How well validated are PTTT and component parts for predicting in-patient deterioration?

Nine validation papers meeting inclusion criteria were excluded from analysis: eight did not report any performance characteristics of the PTTT for predicting deterioration[20–27] and one study calculated incorrect sensitivity/specificity outcomes[12] (Supplementary Table 4). The remaining 27 validation studies, evaluating the performance of 18 unique PTTT, are described in Table 3. Four studies evaluated multiple PTTTs[3,19,28,29] and one paper described three separate studies of the same PTTT[30].

[TABLE 3]

Five cohort studies were included[14,31–34], three based on the same dataset. All other studies were either case-control or chart reviews. Thirteen papers implemented the PTTT in practice[23,30,31,34–43], while the remaining studies 'bench tested' the PTTT – researchers retrospectively calculated the score based on data abstracted from medical charts and records. All studies were conducted in specialist centres with only one multi-centre study reported[44].

Outcome measures

For peer review only - http://bmjopen.bmj.com/site/about/guidelines.xhtml

PTTT were evaluated for their ability to predict a wide range of clinical outcomes. Composite measures were used in eight studies[14,23,29,32,33,37,45,46], cardiac/respiratory arrest or a "code call" was used (singularly or part of a composite outcome) in six studies[23,28,29,37,45,47], while 22 studies used transfer to a to PICU or Paediatric High-Dependency Unit (PHDU) as the main outcome[3,11,19,23,28–34,36,37,39,41–44,46,48,49].

Predictive ability of individual PTTT components

Three validation papers reported on the performance characteristics of individual components of the tool for predicting adverse outcomes[11,33,42]. Parshuram and colleagues, for instance, reported Area Under the Receiver Operating Characteristic curve (AUROC) values for individual PTTT items of a pilot version of the Bedside PEWS: ranging from 0.54 (bolus fluid) to 0.81 (heart rate), compared to 0.91 for the overall PTTT[11]. All other studies reported outcomes for the PTTT as a whole.

Paediatric Early Warning System (PEWS) score

The predictive ability of the 16-item PEWS score was assessed by one internal[47] (AUROC=0.90) and two external case-control studies[28,29] (AUROC range =0.82-0.88) with a range of outcome measures and scoring thresholds. One case-control study used an observed prevalence rate to calculate a positive predictive value (PPV) of 4.2% for the tool in predicting code calls[47] (for every 1,000 patients triggering the PTTT, 42 would be expected to deteriorate).

Bedside PEWS and derivatives

The Bedside PEWS was evaluated in one internal[11] (AUROC=0.91) and five external case-control studies[19,28,29,44,46] (AUROC range=0.73-0.90) for a range of different outcome measures and at different scoring thresholds. One case-control study calculated a PPV of 2.1% for identifying children requiring urgent PICU transfer within 24 hours of admission, based on locally observed prevalence rates[19]. A modified version of the Bedside PEWS (with temperature added) demonstrated an AUROC of 0.86 in an external case-control study with a composite outcome of death, arrest or unplanned PICU transfer[29].

Brighton PEWS and derivatives

Six different PTTT based on the original Brighton PEWS were evaluated across 11 studies[19,29,31,37,39–42,45,48,50]. The Modified Brighton PEWS (a) was evaluated for its ability to predict PICU transfers in one large prospective cohort study (AUROC=0.92, PPV=5.8%)[31],

For peer review only - http://bmjopen.bmj.com/site/about/guidelines.xhtml

and an external case-control study tested the same score for predicting urgent PICU transfers within 24 hours of admission (AUROC=0.74, PPV= 2.1%)[19].

An external case-control study used a composite measure of death, arrest or PICU transfer to evaluate the Modified Brighton PEWS (b) (AUROC=0.79) and the Modified Brighton PEWS (d) (AUROC=0.74)[29]. The latter tool was evaluated in a further internal case-control study for predicting PICU transfer (AUROC=0.82) [48].

The Children's Hospital Early Warning Score (CHEWS) had a reported AUROC of 0.90 for predicting PICU transfers or arrests in a large internal case-control study[50]. A modification for cardiac patients, the Cardiac CHEWS (C-CHEWS) was evaluated by one internal study on a cardiac unit[37] (AUROC = 0.90) looking at arrests or unplanned PICU transfers, and two external studies of oncology / haematology units[41,42] for the same outcome (AUROC=0.95). Finally, the Children's Hospital Los Angeles (CHLA) PEWS was evaluated by in a small internal case-control study for prediction of re-admission to PICU after initial PICU discharge[40] (AUROC=0.71).

Melbourne Activation Criteria (MAC) and derivatives

The MAC was assessed by one external case-control study with an outcome of death, arrest or unplanned PICU transfer[29] (AUROC=0.71) and a large external cohort study with an outcome of death or unplanned PICU or HDU transfer[33] (AUROC=0.79, PPV=3.6%). A derivative of the MAC using an aggregate score, the Cardiff & Vale PEWS (C&VPEWS), was tested using the same cohort and outcome measures in an earlier external study (AUROC=0.86, PPV=5.9%)[32] and was the best performing PTTT in an external case-control study evaluating multiple PTTT[29] (AUROC=0.89).

Bristol PEWT

The Bristol PEWT was evaluated by five external validation studies: two chart review studies[3,35] (no AUROC), one small cohort study of PICU transfers[34] (AUROC=0.91, PPV=11%), and two case-control studies looking at code calls[28] (AUROC=0.75) and a composite of death, arrests and PICU transfers[29] (AUROC=0.62).

Other PTTT

The NHS Institute for Improvement and Innovation (NHS III) PEWS was tested by one external cohort study looking at a composite of death or unplanned transfers to PICU or HDU[14] (AUROC=0.88, PPV=4.3%) and one external case-control study looking at a

For peer review only - http://bmjopen.bmj.com/site/about/guidelines.xhtml

For peer review only

composite of death, arrests and PICU transfers[29] (AUROC=0.82). Zhai and colleagues developed and retrospectively evaluated a logistic regression algorithm in an internal case-control study looking at urgent PICU transfers in the first 24 hours of admission[19] (AUROC =0.91, PPV=4.8%).

Across PTTT, studies reporting performance characteristics of a tool at a range of different scoring thresholds demonstrate the expected interaction and trade-off between sensitivity and specificity – at lower triggering thresholds, sensitivity is high but specificity is low; at higher thresholds, the opposite is true.

Inter-rater reliability and completeness of data

Accurate assessment of the ability of a PTTT to predict clinical deterioration is contingent on accuracy and reliability of tool scoring (whether by bedside nurses in practice or by researchers abstracting data) and the availability of underpinning observations. Only five papers made reference to accuracy or reliability of scoring[28,31,37,42,45], with mixed results: for example, two nurses separately scoring a sub-set of patients on the Modified Brighton PEWS (a) achieved an intra-class coefficient of 0.92[31], but a study nurse and bedside nurse achieved only 67% agreement in scoring the C-CHEWS tool[37]. Completeness of data was reported in 11 studies[11,14,19,29,30,32,33,42,44,45,47]. An evaluation of the Modified Bedside PEWS (a) reported that "the PEWS was correctly performed and could be used for inclusion in the study" in 59% of cases[30], a prospective study bench-testing the C&VPEWS found an average completeness rate of 44% for the seven different parameters in daily practice[32], while a multi-centre study of the Bedside PEWS reported that "only 5.1% [of observation sets] had measurements on all 7 items"[44].

Question 2 – how effective are early warning systems at reducing mortality and critical events in hospitalised children?

Eleven papers meeting inclusion criteria were excluded from analysis for providing insufficient statistical information (e.g., denominator data, absolute numbers of events) to calculate effect sizes[39,51–59]. Further details on papers excluded from analysis are provided in Supplementary Table 5. Findings from the 19 studies included in the analysis are summarised in Table 4.

[TABLE 4]

Type of early warning system interventions

For peer review only - http://bmjopen.bmj.com/site/about/guidelines.xhtml

Seventeen interventions involved the introduction of a new PTTT[13,15–18,60–72], one intervention introduced a mandatory triggering element to an existing PTTT[71], and one study reported a large, multi-centre analysis of MET introduction with no details on PTTT use[73]. Twelve interventions included the introduction of a new MET or RRT[13,15–18,60–65,69], while four further interventions introduced a new PTTT in a hospital with an existing MET or RRT. Only three studies therefore evaluated a PTTT in the absence of a dedicated response team[67,68,70]. A staff education programme was explicitly described in ten interventions[13,15,17,61,62,64,67,68,70,72].

Of the 18 studies that used a PTTT, only seven used a tool that had been formally evaluated for validity: three used the Bedside PEWS[64,65,70], two used the MAC[13,62], one used the Modified Brighton PEWS (b)[72] and one used the C-CHEWS[67]. One study did not report the PTTT used[61], while ten studies used a variety of calling criteria and local modifications to validated tools that had not been evaluated for validity[15–18,60,63,66,68,69,71].

Mortality (ward or hospital wide)

Two uncontrolled before-after studies (both with MET/RRT) reported significant mortality rate reductions post intervention: one in hospital wide deaths per 100 discharges[17] (RR=0.82, 95% CI=0.70-0.95) and one in total hospital deaths per 1,000 admissions (RR=0.65, 0.57-0.75) and deaths on the ward ('unexpected deaths') per 1,000 admissions[62] (RR=0.35, 0.13-0.92). Seven studies found no reductions in mortality, including two high quality multi-centre studies[13,15,60,63–65,73]. Parshuram and colleagues conducted a cluster randomised trial and found no difference in all-cause hospital mortality rates between 10 hospitals randomly selected to receive an intervention centred around use of the Bedside PEWS and 11 usual care hospitals, one year post intervention (OR=1.01, 0.61-1.69)[64]. Kutty *et al.* assessed the impact of MET implementation in 38 US paediatric hospitals with an interrupted time series study, and reported no difference in the slope of hospital mortality rates five years post intervention and the expected slope based on pre-implementation trends (OR = 0.94, 0.93-0.95)[73].

PICU mortality

Two uncontrolled before-after studies (both with MET/RRT) reported a significant post-intervention reduction in rates of PICU mortality among ward transfers (RR=0.31, 0.13-0.72)[18], and PICU mortality rates among patients readmitted within 48 hours (RR=0.43, 0.17-0.99)[63]. Six studies (including a high quality cluster randomised trial and interrupted time

For peer review only - http://bmjopen.bmj.com/site/about/guidelines.xhtml

series study) reported no post-intervention change in PICU mortality using a variety of metrics[64–69].

Cardiac and respiratory arrests

Two uncontrolled before-after studies (both with RRT/MET) reported significant post-intervention rate reductions in sub-categories of cardiac arrests: one in 'near cardiopulmonary arrests'[63] (RR=0.54, 0.52-0.57) but not 'actual cardiopulmonary arrests' and one in 'preventable cardiac arrests'[62] (RR=0.45, 0.20-0.97) but not 'unexpected cardiac arrests'. One uncontrolled before-after study (with RRT/MET) reported a significant post intervention reduction in rates of ward respiratory arrests per 1,000 patient-days[16] (RR=0.27, 0.07-0.95). Seven studies (including one high quality cluster randomised trial and one high quality interrupted time series study) found no change in cardiac arrest rates using a variety of metrics[13,15,16,61,64,65] or cardiac and respiratory arrests combined[60].

Calls for urgent review / assistance

Two uncontrolled before-after studies (all with RRT/MET) reported significant post-intervention reductions in rates of code calls[17,63] (RR=0.29, 0.10-0.65; RR=0.71, 0.61-0.83) while three studies found no change in rates of code calls[15,18,72]. One uncontrolled before-after study in a community hospital (without RRT/MET) found significant post intervention reductions in rates of urgent calls to the in-house paediatrician (RR=0.23, 0.11-0.46) and respiratory therapist[70] (RR=0.36, 0.13-0.95). Two uncontrolled before-after studies (with RRT/MET) found increases in rates of RRT calls[72] (RR=1.59, 1.33-1.90) and outreach team calls[66] (RR=1.92, 1.79-2.07). One study found no change in rates of RRT calls[71].

PICU transfers

One uncontrolled before-after study (without RRT/MET) found a significant post-intervention decrease in the rate of unplanned PICU transfers per 1,000 patient-days[67] (RR=0.70, 0.56-0.88). Four studies (including one high quality cluster randomised trial and one high quality interrupted time series study) found no change in rates of PICU admissions post intervention[64–66,70].

PICU outcomes

Two studies, one interrupted time series and one multi-centre cluster randomised trial (both with RRT/MET), found significant reductions in rates of 'critical deterioration events' (life-sustaining interventions administered within 12 hours of PICU admission) relative to pre-

For peer review only - http://bmjopen.bmj.com/site/about/guidelines.xhtml

implementation trends and relative to control hospitals respectively (IRR=0.38, 0.20-0.75; OR=0.77, 0.61-0.97)[64,65]. One controlled before-after study (without RRT/MET) reported a significant reduction in rates of invasive ventilation given to emergency PICU admissions post intervention (RR=0.83, 0.72-0.97) with no significant change observed in a control group of patients admitted to PICU from outside of the hospital[68]. One uncontrolled before-after study reported a significant post-intervention decrease in rates of PICU admissions receiving mechanical ventilation (RR=0.85, 0.73-0.99) but an increase in rates of early intubation (RR=1.87, 1.33-2.62)[69].

Implementation outcomes

Only three studies reported outcomes relating to the quality of implementation of the intervention. One study reported 99% of audited observation sets of the Bedside PEWS had at least 5 vital signs present post-intervention, up from 76% pre-intervention (no change in control hospitals)[64]. A previous study of the same PTTT reported 3% of audited cases had used the incorrect age chart but reported an intra-class coefficient of 0.90 for agreement between bedside nurses scoring the PTTT in practice and research nurses retrospectively assigned scores[70]. Finally, error rates in C-CHEWS scoring were reported to have reduced from an initial 47% to below 10% by the end of the study[67].

DISCUSSION

This paper reviewed the published PTTT and early warning system literature in order to assess the validity of PTTT for predicting in-patient deterioration (Question 1) and the effectiveness of early warning system interventions (with or without PTTT) for reducing mortality and morbidity outcomes in hospitalised children (Question 2). We believe that the consideration of broader ‘early warning systems’ differentiates this paper from previous reviews, as does the inclusion of two recently published high-quality effectiveness studies[64,73].

How well validated are existing tools for predicting in-patient deterioration?

Given a growing understanding and emphasis on the importance of local context in healthcare interventions, it is perhaps not surprising that such a wide range of PTTT have been developed and evaluated internationally, and modifications to existing PTTT are common. The result, however, is that a large number of different PTTT have been narrowly validated, but none have been broadly validated across a variety of different settings and

For peer review only

populations. With only one exception[44], all studies evaluating the validity of PTTT have been single-centre reports from specialist units, greatly limiting the generalisability of the findings.

PTTT such as the Bedside PEWS, C&VPEWS, NHS III PEWS and C-CHEWS have demonstrated very good (AUROC ≥0.80) or excellent (AUROC ≥ 0.90) diagnostic accuracy, typically for predicting PICU transfers, in internal and external validation studies[11,14,19,29,32,37,42,44]. However, methodological issues common to the validation studies mean that such results need to be interpreted with a degree of caution. Firstly, each of the studies was conducted in a clinical setting where paediatric in-patients are subject to various forms of routine clinical intervention throughout their admission. There are numerous statistical modelling techniques which can account for co-occurrence of clinical interventions and the longitudinal nature of the predictors[74,75], but none of these were used in the validation studies and so estimates of predictive ability are likely to be distorted. Indeed, the majority of outcomes used in the validation studies are clinical interventions themselves (e.g., PICU transfer). Secondly, while it understandable that a majority of studies 'bench-tested' the PTTT rather than implement it into practice before evaluation, the process of abstracting PTTT scores retrospectively from patient charts and medical records introduces a number of sources of potential bias or inaccuracy. For instance, several studies reported either high levels of missing data (i.e., some of the observations required to populate the PTTT score being evaluated were not routinely collected or recorded and so were scored as 'normal')[11,19,32,44,45] or difficulty in abstracting certain descriptive or subjective PTTT components[19,28,41,49]. Assuming missing values are normal, or excluding some PTTT items for analysis are both likely to result in underscoring of the PTTT and skew the results. Finally, studies which evaluated a PTTT that had been implemented in practice are at risk of overestimating the ability of PTTT to predict proxy outcomes such as PICU transfer, inasmuch as high PTTT scores or triggers automatically direct staff towards escalation of care, or clinical actions which make escalation of care more likely.

The findings reported in several PTTT studies point towards two potential challenges for some centres in implementing and sustaining a PTTT in clinical practice. As noted above, a number of studies that retrospectively 'bench-tested' a PTTT reported that the observations that were required to score the tool were not always routinely collected or recorded in their centre. It may be that the introduction of a PTTT into practice would help create a framework to ensure that core vital signs and observations were collected more routinely (as demonstrated by Parshuram and colleagues[64]) but this would obviously have resource

For peer review only - http://bmjopen.bmj.com/site/about/guidelines.xhtml

implications that could be a potential barrier for some centres. Such considerations are important, as evidence from the adult literature points to the potential for tools to inadvertently mask deterioration when core observations are missing[76]. Secondly, PPV values reported in cohort studies, and case-control studies that adjusted for outcome prevalence, were uniformly low (between 2.3%-5.9%)[14,19,31–33,47]. They demonstrate that even PTTT which demonstrate good predictive performance are likely to generate a large amount of 'false alarms' because adverse outcomes are so rare. For some centres, these issues may be mitigated to some extent by dedicated response teams or other available resources, but other hospitals may not be able to sustain the increased workload of responding to PTTT triggers.

How effective are early warning systems for reducing mortality and morbidity?

We found limited evidence for early warning system interventions reducing mortality or arrest rates in hospitalised children. While some effectiveness papers did report significant reductions in rates of mortality (on the ward or in PICU) or cardiac arrests after implementation of different early warning system interventions[16–18,62,63], they were all uncontrolled before-after studies which have inherent limitations in terms of establishing causality. They do not preclude the possibility that outcome rates would have improved over time regardless of the intervention[77] or changes were caused by other factors, and their inclusion is accordingly discouraged by some Cochrane review groups[78]. Three high quality multi-centre studies - two interrupted time series studies and a recent cluster randomised trial – found no changes in rates or trends of mortality or arrests post intervention[64,65,73].

There was also limited evidence for early warning systems reducing PICU transfers or calls for urgent review. Again, a small number of uncontrolled before-after studies reported significant reductions post-intervention[15,17,63], but several other studies reported significant increases in transfers or calls for review[66,72] or no post-intervention changes. We did find moderate evidence across four studies – including a controlled before-after study, a multi-centre interrupted time series study and a multi-centre cluster randomised trial - for early warning system interventions reducing rates of early critical interventions in children transferred to PICU[64,65,68,69]. Such results are promising, but corresponding reductions in hospital or PICU mortality rates have not yet been reported.

Implementing complex interventions in a healthcare setting is challenging and evidence from the adult literature points to challenges and barriers to successfully implement TTT in practice[79–81]. However, given so few effectiveness studies reported on implementation outcomes, it is difficult to know whether negative findings reflect poor effectiveness or

For peer review only - http://bmjopen.bmj.com/site/about/guidelines.xhtml

implementation of early warning systems. Again, effectiveness studies were predominantly carried out in specialist centres – and in all but three cases[67,68,70], involved the use of a dedicated response team – which greatly limits the generalisability of findings outside of these contexts.

Limitations of the review

There are several limitations of the current review. Firstly, despite purposely widening the scope of the effectiveness review question to include paediatric 'early warning systems' with or without a PTTT, we identified very few studies that did not employ a PTTT as part of the intervention . In part, this likely reflects the fact that PTTT have become almost synonymous with early warning systems, but it is also possible that our search strategy may have missed some broader early warning system initiatives that were not explicitly labelled as such. Secondly, our inclusion criteria for study selection were deliberately broad and so resulted in our including several validation and effectiveness studies that were subsequently excluded from analysis due to insufficient statistical detail or methodological issues. Thirdly, the scope of the current review was limited to consideration of quantitative validation and effectiveness studies. We are mindful of research suggesting that implementing PTTT in practice may confer secondary benefits including, but not limited to improvements in communication, teamwork and empowerment of junior staff to call for assistance[82–84]. Finally, we opted not to conduct a meta-analysis of effectiveness findings due to the heterogeneity of outcome metrics, interventions and study designs, populations and settings. Given the large sample sizes required to detect changes in rare adverse events, we believe further work is needed to harmonise outcome measures used to evaluate early warning system interventions internationally, in order to facilitate pooling of findings across studies.

Conclusion

The PTTT literature is currently characterised by an 'absence of evidence' rather than an 'evidence of absence'. PTTT seem like a logical tool for helping staff detect and respond to deteriorating patients, but the existing evidence base is too limited to form clear judgements of their utility. We would argue that there has been too much confidence placed in the statistical findings of validation studies of PTTT, given methodological limitations in the study designs. There is evidence of consistently high false-alarm rates and bench-testing studies point to many PTTT parameters not being reliably recorded in practice: as such there is reason for caution in considering the viability of PTTT for all hospitals. Almost all of the

For peer review only - http://bmjopen.bmj.com/site/about/guidelines.xhtml

early warning systems and PTTT reported in the literature have been developed and evaluated in specialist centres, typically in units with access to dedicated response teams – yet PTTT appear to be commonly adopted by non-specialist units with little modification. There is currently limited evidence that 'early warning systems' incorporating a PTTT reduce deterioration or death in practice. As such, we would urge caution among policymakers in calling for their use to become mandatory across all hospitals. We acknowledge the potential for PTTT to confer a range of secondary benefits in areas such as communication, teamwork and empowerment of junior staff. More work is required to understand the wider impact of PTTT implementation in different clinical settings before it is possible to evaluate their overall contribution to the wider safety mechanisms and systems aimed at identifying and responding to deteriorating in paediatric patients.

For peer review only

FOOTNOTES

Contributors

RT: screening and review of papers, contribution to design of work, preparation of manuscript; CH: screening and review of papers, contribution to concept and design of work, review of manuscript; FL: contribution to design of work, screening and review of papers, review of manuscript; KH: contribution to concept and design of work, screening and review of papers, review of manuscript; CP, DR, BM, AO, DE, RS, GS, DL, LT, DA, AL, ETJ: contribution to concept and design of work, screening and review of papers, review of manuscript; MM: information specialist, review of manuscript.

Acknowledgements

We would like to acknowledge the contribution of Dr James Bunn to the review.

Competing interests

None declared.

Funding

This study is funded by the National Institute for Health Research (NIHR) Health Services and Delivery Research (HS&DR) programme (12/178/17).

Data sharing statement

No additional data are available.

For peer review only

FIGURE LEGENDS

Figure 1 – PRISMA flow diagram of study inclusion

For peer review only

For peer review only - http://bmjopen.bmj.com/site/about/guidelines.xhtml

REFERENCES

1 Pearson G a (Ed). Why Children Die: A Pilot Study 2006; England (South West, North East and West Midlands), Wales and Northern Ireland. 2008. doi:ISBN: 978-0-9558055-0-9

2 Joint Commission. 2009 National Patient Safety Goals, Requirements and Rationales. *Jt Comm Perspect Patient Saf* 2009;**8**:8–12.

3 Tume L. The deterioration of children in ward areas in a specialist children's hospital. *Nurs Crit Care* 2007;**12**:12–9. doi:10.1111/j.1478-5153.2006.00195.x

4 NICE. Acutely ill patients in hospital: Recognition of and response to acute illness in adults in hospital. *NICE Clin Guidel 50* 2007;:1–107. doi:10.1136/bmj.39272.679688.47

5 Roland D, Oliver A, Edwards ED, *et al.* Use of paediatric early warning systems in Great Britain: has there been a change of practice in the last 7 years? *Arch Dis Child* 2014;**99**:26–9. doi:10.1136/archdischild-2012-302783

6 Chapman SM, Wray J, Oulton K, *et al.* Systematic review of paediatric track and trigger systems for hospitalised children. Resuscitation. 2016;**109**:87–109. doi:10.1016/j.resuscitation.2016.07.230

7 Lambert V, Matthews A, MacDonell R, *et al.* Paediatric early warning systems for detecting and responding to clinical deterioration in children: a systematic review. *BMJ Open* 2017;**7**:e014497. doi:10.1136/bmjopen-2016-014497

8 Moher D, Liberati A, Tetzlaff J, *et al.* Preferred reporting items for systematic reviews and meta-analyses: the PRISMA statement. *BMJ* 2009;**339**. doi:10.1136/bmj.b2535

9 Downs SH, Black N. The feasibility of creating a checklist for the assessment of the methodological quality both of randomised and non-randomised studies of health care interventions. *J Epidemiol Community Health* 1998;**52**:377–84. doi:10.1136/jech.52.6.377

10 Monaghan A. Detecting and managing deterioration in children. *Paediatr Nurs* 2005;**17**:32–5. doi:10.7748/paed2005.02.17.1.32.c964

11 Parshuram CS, Hutchison J, Middaugh K. Development and initial validation of the Bedside Paediatric Early Warning System score. *Crit Care* 2009;**13**:R135–R135. doi:10.1186/cc7998

12 Haines C, Perrott M, Weir P. Promoting care for acutely ill children - Development and evaluation of a Paediatric Early Warning Tool. *Intensive Crit Care Nurs* 2006;**22**:73–81. doi:10.1016/j.iccn.2005.09.003

13 Tibballs J, Kinney S, Duke T, *et al.* Reduction of paediatric in-patient cardiac arrest and death with a medical emergency team: preliminary results. *Arch Dis Child* 2005;**90**:1148–52. doi:10.1136/adc.2004.069401

14 Mason BW, Edwards ED, Oliver A, *et al.* Cohort study to test the predictability of the NHS Institute for Innovation and Improvement Paediatric Early Warning System. *Arch Dis Child* 2016;:archdischild – 2015–308465. doi:10.1136/archdischild-2015-308465

15 Brilli RJ, Gibson R, Luria JW, *et al.* Implementation of a medical emergency team in a large pediatric teaching hospital prevents respiratory and cardiopulmonary arrests outside the intensive care unit. Pediatr. Crit. Care Med. 2007;**8**:236–46.

For peer review only - http://bmjopen.bmj.com/site/about/guidelines.xhtml

doi:10.1097/01.PCC.0000262947.72442.EA

16 Hunt EA, Zimmer KP, Rinke ML, *et al.* Transition from a traditional code team to a medical emergency team and categorization of cardiopulmonary arrests in a children's center. *Arch Pediatr Adolesc Med* 2008;**162**:117–22. doi:10.1001/archpediatrics.2007.33

17 Sharek PJ, Parast LM, Leong K, *et al.* Effect of a rapid response team on hospital-wide mortality and code rates outside the ICU in a Children's Hospital. *JAMA* 2007;**298**:2267–74. doi:10.1097/01.SA.0000307935.42104.ee

18 Anwar-Ul-Haque, Saleem AF, Zaidi S, *et al.* Experience of pediatric rapid response team in a tertiary care hospital in Pakistan. *Indian J Pediatr* 2010;**77**:273–6. doi:10.1007/s12098-010-0032-2

19 Zhai H, Brady P, Li Q, *et al.* Developing and evaluating a machine learning based algorithm to predict the need of pediatric intensive care unit transfer for newly hospitalized children. *Resuscitation* 2014;**85**:1065–71. doi:10.1016/j.resuscitation.2014.04.009

20 Garlick J, Furlong-Dillard J, Alexander M, *et al.* Modified pediatric early warning scores, a predictor of appropriate transfer to higher level of care. *Crit Care Med* 2013;**41**:A166–7. doi:http://dx.doi.org/10.1097/01.ccm.0000439917.60065.34

21 Medar S, Weingarten-Arams J, Katyal C. Pediatric early warning score (PEWS) is a reliable predictor of rapid response team (RRT) call. *Crit Care Med* 2015;**43**:228. doi:http://dx.doi.org/10.1097/01.ccm.0000474735.10884.b3

22 Bell D, Mac A, Ochoa Y, *et al.* The texas children's hospital pediatric advanced warning score as a predictor of clinical deterioration in hospitalized infants and children: A modification of the pews tool. *J Pediatr Nurs* 2013;**28**. doi:10.1016/j.pedn.2013.04.005

23 McLellan MC, Connor JA. The Cardiac Children's Hospital Early Warning Score (C-CHEWS). *J Pediatr Nurs* 2013;**28**:171–8. doi:10.1016/j.pedn.2012.07.009

24 Rahman ZH, Leahy NE, Sessler K, *et al.* Application of the burn center Pediatric Early Warning Score (PEWS) tool to clinical practice: A pilot validation study. *J Burn Care Res* 2014;**35**:S187. doi:http://dx.doi.org/10.1097/01.bcr.0000445189.61189.9d

25 Hopkins M, Rowan C, Rigby M, *et al.* PEWS score predicts the need for PICU transfer and critical care interventions. *Crit Care Med* 2013;**Conference**:43rd Critical Care Congress of the Society of Crit. doi:http://dx.doi.org/10.1097/01.ccm.0000439922.90559.23

26 Gawronski O, Bertaina A, Broccati F, *et al.* Bedside pews in an Italian pediatric bone marrow transplant unit: Preliminary results of a longitudinal retrospective chart review. *Intensive Care Med* 2013;**39**:S130. doi:http://dx.doi.org/10.1007/s00134-013-2950-8

27 Sefton G, Lane S, Carrol ED. Performance of individual predictors of deterioration used in a paediatric early warning score. *Pediatr Crit Care Med* 2014;**15**:29–30. doi:http://dx.doi.org/10.1097/01.pcc.0000448835.89804.43

28 Robson M-AJ, Cooper CL, Medicus L a, *et al.* Comparison of three acute care pediatric early warning scoring tools. *J Pediatr Nurs* 2013;**28**:e33–41. doi:10.1016/j.pedn.2012.12.002

29 Chapman SM, Wray J, Oulton K, *et al.* 'The Score Matters': wide variations in

For peer review only

predictive performance of 18 paediatric track and trigger systems. *Arch Dis Child* 2017;:archdischild – 2016–311088. doi:10.1136/archdischild-2016-311088

30 Fuijkschot J, Vernhout B, Lemson J, *et al.* Validation of a paediatric early warning score: First results and implications of usage. *Eur J Pediatr* 2015;**174**:15–21. doi:10.1007/s00431-014-2357-8

31 Tucker KM, Brewer TL, Baker RB, *et al.* Prospective evaluation of a pediatric inpatient early warning scoring system. *J Spec Pediatr Nurs* 2009;**14**:79–85. doi:10.1111/j.1744-6155.2008.00178.x

32 Edwards ED, Powell C V, Mason BW, *et al.* Prospective cohort study to test the predictability of the Cardiff and Vale paediatric early warning system. *Arch Dis Child* 2009;**94**:602–6. doi:10.1136/adc.2008.142026

33 Edwards ED, Mason BW, Oliver A, *et al.* Cohort study to test the predictability of the Melbourne criteria for activation of the medical emergency team. *Arch Dis Child* 2011;**96**:174–9. doi:10.1136/adc.2010.187617

34 O'Loughlin K, Ruparelia K, Vince T, *et al.* The effectiveness of the paediatric early warning tool (PEWT) in identifying children requiring admission to a critical care unit. *Arch Dis Child* 2012;**97**:A154. doi:http://dx.doi.org/10.1136/archdischild-2012-301885.362

35 Wright D, Sefton G, Horan M. Charted observations and the use of a paediatric early warning tool did not predict the majority of cardio-respiratory arrest calls in a paediatric hospital. *Arch Dis Child* 2011;**96**:A59. doi:http://dx.doi.org/10.1136/adc.2011.212563.134

36 Ahmed M, Sobithadevi D, Lall R, *et al.* Burton paediatric early warning system score. *Arch Dis Child* 2012;**97**:A421. doi:http://dx.doi.org/10.1136/archdischild-2012-302724.1483

37 Mclellan MC, Gauvreau K, Connor JA. Validation of the cardiac children's hospital early warning score: An early warning scoring tool to prevent cardiopulmonary arrests in children with heart disease. *Congenit Heart Dis* 2014;**9**:194–202. doi:10.1111/chd.12132

38 Clayson R, Selby A, Tume L. Is a paediatric early warning tool (PEWT) useful in cardiac patients? *Pediatr Crit Care Med* 2014;**15**:35–6. doi:http://dx.doi.org/10.1097/01.pcc.0000448865.31975.d1

39 Fenix JB, Gillespie CW, Levin A, *et al.* Comparison of Pediatric Early Warning Score to Physician Opinion for Deteriorating Patients. *Hosp Pediatr* 2015;**5**:474–9. doi:10.1542/hpeds.2014-0199

40 Mandell IM, Bynum F, Marshall L, *et al.* Pediatric Early Warning Score and unplanned readmission to the pediatric intensive care unit. *J Crit Care* 2015;**30**:1090–5. doi:10.1016/j.jcrc.2015.06.019

41 Agulnik A, Forbes PW, Stenguist N, *et al.* Validation of a Pediatric Early Warning Score in Hospitalized Pediatric Oncology and Hematopoietic Stem Cell Transplant Patients. *Pediatr Crit Care Med* 2016;**17**:e146–53. doi:http://dx.doi.org/10.1097/PCC.0000000000000662

42 Agulnik A, Mendez Aceituno A, Mora Robles LN, *et al.* Validation of a pediatric early warning system for hospitalized pediatric oncology patients in a resource-limited setting. *Cancer* 2017;**123**:4903–13. doi:10.1002/cncr.30951

For peer review only - http://bmjopen.bmj.com/site/about/guidelines.xhtml

43 Blackstone B, Sharmila A, Pienaar A, *et al.* G453(P) 'Between the flags' paediatric early warning system is a sensitive tool to detect deterioration in children from district general hospitals admitted to paediatric intensive care. *Arch Dis Child* 2017;**102**:A178 LP – A179.http://adc.bmj.com/content/102/Suppl_1/A178.2.abstract

44 Parshuram CS, Duncan HP, Joffe AR, *et al.* Multicentre validation of the bedside paediatric early warning system score: a severity of illness score to detect evolving critical illness in hospitalised children. *Crit Care* 2011;**15**:R184. doi:10.1186/cc10337

45 Akre M, Finkelstein M, Erickson M, *et al.* Sensitivity of the pediatric early warning score to identify patient deterioration. *Pediatrics* 2010;**125**:e763–9. doi:10.1542/peds.2009-0338

46 Gawronski O, Ciofi Degli Atti ML, Di Ciommo V, *et al.* Accuracy of Bedside Paediatric Early Warning System (BedsidePEWS) in a Pediatric Stem Cell Transplant Unit. *J Pediatr Oncol Nurs* 2016;**33**:249–56. doi:10.1177/1043454215600154

47 Duncan H, Hutchison J, Parshuram CS. The pediatric early warning system score: A severity of illness score to predict urgent medical need in hospitalized children. *J Crit Care* 2006;**21**:271–8. doi:10.1016/j.jcrc.2006.06.007

48 Skaletzky SM, Raszynski A, Totapally BR. Validation of a modified pediatric early warning system score: a retrospective case-control study. *Clin Pediatr (Phila)* 2012;**51**:431–5. doi:10.1177/0009922811430342

49 Ross C, Harrysson I, Goel V, *et al.* Sensitivity and specificity of a pediatric early warning system using data-driven vital signs. *Crit Care Med* 2015;**43**:39. doi:http://dx.doi.org/10.1097/01.ccm.0000473979.19857.a8

50 McLellan M, Gauvreau K, Connor JA. Validation of the children's hospital early warning scoring system for identifying hospitalized children at risk for arrest or ICU transfer. *Pediatr Crit Care Med* 2014;**15**:30. doi:http://dx.doi.org/10.1097/01.pcc.0000448836.27923.e3

51 Mistry KP, Turi J, Hueckel R, *et al.* Pediatric Rapid Response Teams in the Academic Medical Center. *Clin Pediatr Emerg Med* 2006;**7**:241–7. doi:10.1016/j.cpem.2006.08.010

52 Demmel KM, Williams L, Flesch L. Implementation of the pediatric early warning scoring system on a pediatric hematology/oncology unit. *J Pediatr Oncol Nurs* 2010;**27**:229–40. doi:10.1177/1043454209358410

53 Sandhu S, Ralph T, Bradbury K, *et al.* 726 The Paediatric Early Warning Tool in Combination with a Nurse-Led Outreach Team: Impact on Outcomes for Critically Ill Children. *Pediatr Res* 2010;**68**:368.http://dx.doi.org/10.1203/00006450-201011001-00726

54 Randhawa S, Roberts-Turner R, Woronick K, *et al.* Implementing and Sustaining Evidence-Based Nursing Practice to Reduce Pediatric Cardiopulmonary Arrest. *West J Nurs Res* 2010;**33**:443–56. doi:10.1177/0193945910379585

55 Camacho EA, Holman D, Badgett V, *et al.* Modification of a Pediatric Early Warning Tool for Use With Patients in a Specialized Cardiac/Renal Unit. *J Pediatr Nurs* 2011;**26**:e2–3. doi:http://dx.doi.org/10.1016/j.pedn.2010.12.021

56 Heyden M V, Mudge B, Casella S. Initiation of a dedicated pediatric rapid response team reduces cardiopulmonary arrests outside of the intensive care unit in a small children's hospital. *Crit Care Med* 2012;**40**:147.

For peer review only - http://bmjopen.bmj.com/site/about/guidelines.xhtml

doi:http://dx.doi.org/10.1097/01.ccm.0000425605.04623.4b

57 Norville R, Wills-Bagnato P, Staton S, *et al.* Improvement in early recognition of deteriorating pediatric bone marrow transplant patients. *Biol Blood Marrow Transplant* 2013;**19**:S173. doi:http://dx.doi.org/10.1016/j.bbmt.2012.11.152

58 Ambati SR, Sweberg T, Silver P, *et al.* Effect of simulation based curriculum on the utilization of rapid response team activations (RRT). *Crit Care Med* 2014;**42**:A1432. doi:http://dx.doi.org/10.1097/01.ccm.0000457794.10321.00

59 Ocholi T, Downing E, Rzeskiewicz A, *et al.* Impact of the Bedside PEWS on early detection of deteriorating patients. *Arch Dis Child* 2014;**99**:A89–90. doi:http://dx.doi.org/10.1136/archdischild-2014-306237.207

60 Zenker P, Schlesinger A, Hauck M, *et al.* Implementation and impact of a rapid response team in a children's hospital. *Jt Comm J Qual Patient Saf* 2007;**33**:418–25. doi:10.1016/S1553-7250(07)33048-1

61 Hanson CC, Randolph GD, Erickson JA, *et al.* A reduction in cardiac arrests and duration of clinical instability after implementation of a paediatric rapid response system. *Postgrad Med J* 2010;**86**:314–8. doi:10.1136/qshc.2007.026054

62 Tibballs J, Kinney S. Reduction of hospital mortality and of preventable cardiac arrest and death on introduction of a pediatric medical emergency team. *Pediatr Crit Care Med* 2009;**10**:306–12. doi:10.1097/PCC.0b013e318198b02c

63 Kotsakis A, Lobos A-T, Parshuram C, *et al.* Implementation of a multicenter rapid response system in pediatric academic hospitals is effective. *Pediatrics* 2011;**128**:72–8. doi:10.1542/peds.2010-0756

64 Parshuram CS, Dryden-Palmer K, Farrell C, *et al.* Effect of a pediatric early warning system on all-cause mortality in hospitalized pediatric patients: The epoch randomized clinical trial. *JAMA* 2018;**319**:1002–12.

65 Bonafide CP, Localio AR, Roberts KE, *et al.* Impact of Rapid Response System Implementation on Critical Deterioration Events in Children. *JAMA Pediatr* 2014;**168**:25. doi:10.1001/jamapediatrics.2013.3266

66 Duns N, Ceely B, Festa M, *et al.* Introduction of a track and trigger system is associated with increased paediatric intensive care outreach utilisation and a trend towards improved patient outcomes in a tertiary children's hospital. *Aust Crit Care* 2014;**27**:61. doi:http://dx.doi.org/10.1016/j.aucc.2013.10.058

67 Agulnik A, Mora Robles LN, Forbes PW, *et al.* Improved outcomes after successful implementation of a pediatric early warning system (PEWS) in a resource-limited pediatric oncology hospital. *Cancer* 2017;**123**:2965–74. doi:10.1002/cncr.30664

68 Sefton G, McGrath C, Tume L, *et al.* What impact did a Paediatric Early Warning system have on emergency admissions to the paediatric intensive care unit? An observational cohort study. *Intensive Crit care Nurs* 2015;**31**:91–9. doi:10.1016/j.iccn.2014.01.001

69 Kolovos NS, Gill J, Michelson PH, *et al.* Reduction in Mortality Following Pediatric Rapid Response Team Implementation. *Pediatr Crit Care Med* 2018;:1. doi:10.1097/PCC.0000000000001519

70 Parshuram CS, Bayliss A, Reimer J, *et al.* Implementing the Bedside Paediatric Early Warning System in a community hospital: A prospective observational study. *Paediatr Child Health* 2011;**16**:e18–

For peer review only - http://bmjopen.bmj.com/site/about/guidelines.xhtml

22.http://www.pubmedcentral.nih.gov/articlerender.fcgi?artid=3077313&tool=pmcentrez&rendertype=abstract

71 Panesar R, Polikoff LA, Harris D, *et al.* Characteristics and outcomes of pediatric rapid response teams before and after mandatory triggering by an elevated Pediatric Early Warning System (PEWS) score. *Hosp Pediatr* 2014;**4**:135–40. doi:10.1542/hpeds.2013-0062

72 Douglas K, Collado JC, Keller S. Implementation of a Pediatric Early Warning Scoring System at an Academic Medical Center. *Crit Care Nurs Q* 2016;**39**:363–70. doi:10.1097/CNQ.0000000000000130

73 Kutty S, Jones PG, Karels Q, *et al.* Association of Pediatric Medical Emergency Teams With Hospital Mortality. *Circulation* 2018;**137**:38–46. doi:10.1161/CIRCULATIONAHA.117.029535

74 Guerra MW, Shults J, Amsterdam J, *et al.* The analysis of binary longitudinal data with time-dependent covariates. *Stat Med* 2012;**31**:931–48. doi:10.1002/sim.4465

75 Selig JP, Preacher KJ, Little TD. Modeling Time-Dependent Association in Longitudinal Data: A Lag as Moderator Approach. *Multivariate Behav Res* 2012;**47**:697–716. doi:10.1080/00273171.2012.715557

76 Clifton DA, Clifton L, Sandu D-M, *et al.* ‘Errors’ and omissions in paper-based early warning scores: the association with changes in vital signs-a database analysis. *BMJ Open* 2015;**5**:e007376. doi:10.1136/bmjopen-2014-007376

77 Joffe AR, Anton NR, Burkholder SC. Reduction in hospital mortality over time in a hospital without a pediatric medical emergency team: limitations of before-and-after study designs. *Arch Pediatr Adolesc Med* 2011;**165**:419–23. doi:10.1001/archpediatrics.2011.47

78 Goodacre S. Uncontrolled before-after studies: Discouraged by Cochrane and the EMJ. *Emerg Med J* 2015;**32**:507–8. doi:10.1136/emermed-2015-204761

79 Niegsch M, Fabritius ML, Anhoj J. Imperfect Implementation of an Early Warning Scoring System in a Danish Teaching Hospital: A Cross-Sectional Study. *PLoS One* 2013;**8**. doi:10.1371/journal.pone.0070068

80 Hands C, Reid E, Meredith P, *et al.* Patterns in the recording of vital signs and early warning scores: Compliance with a clinical escalation protocol. *BMJ Qual Saf* 2013;**22**:719–26. doi:http://dx.doi.org/10.1136/bmjqs-2013-001954

81 Lydon S, Byrne D, Offiah G, *et al.* A mixed-methods investigation of health professionals’ perceptions of a physiological track and trigger system. *BMJ Qual* 2015;:688–95. doi:10.1136/bmjqs-2015-004261

82 Bonafide CP, Roberts KE, Weirich CM, *et al.* Beyond statistical prediction: Qualitative evaluation of the mechanisms by which pediatric early warning scores impact patient safety. *J Hosp Med* 2013;**8**:248–53. doi:10.1002/jhm.2026

83 Ennis L. Paediatric early warning scores on a children’s ward: a quality improvement initiative. *Nurs Child Young People* 2014;**26**:25–31. doi:10.7748/ncyp.26.7.25.e478

84 Kaul M, Snethen J, Kelber ST, *et al.* Implementation of the Bedside Paediatric Early Warning System (BedsidePEWS) for nurse identification of deteriorating patients. *J Spec Pediatr Nurs* 2014;**19**:339–49. doi:10.1111/jspn.12092

For peer review only

For peer review only - http://bmjopen.bmj.com/site/about/guidelines.xhtml

Table 2– summary of PTTTs

PTTT name (references)	Development / modification details	Score / trigger	Choice of thresholds / parameters	Age-dependent thresholds?	No. of items in the tool*	PTTT parameters															
						Respiratory rate	Heart rate	Respiratory effort / distress	LOC / behaviour	Oxygen saturation	Capillary refill time	Oxygen therapy	Systolic blood pressure	Pain	Staff concern	Skin colour	Airway problems	Temperature	Pulses	Family concern	Other items
Paediatric Early Warning System score and derivatives																					
Paediatric Early Warning System (PEWS) score[28,47]	Developed for use in Canadian tertiary centre[47]. Nurse-generated candidate items reduced by focus groups/Delphi and evaluation with clinical dataset (code blue calls, n=87; controls, n=128). Development and validation datasets not independent.	Score	Expert opinion	Yes	16	✓	✓		✓	✓	✓	✓	✓				✓	✓	✓		Bolus fluid, medications, home oxygen, any previous admission to an ICU, central venous line in situ, transplant recipient, severe cerebral palsy, gastrostomy tube, greater than 3 medical specialties involved in care
Bedside Paediatric Early Warning Score (PEWS)[11,19,25,26,28,44,46,59,64,65,70]	Developed for use in US tertiary centre[11]. Routinely collected items assessed for discriminatory ability using clinical dataset (PICU admission, n=60; controls, n=120). Development and validation set not independent.	Score	Expert opinion	Yes	7	✓	✓	✓		✓	✓	✓	✓								
Modified Bedside PEWS (a)[30]	Modification to Bedside PEWS for use in Dutch tertiary centre. Added temperature; modified wording of respiratory effort and oxygen therapy items.	Score	Expert opinion	Yes	8	✓	✓	✓		✓	✓	✓	✓					✓			
Modified Bedside PEWS (b)[49]	Modification to Bedside PEWS for use in US tertiary centre. Changed normal thresholds for HR and RR based on analysis of local clinical data.	Score	HR / RR data driven	Yes	7	✓	✓	✓		✓	✓	✓	✓								
Brighton PEWS and derivatives																					
Brighton PEWS[10,54]	Initial development for use in UK tertiary centre. Adapted from existing adult scores, but amended based on	Score	Expert opinion	No	5	✓	✓	✓	✓		✓	✓				✓					¼ hourly nebulisers, persistent vomiting post-surgery

For peer review only - http://bmjopen.bmj.com/site/about/guidelines.xhtml

	local clinical consensus. Small audit of patients (n=30) described but no formal validation.																				
Modified Brighton PEWS (a)[19,31,39]	Modification of Brighton PEWS for use in general medical ward of a US tertiary centre. Altered thresholds for oxygen therapy; changed wording for respiratory effort; modified escalation algorithm.	Score	Expert opinion	No	5	✓	✓	✓	✓		✓	✓				✓					¼ hourly nebulisers, persistent vomiting post-surgery
Modified Brighton PEWS (b)[45,72]	Modification of Brighton PEWS for use in US tertiary centre. Added age-dependent thresholds for HR and RR.	Score	Expert opinion	Yes	5	✓	✓	✓	✓		✓	✓				✓					¼ hourly nebulisers, persistent vomiting post-surgery
Modified Brighton PEWS (c)[52]	Modification of Brighton PEWS for use in a US haematology / oncology unit. Altered thresholds; changed respiratory effort wording; modified escalation algorithm; added and removed items. No formal validation study reported.	Score	Expert opinion	No	3	✓	✓	✓	✓		✓	✓				✓				✓	
Modified Brighton PEWS (d)[48]	Modification of Brighton PEWS for use in a US tertiary centre. Modified wording of Behaviour component, added age-dependent thresholds for HR and RR; removed nebulisers and persistent vomiting.	Score	Expert opinion	Yes	3	✓	✓	✓	✓		✓	✓				✓					
Modified Brighton PEWS (e)[71]	Modification of Brighton PEWS for use in a US tertiary centre. Modified wording of Behaviour and respiratory effort items; altered thresholds for O2 therapy; removed nebs and persistent vomiting items. No formal validation study reported.	Score	Expert opinion	No	3	✓	✓	✓	✓		✓	✓				✓					
Texas Children's Hospital (TCH) PAWS[22]	Modification of Brighton PEWS for use in a US tertiary centre. Modified wording of Behaviour category; added scoring items to Resp. and Cardiovascular categories; changed O2 therapy thresholds; modified escalation algorithm.	Score	Expert opinion	No	5	✓	✓	✓	✓	✓	✓	✓				✓					Hourly respiratory treatments; persistent vomiting post-surgery
Children's Hospital Early Warning Score (CHEWS)[50]	Modification of Brighton PEWS for use in a US tertiary centre. Altered thresholds for O2 therapy; changed wording for Behaviour and Resp. categories; added staff and family	Score	Expert opinion	No	5	✓	✓	✓	✓		✓	✓			✓	✓				✓	

For peer review only

	concern; removed nebs and vomiting; modified escalation algorithm.																			
Children's Hospital Cardiac Early Warning Score (C-CHEWS) [23,41,42,67]	Modification of Brighton PEWS for cardiac ward of a US tertiary centre. Altered O2 therapy thresholds; added items to Behaviour, Resp. and Cardiovascular categories; added family & staff concern; added age-related thresholds; removed nebs and vomiting items; modified escalation algorithm.	Score	Expert opinion	Yes	5	✓	✓	✓	✓	✓	✓	✓		✓	✓	✓			✓	✓	
Burn-specific PEWS[24]	Modification of Brighton PEWS, for use in a specialist Burn Centre of a US tertiary centre. Added temperature; added intake and output scoring items; added Skin component.	Score	Expert opinion	No	6	✓	✓	✓	✓	✓	✓	✓				✓			✓		Intake; outputs; skin
Children's Hospital Los Angeles (CHLA) PEWS[40]	Modification of Brighton PEWS for use in a US tertiary centre. Added medical history scoring item; added single ventricle physiology scoring item; changed O2 therapy thresholds; added items to Resp. category.	Score	Expert opinion		4	✓	✓	✓	✓		✓	✓				✓					RRT, code blue, or transfer from/to PICU in past 2 weeks; single ventricle physiology; any assisted ventilation
Melbourne Activation Criteria and derivatives																				
Melbourne Activation Criteria (MAC)[3,13,33,62]	Initial development for use in an Australian tertiary centre to activate MET. Adapted from adult MET calling criteria, using age-appropriate thresholds. No formal validation study reported.	Trigger	Expert opinion	Yes	9	✓	✓	✓	✓	✓			✓		✓		✓				Cardiac or respiratory arrest
Modified MAC[63]	Modification of MAC for use in a Canadian tertiary centre, to activate a RRS. Removed cardiac / respiratory arrest outcome. No formal validation study reported.	Trigger	Expert opinion	Yes	8	✓	✓	✓	✓	✓			✓		✓		✓				
Cardiff & Vale Paediatric Early Warning Score (C&VPEWS)[32,33]	Modification of MAC for evaluation in a UK tertiary centre. Removed cardiac / respiratory arrest outcome; altered thresholds of some items; evaluated as aggregate score rather than single-item trigger.	Score	Expert opinion	Yes	8	✓	✓	✓	✓	✓			✓		✓		✓				
Bristol Paediatric Early Warning Tool and derivatives																				

For peer review only - http://bmjopen.bmj.com/site/about/guidelines.xhtml

Bristol Paediatric Early Warning Tool (PEWT) [3,12,28,34,35]	Initial development for use in a UK tertiary centre. Initial candidate items drawn from un-validated Plymouth tool – retrospectively evaluated for ability to predict adverse events among cases (n=360, HDU or PICU transfers). Development and validation dataset not independent.	Trigger	APLS values	Yes	14	✓	✓		✓	✓	✓	✓	✓	✓	✓		✓				Required nebulised adrenaline; hyperkalaemia; suspected meningococcus; diabetic ketoacidosis; persistent convulsion
Modified Bristol PEWT (a)[68]	Modification of Bristol PEWT for a UK tertiary centre. Adjusted wording of Airway parameters; added respiratory items; added AVPU evaluation; removed suspected meingococcus and diabetic ketoacidosis; added ph<7.2 and unresolved pain. No formal validation study reported.	Trigger	APLS values	Yes	14	✓	✓	✓	✓	✓	✓	✓	✓	✓	✓		✓				Required nebulised adrenaline or no improvement after nebulisers; pH<7.2; unresolved pain or current analgesic therapy; fitting
Modified Bristol PEWT (b)[38]	Modification of Bristol PEWT for cardiac ward of a UK tertiary centre. Amended HR and RR thresholds. Adjusted wording of Airway parameters; added respiratory items; added AVPU evaluation; removed suspected meingococcus and diabetic ketoacidosis; added ph<7.2 and unresolved pain	Trigger	HR / RR data driven	Yes	14	✓	✓	✓	✓	✓	✓	✓	✓	✓	✓		✓				Required nebulised adrenaline or no improvement after nebulisers; pH<7.2; unresolved pain or current analgesic therapy; fitting
Other PTTT																					
NHS Institute for Innovation and Improvement (NHS III) PEWS[14]	Designed as part of a NHS Institute fellowship project. Adapted from adult scores and Brighton PEWS. No formal development or internal validation study published.	Score	APLS values	Yes	6	✓	✓	✓	✓			✓			✓						
Paediatric Medical Emergency Team (PMET) triggering criteria (a)[15]	Initial development for use in a US tertiary centre to activate a MET. Retrospective chart review of case patients (n-44, code calls) used to generate candidate items. Clinical judgement used to select final items. No formal validation of final tool reported.	Trigger	Expert opinion	No	4			✓	✓	✓					✓	✓				✓	Worsening retractions; cyanosis
Paediatric Medical Emergency Team (PMET)	Initial development for use in a US tertiary centre to activate a MET. Minimal description of tool development – authors deliberately	Trigger	Expert opinion	Uncl ear	12	✓	✓	✓	✓	✓					✓					✓	Cardiac or respiratory arrest; seizures with apnoea; progressive lethargy; circulatory compromise/acute shock syndrome

For peer review only - http://bmjopen.bmj.com/site/about/guidelines.xhtml

triggering criteria (b)[16]	chose broad criteria and categories of illness rather than specific vital signs. No formal validation study reported.																				
Paediatric Rapid Response Team (PRRT) triggering criteria (a)[17]	Initial development for use in a US tertiary centre, to activate a RRT. Triggering items elected through expert consensus locally – reference to similarity to MAC and PMET triggering criteria (a). No formal validation study reported.	Trigger	Expert opinion	No	6	✓	✓		✓	✓			✓		✓						
Paediatric Rapid Response Team (PRRT) triggering criteria (b)[18]	Initial development for use in calling RRT team in a tertiary centre in Pakistan. Minimal explanation for selection of calling criteria. No formal validation study reported in the literature.	Trigger	Unclear	Yes	8	✓	✓	✓	✓	✓			✓		✓						Convulsion
Logistic regression algorithm[19]	Initial development based on data mining of electronic health records in US tertiary-centre. Extracted 24 hours of clinical data from inpatients (n=6,722 controls, 526 PICU transfers) and used logistic regression model to select 29 item tool. Validation performed on subset of development dataset.	Score	Expert opinion	Yes	29	✓	✓	✓	✓	✓	✓		✓	✓				✓			Acuity level (local measure); tissue perfusion and oxygenation

* Multiple parameters are often required to be collected for each scoring item/category, e.g., scoring the 'Cardiovascular' category in the Brighton PEWS requires collection / evaluation of heart rate, skin colour and capillary refill time

† Denotes a study included in the effectiveness review

APLS: Advanced Paediatric Life Support; AVPU: Alert, Voice, Pain, Unresponsive; GCS: Glasgow Coma Scale; HR: Heart rate; LOC: Level of consciousness; PHDU: Paediatric High-Dependency Unit; PICU: Paediatric Intensive Care Unit; PTTS: Paediatric Track and Trigger Tool; RR: Respiratory rate.

For peer review only - http://bmjopen.bmj.com/site/about/guidelines.xhtml

Table 3 – summary of PTTT validation study outcomes

PTTT	First author, year	Country	Study population	Study design	Number of centres	PTTT used in practice?	Internal / external validation study?	Outcome measures	Sample size	Score or trigger?	Score tested / maximum score	Which score used (frequency of scoring)?*	AUROC	Sensitivity	Specificity	PPV	NPV	Notes on accuracy / reliability of scoring and missing data	Quality score (max = 24)
Paediatric Early Warning System (PEWS) score	Duncan 2006[47]	Canada	All in-patients	Case-control study (retrospective)	1	No	Int	Code blue call for actual or impending cardiopulmonary arrest	215 (87 cases)	S	5 / 26	Max 24hrs before event (hourly)	0.90	78.0	95.0	4.2†		No details on data abstraction. 13% of eligible cases and 84% of eligible controls excluded due to incomplete clinical data.	14
	Robson 2013[28]	US	All in-patients	Case-control study (retrospective)	1	No	Ext	Code blue call	192 (96 cases)	S	5 / 32	Max 24hrs before event (6 hourly)	0.85	86.6	72.2			Four researchers scored PTTT from 20 charts, inter-rater reliability of 0.95. No details on extent of missing data.	8
	Chapman 2017[29]	UK	All in-patients	Case-control study (retrospective)	1	No	Ext	Death, arrest or unplanned PICU transfer	608 (297 cases)	S	7 / 32	Max 48hrs before event (per usual practice)	0.82	70.0	75.0	*72.6*	*72.0*	Data abstraction by single researcher. 36% of observation sets contained HR, RR, O2 Sats, systolic BP, temperature and assessment of consciousness.	17
Bedside PEWS	Parshuram 2009[11]	Canada	All in-patients	Case-control study (retrospective)	1	No	Int	Urgent PICU transfer (without code blue call)	180 (60 cases)	S	8 / 26	Max 24hrs before event (hourly)	0.91	82.0	93.0			Availability of scoring items in medical records varied from 27% (cap refill time) to 93% (oxygen therapy).	21

For peer review only - http://bmjopen.bmj.com/site/about/guidelines.xhtml

	Parshuram 2011[44]	Canada & UK	All in-patients	Case-control study (prospective)	4	No	Ext	Urgent PICU transfer or immediate call to resuscitation team	2,074 (686 cases)	S	7 / 26	Max 24hrs before event (hourly)	0.87	64.0	91.0			PTTT scores calculated electronically after abstraction by research nurse. 5.1% of records had all 7 items recorded, 31% had at least 5 items.	22
	Robson 2013[28]	US	All in-patients	Case-control study (retrospective)	1	No	Ext	Code blue call	192 (96 cases)	S	7 / 26	Max 24hrs before event (6 hourly)	0.73	56.3	78.1			See above.	8
	Zhai 2014[19]	US	All in-patients	Case-control study (retrospective)	1	No	Ext	Urgent PCU transfer within 24 hrs of admission	6,352 (53 cases)	S	7 / 26	Max 24 hrs before event (hourly)	0.82	73.6	71.7	2.1†		Data extracted from electronic health records. Excluded two items of Bedside PEWS (oxygen therapy and respiratory effort) due to difficulty abstracting.	17
	Gawronski 2016[46]	Italy	Stem Cell Transplant Unit	Case-control study (retrospective)	1	No	Ext	Unexpected death, urgent consult with RRT or urgent PICU transfer	99 (19 cases)	S	6 / 26	Score 4hrs before event	0.90	79.0	97.5			Data abstracted by research nurses. No details on extent of missing data. Conflicting / missing observations resolved by interviews with clinical staff.	15
	Chapman 2017[29]	UK	All in-patients	Case-control study (retrospective)	1	No	Ext	Death, arrest or PICU transfer	608 (297 cases)	S	6 / 26	Max 48hrs before event (per usual practice)	0.88	72.0	89.0	*86.0*	*77.0*	See above.	17

For peer review only - http://bmjopen.bmj.com/site/about/guidelines.xhtml

Modified Bedside PEWS (a)	Fuijkschot 2015[30] (study 1)	Netherlands	Oncology ward	Case-cohort study (retrospective)	1	Yes	Int	Emergency medical intervention or reviewed by PICU staff or staff concern	118 (15 cases)	S	8 / 28	Unclear (minimum 8 hourly)				*73.0*		41% of admissions excluded from study due to incomplete PTTT scores.	10
	Fuijkschot 2015[30] (study 2)	Netherlands	All in-patients	Case-cohort study (retrospective)	1	Yes	Int	PICU transfer	Unclear (24 cases)	S	8 / 28	Score 2-6hrs before event (minimum 8 hourly)		66.6				High rate of exclusions reported due to missing data.	10
	Fuijkschot 2015[30] (study 3)	Netherlands	All in-patients	Case-cohort study (prospective)	1	Yes	Int	Emergency medical intervention	Unclear (14 cases)	S	8 / 28	Unclear (minimum 8 hourly)		100				No details on missing data.	10
	Chapman 2017[29]	UK	All in-patients	Case-control study (retrospective)	1	No	Ext	Death, arrest or PICU transfer	608 (297 cases)	S	7 / 28	Max 48hrs before event (per usual practice)	0.87	69.0	91.0	*87.9*	*79.0*	See above.	17
Modified Bedside PEWS (b)	Ross 2015[49]	US	All in-patients	Case-control study (retrospective)	1	No	Int	Urgent PICU transfer	4628 (848 cases)	S	8 / 26	Max during admission		70.0	84.0			No details on data abstraction. Respiratory effort category excluded due to difficulty abstracting. No details on missing data.	9
Modified Brighton PEWS (a)	Tucker 2008[31]	US	General medical unit	Cohort study (prospective)	1	Yes	Int	PICU transfer	2,979 (51 cases)	S	3 / 11	Max during admission (4 hourly)	0.89	90.2	74.4	5.8	99.8	Intraclass coefficient of 0.92 reported for two bedside nurses scoring 55 patients. No details on missing data.	14

For peer review only - http://bmjopen.bmj.com/site/about/guidelines.xhtml

	Zhai 2014[19]	US	All in-patients	Case-control study (retrospective)	1	No	Ext	Urgent PCU transfer within 24 hrs of admission	6,352 (53 cases)	S	2 / 11	Max 24hrs before event (hourly)	0.74	68.4	81.6	2.3		Data extracted from electronic health records. Only included records with complete PEWS score: 64% of eligible cases and 51% of eligible controls excluded.	17
	Fenix 2015[39]	US	PICU transfers among all in-patients (excluding haematology oncology, surgical and cardiac wards)	Case-control study (retrospective)	1	Yes	Ext	Non-elective PICU transfer followed by deterioration event	97 PICU transfers (51 cases of PICU transfer followed by 'deterioration event')	S	3 / 11	Max during admission		80.0	43.0	*61.0*	*67.0*	No details on missing data.	15
Modified Brighton PEWS (b)	Akre 2010[45]	US	All in-patients	Chart review study (retrospective)	1	No	Int	Rapid response team call or code blue call	186 cases (170 RRT calls, 16 code calls)	S	4 / 13	Max 24 hrs before event (minimum 4 hourly)		85.5				Scores abstracted from charts by single nurse, having calibrated with advanced nurse practitioner. Categories scored missing if any items missing. 25% of charts missing behavioural state, 26% cardiovascular colour.	14
	Chapman 2017[29]	UK	All in-patients	Case-control study (retrospective)	1	No	Ext	Death, arrest or PICU transfer	608 (297 cases)	S	4 / 13	Max 48hrs before event (per usual practice)	0.79	61.0	84.0	*78.4*	*69.0*	See above.	17

For peer review only

Modified Brighton PEWS (d)	Skaletzky 2012[48]	US	Medical surgical wards	Case-control study (retrospective)	1	No	Int	PICU transfer	350 (100 cases)	S	2.5 / 9	Max 48hrs before event (4 hourly)	0.81	62.0	89.0			Data abstracted from medial charts and notes. Behaviour category abstracted from LOC. No details on missing data.	15
	Chapman 2017[29]	UK	All in-patients	Case-control study (retrospective)	1	No	Ext	Death, arrest or PICU transfer	608 (297 cases)	S	4 / 9	Max 48hrs before event (per usual practice)	0.74	46.0	90.0	*81.3*	*63.0*	See above.	17
Children's Hospital Early Warning Score (CHEWS)	McLellan 2014[50]	US	All in-patients	Case-control study (retrospective)	1	Yes	Int	Arrest or unplanned PICU transfer	1,136 (360 cases)	S	4 / 12	Max in admission (4 hourly)	0.90	84.2	80.9			No details on missing data.	10
Children's Hospital Cardiac Early Warning Score (C-CHEWS)	McLellan 2013[23]	US	Cardiovascular unit	Case-control study (retrospective)	1	Yes	Int	Arrest or unplanned PICU transfer	312 (64 cases)	S	3 / 12	Max 18hrs before event (4 hourly)	0.86	95.3	76.2	*50.8*	*98.4*	Study nurse and bedside nurses assessed scores for 37 patients, 67% agreement. No details on missing data.	9
	Agulnik 2016[41]	US	Oncology unit	Case-control study (retrospective)	1	Yes	Ext	Unplanned PICU transfer	330 (110 cases)	S	4 / 12	Max 24 hours before event (4 hourly)	0.96	86.0	95.0			PTTT scores abstracted by researcher. Did not abstract if vital signs were present but no PTTT score calculated by nurse. No details on missing data.	14

For peer review only

	Agulnik 2017[42]	Guatemala	Oncology unit	Case-control study (retrospective)	1	Yes	Ext	Unplanned PICU transfer	258 (129 cases)	S	4 / 12	Max 24hrs before event (3 hourly)		91.0	88.0			Researcher evaluated charts and calculated scores, reporting 14% error rate (PTTT score calculated incorrectly) and 3% omission rate (vital signs recorded but no PTTT score calculated). 1 out of 130 cases excluded due to missing PTTT documentation.	16
Children's Hospital Los Angeles (CHLA) PEWS	Mandell 2015[40]	US	In-patients discharged from PICU to ward	Case-control study (retrospective)	1	Yes	Int	Early unplanned re-admission to PICU (within 48 hours of discharge from PICU to ward)	189 (38 cases)	S	2 / 10	First score assigned on ward, post PICU discharge	0.71	76.0	56.0			No details on missing data.	12
Melbourne Activation Criteria (MAC)	Tume 2007[3]	UK	In-patients with an unplanned PICU transfer	Chart review study (retrospective)	1	No	Ext	Unplanned PICU transfer	33 cases	T	N/A	Unclear		87.8				Data abstracted by two reviewers. Reference to "large number of missing records and observation charts".	11
	Tume 2007[3]	UK	In-patients with an unplanned PHDU transfer	Chart review study (retrospective)	1	No	Ext	Unplanned PHDU transfer	32 cases	T	N/A	Unclear		87.5				See above.	11

For peer review only - http://bmjopen.bmj.com/site/about/guidelines.xhtml

	Edwards 2011[33]	UK	All in-patients	Cohort study (retrospective)	1	No	Ext	Death or unplanned PICU or HDU transfer	1,000 (16 cases)	T	N/A	Any trigger over admission (per usual practice)	0.79	68.3	83.2	3.6	99.7	Observation charts altered to include all PTTT parameters. 56% of records missing at least one component. Missing data assumed to be normal.	17
	Chapman 2017[29]	UK	All in-patients	Case-control study (retrospective)	1	No	Ext	Death, arrest or PICU transfer	608 (297 cases)	T	N/A	Max 48hrs before event (per usual practice)	0.71	93.0	49.0	*64.0*	*88.0*	See above.	17
Cardiff & Vale Paediatric Early Warning Score (C&VPEWS)	Edwards 2009[32]	UK	All in-patients	Cohort study (prospective)	1	No	Int	Death or unplanned PICU or HDU transfer	1,000 (16 cases)	S	2 / 8	Max score during admission (per usual practice)	0.86	69.5	89.9	5.9	99.7	Observation charts altered to include all PTTT parameters. 56% of records missing at least one component. Missing data assumed to be normal.	18
	Chapman 2017[29]	UK	All in-patients	Case-control study (retrospective)	1	No	Ext	Death, arrest or PICU transfer	608 (297 cases)	S	3 / 8	Max 48hrs before event (per usual practice)	0.89	80.0	86.0	*84.0*	*82.0*	See above.	17
Bristol Paediatric Early Warning Tool (PEWT)	Tume 2007[3]	UK	In-patients with an unplanned PICU transfer	Chart review (retrospective)	1	No	Ext	Unplanned PICU transfer	33 cases	T	N/A	Unclear		87.8				See above.	11
	Tume 2007[3]	UK	In-patients with an unplanned PHDU transfer	Chart review (retrospective)	1	No	Ext	Unplanned PHDU transfer	32 cases	T	N/A	Unclear		84.4				See above.	11

For peer review only

	Wright 2011[35]	UK	All in-patients	Chart review (retrospective)	1	Yes	Ext	Cardiac arrest	55 cases	T	N/A	If triggered 24hrs before event		49.1				One case excluded due to missing notes. No details on missing data.	11
	O'Loughlin 2012[34]	UK	All in-patients	Cohort study (prospective)	1	Yes	Ext	PICU transfer	331 (7 cases)	T	N/A	Triggered during admission (12hrly)	0.91	100	81.0	11.0		No details on missing data.	6
	Robson 2013[28]	US	All in-patients	Case-control study (retrospective)	1	No	Ext	Code blue call	192 (96 cases)	T	N/A	Triggered 24hrs before event (6hrly)	0.75	76.3	61.5			See above.	8
	Chapman 2017[29]	UK	All in-patients	Case-control study (retrospective)	1	No	Ext	Death, arrest or PICU transfer	608 (297 cases)	T	N/A	If triggered 48hrs before event (per usual practice)	0.62	96.0	28.0	*56.0*	*88.0*	See above.	17
Modified Bristol Paediatric Early Warning Tool (PEWT) (b)	Clayson 2014[38]	UK	Cardiac ward	Cohort study (prospective)	1	Yes	Int	'A deteriorating patient'	126 (unclear number of cases)	T	N/A	Unclear				*12.5*	*97.0*	No details on missing data.	5
NHS Institute for Innovation and Improvement (NHS III) PEWS	Mason 2016[14]	UK	All in-patients	Cohort study (retrospective)	1	No	Ext	Death or unplanned PICU or HDU transfer	1,000 (16 cases)	S	2 / 7	Max score over admission (per usual practice)	0.88	80.0	81.0	4.3	99.7	Observation charts altered to include all PTTT parameters. 56% of records missing at least one component. Missing data assumed to be normal.	15

For peer review only - http://bmjopen.bmj.com/site/about/guidelines.xhtml

	Chapman 2017[29]	UK	All in-patients	Case-control study (retrospective)	1	No	Ext	Death, arrest or PICU transfer	608 (297 cases)	S	2 / 7	Max 48hrs before event (per usual practice)	0.82	83.0	65.0	*69.6*	*80.0*	See above.	17
Logistic regression algorithm	Zhai 2014[19]	US	All in-patients	Case-control study (retrospective)	1	No	Ext	Urgent PICU transfer within 24 hrs of admission	6,352 (53 cases)	S	> 0.5	Max 24hrs before event (hourly)	0.91	84.9	85.9	4.8		Data extracted from electronic health records. No details on extent of missing data but authors report that "missing data was a major cause of incorrect prediction".	17
Burton Paediatric Early Warning Score (BPEWS)	Ahmed 2012[36]	UK	PICU admissions only	Chart review (retrospective)	1	Yes	Int	PICU admission	23	S	4 / 19	Max 24hrs before event (unclear)		93.0				Data extracted from case notes by two reviewers. No details on missing data.	4
'Between the Flags' Paediatric Early Warning System (PEWS)	Blackstone 2017[43]	UK	Urgent PICU admissions only	Chart review (retrospective)	1	Yes	Ext	Urgent PICU admission	100	T	N/A	Unclear		91.0				Data extracted from health records. No details on missing data.	8

For peer review only - http://bmjopen.bmj.com/site/about/guidelines.xhtml

All studies conducted in a specialist / tertiary centre.

PPV and NPV values in italics represent results from case-control studies – these values are misleading in isolation because they assume that the wider prevalence rate of the adverse event is equal to the case to control ratio used in the research study (e.g., if the researchers studied 300 cases and 300 controls, the prevalence rate of adverse events for the calculation of PPV is 50%). As per the cohort studies, prevalence rates of critical events are typically far lower among hospitalised paediatric populations than the case/control ratios used in studies, and so PPV values would be considerably lower in clinical practice.

Studies classified as internal validation if the setting for the study was the same hospital and same research team as those who developed the score. Studies classified as external validation if the score was tested in a different centre and by a different research team to those who developed it.

* Typically, study researchers collected or abstracted multiple PTTT scores for each patient at different time points, but can only use one score per patient for the analysis of the tool’s predictive ability. This column specifies which score the researchers used. In most cases, the study team used the maximum PTTT score recorded for each patient in a given study window – e.g., 24 hours prior to a critical event for case patients. The text in parentheses describes the frequency with which scores were assessed or abstracted for each patient, if this information was described in the paper.

† Case-control study, but PPV value calculated based on clinical prevalence of event as measured at local centre during the study

AUROC, area under the receiver operating characteristic curve; Ext, external validation; HFNC, high flow nasal cannula; Int, Internal validation; Max, maximum; N/A, not applicable; NPV, negative predictive value; PHDU, paediatric high-dependency unit; PICU, paediatric intensive care unit; PPV, positive predictive value; PTTT, paediatric track and trigger tool; RRT, rapid response team; S, score; T, trigger; UK, United Kingdom; US, United States.

For peer review only

Table 4 – summary of early warning system effectiveness study outcome

Outcome	First author, year	Intervention: Implemented a new PTTT	Intervention: Implemented new RRT / MET	Intervention: Modified escalation process	Intervention: Staff training / education	PTTT	Country	Number of centres	Specialist unit?	Existing RRT / MET?	Population	Study design	Study duration in months	Events before, *n* (rate)	Events after, *n* (rate)	Effect size (95% CI)	P Value	Quality score (max = 26)
**MORTALITY**																		
Deaths on ward (per 1,000 admissions)	Tibballs 2005[13]	✓	✓		✓	Melbourne Activation Criteria	Australia	1	Y	N	All in-patients	Uncontrolled before-after study (prospective)	53 (41 before, 12 after)	13 (0.12)	2 (0.06)	RR = 0.45 (0.10-1.99) †	0.29	10
Hospital-wide deaths (per 100 discharges)	Sharek 2007[17]	✓	✓		✓	Paediatric Rapid Response Team triggering criteria	US	1	Y	N	All in-patients	Uncontrolled before-after study (prospective)	84 (67 before, 17 after)	547 (1.01)	158 (0.83)	RR = 0.82 (0.70-0.95)	**.007**	15
Hospital wide deaths, excluding neonate ICU and ED (per 1,000 discharges)	Zenker 2007[60]	✓	✓			RRT activation criteria*	US	1	Y	N	All in-patients	Uncontrolled before-after study (prospective)	34 (23 before, 11 after)	97 (4.30)	52 (4.45)	RR=1.04 (0.74-1.45) †	.57	12
Deaths outside ICU (per 1,000 non-ICU patient-days)	Brilli 2007[15]	✓	✓		✓	Paediatric Medical Emergency Team triggering criteria (a)	US	1	Y	N	All in-patients	Uncontrolled before-after study (prospective)	27 (15 before, 12 after)	9 (0.10)	2 (0.04)	RR=0.39 (0.08-1.80) †	.13	14

For peer review only - http://bmjopen.bmj.com/site/about/guidelines.xhtml

Ward death rate (per 1,000 ward admissions)	Hanson 2010[61]	✓	✓		✓	*Not described*	US	1	Y	N	All in-patients	Uncontrolled before-after study (retrospective)	36 (24 before, 12 after)	13 (1.50)	2 (0.45)	RR = 0.30 (0.07–1.31) †	.07	18
Total hospital deaths (per 1,000 admissions)	Tibballs 2009[62]	✓	✓		✓	Melbourne Activation Criteria	Australia	1	Y	N	All in-patients	Uncontrolled before-after study (prospective)	89 (41 before, 48 after)	459 (4.38)	398 (2.87)	RR = 0.65 (0.57-0.75)	**< .0001**	15
Deaths on ward (per 1,000 admissions)	Tibballs 2009[62]	✓	✓		✓	Melbourne Activation Criteria	Australia	1	Y	N	All in-patients	Uncontrolled before-after study (prospective)	89 (41 before, 48 after)	13 (0.12)	6 (0.04)	RR = 0.35 (0.13-0.92)	**.03**	15
All-cause hospital mortality (per 1,000 admissions)	Kotsakis 2011[63]	✓	✓			Modified Melbourne Activation Criteria	Canada	4	Y	N	All in-patients	Uncontrolled before-after study (prospective)	48 (24 before, 24 after)	553 (9.97)	540 (9.65)	RR = 0.97 (0.83-1.12)	.65	18
All cause hospital mortality (per 1,000 discharges)	Parshuram 2018[64]	✓	✓		✓	Bedside PEWS	Belgium, Ireland, Netherlands, England, Italy, Canada, New Zealand	21	Y	N	All in-patients	Cluster randomised trial (prospective)	18 (6 pre, 12 post)	Con: 61 (1.31)<br>Int: 52 (1.95)	Con: 147 (1.56)<br>Int: 97 (1.93)	OR=1.01 (0.61-1.69)	.96	23
Hospital mortality (per 1,000 admissions)	Kutty 2018[73]		✓			NR	US	38	Y	N	All in-patients	Interrupted Time Series (retrospective)	180 (60 before, 120 after)	NA	NA	OR=0.94 (0.93-0.95)	.98	20
**PICU MORTALITY**																		
PICU mortality after PICU admission from ward (per PICU admission)	Anwar-al-Haque, 2010[18]	✓	✓			Paediatric Rapid Response Team triggering criteria (b)	Pakistan	1	Y	N	All in-patients	Uncontrolled before-after study (retrospective)	18 (9 before, 9 after)	23 (51.11)	5 (15.63)	RR = 0.31 (0.13-0.72) †	**.007**†	6

For peer review only - http://bmjopen.bmj.com/site/about/guidelines.xhtml

PICU mortality after PICU readmission within 48 hrs of discharge (per 1,000 admissions)	Kotsakis 2011[63]	✓	✓			Modified Melbourne Activation Criteria	Canada	4	Y	N	All in-patients	Uncontrolled before-after study (prospective)	48 (24 before, 24 after)	16 (0.29)	7 (0.13)	RR = 0.43 (0.17-0.99)	**<.05**	18
PICU mortality after urgent PICU admission from ward (per 1,000 admissions)	Kotsakis 2011[63]	✓	✓			Modified Melbourne Activation Criteria	Canada	4	Y	N	All in-patients	Uncontrolled before-after study (prospective)	48 (24 before, 24 after)	70 (1.3)	61 (1.1)	RR = 0.90 (0.70-1.00)	.25	18
Death prior to discharge (per unplanned PICU transfer)	Bonafide 2014[65]	✓	✓			Bedside PEWS	US	1	Y	N	All in-patients	Interrupted Time Series study (prospective)	59 (32 before, 27 after)	51 (6.3)	56 (6.5)	RR = 1.03 (0.72-1.49) †	.99	23
PICU mortality (per PICU admission)	Duns 2014[66]	✓				Between the Flags (BTS) tool*	Australia	1	Y	Y	All in-patients	Uncontrolled before-after study (prospective)	48 (24 before, 24 after)	30 (8.57)	20 (5.49)	RR=0.64 (0.37-1.11) †	.14	7
Death in PICU (per 1,000 patient-days)	Agulnik 2017[67]	✓			✓	Children's Hospital Cardiac Early Warning Score (C-CHEWS)	Guatemala	1	Y	N	Oncology unit	Uncontrolled before-after study (retrospective)	24 (12 before, 12 after)	21 (1.25)	22 (1.10)	RR=0.89 (0.49-1.61) †	.76	19
Death in PICU (per emergency PICU admission)	Sefton 2015[68]	✓		✓	✓	Modified Bristol PEWT (a)	UK	1	Y	N	All PICU admissions	Controlled before-after study (retrospective)	24 (12 before, 12 after)	17 (10.8)	14 (8.4)	RR = 0.78 (0.40-1.53) †	.47	16
Deaths in PICU (per unplanned PICU admission)	Kolovos, 2018	✓	✓			RRT activation criteria*	US	1	Y	N	All unplanned PICU admissions	Uncontrolled before-after study (retrospective)	78 (42 before, 36 after)	54† (4.9)	40† (3.8)	RR = 0.77 (0.52–1.15) †	.20†	12

For peer review only - http://bmjopen.bmj.com/site/about/guidelines.xhtml

PICU mortality (per 1,000 discharges)	Parshuram 2018[64]	✓	✓		✓	Bedside PEWS	Belgium, Ireland, Netherlands, England, Italy, Canada, New Zealand	21	Y	N	All in-patients	Cluster randomised trial (prospective)	18 (6 pre, 12 post)	Con: 34 (0.73)<br>Int: 33 (1.24)	Con: 91 (0.96)<br>Int: 56 (1.12)	OR=0.95 (0.48-1.86)	.88	23
**CARDIAC ARREST**																		
Cardiac arrests on ward (per 1,000 admissions)	Tibballs 2005[13]	✓	✓		✓	Melbourne Activation Criteria	Australia	1	Y	N	All in-patients	Uncontrolled before-after study (prospective)	53 (41 before, 12 after)	20 (0.19)	4 (0.11)	RR = 0.58 (0.20-1.70)	.33	10
Cardiopulmonary arrests (per 1,000 non-ICU patient-days)	Brilli 2007[15]	✓	✓		✓	Paediatric Medical Emergency Team triggering criteria (a)	US	1	Y	N	All in-patients	Uncontrolled before-after study (prospective)	27 (15 before, 12 after)	7 (0.08)	2 (0.04)	RR=0.50 (0.10-2.42) †	.11	14
Ward cardiac arrest rate (per 1,000 ward admissions)	Hanson 2010[61]	✓	✓		✓	*Not described*	US	1	Y	N	All in-patients	Uncontrolled before-after study (retrospective)	36 (24 before, 12 after)	11 (1.27)	2 (0.45)	RR = 0.35 (0.08–1.58) †	.13	18
Ward cardiopulmonary arrests (per 1,000 patient-days)	Hunt 2008[16]	✓	✓			Paediatric Medical Emergency Team triggering criteria	US	1	Y	N	All in-patients	Uncontrolled before-after study (prospective)	24 (12 before, 12 after)	5 (0.10)	5 (0.10)	RR = 0.98 (0.22–4.24)	.97	17
Preventable cardiac arrests (per 1,000 admissions)	Tibballs 2009[62]	✓	✓		✓	Melbourne Activation Criteria	Australia	1	Y	N	All in-patients	Uncontrolled before-after study (prospective)	89 (41 before, 48 after)	17 (0.16)	10 (0.07)	RR = 0.45 (0.20-0.97)	**.04**	15

For peer review only - http://bmjopen.bmj.com/site/about/guidelines.xhtml

Unexpected cardiac arrests (per 1,000 admissions)	Tibballs 2009[62]	✓	✓		✓	Melbourne Activation Criteria	Australia	1	Y	N	All in-patients	Uncontrolled before-after study (prospective)	89 (41 before, 48 after)	20 (0.19)	24 (0.17)	RR = 0.91 (0.50-1.64)	.75	15
Actual cardiopulmonary arrests (per 1,000 ward admissions)	Kotsakis 2011[63]	✓	✓			Modified Melbourne Activation Criteria	Canada	4	Y	N	All in-patients	Uncontrolled before-after study (prospective)	48 (24 before, 24 after)	69 (1.9)	66 (1.8)	RR = 0.95 (0.76-1.96)	.68	18
Near cardiopulmonary arrests (per 1,000 admissions)	Kotsakis 2011[63]	✓	✓			Modified Melbourne Activation Criteria	Canada	4	Y	N	All in-patients	Uncontrolled before-after study (prospective)	48 (24 before, 24 after)	123 (3.4)	67 (1.9)	RR = 0.54 (0.52-0.57)	**<.001**	18
Cardiac arrests on ward (per 1,000 non-ICU patient-days)	Bonafide 2014[65]	✓	✓			Bedside PEWS	US	1	Y	N	All in-patients	Interrupted Time Series study (prospective)	59 (32 before, 27 after)	6† (0.03)	2† (0.01)	RR = 0.36 (0.07-1.78) †	.21	23
Cardiac arrests (per 1,000 patient-days)	Parshuram 2018[64]	✓	✓		✓	Bedside PEWS	Belgium, Ireland, Netherlands, England, Italy, Canada, New Zealand	21	Y	N	All in-patients	Cluster randomised trial (prospective)	18 (6 pre, 12 post)	Con: 18 (0.11)<br>Int: 15 (0.12)	Con: 32 (0.10)<br>Int: 27 (0.11)	RR=1.02 (0.65-1.62)	.92	23
**RESPIRATORY ARREST**																		
Ward respiratory arrests (per 1,000 patient-days)	Hunt 2008[16]	✓	✓			Paediatric Medical Emergency Team triggering criteria	US	1	Y	N	All in-patients	Uncontrolled before-after study (prospective)	24 (12 before, 12 after)	11 (0.23)	3 (0.06)	RR = 0.27 (0.07-0.95)	**.04**	17
**CARDIAC OR RESPIRATORY ARREST**																		

For peer review only - http://bmjopen.bmj.com/site/about/guidelines.xhtml

Cardiac or respiratory arrest (per 1,000 discharges)	Zenker 2007[60]	✓	✓			RRT activation criteria*	US	1	Y	N	All in-patients	Uncontrolled before-after study (prospective)	34 (23 before, 11 after)	180 (7.98)	60 (5.13)	RR=0.64 (0.48-0.86) †	.19	12
Code calls (per 1,000 non-ICU patient-days)	Brilli 2007[15]	✓	✓		✓	Paediatric Medical Emergency Team triggering criteria (a)	US	1	Y	N	All in-patients	Uncontrolled before-after study (prospective)	27 (15 before, 12 after)	25 (0.27)	6 (0.11)	RR=0.42 (0.17-1.03) †	.06†	14
Code calls (per 1,000 non-ICU patient-days)	Sharek 2007[17]	✓	✓		✓	Paediatric Rapid Response Team triggering criteria	US	1	Y	N	All in-patients	Uncontrolled before-after study (prospective)	84 (67 before, 17 after)	53 (0.52)	5 (0.15)	RR = 0.29 (0.10-0.65)	**.008**	15
Code calls (per 1,000 admissions)	Anwar-al-Haque 2010[18]	✓	✓			Paediatric Rapid Response Team triggering criteria (b)	Pakistan	1	Y	N	All in-patients	Uncontrolled before-after study (retrospective)	18 (9 before, 9 after)	26 (5.25)	12 (2.73)	RR = 0.52 (0.26-1.03)	.06	6
**CALLS FOR URGENT REVIEW / ASSISTANCE**																		
Urgent calls to respiratory therapist (per 1,000 patient-days)	Parshuram 2011[70]	✓		✓	✓	Bedside PEWS	Canada	1	N	N	All in-patients	Uncontrolled before-after study (prospective)	8 (3 before, 5 after)	8 (9.5)	8 (3.4)	RR = 0.36 (0.13-0.95) †	**.04**†	23
Urgent calls to paediatrician (per 1,000 patient-days)	Parshuram 2011[70]	✓		✓	✓	Bedside PEWS	Canada	1	N	N	All in-patients	Uncontrolled before-after study (prospective)	8 (3 before, 5 after)	19 (22.6)	12 (5.1)	RR = 0.23 (0.11-0.46) †	**<.0001**	23
Code blue calls on the ward (per 1,000 admissions)	Kotsakis 2011[63]	✓	✓			Modified Melbourne Activation Criteria	Canada	4	Y	N	All in-patients	Uncontrolled before-after study (prospective)	48 (24 before, 24 after)	210 (3.75)	150 (2.70)	RR = 0.71 (0.61-0.83)	**<.0001**	18

For peer review only - http://bmjopen.bmj.com/site/about/guidelines.xhtml

Urgent calls to outreach team (per 1,000 admissions)	Duns 2014[66]	✓				Between the Flags tool*	Australia	1	Y	Y	All in-patients	Uncontrolled before-after study (prospective)	48 (24 before, 24 after)	1,058 (39.5)	2,120 (76.0)	RR=1.92 (1.79-2.07) †	**.02**	7
RRT calls (per 1,000 patient-days)	Panesar 2014[71]			✓		Modified Brighton PEWS (e)	US	1	Y	Y	All in-patients	Uncontrolled before-after study (retrospective)	42 (18 before, 24 after)	44 (3.14)	69 (4.23)	RR = 1.35 (0.92-1.96) †	.11	15
RRT calls (per 1,000 patient days)	Douglas 2016[72]	✓		✓	✓	Modified Brighton PEWS (b)	US	1	Y	Y	All in-patients	Uncontrolled before-after study (retrospective)	24 (12 before, 12 after)	194 (6.17)	292 (9.80)	RR = 1.59 (1.33-.1.90) †	**<.001**	12
Code calls (per 1,000 patient days)	Douglas 2016[72]	✓		✓	✓	Modified Brighton PEWS (b)	US	1	Y	Y	All in-patients	Uncontrolled before-after study (retrospective)	24 (12 before, 12 after)	31 (0.98)	20 (0.67)	RR = 0.68 (0.39-1.19) †	.21	12
**PICU TRANSFERS**																		
Transfers from ward to other specialist units (per 1,000 patient-days)	Parshuram 2011[70]	✓		✓	✓	Bedside PEWS	Canada	1	N	N	All in-patients	Uncontrolled before-after study (prospective)	8 (3 before, 5 after)	5 (5.9)	19 (8.1)	RR = 1.37 (0.51-3.63) †	.54†	23
Clinical deterioration events on ward prior to transfer to specialist unit (per 1,000 patient-days)	Parshuram 2011[70]	✓		✓	✓	Bedside PEWS	Canada	1	N	N	All in-patients	Uncontrolled before-after study (prospective)	8 (3 before, 5 after)	2 (2.4)	1 (0.43)	RR = 0.18 (0.02-1.97) †	.16†	23

For peer review only - http://bmjopen.bmj.com/site/about/guidelines.xhtml

PICU transfers (per 1,000 admissions)	Duns 2014[66]	✓				Between the Flags tool*	Australia	1	Y	Y	All in-patients	Uncontrolled before-after study (prospective)	48 (24 before, 24 after)	350 (13.1)	364 (13.1)	RR=1.00 (0.86-1.16) †	.98	7
Unplanned PICU transfers from ward (per 1,000 non-ICU patient-days)	Bonafide 2014[65]	✓	✓			Bedside PEWS	US	1	Y	N	All in-patients	Interrupted Time Series study (prospective)	59 (32 before, 27 after)	874 (4.54)	936 (5.25)	IRR = 0.73 (0.46–1.14)	.16	23
Unplanned transfers to PICU from ward (per 1,000 patient-days)	Agulnik 2017[67]	✓			✓	Children's Hospital Cardiac Early Warning Score	Guatemala	1	Y	N	Oncology unit	Uncontrolled before-after study (retrospective)	24 (12 before, 12 after)	157 (9.3)	130 (6.5)	RR = 0.70 (0.56-0.88) †	**.003**	19
Urgent PICU admissions (per 1,000 patient-days)	Parshuram 2018[64]	✓	✓		✓	Bedside PEWS	Belgium, Ireland, Netherlands, England, Italy, Canada, New Zealand	21	Y	N	All in-patients	Cluster randomised trial (prospective)	18 (6 pre, 12 post)	Con: 652 (4.01)<br>Int: 469 (3.62)	Con: 1178 (3.83)<br>Int: 828 (3.29)	RR=0.95 (0.82-1.09)	.45	23
**PICU OUTCOMES**																		
Critical deterioration events after PICU transfer (per 1,000 non-ICU patient-days)	Bonafide 2014[65]	✓	✓			Bedside PEWS	US	1	Y	N	All in-patients	Interrupted Time Series study (prospective)	59 (32 before, 27 after)	260† (1.35)	282† (1.58)	IRR = 0.38 (0.20-0.75)	**.01**	23

For peer review only - http://bmjopen.bmj.com/site/about/guidelines.xhtml

Mechanical ventilation within 1hr of unplanned PICU transfer (per unplanned transfer to PICU)	Bonafide 2014[65]	✓	✓			Bedside PEWS	US	1	Y	N	All in-patients	Interrupted Time Series study (prospective)	59 (32 before, 27 after)	45 (5.1)	42 (4.5)	RR = 0.87 (0.58-1.31) †	.51	23
Mechanical ventilation within 12hrs of unplanned PICU transfer (per unplanned transfer to PICU)	Bonafide 2014[65]	✓	✓			Bedside PEWS	US	1	Y	N	All in-patients	Interrupted Time Series study (prospective)	59 (32 before, 27 after)	112 (12.8)	103 (11.0)	IRR = 0.17 (0.07-0.44)	**<0.001**	23
Vasopressor within 1hr of unplanned PICU transfer (per unplanned transfer to PICU)	Bonafide 2014[65]	✓	✓			Bedside PEWS	US	1	Y	N	All in-patients	Interrupted Time Series study (prospective)	59 (32 before, 27 after)	41 (4.7)	16 (1.7)	RR = 0.36 (0.21-0.64) †	**<0.001**	23
Vasopressor s within 12hrs of unplanned PICU transfer (per unplanned transfer to PICU)	Bonafide 2014[65]	✓	✓			Bedside PEWS	US	1	Y	N	All in-patients	Interrupted Time Series study (prospective)	59 (32 before, 27 after)	71 (8.1)	57 (6.1)	IRR = 0.20 (0.06-0.62)	**.006**	23
Invasive ventilation in PICU (per emergency PICU admission)	Sefton 2015[68]	✓		✓	✓	Modified Bristol PEWT (a)	UK	1	Y	N	All PICU admissions	Controlled before-after study (retrospective)	24 (12 before, 12 after)	118 (75.2)	104 (62.7)	RR = 0.83 (0.72-0.97) †	**.002**	16

For peer review only

For peer review only - http://bmjopen.bmj.com/site/about/guidelines.xhtml

Inotropes in PICU (per emergency PICU admission)	Sefton 2015[68]	✓		✓	✓	Modified Bristol PEWT (a)	UK	1	Y	N	All PICU admissions	Controlled before-after study (retrospective)	24 (12 before, 12 after)	50 (31.8)	40 (24.1)	RR = 0.76 (0.53-1.08) †	.12	16
Intubation within 24hrs of PICU admission (per 1,000 patient-days)	Agulnik 2017[67]	✓			✓	Children's Hospital Cardiac Early Warning Score	Guatemala	1	Y	N	Oncology unit	Uncontrolled before-after study (retrospective)	24 (12 before, 12 after)	11 (0.65)	18 (0.90)	RR=1.38 (0.65-2.92) †	.46	19
Vasopressors within 24hrs of PICU admission (per 1,000 patient-days)	Agulnik 2017[67]	✓			✓	Children's Hospital Cardiac Early Warning Score	Guatemala	1	Y	N	Oncology unit	Uncontrolled before-after study (retrospective)	24 (12 before, 12 after)	29 (1.72)	37 (1.86)	RR=1.08 (0.66-1.75) †	.60	19
Mechanical ventilation during PICU admission (per PICU admission)	Kolovos 2018[69]	✓	✓			RRT activation criteria*	US	1	Y	N	All unplanned PICU admissions	Uncontrolled before-after study (retrospective)	78 (42 before, 36 after)	285 (25.98)	233 (22.09)	RR = 0.85 (0.73-0.99) †	**.03**†	12
Intubation within 1hr of PICU admission (per PICU admission)	Kolovos 2018[69]	✓	✓			RRT activation criteria*	US	1	Y	N	All unplanned PICU admissions	Uncontrolled before-after study (retrospective)	78 (42 before, 36 after)	49 (4.47)	88 (8.34)	RR = 1.87 (1.33-2.62)	**.0003**	12
Significant clinical deterioration events (per 1,000 patient-days)	Parshuram 2018[64]	✓	✓		✓	Bedside PEWS	Belgium, Ireland, Netherlands, England, Italy, Canada, New Zealand	21	Y	N	All in-patients	Cluster randomised trial (prospective)	18 (6 pre, 12 post)	Con: 144 (0.89)<br>Int: 80 (0.62)	Con: 259 (0.84)<br>Int: 127 (0.50)	RR=0.77 (0.61-0.97)	**.03**	23

For peer review only - http://bmjopen.bmj.com/site/about/guidelines.xhtml

For peer review only

P-values in bold denote statistical significance (<0.05).

A critical deterioration event is defined as transfer to the intensive care unit (ICU) followed by non-invasive or invasive mechanical ventilation or vasopressor infusion within 12 hours[65]

*Indicates a PTTT not described or validated in the published literature

† Data calculated by research team, based on data presented in the journal article. All data calculated via https://www.medcalc.org.

Con, Control group; ED, emergency department; Int, Intervention group; IRR, incident risk ratio; MET, medical emergency team; OR, odds ratio; PICU, paediatric intensive care unit; PTTT, paediatric track and trigger tool; RRT, rapid response team; RR, relative risk.

For peer review only

For peer review only - http://bmjopen.bmj.com/site/about/guidelines.xhtml

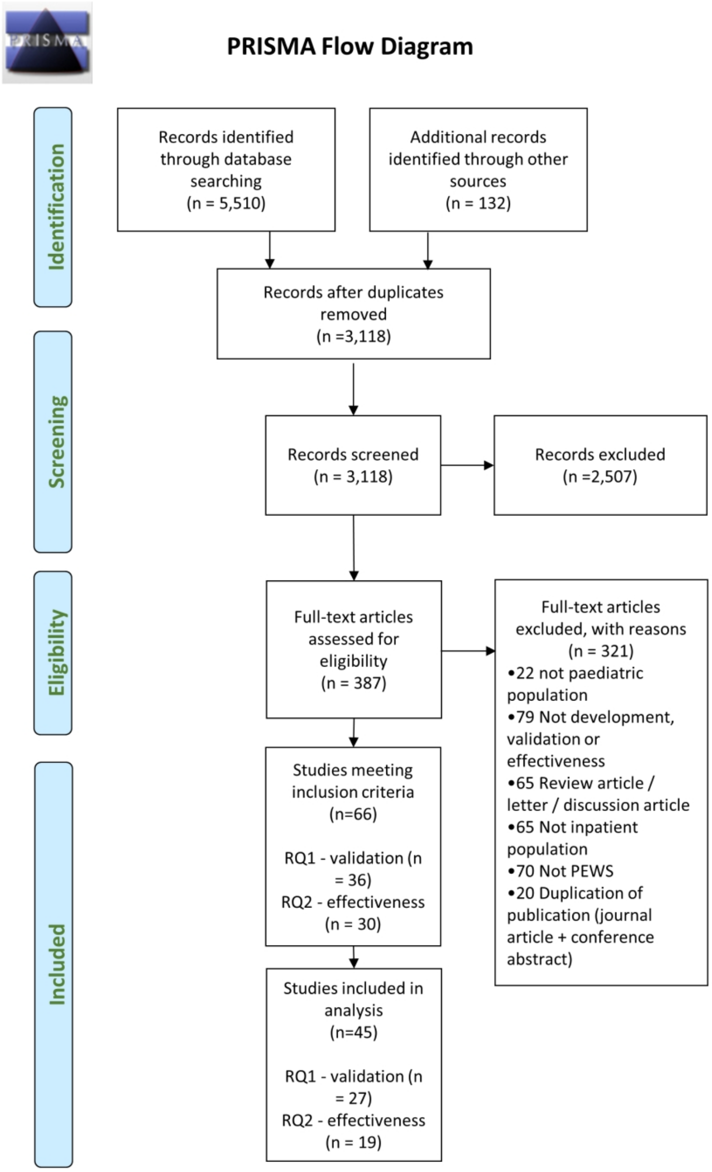

Figure 1: PRISMA flow diagram of study inclusion

190x254mm (300 x 300 DPI)

Supplementary Table 1 – Details of search strategy

Database Search

The search was across a range of databases from their inception to January 2015 then an update was carried out in September 2016 and the second update May 2018.

A preliminary search strategy was developed using a set of key papers known to the group for Ovid Medline using both text words and Medical subject headings. The search strategy was modified according to the indexing systems of the other databases.

Databases and Database platform	Original search results January 2015	Update September 2016	Update May 2018
British Nursing Index (Proquest)	19	12	25
CINAHL (Cumulative Index of Nursing and Allied Health Literature) (Ebsco)	206	17	29
Cochrane Central Register of Controlled Trials (Wiley)	43	4	30
EMBASE (OVID)	1065	206	431
HMIC (Health Management Information Centre) (OVID)	70	1	75
Medline (OVID)	943	135	328
Medline in Process (OVID)	43	69	45
Scopus (Elsevier)	747	85	234
Web of Knowledge (Science Science Citation Indexes) (Thomson Reuter)	400	82	166
**Total**	**3536** *(prior to removing duplicates and irrelevant studies)*	**611** *(prior to removing duplicates and irrelevant studies)*	**1363** *(prior to removing duplicates and irrelevant studies)*

Supplementary search

For peer review only

Search Information

Supplementary search

NB. Restricted each of the below searches by dates: 01/01/2016 – 16/05/2018

Trials Registers	Hits January 2015	Update September 2016	Update June 2018
ClinicalTrials.gov https://clinicaltrials.gov/	6	4	0
UK Clinical Trials Gateway http://www.ukctg.nihr.ac.uk/default.aspx	3 (duplicates)	5 (1 duplicate)	0
The WHO trial search portal for studies worldwide: http://apps.who.int/trialsearch	1 (duplicate)	0	0
Journal site	Hits		
Archives of Disease in Childhood http://adc.bmj.com/	14	4	7
BMJ http://www.bmj.com/theBMJ	1	0	1
BMJ Quality and safety http://qualitysafety.bmj.com/	7	4	2
JAMA Pediatrics http://archpedi.jamanetwork.com/journal.aspx	1	0	0
Journal of Critical Care http://www.jccjournal.org/	3	1	0
Journal of Pediatrics (American) http://www.jpeds.com/	1	0	2
Journal of Paediatrics and Child Health (Australian) http://onlinelibrary.wiley.com/journal/10.1111/(ISSN)1440-1754	2	2	0

For peer review only

Lancet<br>http://www.thelancet.com/	0	0	0
New England Journal of Medicine<br>http://www.nejm.org/	0	0	0
Pediatrics<br>http://pediatrics.aappublications.org/	6	2	0
Pediatric Critical Care Medicine<br>http://journals.lww.com/pccmjournal/pages/default.aspx	14	6	3
Websites and organisations	HITS		
American Society of Anesthesiologists<br>https://www.asahq.org/	1	0	0
American Academy of Pediatrics<br>http://www.aap.org/en-us/Pages/Default.aspx	1		0
Association of Anaesthetists of Great Britain and Ireland<br>http://www.aagbi.org/	0	0	0
Australian Medical Council<br>http://www.amc.org.au/	1	0	0
Royal College of Paediatrics and Child Health<br>http://www.rcpch.ac.uk/	1	0	4
Paediatric Nursing Association Europe<br>http://www.rcn.org.uk/	9		0
European Federation of Critical Care Nursing Associations<br>http://www.efccna.org/	No Search Option	No Search Option	No Search Option
Royal Australasian College of Physicians (Division of Child Health)<br>https://www.racp.edu.au/page/paed-policy	0	0	0
Royal College of Physicians (inclusive of National Clinical Guideline Centre)<br>https://www.rcplondon.ac.uk/	2	0	0
The NHS Institute for Innovation and Improvement<br>http://www.institute.nhs.uk/	4	Site cease to exist	Site cease to exist
NICE: Eyes on Evidence	4	1	1

For peer review only

https://www.evidence.nhs.uk/about-evidence-services/bulletins-and-alerts/eyes-on-evidence			
**TOTAL**	82	30	20

Total = 112

Search Strategies

British Nursing Index
"Paediatric Early Warning" OR ("pediatric early warning" OR "pediatric rapid response") OR ("paediatric rapid response" OR "Bedside paediatric early warning") OR ("Pediatric Advanced Warning Score" OR "Paediatric Advanced Warning Score")

Cochrane Controlled Register of Trials (CENTRAL)
Last Saved: 16/05/2018 11:39:08.703
Description:

ID	Search
#1	"early warning score*"
#2	"early warning system*"
#3	"early warning tool*"
#4	"VitalPAC Early Warning Score"
#5	"activation criteria"
#6	"Rapid Response Team"
#7	"Rapid Response system*"
#8	"Track and trigger"
#9	"trigger tools"
#10	"calling criteria"
#11	"Alert criteria"

For peer review only

#12 "Rapid Response"
#13 #1 or #2 or #3 or #4 or #5 or #6 or #7 or #8 or #9 or #10 or #11 or #12
#14 pediatric* or paediatric* or infant* or child* or baby or toddler or babies or teen* or adolescent*
#15 #13 and #14
#16 "Pediatric Early Warning"
#17 "Paediatric Early Warning"
#18 "p?ediatric alert"
#19 "Pediatric Rapid Response"
#20 "Pediatric Advanced Warning Score*"
#21 "Paediatric Advanced Warning Score*"
#22 "infant early warning"
#23 "Bedside PEWS"
#24 "Bedside paediatric early warning"
#25 #16 or #17 or #18 or #19 or #20 or #21 or #22 or #23 or #24
#26 #15 or #25 Publication Year from 2016 to 2018

Cumulative Index to Nursing and Allied Health Literature (CINAHL) via EBSCO

	Search ID#	Search Terms
☐	S11	S7 OR S10
☐	S10	S1 AND S8
☐	S9	S2 AND S8
☐	S8	S3 AND S4
☐	S7	S5 OR S6
☐	S6	TX "infant early warning" OR TX "bedside PEWS" OR TX "Bedside paediatric early warning"
☐	S5	TX "p?ediatric early warning system" OR TX "P?ediatric Early Warning" OR TX "p?ediatric early warning score" OR TX "p?ediatric risk of mortality" OR TX "P?ediatric Rapid Response Team" OR TX "P?ediatric alert"
☐	S4	AB pediatric* or paediatric* or infant*1 or child* or baby or toddler or babies or teen* or adolescent*

For peer review only

☐	S3	TX "track-and-trigger" OR TX "VitalPAC Early Warning Score" OR TX "activation criteria". OR TX "trigger tool*" OR TX "Rapid Response" OR TX "activation criteria". OR TX "early warning" OR TX "Alert criteria" OR TX outreach N3 emergency
☐	S2	Detecting W3 deterioration
☐	S1	"early warning"

Database of Abstracts of Review of Effects (DARE)
(Paediatric early warning) OR (pediatric early warning) OR (Paediatric Rapid Response) IN DARE
(early warning) OR (track-and-trigger system) OR (Rapid Response) IN DARE
(emergency team) AND (early warning) IN DARE

Excerpta Medica Database (EMBASE)
Database: EMBASE <1947-Present>
Search Strategy:
----
1 ("early warning" adj5 scor*).ab,ti. (568)
2 ("early warning" adj5 system* adj5 (deteriorat* or mortality or death or outcome* or harm* or safety)).ab,ti. (51)
3 "acute illness severity".mp. (38)
4 early intervention/ and ((prevent* or reduc* or improv*) adj5 (deteriorat* or mortality or death or outcome* or harm* or safety)).ab,ti. (1185)
5 ("early medical intervention" adj5 (tool* or scor* or index* or indicator* or indice* or assessment* or guide* or instrument* or criteria or parameter* or deteriorat* or mortality or death or monitor* or outcome* or harm* or safety)).ab,ti. (10)
6 *"severity of illness index"/ and ((tool* or scor* or index* or indicator* or indice* or assessment* or instrument* or criteria or parameter*) adj5 ((prevent* or reduc* or improv*) adj5 (deteriorat* or mortality or death or outcome* or harm* or safety))).ab,ti. (3)
7 exp Health Status Indicators/ and ((tool* or scor* or index* or indicator* or indice* or assessment* or instrument* or criteria or parameter*) adj3 ((prevent* or reduc* or improv*) adj3 (deteriorat* or mortality or death or outcome* or harm* or safety))).ab,ti. (7)
8 rapid response team/ (849)
9 "alarm monitor"/ and (prevent* or reduc* or improv*).mp. (245)
10 ("clinical alarm" adj5 (prevent* or reduc* or improv*)).mp. (2)
11 (outreach adj3 emergency).tw. (46)
12 VitalPAC Early Warning Score.tw. (15)
13 medical emergency team.tw. (395)
14 Rapid Response Systems.mp. (140)

For peer review only

15 ("rapid response" adj5 (prevent* or reduc* or improv*)).tw. (191)
16 ("medical device" adj3 (prevent* or reduc* or improv*)).mp. (187)
17 (((Detecting or managing) adj3 deterioration) and warning).tw. (11)
18 track-and-trigger system.tw. (24)
19 (Track adj trigger).tw. (4)
20 (Track and trigger).tw. (241)
21 trigger tools.tw. (47)
22 ("alert criteria" or "activation criteria" or "calling criteria").tw. (209)
23 SBAR technique*.mp. (5)
24 (score adj3 severity of illness).tw. (393)
25 or/1-24 (4295)
26 limit 25 to (infant <to one year> or child <unspecified age> or preschool child <1 to 6 years> or school child <7 to 12 years> or adolescent <13 to 17 years>) (533)
27 P?ediatric Early Warning.mp. (120)
28 p?ediatric alert.tw. (7)
29 p?ediatric early warning systems.mp. (4)
30 p?ediatric risk of mortality.tw. (527)
31 P?ediatric Rapid Response Team.tw. (14)
32 Point-of-Care Systems/ and ((paediatric or pediatric) adj3 (improve or identify or detect* or outcome or early or critical or emergency)).tw. (23)
33 P?ediatric Advanced Warning Score.tw. (3)
34 neonatal early warning.tw. (1)
35 infant early warning.tw. (0)
36 p?ediatric rapid response.tw. (31)
37 Bedside paediatric early warning.tw. (5)
38 Bedside PEWS.tw. (7)
39 or/27-38 (707)
40 26 or 39 (1155)
41 limit 40 to human (1065)

Health Management Information Consortium (HMIC) database
Database: HMIC Health Management Information Consortium
Search Strategy:

For peer review only

1 ("early warning" adj5 scor*).ab,ti. (23)
2 ("early warning" adj5 system* adj5 (deteriorat* or mortality or death or outcome* or harm* or safety)).ab,ti. (6)
3 "acute illness severity".mp. (3)
4 "early medical intervention"/ and ((prevent* or reduc* or improv*) adj5 (deteriorat* or mortality or death or outcome* or harm* or safety)).ab,ti. (0)
5 ("early medical intervention" adj5 (tool* or scor* or index* or indicator* or indice* or assessment* or guide* or instrument* or criteria or parameter* or deteriorat* or mortality or death or monitor* or outcome* or harm* or safety)).ab,ti. (0)
6 Health Status Indicators.mp. and ((tool* or scor* or index* or indicator* or indice* or assessment* or instrument* or criteria or parameter*) adj3 ((prevent* or reduc* or improv*) adj3 (deteriorat* or mortality or death or outcome* or harm* or safety))).ab,ti. (0)
7 exp "Severity of illness index"/ and ((tool* or scor* or index* or indicator* or indice* or assessment* or instrument* or criteria or parameter*) adj5 ((prevent* or reduc* or improv*) adj5 (deteriorat* or mortality or death or outcome* or harm* or safety))).ab,ti. (0)
8 "activation criteria".ab,ti. (2)
9 exp Rapid response teams/ (39)
10 Clinical Alarms.mp. (0)
11 (outreach adj3 emergency).tw. (2)
12 VitalPAC Early Warning Score.tw. (0)
13 medical emergency team.tw. (15)
14 Rapid Response Systems.mp. (8)
15 Rapid Response Team.tw. (27)
16 ((Detecting or managing) adj3 deterioration).tw. (1)
17 track-and-trigger system.tw. (2)
18 (Track adj trigger).tw. (1)
19 (Track and trigger).tw. (8)
20 trigger tools.tw. (4)
21 Calling criteria.tw. (1)
22 Alert criteria.mp. (1)
23 Rapid response.tw. (111)
24 (score adj3 severity of illness).tw. (3)
25 or/1-24 (171)
26 (pediatric* or paediatric* or infant*1 or child* or baby or toddler or babies or teen* or adolescent*).mp. (40161)
27 25 and 26 (14)
28 p?ediatric alert.tw. (0)
29 p?ediatric early warning systems.mp. (1)
30 p?ediatric risk of mortality.tw. (4)

For peer review only

31 Pediatric Rapid Response Team.tw. (0)
32 Point-of-Care.mp. and ((paediatric or pediatric) adj3 (improve or identify or detect* or outcome or early or critical or emergency)).tw. (0)
33 Pediatric Advanced Warning Score.tw. (0)
34 neonatal early warning.tw. (0)
35 infant early warning.tw. (0)
36 paediatric rapid response.tw. (1)
37 pediatric rapid response.tw. (0)
38 Bedside paediatric early warning.tw. (0)
39 Bedside PEWS.tw. (0)
40 p?ediatric early warning.mp. (2)
41 care.mp. and ((paediatric or pediatric) adj3 (improve or identify or detect* or outcome or early or critical or emergency)).tw. [mp=title, other title, abstract, heading words] (57)
42 or/28-41 (59)
43 27 or 42 (70)

Medline
Database: Ovid MEDLINE(R) <1946 to January Week 2 2015>
Search Strategy:
----
1 ("early warning" adj5 scor*).ab,ti. (260)
2 ("early warning" adj5 system* adj5 (deteriorat* or mortality or death or outcome* or harm* or safety)).ab,ti. (24)
3 "acute illness severity".mp. (21)
4 "early medical intervention"/ and ((prevent* or reduc* or improv*) adj5 (deteriorat* or mortality or death or outcome* or harm* or safety)).ab,ti. (99)
5 ("early medical intervention" adj5 (tool* or scor* or index* or indicator* or indice* or assessment* or guide* or instrument* or criteria or parameter* or deteriorat* or mortality or death or monitor* or outcome* or harm* or safety)).ab,ti. (7)
6 exp Health Status Indicators/ and ((tool* or scor* or index* or indicator* or indice* or assessment* or instrument* or criteria or parameter*) adj3 ((prevent* or reduc* or improv*) adj3 (deteriorat* or mortality or death or outcome* or harm* or safety))).ab,ti. (166)
7 "Severity of Illness Index"/ and ((tool* or scor* or index* or indicator* or indice* or assessment* or instrument* or criteria or parameter*) adj5 ((prevent* or reduc* or improv*) adj5 (deteriorat* or mortality or death or outcome* or harm* or safety))).ab,ti. (274)
8 exp Hospitals/ and ((Detecting or managing) adj3 deterioration).tw. (2)
9 ("medical device" adj3 (prevent* or reduc* or improv*)).mp. (58)
10 ("alert criteria" or "activation criteria" or "calling criteria").tw. (121)

For peer review only

11 Hospital Rapid Response Team/ (334)
12 Clinical Alarms/ (332)
13 (outreach adj3 emergency).tw. (32)
14 VitalPAC Early Warning Score.tw. (10)
15 medical emergency team.tw. (247)
16 Rapid Response Systems.mp. (87)
17 Rapid Response Team.tw. (185)
18 (((Detecting or managing) adj3 deterioration) and warning).tw. (8)
19 track-and-trigger system.tw. (14)
20 (Track adj trigger).tw. (2)
21 (Track and trigger).tw. (137)
22 trigger tools.tw. (22)
23 SBAR technique*.mp. (3)
24 ("rapid response" adj5 (prevent* or reduc* or improv*)).tw. (117)
25 (score adj3 severity of illness).tw. (243)
26 or/1-25 (2286)
27 limit 26 to (humans and "all child (0 to 18 years)") (453)
28 P?ediatric Early Warning.mp. (38)
29 p?ediatric alert.tw. (5)
30 p?ediatric early warning systems.mp. (3)
31 p?ediatric risk of mortality.tw. (400)
32 P?ediatric Rapid Response Team.tw. (6)
33 Point-of-Care Systems/ and ((paediatric or pediatric) adj3 (improve or identify or detect* or outcome or early or critical or emergency)).tw. (79)
34 P?ediatric Advanced Warning Score.tw. (2)
35 neonatal early warning.tw. (0)
36 infant early warning.tw. (0)
37 p?ediatric rapid response.tw. (20)
38 Bedside paediatric early warning.tw. (2)
39 Bedside PEWS.tw. (2)
40 or/28-39 (542)
41 27 or 40 (943)

Scopus

For peer review only

(TITLE-ABS-KEY ("Paediatric Early Warning" OR "Pediatric Early Warning" OR "Pediatric Advanced Warning Score" OR "Paediatric Advanced Warning Score" OR "neonatal early warning" OR "infant early warning" OR "pediatric rapid response" OR "Paedatric rapid response")) OR (((TITLE-ABS-KEY ("early warning" W/5 scor*)) OR (TITLE-ABS-KEY ("Rapid Response")) OR (TITLE-ABS-KEY ("track-and-trigger system")) OR (TITLE-ABS-KEY ("track and trigger")) OR (TITLE-ABS-KEY ("trigger tool*")) OR (TITLE-ABS-KEY ("alert criteria")) OR (TITLE-ABS-KEY ("activation criteria")) OR (TITLE-ABS-KEY ("VitalPAC Early Warning Score"))) AND (TITLE-ABS-KEY (pediatric* OR paediatric* OR infant* OR child* OR baby OR toddler OR babies OR teen* OR adolescent*))) AND (LIMIT-TO (SUBJAREA , "MEDI") OR LIMIT-TO (SUBJAREA , "NURS") OR LIMIT-TO (SUBJAREA , "NEUR"))

Web of Science

# 19	400	#17 OR #1<br>**Refined by:** [excluding] **WEB OF SCIENCE CATEGORIES:** (PARASITOLOGY OR PUBLIC ENVIRONMENTAL OCCUPATIONAL HEALTH OR BIOCHEMISTRY MOLECULAR BIOLOGY OR OPTICS OR HEALTH CARE SCIENCES SERVICES OR MYCOLOGY OR MANAGEMENT OR LINGUISTICS OR INSTRUMENTS INSTRUMENTATION OR MICROBIOLOGY OR INFORMATION SCIENCE LIBRARY SCIENCE OR MATHEMATICAL COMPUTATIONAL BIOLOGY OR GERIATRICS GERONTOLOGY OR ENGINEERING BIOMEDICAL OR FOOD SCIENCE TECHNOLOGY OR ENVIRONMENTAL STUDIES OR ENGINEERING ENVIRONMENTAL OR ENGINEERING ELECTRICAL ELECTRONIC OR HEALTH POLICY SERVICES OR TOXICOLOGY OR EDUCATION EDUCATIONAL RESEARCH OR NUTRITION DIETETICS OR SUBSTANCE ABUSE OR ECONOMICS OR MEDICINE RESEARCH EXPERIMENTAL OR STATISTICS PROBABILITY OR DEVELOPMENTAL BIOLOGY OR MEDICAL INFORMATICS OR SOCIOLOGY OR DENTISTRY ORAL SURGERY MEDICINE OR PSYCHOLOGY EXPERIMENTAL OR COMPUTER SCIENCE ARTIFICIAL INTELLIGENCE OR METEOROLOGY ATMOSPHERIC SCIENCES OR CHEMISTRY ANALYTICAL OR MEDICAL LABORATORY TECHNOLOGY OR CELL BIOLOGY OR DEMOGRAPHY OR BUSINESS FINANCE OR COMPUTER SCIENCE INTERDISCIPLINARY APPLICATIONS OR AUDIOLOGY SPEECH LANGUAGE PATHOLOGY OR PSYCHOLOGY DEVELOPMENTAL OR COMPUTER SCIENCE INFORMATION SYSTEMS OR PLANNING DEVELOPMENT)<br>Indexes=SCI-EXPANDED, SSCI, CPCI-S, CPCI-SSH Timespan=1900-2015
# 18	499	#17 OR #1<br>Indexes=SCI-EXPANDED, SSCI, CPCI-S, CPCI-SSH Timespan=1900-2015
#	487	#16 AND #15

For peer review only

17		Indexes=SCI-EXPANDED, SSCI, CPCI-S, CPCI-SSH Timespan=1900-2015
# 16	8,044	#14 OR #13 OR #12 OR #11 OR #10 OR #9 OR #8 OR #7 OR #6 OR #5 OR #4 OR #3 OR #2<br>Indexes=SCI-EXPANDED, SSCI, CPCI-S, CPCI-SSH Timespan=1900-2015
# 15	1,689,232	**TOPIC:** ((pediatric* OR paediatric* OR infant* OR child* OR baby OR toddler OR babies OR teen* OR adolescent*))<br>Indexes=SCI-EXPANDED, SSCI, CPCI-S, CPCI-SSH Timespan=1900-2015
# 14	130	**TOPIC:** ("Severity of Illness Index" and ((tool* or scor* or index* or indicator* or indice* or assessment* or instrument* or criteria or parameter*) SAME ((prevent* or reduc* or improv*) SAME (deteriorat* or mortality or death or outcome* or harm* or safety))))<br>Indexes=SCI-EXPANDED, SSCI, CPCI-S, CPCI-SSH Timespan=1900-2015
# 13	63	**TOPIC:** (("early medical intervention" SAME (tool* or scor* or index* or indicator* or indice* or assessment* or guide* or instrument* or criteria or parameter* or deteriorat* or mortality or death or monitor* or outcome* or harm* or safety)))<br>Indexes=SCI-EXPANDED, SSCI, CPCI-S, CPCI-SSH Timespan=1900-2015
# 12	28	**TOPIC:** ("early medical intervention" and ((prevent* or reduc* or improv*) SAME (deteriorat* or mortality or death or outcome* or harm* or safety)))<br>Indexes=SCI-EXPANDED, SSCI, CPCI-S, CPCI-SSH Timespan=1900-2015
# 11	1,206	**TOPIC:** ("early warning" SAME system* SAME (deteriorat* or mortality or death or outcome* or harm* or safety))<br>Indexes=SCI-EXPANDED, SSCI, CPCI-S, CPCI-SSH Timespan=1900-2015
# 10	2	**TOPIC:** ("SBAR technique")<br>Indexes=SCI-EXPANDED, SSCI, CPCI-S, CPCI-SSH Timespan=1900-2015
# 9	7	**TOPIC:** ("VitalPAC Early Warning Score")<br>Indexes=SCI-EXPANDED, SSCI, CPCI-S, CPCI-SSH Timespan=1900-2015
# 8	123	**TOPIC:** ("activation criteria")<br>Indexes=SCI-EXPANDED, SSCI, CPCI-S, CPCI-SSH Timespan=1900-2015
# 7	16	TS=("alert criteria")<br>Indexes=SCI-EXPANDED, SSCI, CPCI-S, CPCI-SSH Timespan=1900-2015
# 6	159	TS=("trigger tool*")<br>Indexes=SCI-EXPANDED, SSCI, CPCI-S, CPCI-SSH Timespan=1900-2015
#	45	TS=("track and trigger")

For peer review only

5		Indexes=SCI-EXPANDED, SSCI, CPCI-S, CPCI-SSH Timespan=1900-2015
# 4	15	TS=("track-and-trigger system") Indexes=SCI-EXPANDED, SSCI, CPCI-S, CPCI-SSH Timespan=1900-2015
# 3	6,100	TS=("Rapid Response") Indexes=SCI-EXPANDED, SSCI, CPCI-S, CPCI-SSH Timespan=1900-2015
# 2	604	TS=("early warning" SAME scor*) Indexes=SCI-EXPANDED, SSCI, CPCI-S, CPCI-SSH Timespan=1900-2015
# 1	88	TS=("Paediatric Early Warning" OR "Pediatric Early Warning" OR "Pediatric Advanced Warning Score" OR "Paediatric Advanced Warning Score" OR "neonatal early warning" OR "infant early warning" OR "pediatric rapid response" OR "Paedatric rapid response") Indexes=SCI-EXPANDED, SSCI, CPCI-S, CPCI-SSH Timespan=1900-2015

PUMA Supplementary searches

Search terms to use:
"Pediatric Early warning"
"Paediatric Early warning"
“Pediatric Rapid Response Team”
“Paediatric Rapid Response Team”
PEWS
“Paediatric trigger tools”
“Pediatric trigger tools”

For peer review only

Supplementary Table 2 - PICOS criteria for inclusion of studies

Question 1 – development / validation studies

Parameter	Inclusion criteria	Exclusion criteria
Patients	Children aged 0-18 who are in-patients in a hospital	Adult patients; children in emergency departments or neonatal unit
Intervention	Development or validation of a PTTT	Acuity or triage tools, tools developed for use in emergency departments
Comparator	Not applicable	
Outcomes	Mortality and critical events including: arrests, code calls, transfer to higher level of care (e.g., ICU/HDU), senior review, RRT/MET activation, acuity at PICU admission and critical interventions on the ward or PICU	
Study design	Chart or case reviews; cohort studies; case-control studies, observational studies	Reviews, editorials or opinion pieces

Question 2 – effectiveness studies

Parameter	Inclusion criteria	Exclusion criteria
Patients	Children aged 0-18 who are in-patients in a hospital	Adult patients<br>Children in emergency departments or neonatal unit
Intervention	Implementation of any 'paediatric early warning system' intervention (with or without a PTTT) – including implementing a new PTTT, RRT/MET implementation, educational initiatives or communications tools aimed at improving identification of deteriorating in-patients	Acuity or triage tools, tools developed for use in emergency departments, interventions whose purpose was not identification of deteriorating in-patients
Comparator	Not applicable	
Outcomes	Mortality and critical events including: arrests, code calls, transfer to higher level of care (e.g., ICU/HDU), senior review, RRT/MET activation, acuity at PICU admission and critical interventions on the ward or PICU	
Study design	Randomised controlled trials, non-randomised controlled trials, before-after studies (controlled or uncontrolled); interrupted time series studies	Reviews, editorials or opinion pieces

For peer review only

Supplementary Table 3 – Template Quality Assessment Forms

QUALITY ASSESSMENT FOR DEVELOPMENT AND VALIDATION STUDIES

Criteria		Yes (2)	Partial (1)	No (0)	N/A	Score
**1**	Is the hypothesis / aim / objective of the study clearly described?	Easily identified in introduction / method.	Vague / incomplete or found in other parts of paper (than introduction/method)	Aim / Objective no reported		
**2**	Was the score developed comprehensively?	Evidence base / Expert opinion / Delphi method	Decided within research team	No info / unclear		
**3**	Are the characteristics of the patients in the study clearly described?	Reproducible criteria used to categorise participants	Poorly define criteria / incomplete information	No baseline / demographic info		
**4**	Is the study design well described and appropriate?	Well described, easy to find in paper	Design not clearly described / design only partially answers the question	Design poorly described or does not answer study question		
**5**	Are the study sample representative of the intended population?	A full description of the target population is given with the sample selected in a non-biased manner	Sample selected from a known population however, selection strategy likely introduces bias but not enough to seriously distort results	Sample recruited from an unknown population in an opportunistic fashion		
**6**	Are population characteristics controlled for and adequately described?	Appropriate control at design/analysis stage	Incomplete control/description or not considered but unlikely to seriously influence results	Not controlled for and likely to seriously influence results		
**7**	Was compliance/use of the PEWS reliable?	Compliance / use was well described and reliably implemented	Compliance / use was not well described or not reliably implemented	Compliance / use was not reported		
**8**	Was consideration given for data collected at different times / sites	Well described reason why data was collected at different time points	Data was collected at different times due to specific opportunity	No explanation for data collection at different time points	Data was collected at the same time point	
**9**	Are the main findings clearly described?	Simple outcome data reported for all major findings	Incomplete or inappropriate descriptive statistics	No/inadequate descriptive statistics		
**10**	Are methods of analysis adequately described and appropriate?	Described and appropriate	Not reported but probably appropriate or some tests appropriate, some not	Methods not described and cannot be determined		
**11**	Are the conclusions supported by the results	All conclusions supported by data	Some of the major conclusions are supported by the data; some are not or speculative interpretations are not indicated as such	None/few of major conclusions supported by the data		
**12**	How was missing data handled	Missing data was reported and handled appropriately	Missing data was reported but unable to determine how it was handled or it wasn't handled appropriately	Missing data was not reported	No missing data	
					**Total**	

MAX. Score: 24

For peer review only

QUALITY ASSESSMENT FOR EFFECTIVENESS STUDIES

Criteria		Yes (2)	Partial (1)	No (0)	N/A	Score
1	Is the hypothesis / aim / objective of the study clearly described?	Easily identified in introduction / method.	Vague / incomplete or found in other parts of paper (than introduction/method)	Aim / Objective no reported		
2	Was the score developed comprehensively?	Evidence base / Expert opinion / Delphi method	Decided within research team	No info / unclear		
3	Are the characteristics of the patients in the study clearly described?	Reproducible criteria used to categorise participants	Poorly define criteria / incomplete information	No baseline / demographic info		
4	Is the study design well described and appropriate?	Well described, easy to find in paper	Design not clearly described / design only partially answers the question	Design poorly described or does not answer study question		
5	Are the study sample representative of the intended population?	A full description of the target population is given with the sample selected in a non-biased manner	Sample selected from a known population however, selection strategy likely introduces bias but not enough to seriously distort results	Sample recruited from an unknown population in an opportunistic fashion		
6	Was the PEWS well implemented?	Implementation was well reported and appropriately applied	Implementation was not well reported or not appropriate	No info / unclear		
7	Are population characteristics controlled for and adequately described?	Appropriate control at design/analysis stage	Incomplete control/description or not considered but unlikely to seriously influence results	Not controlled for and likely to seriously influence results		
8	Was compliance/use of the PEWS reliable?	Compliance / use was well described and reliably implemented	Compliance / use was not well described or not reliably implemented	Compliance / use was not reported		
9	Was consideration given for data collected at different times / sites	Well described reason why data was collected at different time points	Data was collected at different times due to specific opportunity	No explanation for data collection at different time points	Data was collected at the same time point	
10	Are the main findings clearly described?	Simple outcome data reported for all major findings	Incomplete or inappropriate descriptive statistics	No/inadequate descriptive statistics		
11	Are methods of analysis adequately described and appropriate?	Described and appropriate	Not reported but probably appropriate or some tests appropriate, some not	Methods not described and cannot be determined		
12	Are the conclusions supported by the results	All conclusions supported by data	Some of the major conclusions are supported by the data; some are not or speculative interpretations are not indicated as such	None/few of major conclusions supported by the data		
13	How was missing data handled	Missing data was reported and handled appropriately	Missing data was reported but unable to determine how it was handled or it wasn't handled appropriately	Missing data was not reported	No missing data	
					**Total**	

MAX. Score: 26

Supplementary Table 4 –Validation papers excluded from analysis

PTTT	First author, year	Country	Study population	Study design	Number of centres	PTTT used in practice?	Internal / external validation study?	Outcome measures	Sample size	Score or trigger?	Study overview and reason for exclusion from validation results	Quality score (max = 24)
Modified Brighton PEWS (a)	Garlick 2013[20]	US	All in-patients (MET calls only)	Case-control study (retrospective)	1	N	Ext	Transfer to PICU	267 (116 cases)	S	Describes review of MET calls (n=267) to evaluate predictive ability of Modified Brighton PEWS tool for identifying children requiring transfer to PICU (n=116). Results presented in terms of association between PEWS and odds of transfer to higher level of care – no evaluation of performance characteristics such as AUROC, sensitivity or specificity.	8
	Medar 2015[21]	Unclear	RRT calls only	Chart review (retrospective)	1	NR	Ext	RRT call	61	S	Describes retrospective review of RRT calls (n=61) to evaluate Modified Brighton PEWS at time of admission and time of RRT call. Report higher median PEWS score for patients at time of RRT call compared to admission. No evaluation of performance characteristics such as AUROC, sensitivity or specificity.	6
Texas Children's Hospital (TCH) PAWS	Bell 2013[22]	US	General medical ward & two specialist units	Chart review (retrospective)	1	Y	Int	Other validated scales (e.g., Glasgow Coma Scale)	150	S	Describes development and implementation of the TCH PAWS tool in three wards of a specialist paediatric unit in the US. TCH PAWS amended locally from the Brighton PEWS. Reports on internal reliability (correlation coefficients between 3 categories of the score) and inter-rater reliability of scoring among nurses. Also compares scores on sub-categories to other measures, e.g., the Behavioural sub-score is compared to the Glasgow Coma Scale. No evaluation of performance characteristics such as AUROC, sensitivity or specificity.	12
Cardiac Children's Hospital Early Warning Score (C-CHEWS)	McLellan 2013[23]	US	Cardiac unit	Tool development	1	Y	Int	Cardiac ICU transfer	27	S	Describes the development and implementation of a modified version of the Children's Hospital Early Warning score for cardiac patients. Results focus on tool modification and implementation challenges – no evaluation of performances characteristics such as AUROC, sensitivity or specificity. Validation of the tool described in a separate paper.	9
Burn-specific PEWS	Rahman 2014[24]	US	Specialist burn unit	Chart review (retrospective)	1	Y	Int	Burn injuries	50	S	Conference abstract only. Describes development and implementation of a modified version of the Brighton PEWS, for use with in-patients with burn injuries. Analysis of 50 randomly selected charts – results focus on compliance with scoring and relationship between PTTT score and extent of burn injuries. No evaluation of performance characteristics such as AUROC, sensitivity or specificity.	13

For peer review only

Bedside Paediatric Early Warning Score (PEWS)	Hopkins 2013[25]	US	All in-patients (code blue and RRT calls only)	Chart review (retrospective)	1	N	Ext	PICU transfer and critical intervention in PICU among RRT and code calls	113 (64 cases)	S	Conference abstract only. Describes retrospective chart review of code blue and RRT calls over a year – Bedside PEWS scores calculated and comparisons drawn between patients eventually transferred to PICU and those who stayed on ward. Preliminary analysis given in terms of mean PEWS scores for different groups – no evaluation of performance characteristics such as AUROC, sensitivity or specificity.	6
	Gawronski 2013[26]	Italy	Bone marrow transplant unit	Case-control study (retrospective)	1	N	Ext	Urgent PICU transfer, PICU consult or death	21 (11 cases)	S	Conference abstract only. Describes case-control study evaluating Bedside PEWS in an Italian bone marrow transplant unit, in relation to urgent PICU transfers or consultations. Preliminary analysis only – comparison of mean PTTT scores for cases and controls. No evaluation of performance characteristics such as AUROC, sensitivity or specificity.	6
Bristol Paediatric Early Warning Tool (PEWT)	Haines 2006[12]	UK	All in-patients	Chart review (retrospective)	1	Y	Int	Transfer to PICU or HDU	360 (180 cases)	T	Describes development and piloting of the Bristol PEWT in a UK tertiary centre. Only included children who would have triggered the pilot version of the tool (n=360) and then identified PICU or HDU transfers from this population. Paper presents specificity and sensitivity outcomes but they are incorrectly calculated, so results not included in analysis.	9
Modified Bristol PEWT (a)	Sefton 2014[27]	UK	All in-patients	Chart review (retrospective)	1	Y	Int	Transfer to PICU, cardiac / respiratory arrest or unexpected death	Unclear	T	Conference abstract only. Describes a retrospective review of 5 years of data from locally implemented PTTT in a UK tertiary centre, presenting a multiple regression model identifying seven components (including age) most strongly associated with subsequent adverse event if triggered. Of the six clinical elements, all were associated with increased odds of an adverse event, except nurse concern which was significantly associated with decreased odds of an adverse event. No evaluation of overall PTTT performance characteristics such as AUROC, sensitivity or specificity.	10

All studies conducted in a specialist / tertiary centre.

Studies classified as internal validation if the setting for the study was the same hospital and same research team as those who developed the score. Studies classified as external validation if the score was tested in a different centre and by a different research team to those who developed it.

AUROC, area under the receiver operator characteristic curve; Ext, external validation ; HFNC, high flow nasal cannula; Int, Internal validation; NPV, negative predictive value; PHDU, paediatric high-dependency unit; PICU, paediatric intensive care unit ; PPV, positive predictive value; PTTT, paediatric track and trigger tool; RRT, rapid response team; S, score; T, trigger; UK, United Kingdom; US, United States;

For peer review only

Supplementary Table 5 – Effectiveness papers excluded from analysis

First author, year	Intervention				PTTT	Country	Number of centres	Specialist unit?	Existing RRT / MET?	Population	Study design	Study duration in months (before & after intervention)	Description and reason for excluding from analysis	Quality score (max = 26)
	Implemented a new PTTT	Implemented new RRT / MET	Modified escalation process	Staff training / education										
Mistry 2006[51]	✓	✓		✓	Paediatric Rapid Response Team activation criteria*	US	1	Y	N	All in-patients	Uncontrolled before-after study (prospective)	11 (6 before, 5 after)	Describes implementation of a PRRT with calling criteria (not defined). Looked at impact on mortality, cardiac arrests and PICU outcomes among PICU transfers. Reports absolute decreases in numbers of deaths and arrests post-intervention, but no denominator data provided or further statistical details given.	3
Demmel 2010[52]	✓				Modified Brighton PEWS (e)	US	1	Y	Y	Haematology / oncology patients	Uncontrolled before-after study (prospective)	Unclear (unclear, 8 after)	Implemented a locally modified version of the Brighton PEWS in a specialist haematology / oncology unit. Discusses challenges in the development and implementation of the tool. Refers to number of days between cardiopulmonary arrests being 299 immediately before implementation, and 1,053 days eight months after implementation – however, no denominator data or further statistical details given.	8
Sandhu 2010[53]		✓			Unclear	UK	1	Y	N	Unclear	Uncontrolled before-after study (retrospective)	Unclear (unclear, 3 months)	Conference abstract only. Reported implementing an 'outreach response team' alongside an existing 'paediatric early warning tool' (unclear which tool) in a UK tertiary centre. Reference to comparable triggering rate of PTTT before (28% of patients) and after (28% of patients) piloting the outreach team, and 2 arrests before piloting, and 0 after – but no denominator data or further statistical details given.	8
Randhawa 2011[54]	✓		✓	✓	Brighton PEWS	US	1	Y	Y	All in-patients	Uncontrolled before-after study (prospective)	Unclear	Describes implementation of the Brighton PEWS in a specialist paediatric centre. Details various cycles of change during implementation of the tool across different wards, and efforts at staff education. Reports reduction in rate of cardiopulmonary arrests post-intervention, but no absolute numbers, denominator data or further statistical details given.	12

For peer review only - http://bmjopen.bmj.com/site/about/guidelines.xhtml

Camacho 2011[55]	✓				Modified Brighton PEWS (a) †	US	1	Y	NR	Cardiac and renal patients	Uncontrolled before-after study (prospective)	8 (3 before, 5 after)	Conference abstract only. Reported piloting and modifying Tucker's modified Brighton PEWS for specialist cardiac and renal population. Unclear if RRT/MET in place. Referred to there being 5 code calls in the quarter (3 months) before implementation, and 0 in the following 5 months. However, no denominator data or further statistical details given.	8
Heyden 2012[56]	✓	✓			Paediatric Rapid Response Team activation criteria*	US	1	Y	N	All in-patients	Uncontrolled before-after study (retrospective)	72 (24 before, 48 after)	Conference abstract only. Describes implementation of an RRT in a US tertiary centre, with an associated 'broad calling criteria' (limited details given). Reports number of cardiac arrests on ward and PICU before and after intervention, and refers to increase in RRT calls over time. No denominator data or further statistical details given.	7
Somberg 2013	✓	✓			Unclear	US	1	N	N	All in-patients	Uncontrolled before-after study (unclear)	Unclear	Conference abstract only. Reported developing and implementing a PTTT (tool not named) and RRT for a paediatric unit in a community hospital. Reference to no intubation or code calls since intervention, but no pre-intervention comparison, time frames, denominator data or further statistical details given.	2
Norville 2013[57]	✓				Texas Children's Hospital (TCH) Paediatric Advanced Warning Score (PAWS)†	US	1	Y	Y	Bone marrow transplant patients	Uncontrolled before-after study (unclear)	23 (12 before, 11 after)	Conference abstract only. Describes implementation of TCH PAWS, with amended algorithm for specialist bone marrow transplant unit. Looked at impact on code calls and RRT calls – refers to 3 code calls and 18 RRT calls pre-intervention, compared to 0 codes and 25 RRT calls post-intervention. No denominator data or further statistical details given.	5
Ambati 2014[58]				✓	Not applicable	US	1	Y	Y	Unclear	Uncontrolled before-after study (unclear)	48 (12 before, 36 after)	Conference abstract only. Reported effect of implementing a "simulation based curriculum" for clinical staff on subsequent RRT utilisation. Reference to increase in RRT calls year on year post implementation, but no denominator data or further statistical details given.	3
Ocholi 2014[59]	✓				Bedside Paediatric Early Warning Score (PEWS)	UK	1	Y	N	Unclear	Uncontrolled before-after study (unclear)	12 months (6 before, 6 after)	Conference abstract only. Describes implementation of Bedside PEWS in a UK tertiary centre. Looked at impact of intervention on ward outcomes and outcomes of children transferred to PICU. Reference to impact of tool on number of 'adverse incidents' (not defined) on the ward and median length of stay in PICU among PICU transfers, but no denominator data or further statistical details given.	6
Fenix 2016[39]	✓			✓	Unclear	US	1	Y	NR	Two general paediatric wards	Uncontrolled before-after study (retrospective)	46 months (16 before, 30 after)	Conference abstract only. Describes implementation of a 'Situational Awareness' tool, with integrated PTTT (unclear which tool) in a tertiary centre. Retrospective review of rates of Critical Deterioration (CD) events on two of seven general paediatric wards. Reports a significant decrease in trend and trajectory of CD events post-implementation, but no event numbers, denominator data or further statistical details given.	6

For peer review only

* Indicates PTTT not fully described or validated in the published literature

† PTTT modified by local team, but exact modifications not described

MET, medical emergency team; PICU, paediatric intensive care unit; PTTT, paediatric track and trigger tool; RRT, rapid response team; UK, United Kingdom; US, United States.

For peer review only

PRISMA 2009 Checklist

Section/topic	#	Checklist item	Reported on page #
**TITLE**			
Title	1	Identify the report as a systematic review, meta-analysis, or both.	1
**ABSTRACT**			
Structured summary	2	Provide a structured summary including, as applicable: background; objectives; data sources; study eligibility criteria, participants, and interventions; study appraisal and synthesis methods; results; limitations; conclusions and implications of key findings; systematic review registration number.	2-3
**INTRODUCTION**			
Rationale	3	Describe the rationale for the review in the context of what is already known.	5-6
Objectives	4	Provide an explicit statement of questions being addressed with reference to participants, interventions, comparisons, outcomes, and study design (PICOS).	6
**METHODS**			
Protocol and registration	5	Indicate if a review protocol exists, if and where it can be accessed (e.g., Web address), and, if available, provide registration information including registration number.	7
Eligibility criteria	6	Specify study characteristics (e.g., PICOS, length of follow-up) and report characteristics (e.g., years considered, language, publication status) used as criteria for eligibility, giving rationale.	7-8; Supp table 2
Information sources	7	Describe all information sources (e.g., databases with dates of coverage, contact with study authors to identify additional studies) in the search and date last searched.	7
Search	8	Present full electronic search strategy for at least one database, including any limits used, such that it could be repeated.	Supp table 1
Study selection	9	State the process for selecting studies (i.e., screening, eligibility, included in systematic review, and, if applicable, included in the meta-analysis).	7-8
Data collection process	10	Describe method of data extraction from reports (e.g., piloted forms, independently, in duplicate) and any processes for obtaining and confirming data from investigators.	7-8
Data items	11	List and define all variables for which data were sought (e.g., PICOS, funding sources) and any assumptions and simplifications made.	7
Risk of bias in individual studies	12	Describe methods used for assessing risk of bias of individual studies (including specification of whether this was done at the study or outcome level), and how this information is to be used in any data synthesis.	8; Supp table 3
Summary measures	13	State the principal summary measures (e.g., risk ratio, difference in means).	8
Synthesis of results	14	Describe the methods of handling data and combining results of studies, if done, including measures of consistency (e.g., I^2) for each meta-analysis.	NA

For peer review only - http://bmjopen.bmj.com/site/about/guidelines.xhtml

PRISMA 2009 Checklist

Section/topic	#	Checklist item	Reported on page #
Risk of bias across studies	15	Specify any assessment of risk of bias that may affect the cumulative evidence (e.g., publication bias, selective reporting within studies).	NA
Additional analyses	16	Describe methods of additional analyses (e.g., sensitivity or subgroup analyses, meta-regression), if done, indicating which were pre-specified.	NA
**RESULTS**			
Study selection	17	Give numbers of studies screened, assessed for eligibility, and included in the review, with reasons for exclusions at each stage, ideally with a flow diagram.	9
Study characteristics	18	For each study, present characteristics for which data were extracted (e.g., study size, PICOS, follow-up period) and provide the citations.	9, Tables 2-4
Risk of bias within studies	19	Present data on risk of bias of each study and, if available, any outcome level assessment (see item 12).	Tables 2-4
Results of individual studies	20	For all outcomes considered (benefits or harms), present, for each study: (a) simple summary data for each intervention group (b) effect estimates and confidence intervals, ideally with a forest plot.	Tables 3-4
Synthesis of results	21	Present results of each meta-analysis done, including confidence intervals and measures of consistency.	NA
Risk of bias across studies	22	Present results of any assessment of risk of bias across studies (see Item 15).	NA
Additional analysis	23	Give results of additional analyses, if done (e.g., sensitivity or subgroup analyses, meta-regression [see Item 16]).	NA
**DISCUSSION**			
Summary of evidence	24	Summarize the main findings including the strength of evidence for each main outcome; consider their relevance to key groups (e.g., healthcare providers, users, and policy makers).	16-20
Limitations	25	Discuss limitations at study and outcome level (e.g., risk of bias), and at review-level (e.g., incomplete retrieval of identified research, reporting bias).	16-20
Conclusions	26	Provide a general interpretation of the results in the context of other evidence, and implications for future research.	16-20
**FUNDING**			
Funding	27	Describe sources of funding for the systematic review and other support (e.g., supply of data); role of funders for the systematic review.	21

From: Moher D, Liberati A, Tetzlaff J, Altman DG, The PRISMA Group (2009). Preferred Reporting Items for Systematic Reviews and Meta-Analyses: The PRISMA Statement. PLoS Med 6(7): e1000097. doi:10.1371/journal.pmed1000097

For more information, visit: www.prisma-statement.org.

For peer review only - http://bmjopen.bmj.com/site/about/guidelines.xhtml

PRISMA 2009 Checklist

For peer review only

For peer review only - http://bmjopen.bmj.com/site/about/guidelines.xhtml
